# Genomic Analyses Unveil Helmeted Guinea Fowl (*Numida meleagris*) Domestication in West Africa

**DOI:** 10.1093/gbe/evab090

**Published:** 2021-05-01

**Authors:** Quan-Kuan Shen, Min-Sheng Peng, Adeniyi C Adeola, Ling Kui, Shengchang Duan, Yong-Wang Miao, Nada M Eltayeb, Jacqueline K Lichoti, Newton O Otecko, Maria Giuseppina Strillacci, Erica Gorla, Alessandro Bagnato, Olaogun S Charles, Oscar J Sanke, Philip M Dawuda, Agboola O Okeyoyin, John Musina, Peter Njoroge, Bernard Agwanda, Szilvia Kusza, Hojjat Asadollahpour Nanaei, Rana Pedar, Ming-Min Xu, Yuan Du, Lotanna M Nneji, Robert W Murphy, Ming-Shan Wang, Ali Esmailizadeh, Yang Dong, Sheila C Ommeh, Ya-Ping Zhang

**Affiliations:** 1 State Key Laboratory of Genetic Resources and Evolution, Yunnan Laboratory of Molecular Biology of Domestic Animals, Kunming Institute of Zoology, Chinese Academy of Sciences, Kunming, Yunnan, China; 2 Sino-Africa Joint Research Center, Chinese Academy of Sciences, Nairobi, Kenya; 3 Kunming College of Life Science, University of Chinese Academy of Sciences, Kunming, China; 4 Centre for Biotechnology Research, Bayero University, Kano, Nigeria; 5 Dana-Farber Cancer Institute, Harvard Medical School, Boston, Massachusetts, USA; 6 Nowbio Biotechnology Company, Kunming, China; 7 Faculty of Animal Science and Technology, Yunnan Agricultural University, Kunming, China; 8 Department of Animal breeding and Reproduction Technology, College of Animal Production, University of Bahri, Khartoum, Sudan; 9 State Department of Livestock, Ministry of Agriculture Livestock Fisheries and Irrigation, Nairobi, Kenya; 10 Department of Veterinary Medicine, Università degli Studi di Milano, Italy; 11 Department of Veterinary Medicine, University of Ibadan, Nigeria; 12 Taraba State Ministry of Agriculture and Natural Resources, Jalingo, Nigeria; 13 Department of Veterinary Surgery and Theriogenology, College of Veterinary Medicine, University of Agriculture, Makurdi, Nigeria; 14 National Park Service Headquarter, Federal Capital Territory, Abuja, Nigeria; 15 Department of Zoology, National Museums of Kenya, Nairobi, Kenya; 16 Centre for Agricultural Genomics and Biotechnology, University of Debrecen, Debrecen, Hungary; 17 Department of Animal Science, Faculty of Agriculture, Shahid Bahonar University of Kerman, Iran; 18 Centre for Biodiversity and Conservation Biology, Royal Ontario Museum, Toronto, Ontario, Canada; 19 Howard Hughes Medical Institute, University of California Santa Cruz, California, USA; 20 Department of Ecology and Evolutionary Biology, University of California Santa Cruz, California, USA; 21 College of Biological Big Data, Yunnan Agriculture University, Kunming, China; 22 State Key Laboratory for Conservation and Utilization of Bio-Resources in Yunnan, Yunnan Agricultural University, Kunming, China; 23 Key Laboratory for Agro-Biodiversity and Pest Control of Ministry of Education, Yunnan Agricultural University, Kunming, China; 24 Institute of Biotechnology Research, Jomo Kenyatta University of Agriculture and Technology, Nairobi, Kenya; 25 State Key Laboratory for Conservation and Utilization of Bio-Resource in Yunnan, Yunnan University, Kunming, China; 26 Center for Excellence in Animal Evolution and Genetics, Chinese Academy of Sciences, Kunming, China

**Keywords:** guineafowl, genome, domestication, Africa, selection, breed

## Abstract

Domestication of the helmeted guinea fowl (HGF; *Numida meleagris*) in Africa remains elusive. Here we report a high-quality de novo genome assembly for domestic HGF generated by long- and short-reads sequencing together with optical and chromatin interaction mapping. Using this assembly as the reference, we performed population genomic analyses for newly sequenced whole-genomes for 129 birds from Africa, Asia, and Europe, including domestic animals (*n* = 89), wild progenitors (*n* = 34), and their closely related wild species (*n* = 6). Our results reveal domestication of HGF in West Africa around 1,300–5,500 years ago. Scanning for selective signals characterized the functional genes in behavior and locomotion changes involved in domestication of HGF. The pleiotropy and linkage in genes affecting plumage color and fertility were revealed in the recent breeding of Italian domestic HGF. In addition to presenting a missing piece to the jigsaw puzzle of domestication in poultry, our study provides valuable genetic resources for researchers and breeders to improve production in this species.

## Introduction

The domestication of animals and plants in Africa provides valuable agricultural resources for human societies (Fuller and Hildebrand 2013). In contrast to many plants, only two animals were domesticated in Africa: donkey and HGF (*Numida meleagris*) (Gifford-Gonzalez and Hanotte 2011). After domestication from wild HGF, the domestic HGF spread widely across sub-Saharan Africa, and then underwent a global expansion due to human translocations and its ability to adapt to a wide range of habitats (Blench and MacDonald 2000; Roberts 2002). Nowadays, domestic HGFs together with gooses account for 1.99% of the world’s poultry population (chickens 91.18%, ducks 5.60%, and turkeys 1.22%; FAOSTAT, last accessed December 22, 2020) and are widely valued as source of meat, eggs, and feathers. Production is increasing rapidly. In addition to its roles in natural control of the deer ticks, which are vectors of the Lyme disease (Duffy et al. 1992), domestic HGF also serves as a physiological animal model in studying the neuromuscular, mechanical, and energetic strategies for locomotion.

Significance

The helmeted guinea fowl (HGF, *Numida meleagris*) is the only bird domesticated in sub-Saharan Africa. Its domestication and evolution remains elusive since Charles Darwin. In this study, we provided valuable genomic resource for this bird and revealed the domestication of HGF in West Africa. We also identified selective signals involved in early domestication and recent breeding process. The future integration of genomic evidence from animals, plants, and human populations has potential to provide insights into the dispersal of agriculture in Africa.

Despite the economic and scientific importance of domesticated HGF, its domestication and evolution remain poorly understood. Two competitive hypotheses exist for the single-origin mode. Darwin proposed that domestic HGF evolved from wild guinea fowl in East Africa (Darwin 1883). In contrast, archaeological, linguistic, and ethnographic evidence has pointed to domestication in West Africa (Blench and MacDonald 2000). Recent artistic and osteological evidence suggest dual origins less than 2,000 years ago (YBP) in both West (Mali) and East (Sudan) Africa (Andah et al. 2014). However, this dating is younger than hieroglyph records in Egypt, which date back to around 2400 BC, and even younger than introduction of this bird into Europe around 500 BC by ancient Greeks (Roberts 2002), although there was no indication that those birds were domesticated or wild. Still, the scanty records from archaeology and history, together with the osteological similarities between HGF and the francolin species or even domestic chicken (MacDonald 1992), make hypotheses about the domestication of HGF await testing.

Genetic approaches have been applied to trace the history of domestication for poultries during the past decade (Dalloul et al. 2010; Shapiro et al. 2013; Lu et al. 2015; Zhou et al. 2018; Wang et al. 2020). Previous genetic diversity studies based on mitochondrial DNA (Adeola et al. 2015; Murunga et al. 2018) and microsatellite markers (Kayang et al. 2010; Botchway et al. 2013) revealed an absence of genetic structuring in populations of African domestic HGF, implying a recent domestication accompanied with rapid subsequent dispersal in Africa. Most recently, Vignal et al. assembled the reference genome of HGF (NumMel1.0; accession in NCBI: GCA_002078875.2) and then conducted population genomic analyses with whole-genome sequencing (WGS) for pools of individuals from wild and domestic populations from Europe and Africa to investigate domestication of HGF (Vignal et al. 2019). However, the lack of genomic data of samples from East Africa—one candidate domestication center (Darwin 1883; Andah et al. 2014) hampers hypotheses testing.

Herein, we employ the combined strategy based on high-depth PacBio long-read sequencing, BioNano optical mapping, and high-throughput/resolution chromosome conformation capture (Hi-C) scaffolding to obtain a de novo assembly for HGF. This improved reference provides the backbone for WGS for a total of 129 guinea fowl samples from Africa, Asia, and Europe. These data serve to test the competing hypotheses on the domestication of HGF and further explore genetic diversity and population history. Our results fail to reject the hypothesis of HGF domestication in West Africa.

## Results

### An Improved Helmeted Guineafowl Genome Assembly

**Fig. 1.**
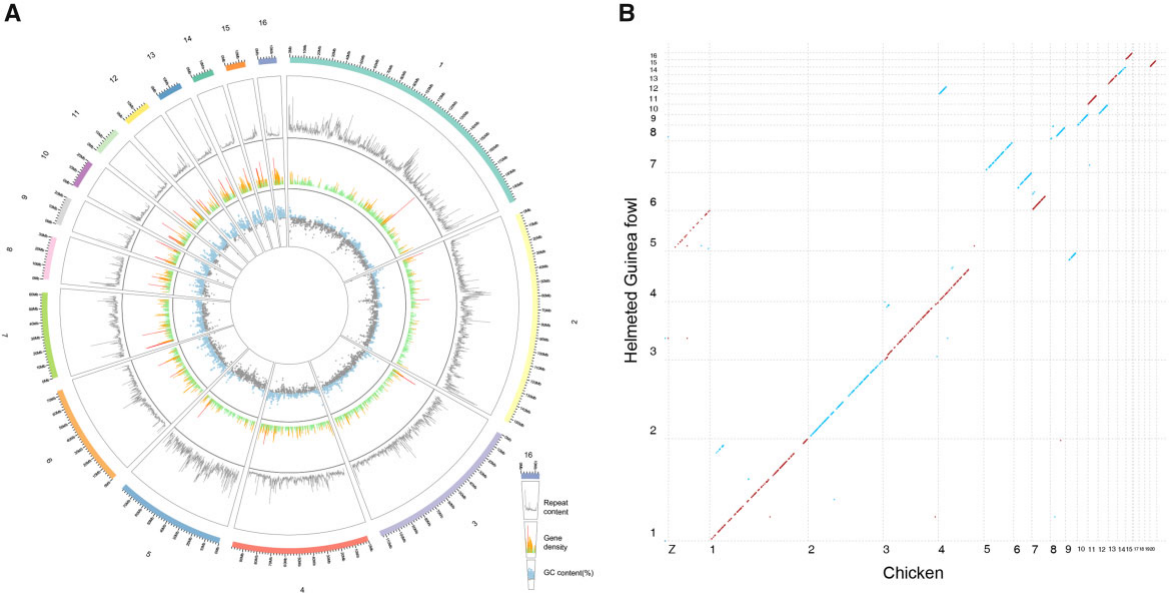
Genome architecture of the HGFv1 assembly. (*A*) Circos plot of HGFv1 assembly, repeat content, gene density, and GC content (%). The 1–16 scaffolds, representing 90% of the total length of 1,127 scaffolds, are shown as the outermost tracks. The repeat content is indicated by the gray line. Regions within the gene density of more than ten genes are shown as red spikes, whereas those with five to ten genes are indicated by orange spikes. Green spikes represent regions with fewer than five genes. Regions of the genome with GC content higher than average (37.54%) are shown in light blue. All data were plotted in 100-kb windows. (*B*) Dot plots showing synteny between the HGFv1 assembly and chicken reference genome GRCg6a (GCA_000002315.5). The sky-blue dots represent the accordant alignments, whereas the firebrick dots are reverse adjustments.

We sequenced multiple samplings of DNA and RNA (supplementary table S1, Supplementary Material online). Using the strategy based on PacBio long-reads sequencing, BioNano optical mapping, and Hi-C scaffolding, we assembled 1,127 scaffolds with total length of 1049.9 Mb to generate assembly HGFv1 (supplementary fig. S1, Supplementary Material online), in which the first 16 scaffolds accounted for 90% (949.6 Mb) of the total assembly, approaching near-chromosome level (fig. 1*A*). We further computed synteny between the HGFv1 assembly and the chicken reference GRCg6a to show HGFv1 scaffold 5 corresponding to GRCg6a chromosome Z; scaffold 4 to chromosomes 4q and 9; scaffold 6 to chromosomes 6 and 7 (fig. 1*B*). The results were in agreement with the karyotypes for HGF and chicken (Shibusawa et al. 2002).

Comparing the HGFv1 assembly with the reference NumMel1.0 (Vignal et al. 2019), the contig N50 increases ∼ 62 times (14.17 vs. 0.23 Mb) whereas the gaps reduce ∼15 Mb (5.38 vs. 20.12 Mb) (table 1). BUSCO assessments for HGFv1 showed that 94.5% of the 4,915 expected avian genes (aves_odb9) were identified as complete. The LAI of 10.29 suggested that the assembly continuity of HGFv1 reaches the reference criteria (Ou et al. 2018). In addition, 2.05 Gb of RNA-seq data obtained from pancreas, hypothalamus, bone marrow, and bursa (Darris et al. 2015) were mapped onto the assemblies of HGFv1 and NumMel1.0, respectively. Overall, 92.28% and 88.48% of the RNA-seq reads could be mapped to the two assemblies, respectively. Around 13.64% of the HGFv1 assembly were characterized as repeats (details in Materials and Methods). Analyses identified 15,173 protein-coding genes, in which 14,373 were functionally annotated. We classified noncoding RNA into rRNA (∼45 kb), snRNA (∼32 kb), tRNA (∼22 kb), and miRNA (∼15 kb).

**Table 1:** Quality Metrics for HGFv1 Assembly Compared with Previous Poultry Genome Assemblies

Genome Assembly	Largest Contig (Mb)a	N50 Contigs (Mb)a	Largest Scaffold (Mb)a	N50 Scaffolds (Mb)a	N gaps (Mb)b	Buscoc	LAId
*Numida meleagris* (HGF) [HGFv1]	42.03	14.17	199.08	98.08	5.38	C: 94.5% [S: 93.3%, D: 1.2%], F: 3.1%, M: 2.4%	10.29
*N. meleagris* (HGF) [NumMel1.0]	1.66	0.23	194.43	97.48	20.12	C: 94.7% [S: 93.7%, D: 1.0%], F: 3.1%, M: 2.2%	N/A
*Gallus gallus* (chicken) [GRCg6a]	65.77	17.49	197.6	91.31	9.78	C: 91.1% [S: 90.0%, D: 1.1%], F: 5.4%, M: 3.5%	7.44
*Anas platyrhynchos* (mallard) [IASCAAS_PekingDuck_PBH1.5]	0.54	0.03	202.84	76.12	2.91	C: 87.2% [S: 85.9%, D: 1.3%], F: 6.7%, M: 6.1%	7.09
*Meleagris gallopavo* (turkey) [Turkey_5.0]	0.26	0.02	190.65	59	35.29	C: 90.5% [S: 89.5%, D: 1.0%], F: 5.4%, M: 4.1%	N/A
*Columba livia* (rock pigeon) [Cliv_2.1]	0.25	0.02	94.47	14.23	20.83	C: 93.8% [S: 92.9%, D: 0.9%], F: 4.0%, M: 2.2%	N/A
*Coturnix japonica* (Japanese quail) [*Coturnix japonica* 2.0]	5.28	0.55	175.65	82.19	10.39	C: 94.6% [S: 93.5%, D: 1.1%], F: 3.2%, M: 2.2%	N/A
*Anser cygnoides* (swan goose) [AnsCyg_PRJNA183603_v1.0]	0.2	0.02	24.05	5.2	35.37	C: 93.4% [S: 92.7%, D: 0.7%], F: 3.9%, M: 2.7%	N/A

a Statistics were calculated by stats.sh script contained in BBMap (v. 38.45).

b Sum of all “*N*” nucleotides in the genome assembly.

c Busco (v. 3.0.2) assessment according to Aves obd9 (*n* = 4,915). C: Complete; S: complete and single copy; D: complete and duplicated; F: fragmented; M: missing.

d Evaluation of assembly continuity for repetitive sequences via the LAI. “N/A” means that intact LTR-RT content and total LTR sequence content are too low to calculate LAI.

### Genome Variation

**Fig. 2.**
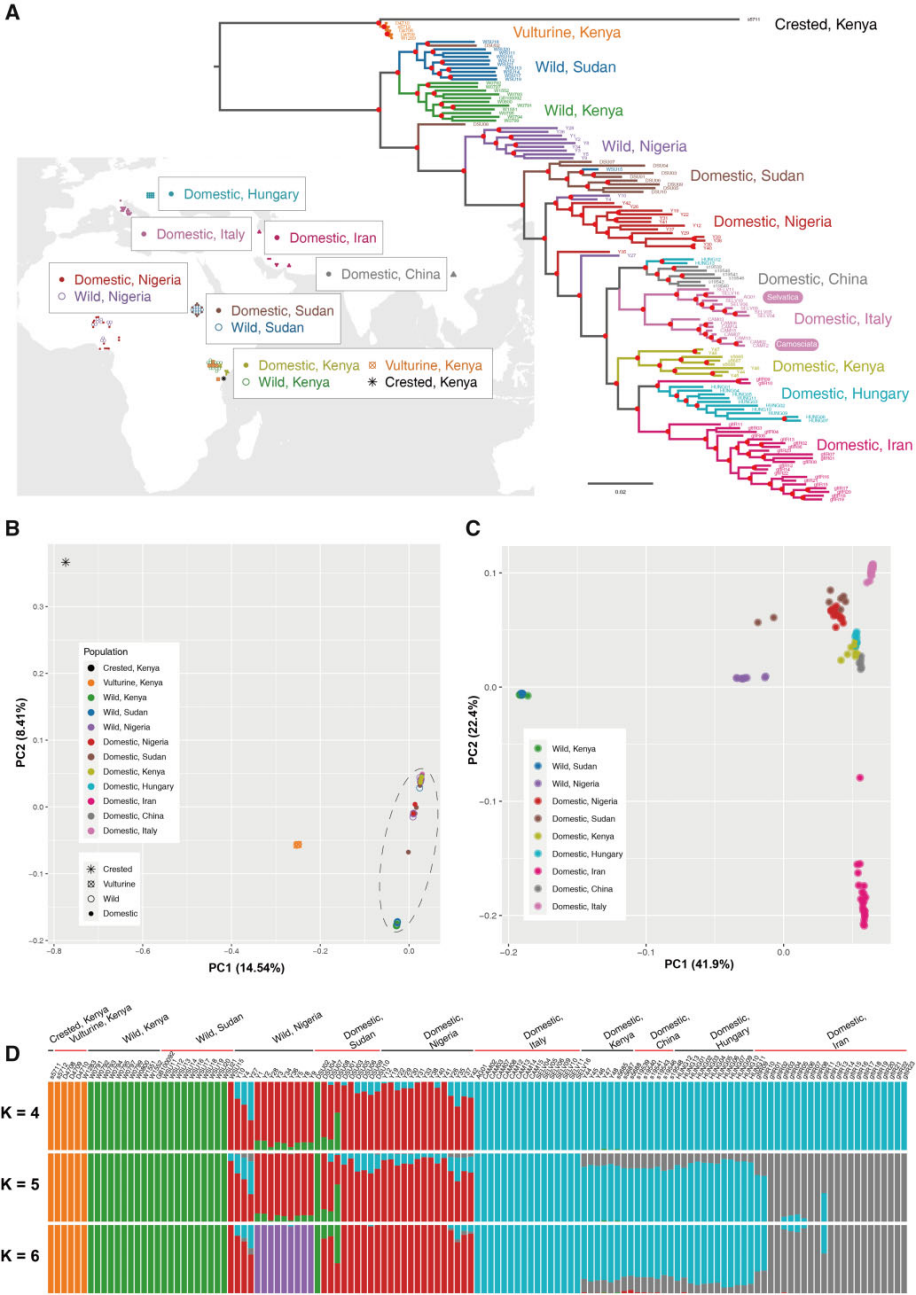
Geographic distribution, phylogeny, and population structure. (*A*) Geographic distribution of guinea fowl samples showing on the world map (downloaded from http://bzdt.ch.mnr.gov.cn). Phylogenetic tree of 89 domestic HGF, 34 wild HGF, and 6 wild relatives (as the outgroup) constructed using 8,662,417 SNPs with FastTree. The red solid dots indicate nodes with more than 85% of 100 bootstraps. (*B*) PCA for HGF and the outgroup based on 8,662,417 biallelic SNPs. (*C*) PCA for HGF without the outgroup based on 4,695,945 SNPs, because the SNPs only in the outgroup were excluded. (*D*) Model-based clustering analysis with *K* = 4, 5, and 6. The lowest cross-validation error value was observed when *K* = 5.

A total of 129 genomes from 89 domestic HGF, 34 wild HGF, 5 vulturine guinea fowl (*Acryllium vulturinum*), and 1 crested guinea fowl (*Guttera pucherani*) samples were resequenced (fig. 2*A* and supplementary table S2, Supplementary Material online). All Illumina reads were mapped to the HGFv1 assembly to an average depth of 18× (ranging from 10.4 to 42.2). Joint variant calling produced 44,035,924 SNPs and 4,214,076 InDels (details in Materials and Methods). Among them, a total of 17,334,420 SNPs and 1,591,307 InDels existed in HGF populations. For convenience, subsequent population genomic analyses used the biallelic SNPs from the 30 autosomal scaffolds with more than 20,000 markers only, which accounted for more than 97.5% length of the assembly.

### Phylogeny and Population Structure

When using vulturine guinea fowl and crested guinea fowl as outgroup taxa, phylogenetic trees constructed with FastTree and RAxML showed that the clade of wild HGF from East Africa (Sudan and Kenya) diverged first, followed by the split of the wild HGF from Nigeria and all the domestic HGF (fig. 2*A* and supplementary fig. S2, Supplementary Material online). Within the domestic HGF, the early branches consist of samples from Sudan and Nigeria. Kenyan domestic samples clustered with non-African domestic HGF samples. This likely reflected Kenyan domestic HGF samples were collected in the coast region receiving gene flows from region(s) out of Africa.

The PCA and ADMIXTURE analyses based on 8,662,417 SNPs after pruning LD agreed with the phylogenetic trees. The early split between HGF and outgroup taxa was indicated in PC1 (fig. 2*B*). When considering HGF only, PC1 separated wild and domestic samples (fig. 2*C*). Nigerian wild HGF samples presented closer genetic relationship to the domestic individuals. Kenyan domestic HGF clustered with other non-African HGF samples, rather than wild or domestic HGF from Africa. The *K* = 2 clustering in ADMIXTURE (supplementary fig. S3, Supplementary Material online) inferred the ancestry component for all the outgroup taxa and wild HGF. The *K* = 3 splits the outgroups, wild and domestic HGF. The *K* = 4 dissected the differentiation between African and non-African domestic HGF (fig. 2*D*). When *K* = 5, which had the lowest cross-validation error value (supplementary fig. S3, Supplementary Material online), a dominant component in the Iranian domestic HGF was detected. The *K* = 6 and *K* = 7 divisions distinguished the ancestry-component for wild HGF from Nigeria and Sudan, respectively (supplementary fig. S3, Supplementary Material online).

The phylogeny, PCA, and ADMIXTURE identified ten individuals as outliers. Three Nigerian (Y10, Y4, and Y27) and one Sudanese (WSU15) “wild” HGF individuals clustered with domestic samples, which potentially indicated feral domesticated HGF. Sudanese “domestic” individuals DSU02 and DSU08 clustered with the wild samples, likely reflecting wild-caught individuals. In addition, Iranian gfIR09 and gfIR10 and Hungarian HUNG12 and HUNG13 grouped with the samples from Hungary and China, respectively. To reduce the effects of population structure and cryptic relatedness (Lawson and Falush 2012), we removed these 10 outliers and re-grouped the remaining 119 samples into 12 populations in subsequent population genomic analyses.

### Population Genomic Diversity

**Fig. 3.**
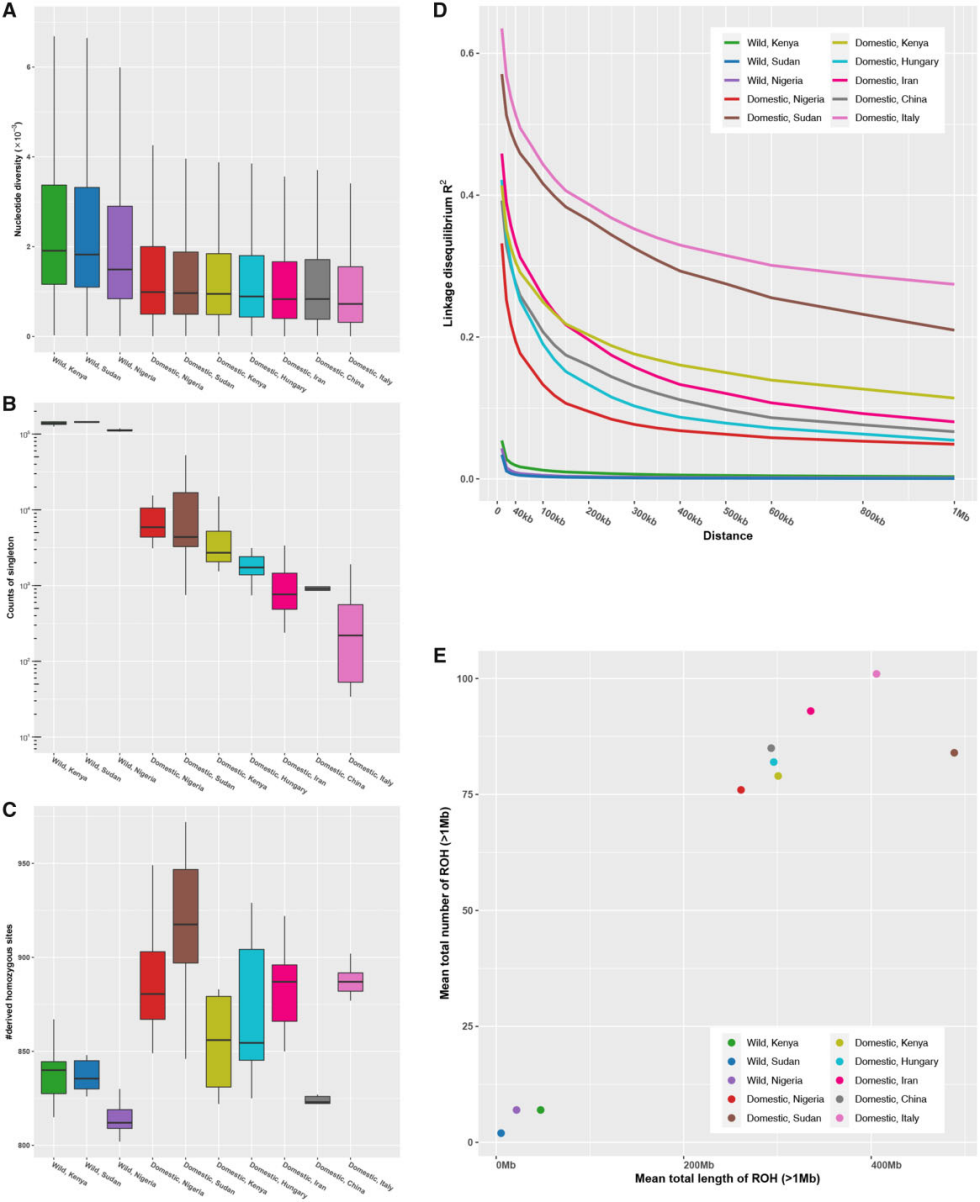
Genetic diversity, genetic load, LD, and runs of homozygosity. (*A*) Nucleotide diversity (Pi). (*B*) The counts of singletons. (*C*) Genetic load in exonic region. (*D*) Decay of LD. (*E*) Runs of homozygosity.

We calculated genetic diversity indexes across ten HGF populations. Wild HGF populations had greater nucleotide diversity (fig. 3*A*) and singleton statistic (fig. 3*B*) than domestic HGF populations. Further, wild HGF samples from Kenya and Sudan had higher diversity than the Nigerian population. Among the domestic HGF samples, the genetic diversity was highest in the Nigerian population and lowest in the Italian population. Domestic HGF, with the exception of the Chinese population, generally had higher mutational load (measured by GERP score > 2) than wild HGF (fig. 3*C*). LD (expressed as *R*^2^) decay rates were higher in wild HGF than in domestic HGF populations (fig. 3*D*). Among the domestic HGF samples, LD decay rates were highest in the Nigerian population and lowest in the Italian population followed by the Sudanese population. Scanning genomes obtained data on runs of homozygosity (ROHs > 1 Mb) and provided insights into inbreeding in HGF populations. Wild HGF populations had a lower mean number and sum length of ROHs than domestic HGF populations (fig. 3*E*). Among the domestic HGF samples, the Nigerian population had the lowest level of ROHs. The Italian and Sudanese populations presented the highest mean number and sum length of ROHs, respectively.

### Detection of Genetic Admixture

We used *f* statistics to explore the divergence and gene flow between wild and domestic HGF. Outgroup *f*_3_ statistics (Raghavan et al. 2014) were obtained for “outgroup-vulturine guinea fowl: wild HGF, target-domestic HGF.” Compared with wild HGF from Kenya and Sudan, wild HGF from Nigeria had more positive *f*_3_ values, suggesting having a closer relationship with domestic HGF (supplementary fig. S4, Supplementary Material online). *D* statistics (Durand et al. 2011) were calculated and showed that the domestic HGF from Sudan had significantly positive *D* values (*Z* > 3), suggesting gene flow between domestic HGF from Sudan and wild HGF from Kenya/Sudan (supplementary fig. S5, Supplementary Material online). Domestic HGF from other regions showed significantly negative *D* values (*Z* < −3), suggesting gene flow between the wild HGF from Nigeria and wild HGF from Kenya/Sudan. Gene flow from the HGF to domestic HGF in Sudan was indicated in the TreeMix (Pickrell and Pritchard 2012) when *m* = 1 (supplementary fig. S6, Supplementary Material online) in 99.9% of the bootstrap replicates. Moreover, we used qpGraph (Patterson et al. 2012) to fit a tree model that satisfied the proposed genetic relationships and gene flows (supplementary fig. S7, Supplementary Material online). Briefly, admixture events from Sudanese wild HGF to Nigerian wild HGF and Sudanese domestic HGF were necessary to explain the observed *f* statistics.

### Inference of Demographic History

**Fig. 4.**
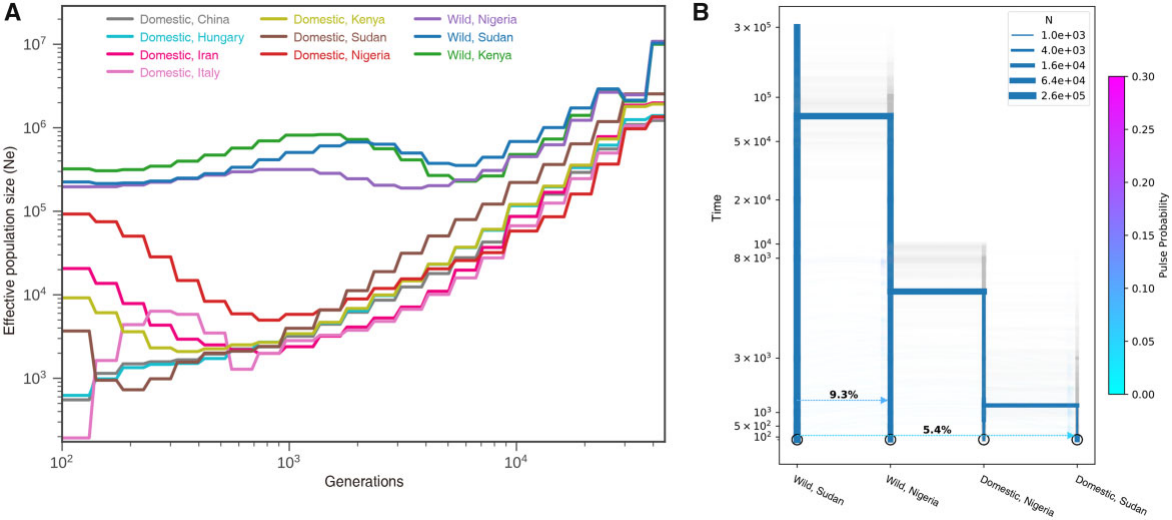
Inference of demographic history. (*A*) The effective population changes through the time estimated by using SMC++. (*B*) The demographic model inferred with momi2.

We applied SMC++ (Terhorst et al. 2017) to infer the changes of effective population size (*N*_e_) of HGF populations. Within the past 8,000 years, inferred *N*_e_ divided into wild and domestic groups (fig. 4*A*). Domestic HGF populations underwent much shaper decreases than wild populations, and the Italian population had the smallest *N*_e_ estimation. This pattern was also observed in the analysis of PopSizeABC (Boitard et al. 2016) (supplementary fig. S8, Supplementary Material online). Incorporating results of phylogeny, genetic admixture, and *N*_e_ estimation, we employed momi2 (Kamm et al. 2020) to test the hypotheses of HGF domestication in East and/or West Africa (Darwin 1883; Blench and MacDonald 2000; Andah et al. 2014) and to estimate the related parameters (details in Materials and Methods). The selected model, which included 100 additional bootstrap iterations (fig. 4*B* and supplementary fig. S9, Supplementary Material online), showed that domestic HGF populations split from wild HGF populations at 5,452 YBP (95% bootstrap interval 9,916–2,548). The divergence between Nigerian and Sudanese domestic HGF was dated to 1,261 YBP (95% bootstrap interval 5,180–162). The intensity of gene flow from wild Sudanese HGF to wild Nigerian HGF (9.3%) was stronger than that from wild to domestic HGF populations in Sudan (5.4%). The time of gene flow from Sudanese wild HGF to Nigerian wild HGF dated to around 1,448 YBP, whereas that from wild to domestic HGF populations in Sudan occurred around 154 YBP.

### Scan of Selective Signals

Because PCA ascertained HGF population structure (fig. 2*C*), we adopted the outlier approach with PCAdapt (Luu et al. 2017) to identify genomic regions that were affected by positive selection based on allele frequency data of 4,695,945 SNPs. Using the threshold of top > 0.1% SNPs (*P* < 0.001), we identified 4,393 SNPs as potentially having selective signals, among which 1,941 were annotated into 453 genes (supplementary table S3 and fig. S10, Supplementary Material online). We checked the derived allele frequency using the six samples from two wild species (one *G. pucherani* and five *A. vulturinum*) as outgroups. We found that 2,887 out of 4,393 SNPs were fixed with derived alleles in all domestic HGF populations except for domestic HGF from Sudan since gene flow from wild HGF was detected in the population (supplementary fig. S6, Supplementary Material online). Most SNPs (3,496 out of 4,393) had high difference of derived allele frequency more than 0.90 between wild and domestic HGF populations. Top-rank loads according to PC1 and PC2 involved 347 and 101 potentially selected genes, respectively. We further applied PBS (target-all combined domestic HGF, control-wild HGF from Nigeria, background-wild HGF from Kenya and Sudan) to detect positive selection using the empirical quantiles of top 0.5% SNPs. A total of 78 genes were detected in both PCAdapt and PBS and they were significantly enriched in GO terms (Fisher’s exact test *P* value < 0.05) related to nervous system (e.g., “extracellular ligand-gated ion channel activity,” GO:0005230; “GABA receptor activity,” GO:0016917) and muscle (“mechanosensitive ion channel activity,” GO:0008381; “cytoskeletal protein binding,” GO:0008092) (table 2). For instance, *GRIA4* encodes an AMPA-sensitive glutamate receptor that functions as a ligand-gated ion channel and mediates synaptic transmission and neuroplasticity (Zhu et al. 2000). The genes of *ACTN1* (Blondelle et al. 2019) and *PIEZO2* (Delle Vedove et al. 2016) play important roles in muscle development and function.

**Table 2:** Selective Genes Associated to the HGF Domestication and Subsequent Breeding

GO ID	Term	Significant Gene	Fisher’s Exact Test *P* Value
GO:0004890	GABA-A receptor activity	*GABRB4*, *GABRG4*	0.0038
GO:0016300	tRNA (uracil) methyltransferase activity	*ALKBH8*	0.0055
GO:0032452	Histone demethylase activity	*KDM6A*	0.0055
GO:0071558	Histone demethylase activity (H3-K27 specific)	*KDM6A*	0.0055
GO:0005230	Extracellular ligand-gated ion channel activity	*GRIA4*, *GABRB4*, *GABRG4*	0.0056
GO:0016917	GABA receptor activity	*GABRB4*, *GABRG4*	0.0058
GO:0008381	Mechanosensitive ion channel activity	*PIEZO2*	0.011
GO:0030507	Spectrin binding	*CAMSAP2*	0.011
GO:0015276	Ligand-gated ion channel activity	*GRIA4*, *GABRB4*, *GABRG4*	0.0148
GO:0022834	Ligand-gated channel activity	*GRIA4*, *GABRB4*, *GABRG4*	0.0148
GO:0004525	Ribonuclease III activity	*DICER1*	0.0164
GO:0004859	Phospholipase inhibitor activity	*RORA*	0.0164
GO:0032296	Double-stranded RNA-specific ribonuclease activity	*DICER1*	0.0164
GO:0032451	Demethylase activity	*KDM6A*	0.0164
GO:0055102	Lipase inhibitor activity	*RORA*	0.0164
GO:0022836	Gated channel activity	*GRIA4*, *GABRB4*, *GABRG4*, *PIEZO2*	0.0203
GO:0015349	Thyroid hormone transmembrane transporter activity	*SLC16A2*	0.0218
GO:0016175	Superoxide-generating NADPH oxidase activity	*NOX3*	0.0218
GO:0050664	Oxidoreductase activity, acting on NAD(P)H, oxygen as acceptor	*NOX3*	0.0272
GO:0008175	tRNA methyltransferase activity	*ALKBH8*	0.0326
GO:0015020	Glucuronosyltransferase activity	*EXT1*	0.0326
GO:0016891	Endoribonuclease activity, producing 5′-phosphomonoesters	*DICER1*	0.0326
GO:0004383	Guanylate cyclase activity	*GUCY1A2*	0.0379
GO:0016893	Endonuclease activity, active with either ribo- or deoxyribonucleic acids and producing 5′-phosphomonoesters	*DICER1*	0.0432
GO:0008092	Cytoskeletal protein binding	*STARD9*, *ACTN1*, *CAMSAP2*, *RORA*	0.0448
GO:0004521	Endoribonuclease activity	*DICER1*	0.0485
GO:0030983	Mismatched DNA binding	*PMS1*	0.0485

**Fig. 5.**
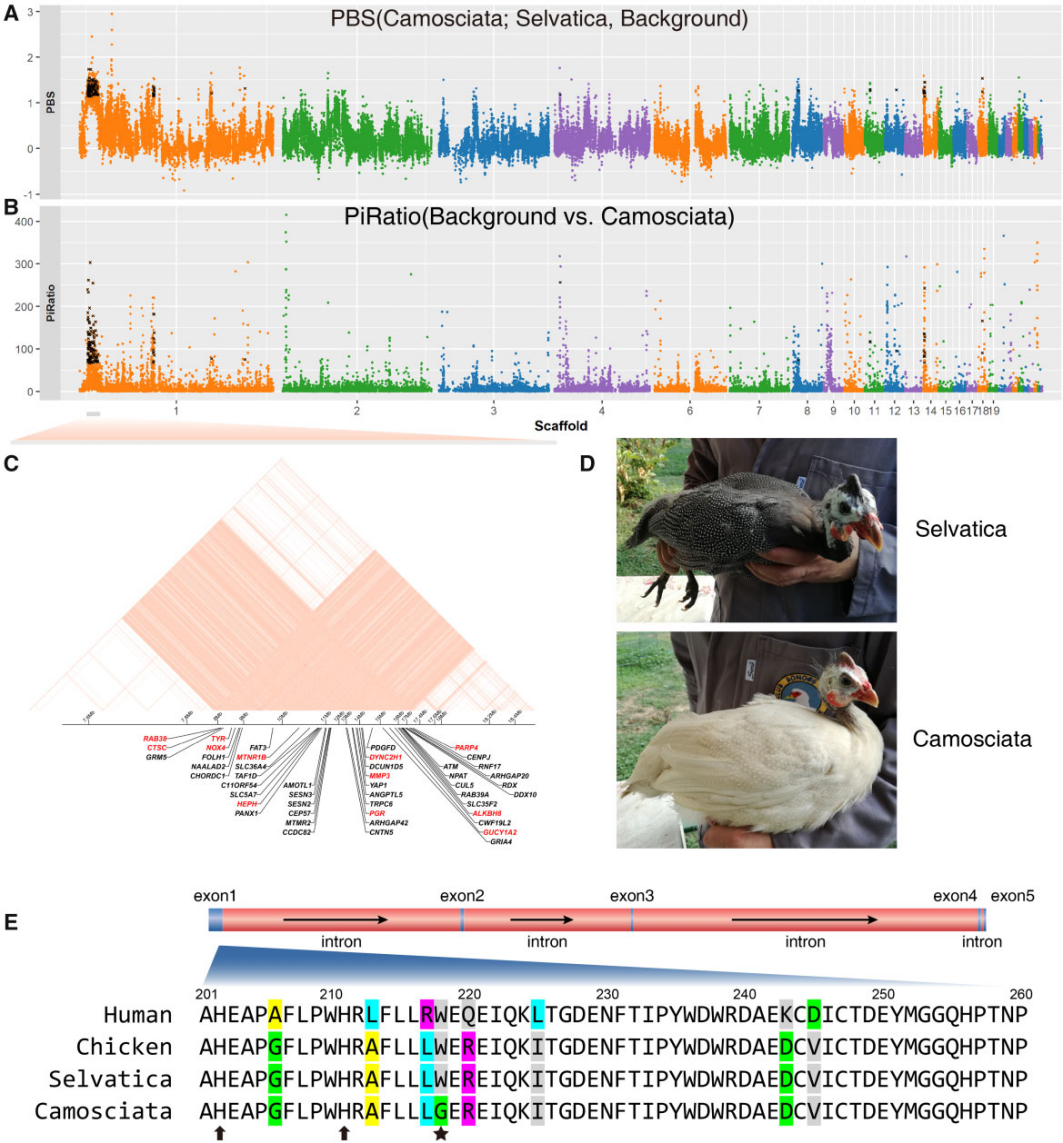
Scan of selective signals in the domestic Italian breed Camosciata. (*A*) The PBS statistic was constructed using the breed Camosciata as target, the breed Selvatica as control and the grouped wild HGF background. (*B*) The Pi-Ratio was calculated from the ratio of nucleotide diversity for the grouped wild HGF background to that for the breed Camosciata. All dots represent the sliding window of 10 kb with 10 kb step size. The windows with values over top 1% quantile for both two statistics were overlapped to identify selective genes which were noted with cross labels. (*C*) The genes located in the LD block of scaffold 1:7,845,520–17,375,296. The selective genes were in red. (*D*) The Camosciata breed with cream white plumage (mutation) and the Selvatica breed with wild plumage type. The Camosciata was selected from a small breeding flock originated in France and brought to Italy in 1922 and differs from the solid white variety due to the pigmented skin of the neck and the visible white spotting of the plumage absent in the solid white birds. The photos were taken at Az. Agricola E. Oggioni in Italy and provided by Maria Giuseppina Strillacci and Erica Gorla. (*E*) The mutation p.Trp218Gly in exon 1 of *TYR* gene. The alignment of protein sequences was shown. This mutation was indicated by star label. The neighboring histidine residues which were essential for TYR function via binding to copper ions were noted with arrows.

Because plumage color is a classical paradigm in poultry domestication and breeding (Domyan et al. 2014; Chen et al. 2015; Zhou et al. 2018), we screened for selective sweeps on the Italian domestic breed Camosciata showing cream white plumage with defined white spots (mutant type; fig. 5) (Ghigi 1936). The breed Selvatica with its gray-black (wild type) plumage, which showed a close relationship with Camosciata (fig. 2*A*), was used for comparison. The PBS (target-Camosciata, control-Selvatica, background-wild HGF from Kenya and Sudan) and Pi-ratio statistic (ratio of background-wild HGF from Kenya and Sudan to target-Camosciata) were used in a sliding window approach (fig. 5*A* and *B*). The top > 1% SNPs were selected as outliers. Overlapping in 141 windows identified 63 potentially selected genes (supplementary table S4, Supplementary Material online). GO categories “melatonin receptor activity (GO:0008502),” “steroid binding (GO:0005496),” “L-malate dehydrogenase activity (GO:0030060),” and “catalytic activity (GO:0003824)” were enriched (Fisher’s exact test *P* value < 0.05) in the GO analyses (table 3). Among the selected genes, tyrosinase gene (*TYR*) accounting for white plumage in chickens (Chen et al. 2015) located in a 9.5 Mb LD block (fig. 5*C*) and the pattern of genotype distribution around this *TYR* gene distinguished breed Camosciata from other HGF populations (supplementary fig. S11, Supplementary Material online). We screened the variants in *TYR* gene across the HGF populations and identified one nonsynonymous mutation p.Trp218Gly fixed in the Camosciata breed with cream white plumage (fig. 5*D*). This locus was conserved across species and located closely to the highly conserved histidine residues which were essential in catalytic activity of tyrosinase via binding to copper ions (fig. 5*E*). The mutation p.Trp218Gly was predicted to be probably damaging with a score of 0.998 (sensitivity: 0.27; specificity: 0.99) in the PolyPhen-2 prediction, suggesting it likely affected tyrosinase function and caused white plumage in the Camosciata breed.

**Table 3:** GO-Enriched Selective Genes in the Domestic Italian Breed Camosciata

GO ID	Term	Significant Genes	Fisher’s Exact Test *P* Value
GO:0008502	Melatonin receptor activity	*MTNR1B*	0.028
GO:0005496	Steroid binding	*PGR*	0.029
GO:0030060	L-malate dehydrogenase activity	*MDH2*	0.041
GO:0003824	Catalytic activity	*RAB38*, *CTSC*, *TYR*, *NOX4*, *HEPH*, *MRE11*, *MMP3*, *MMP13*, *DYNC2H1*, *GUCY1A2*, *ALKBH8*, *ACAT1*, *PARP4*, *LATS2*, *BMX*, *TMEM27*, *CTPS2*, *WWOX*, *MDH2*, *STYXL1*	0.046

## Discussion

By integrating multiple genomic technologies, we provide updated HGF genome assembly HGFv1 to a near-chromosome level. Compared with reference genome NumMel1.0, HGFv1 is more improved in sequence contiguity (table 1). Incorporating PacBio full-length RNA sequencing data also improves the annotation, leading to the characterization of 14,573 protein-coding genes. The HGFv1 assembly is compatible with current poultry reference genomes (Dalloul et al. 2010; Shapiro et al. 2013; Lu et al. 2015; ZhOu et al. 2018; Morris et al. 2020). Thus, HGFv1 provides a robust reference not only for various HGF researches, but also for Galliformes poultry (e.g., chicken, quail, and turkey) studies.

The whole genomes of 129 guinea fowl samples facilitates hypothesis testing on the domestication of HGF. Wild HGF from Nigeria is more closely related to the domestic HGF than the wild HGF from Kenya and Sudan (fig. 2 and supplementary fig. S3, Supplementary Material online). This result reject Darwin’s hypothesis of an East African origin of domestic HGF (Darwin 1883). This pattern is also supported by previous study revealing that the wild HGF populations from Burkina Faso in West Africa rather than those from South Africa showed a closer relationship to domestic HGF populations (Vignal et al. 2019). Among the domestic HGF populations in our study, the domestic HGF from Nigeria has the highest level of genetic diversity and LD decay rate, but the lowest level of ROHs (fig. 3). Further integrating the results of gene flow (supplementary figs. S4 and S6, Supplementary Material online) and demographic changes (fig. 4*A*), we hypothesize a single domestication event in West Africa (fig. 4*B*), which appears to have occurred after the split of wild and domestic HGF around 5,500 YBP but before the divergence of domestic HGF around 1,300 YBP. During this period, a roughly 12-fold population bottleneck occurred. The genetic diversity was decreased but the levels of LD and ROHs were increased (fig. 3). The scenario overlaps with the domestication and diffusion inferred by genomic data of pearl millet (*Cenchrus americanus*) during 5,889–3,685 YBP (Burgarella et al. 2018) and African rice (*Oryza glaberrima*) during 3,200–2,000 YBP (Cubry et al. 2018) in West Africa. The domestication of HGF and some Western African crops likely mirror each other, which are ascribed to cultural responses to the transition from a “green Sahara” to the desert and subsequent climate changes (Kropelin et al. 2008).

The population genomic analyses provide novel insights into the genetic changes as well as their potential effects in ancient domestication and recent breeding of HGF. In general, the accumulation of deleterious mutations was increased (fig. 3*C*). Domestic HGFs are generally less skittish and locomotive than their wild counterparts (Roberts 2002). Among the selected genes with differentiation between wild and domestic HGF (table 2 and supplementary table S3, Supplementary Material online), our analyses identify selective genes such as *GRIA4* involved in nervous system, implying genetic basis for behavioral changes in domestication. *GRIA4* (also known as *GluR4* in mice) belongs to glutamate receptor genes which downregulate excitatory signaling and stress response in domesticated animals (O’Rourke and Boeckx 2020). Interestingly, GRIA4 was also identified as a susceptibility locus for refractive error and myopia (Verhoeven et al. 2013), implying additional roles in visual deterioration involved in domestication (Wang et al. 2016). Meanwhile, our analyses detect several selective genes (e.g., *ACTN1* and *PIEZO2*) playing substantial roles in muscle function. This may explain the reduced locomotion ability (including flying) in domesticated poultry (Wang et al. 2017; Stover et al. 2018). The results suggest selective genes involved in behavioral and locomotive changes facilitating the management of domesticated HGF.

As compared with other domestic HGF populations, Italian population, consisting of two breeds (Camosciata and Selvatica), shows the lowest levels of genetic diversity but the highest levels of LD and ROHs (fig. 3). The *N*_e_ estimation was small (fig. 4*A* and supplementary fig. S8, Supplementary Material online). All the patterns consistent with bottlenecking in extensive breeding practices, as known by the evidences on selection starting in the first half of 1900s (Ghigi 1936). By screening signals of selection, our results identify mutation p. Trp218Gly in *TYR* as the candidate causal locus for white plumage in Camosciata breed. Selected genes *PGR* (progesterone receptor) and *MMP13* (Matrix Metallopeptidase 13), which function in poultry fertility (Shen et al. 2016; Yuan et al. 2016), link with *TYR* in the 9.5 Mb LD block containing other selected genes (fig. 5*C*). The long LD block in the genomes brought challenge to refining the casual loci of white plumage, but provided an opportunity to investigate the pleiotropy and linkage involved in the breeding (Wright et al. 2010). Our results arouse the possibility that the white plumage-orientated selection leads to numerous changes in fecundity and other phenotypes in the breeding of Camosciata.

In summary, we de novo assemble the genome of HGF to obtain a reference-quality avian genome. Together with sequenced population genomes, the data resource has potential to facilitate innovations in genetic resource management and improvement for HGF. Our population genomic analyses provide in-depth insights into the genomic architecture and population history of wild and domestic HGF populations. Our findings in combination with recent genomic analyses of African rice (Cubry et al. 2018; Choi et al. 2019), African yam (Scarcelli et al. 2019), and pearl millet suggest West Africa as a major cradle of both animal and plant domestication.

## Materials and Methods

### Sampling

A total of four adult male domestic (HGF) individuals from China were sampled for de novo genome assembly (supplementary table S1, Supplementary Material online). Independently, a total of 129 samples were collected for WGS (supplementary table S2, Supplementary Material online). The study was approved by the Internal Review Board of Kunming Institute of Zoology, Chinese Academy of Sciences (SYDW20150605001). The sampling of wild species was approved by Kenyan Wildlife Service and Nigeria National Park Service under permit numbers KWS/BRM/5001 and NPH/GEN/530/I/33, respectively. The samples from Sudan were taken from available collections (Weimann et al. 2016). A “no objection for the research” from the Directorate of Veterinary Services, Ministry of Agriculture, Livestock and Fisheries in Kenya under permit number RES/POL/VOL.XXVII/162 was obtained to use domestic Kenyan HGF samples. The Italian HGF populations were available according to the n. OPBA-56-216 document, allowing the use of collected samples for research purpose in available bio-banks. The domestic samples from Hungary, Iran, and Nigeria were collected based on the informed consent of the private HGF owners.

### Genome Assembly and Annotation

We followed the combined strategy (Bickhart et al. 2017) based on PacBio long-read sequencing, BioNano optical mapping, and Hi-C scaffolding to obtain a de novo assembly of HGFv1. The genome synteny between HGFv1 and chicken reference genome GRCg6a (GCA_000002315.5) was checked by using MUMmer4 (Marcais et al. 2018). The assembly quality of HGFv1 was evaluated with BUSCO v3 (Waterhouse et al. 2018), LAI (LTR assembly index) (Ou et al. 2018), and RNA-seq reads mapping (Darris et al. 2015). The HGFv1 assembly was annotated for gene content using the NCBI Eukaryotic Genome Annotation Pipeline. The details were described in the supplementary data, Supplementary Material online.

### Population Genome Resequencing, SNP Calling, and Filtering

Genomic DNA was extracted from muscle or blood samples with the phenol-chloroform method. We followed the manufacturer’s protocols to sequence 129 whole genomes with paired-end 150 strategy on Illumina HiSeq 4000 and NovaSeq 6000 platforms. After removing the adapters and low-quality reads with Btrim v0.3.0 (Kong 2011), the filtered reads were mapped to the reference genome using BWA-MEM (Li 2013) with default parameters. BAM files were sorted and marked PCR duplication by Picard v1.119 using SortSam and MarkDuplicates modules, variants calling was performed using the Genome Analysis Toolkit (McKenna et al. 2010) (GATK, v3.6) with all 129 samples jointly. The details were described in the supplementary data, Supplementary Material online. We finally considered 44,035,924 biallelic SNPs in subsequent analyses.

### Phylogeny and Population Structure

To minimize the nonindependence of variations, we pruned the data set using PLINK v1.9 (Chang et al. 2015) with options “–indep-pairwise 50 10 0.2” (Anderson et al. 2010). The maximum-likelihood (ML) tree was constructed using FastTree v2 (Price et al. 2010) and RAxML (Stamatakis 2014). FastTree used 1,000 resamples to calculate local support values. RAxML used “-b 500” to conduct 500 bootstrap iterations. Principal component analysis (PCA) was performed with smartpca in EIGENSOFT v7.2.0 (Patterson et al. 2006). The individual ancestry coefficients were calculated by ADMIXTURE v1.3 (Alexander et al. 2009), when the value of *K* between 2 and 10. For each *K* value, ten repeated runs were conducted with random, varied seeds.

### Genetic Diversity, LD, and ROHs

We regrouped populations according to PCA and ADMIXTURE results. The nucleotide diversity (Nei and Li 1979) within each population was calculated using R package PopGenome (Pfeifer et al. 2014). We used R package SeqVarTools (Gogarten et al. 2019) to count singletons per individual. We applied a recently developed unbiased estimator for linkage disequilibrium (LD) (Ragsdale and Gravel 2020) that was not sensitive to small population size. We used the R package detectRUNS (Biscarini et al. 2018) to detect ROHs using the pruned data set to eliminate the impact of strong LD. The result was summarized with two measurements defined as the mean of total length of ROHs more than 1 Mb and the number of ROHs.

### Genetic Load

We used the genomic evolutionary rate profiling (GERP) scores computed for the 58 sauropsids multiple whole-genome alignment (ftp://ftp.ensembl.org/pub/release-100/compara/, last accessed May 21, 2020) as a measure of evolutionary constraint acting on the SNPs. Positive GERP scores, larger than 2, represented a substitution deficit, which were expected for sites under selective constraint. We used MUMmer4 to align the HGFv1 assembly to that of NumMel1.0 and extracted one-to-one alignment under the minimum identity of 90% and minimum length of 1,000 using the options “-i 90 -l 1000 -1 -q”. Sequencing data of six samples from two wild species (one *G. pucherani* and five *A. vulturinum*) were used as outgroups to predict the ancestral and derived allelic state of all polymorphic sites. Variant was categorized as ancestral if the six outgroup samples had the same genotype (homozygous reference or homozygous alternative). The 15,768,975 identified variants were extracted for each sample and classified as homozygous ancestral, heterozygous, or homozygous derived. We kept the SNPs under three criteria: 1) succeed in MUMmer4 alignment, 2) had record of GERP score, 3) located in exonic regions. A total of 324,210 polymorphic SNPs within HGF populations were used to count the number of homozygous derived sites per individual for each of HGF populations.

### Detection of Gene Flow

Using the genome wide allele frequency data for each HGF population, we used qp3Pop and qpDstat as implemented in AdmixTools v5.1 (Patterson et al. 2012) to calculate outgroup *f*_3_ statistic (Patterson et al. 2012) and *D* statistic (Durand et al. 2011). We adopted the TreeMix software v1.13 (Pickrell and Pritchard 2012) to build a ML tree setting the vulturine guinea fowl as outgroup. We used the options “-k 1000 -global” to make blocks of 1,000 SNPs. We ran 1,000 replicates for each tree, adding the option “-bootstrap.” When there’s migration event, we add “-se” option to calculate the standard errors of migration weights. We used qpGraph with the parameter “allsnps: NO” in AdmixTools to build an admixture graph. The vulturine guinea fowl was set to be outgroup.

### Temporal Fluctuation of *N*_e_

We randomly selected eight different samples for each of HGF populations to avoid bias in sample size. Because the mutation rate for HGF was unavailable, we used the rate 1.91 × 10^−9^ per site per year (Jarvis et al. 2014) for the chicken lineage.

We adopted SMC++ (Terhorst et al. 2017) to infer Ne changes of HGF.

We also employed PopSizeABC (Boitard et al. 2016) that was accurate even for recent history with the recombination rate of 1.7 cM/Mbp (Pigozzi 2016). The details were described in the supplementary data, Supplementary Material online.

### Inference of Demographic Model

We used momi2 (Kamm et al. 2020) to explore demographic model based on four populations: wild HGF from Sudan, wild HGF from Nigeria, domestic HGF from Nigeria, and domestic HGF from Sudan. We split the extracted folded site frequency spectrum into 100 equally sized blocks for jackknifing and bootstrapping. We introduced two gene flow events originated from wild HGF from Sudan. We set constant population size for wild HGF populations while allowing changes for domestic HGF populations after their divergence from wild ancestor. We fitted 20 independent runs with different starting parameters and kept the model with the biggest log-likelihood value of the three runs. We referred to the Akaike information criterion (AIC) to select model with smallest AIC. We conducted 100 bootstrap calculations for the estimation of parameter range.

### Scan of Selective Sweep

We used the R package PCAdapt (Luu et al. 2017) to detect selective signals under the context of PCA for wild and domestic HGF based on the allele frequency data. The SNPs with minor allele frequency ≤ 0.05 were filtered. We randomly sampled 100,000 SNPs to get a background distribution of statistics, we used the threshold of top 0.1% corresponded to a *P* value cut-off of 0.001. We considered loci over this threshold as outliers under potential selection. We classified the outlier loci according to the association with PC1 and PC2, respectively. The potential selective genes (genes under selection) were characterized according to the genome assembly annotation.

We also used population branch statistic (PBS) (Yi et al. 2010) in the form (target-all combined HGF; control-wild HGF from Nigeria; background-wild HGF from Sudan and Kenya) to detect selective sweep. We used the R package topGO (Alexa and Rahnenfuhrer 2019) with the algorithm set to be “parentchild” for Gene Ontology enrichment analysis.

In contrast to the SNP-based PBS calculation, we adopted PBS and Pi-Ratio statistics (Nei and Li 1979) in sliding window to detect the selective sweeps in Camosciata breed. The SNPs with low frequency (<0.10) were filtered. We used PopGenome (Pfeifer et al. 2014) to calculate the fixation index (*F*_ST_) and nucleotide diversity within population (Pi). We set the window size of 10 kb and the sliding step of 10 kb. Using the data of randomly sampled 10,000 windows, we set the threshold from sample distribution with a *P* value cut-off of 0.01. The windows with values over top 1% quantile for both two statistics were overlapped to identify selective genes relative to such windows. For the block that contained dense significant genes located in scaffold 1:7,845,520–17,375,296, we checked the pairwise LD using the unbiased LD estimator (Ragsdale and Gravel 2020). The potential effect of nonsynonymous mutation of *TYR* gene was evaluated by PolyPhen-2 v2.2.2- release 398 (Adzhubei et al. 2010).

### Statistical Thresholds for Outlier Approaches

To identify a threshold for identifying extreme outliers, we used an approach by randomly sampling from the data to get a background distribution. Specifically, for the SNP-based methods of PCAdapt and PBS, we randomly sampled 100,000 SNPs and used the score within top 0.1% as the threshold corresponding to *P* value cut-off of 0.001. For the SNP-based PBS results, we used a threshold with the top 0.5%. For the window-based PBS and Pi-Ratio analyses, we randomly sampled 10,000 windows of 10 kb to estimate the distribution of PBS and Pi-Ratio scores. We then set top 1% as the threshold corresponding to *P* value cut-off of 0.01 for PBS and Pi-Ratio.

## Data Availability

Raw sequencing data that support the findings of this study have been deposited to the NCBI BioProject database under accession PRJNA639701 and PRJNA639777.

## Supplementary Material

Supplementary data are available at *Genome Biology and Evolution* online.

## Supplementary Material

evab090_Supplementary_Data
