# Erratum to: Genomic analyses unveil helmeted guinea fowl (*Numida meleagris*) domestication in West Africa

**DOI:** 10.1093/gbe/evab174

**Published:** 2021-08-21

**Authors:** Quan-Kuan Shen, Min-Sheng Peng, Adeniyi C Adeola, Ling Kui, Shengchang Duan, Yong-Wang Miao, Nada M Eltayeb, Jacqueline K Lichoti, Newton O Otecko, Maria Giuseppina Strillacci, Erica Gorla, Alessandro Bagnato, Olaogun S Charles, Oscar J Sanke, Philip M Dawuda, Agboola O Okeyoyin, John Musina, Peter Njoroge, Bernard Agwanda, Szilvia Kusza, Hojjat Asadollahpour Nanaei, Rana Pedar, Ming-Min Xu, Yuan Du, Lotanna M Nneji, Robert W Murphy, Ming-Shan Wang, Ali Esmailizadeh, Yang Dong, Sheila C Ommeh, Ya-Ping Zhang

*Genome Biology and Evolution*, Volume 13, Issue 6, June 2021, evab090, https://doi.org/10.1093/gbe/evab090

In the originally published version of this manuscript, there was an error in the title. The title read as: “Genomic Analyses of Unveil Helmeted Guinea Fowl (*Numida meleagris*) Domestication in West Africa”. This has been corrected to: “Genomic Analyses Unveil Helmeted Guinea Fowl (*Numida meleagris*) Domestication in West Africa”. This error was introduced during production and has now been corrected.

