# Supplementary material for: Genomic Analyses Unveil Helmeted Guinea Fowl (Numida meleagris) Domestication in West Africa
Source: Genome Biol Evol. 2021 May 1;13(6):evab090. doi: 10.1093/gbe/evab090 (PMC8214406; doi:10.1093/gbe/evab090)
Supplement: evab090_Supplementary_Data [file evab090_supplementary_data.pdf]

Supplementary Text

Sampling

The YPT651 individual was used in genome sequencing on PacBio Sequel and Illumina HiSeq platforms. Samples of ten tissues from YPT651 were collected for Illumina RNA sequencing. Because the DNA and RNA conservations failed to pass the quality evaluations, we performed additional samplings. The YP160303-001 individual was applied in BioNano Irys mapping and Hi-C sequencing. The sample of testis from Bz-2 and Dz-1 were used in PacBio Sequel RNA sequencing.

Genome assembly and annotation

Genomic DNA was extracted from the liver tissue of YPT651 with a QIAamp DNA Mini Kit (QIAGEN). Illumina sequencing library of short fragments was constructed with insert size of 551bp and a total of 34.34Gb 100bp paired-end reads were used to correct long-reads assembly. The PacBio Sequel subreads were corrected using NextDenovo (https://github.com/Nextomics/NextDenovo) with default parameters. The 30X longest corrected subreads were extracted and then assembled with MECAT2 (Version: v20190314) (Xiao, et al. 2017). The parameter of detecting overlaps in the assemble stage was set as “ASM_OVLP_OPTIONS=-n 100 -z 10 -b 2000 -e 0.5 -j 1 -u 0 -a 400”. The contigs were polished twice with the NGS sequencing data using Pilon (Version 1.23) (Walker, et al. 2014).

High molecular weight genomic DNA was extracted from the blood with the BioNano Prep™ Blood and Cell Culture DNA Isolation Kit. We assessed label density and selected *Nt.BspQ1* to label DNA. The whole-genome map at single-molecule resolution was assembled with IrysSolve (BioNano Genomics). We adopt the runBNG pipelines (Yuan, et al. 2017) in a hybrid assembly mode with “-B 1 -N 2” options followed by solving conflict manually. In total, we got 122 scaffolds with N50 length of 23.514 Mb and the max length of 68.872 Mb, the total length is 1049.692 Mb.

The Hi-C library was prepared and sequenced in Nextomics Biosciences Co., Ltd. Biotinylated DNA fragments were enriched and sheared to a fragment size of 300–500 bp again for preparing the sequencing library, which was sequenced on Illumina HiSeq Nova platform.

Following single-molecule real-time sequencing (SMRT) on the PacBio Sequel platform, we obtained ~10.7 million reads (N50 read length 14.2 kb) with a total length of

~128 Gb after removing the adaptors. The 30x longest corrected subreads were processed through MECAT2, yielding a total of 1,003 contigs. We generated 200.8 Gb of cleaned BioNano Irys map data and *de novo* assembled them into BioNano genome maps. The maps served to extend PacBio assembled contigs, resulting in increasing contig N50 to 68.3 Mb. We also generated 106 Gb of cleaned Hi-C reads, of which roughly 241.7 million reads (68.4%) mapped uniquely to combined PacBio and BioNano assembly, resulting in a final set of approximately 215.8 million valid read-pairs that were used to generate contact information. Clean Hi-C reads were mapped to the draft assembly with juicer (Durand, et al. 2016), and then a candidate chromosome-length assembly was generated automatically referring to the 3d-dna pipeline (Dudchenko, et al. 2017). Manual review and refinement of the candidate assembly was performed in Juicebox Assembly Tools (Version 1.9.1) (Durand, et al. 2016) for quality control and interactive correction. Then the genome was re-assembled using 3d-dna (Dudchenko, et al. 2017) manually. The scaffolds were anchored near-chromosome level to generate the HGFv1 assembly.

We used MUMmer4 (Marcais, et al. 2018) to align the HGFv1 assembly to the chicken reference genome GRCg6a (GCA_000002315.5) to explore the genome synteny between HGF and chicken. We set "nucmer --mum -l 100" to produce the raw alignments. To reduce noise, we set "delta-filter -i 90 -l 10000" to filter the alignments with identity less than 90% and length less than 10 kb. We used "show-coords" to display the final coordinates.

We evaluated the completeness of the HGFv1 assembly using BUSCO v3 (Waterhouse, et al. 2018). The genome assembly quality was assessed by using the LAI (LTR Assembly Index) (Ou, et al. 2018). The corrected short reads with insert size < 1kb were mapped onto the assembly using BWA-MEM (Li 2013). Picard v1.119 was used to sort and remove duplicate reads. SNP calling was performed using SAMtools (Li, et al. 2009). The previously published Illumina RNA sequencing short reads (Darris, et al. 2015) were processed through Trimmomatic 0.32 (Bolger, et al. 2014) to remove reads containing adapter, reads containing poly-N, and low-quality reads. To compare the mapping rates for the two versions of reference, we aligned the RNA-sequencing reads after quality control to the HGFv1 assembly and the reference NumMel1.0 (GCA_002078875.2) (Vignal, et al. 2019), respectively, by using HISAT v2.1.0 with default parameters (Kim, et al. 2015) to check the mapping rates for the two genome assemblies.

We extracted RNA of the ten tissue samples from YPT651 using TRIzol Reagent (Invitrogen) and then purified the RNA using RNAeasy Mini Kit (QIAGEN). *DNase I* (Promega) was used to digest contaminant genomic DNA. According to Illumina's standard pipeline, RNA-sequencing libraries were prepared from the total RNA of each of the ten

tissue samples, and then sequenced on Illumina HiSeq 2500 platform. The sequencing length for each read was 125 bp. The RNA sequencing reads were cleaned with Trimmomatic and aligned to the genome with Tophat2 (Kim, et al. 2013). Alignments were then assembled independently with Cufflinks (Trapnell, et al. 2010) and *de novo* assembled with Trinity (Grabherr, et al. 2011).

To improve the transcriptome-based annotation, we also extracted RNA from testis tissues of Bz-2 and Dz-1 for PacBio SMRTbell library construction. The Iso-Seq library was prepared according to the isoform sequencing protocol using the SMARTer® PCR cDNA Synthesis Kit and SMRTbell template prep kit 1.0 as described by PacBio. Raw PacBio data were processed using SMRT Link v5.0.1 pipelines. Briefly, the minimum predicted consensus accuracy and minimum full passes were set as 0.80 and 1 separately to process subreads into circular consensus sequences. Sequences shorter than 250 bp in length were discarded. Full-length sequences were subjected to isoform-level clustering by IEC algorithm and polishing by Quiver (Chin, et al. 2013). The high-quality isoforms were used for assembly annotation.

The HGFv1 assembly was annotated for gene content using the NCBI Eukaryotic Genome Annotation Pipeline (Pruitt, et al. 2014). For repeat annotation, we searched for tandem repeats with Tandem Repeats Finder (Benson 1999). The transposable elements were identified a combination of homology-based and *de novo* prediction approaches. For the homolog-based prediction, we identified known repeats were identified using RepeatMasker and RepeatProteinMask (Wheeler, et al. 2013) against Repbase (Release 16.10) (Bao, et al. 2015). RepeatModeler and LTR_FINDER (Xu and Wang 2007) were used in the *de novo* prediction. The tRNA library for chicken (galGal2 Feb 2004) was referred when running LTR_FINDER. For protein-coding gene prediction, we employed EVidence Modeler (Haas, et al. 2008) to consolidate RNA sequencing data and protein alignments with *ab initio* gene predictions and homologous method annotation into the final gene set. Both Illumina and PacBio RNA sequencing assemblies were combined and further refined using PASA (Haas, et al. 2003). We performed protein alignments using exonerate and tblastn with avian proteomes of *Anas platyrhynchos*, *Gallus gallus*, *Meleagris gallopavo*, and *Taeniopygia guttata*. And *ab initio* predictions were conducted by using Augustus (Stanke, et al. 2006), GlimmerHMM (Majoros, et al. 2004), and GENESCAN (Lynn, et al. 2001). The intuitive weighting RNA-seq > protein alignments > *ab initio* predictions were fed into EVM. The EVM models were updated with PASA. Protein-coding gene function were assigned according to the best match alignment using Blastp against Swiss-Prot, TrEMBL (Odonovan, et al. 2002), and KEGG (Tanabe and Kanehisa 2005). The InterProScan functional analysis and Gene Ontology IDs were

obtained with InterProScan (Zdobnov and Apweiler 2001). The pathway to which the gene might belong was derived from the matching genes in KEGG. The Gene Ontology enrichment was done with Ontologizer 2.0 (Bauer, et al. 2008) with a p-value cut-off of 0.05. All predicted protein-coding genes of the HGFv1 assembly were used as references. For the non-coding gene annotation, we annotated tRNA with tRNAscan-SE with default parameters (Lowe and Eddy 1997). We used the homologous method to identify rRNA in term of using the rRNA reference sequence data downloaded from Rfam (Burge, et al. 2013). INFERNAL (Nawrocki, et al. 2009) was used to identify snRNA and miRNA.

SNP calling

Variants calling was performed using the Genome Analysis Toolkit (McKenna, et al. 2010) (GATK, v3.6) with all 129 samples jointly. As SNP and InDel datasets was unavailable for Base Quality Score Recalibrator (BQSR) and InDel Realigner, we used the following approach recommend for non-human data (https://gatkforums.broadinstitute.org/gatk/discussion/1706/best-recommendation-for-base-recalibration-on-non-human-data). We performed an initial round variant calling for our original data by HaplotypeCaller without truth/training variants. We employed GATK tool – harder filter to filter the variants as recommend (https://software.broadinstitute.org/gatk/documentation/article.php?id=3225) with default parameter set and then to provide an initial confidence in the SNPs and InDels sets. Then, we filtered the original BAM files by BQSR and InDel Realigner based on the initial confidence. We re-ran HaplotypeCaller and hard filter to get the raw confidence variants sets which were further filtered as described before (Qanbari, et al. 2019). For SNPs, the GATK option was set as “QD < 4.0, QUAL < 30.0, FS > 60.0, MQ < 40.0, MQRankSum < -10.0, ReadPosRankSum < -7.0, ReadPosRankSum > 7.0, BaseQRankSum < -6.0, BaseQRankSum > 6.0, SOR > 3.0”. Cluster Size and ClusterWindowSize were set to 4 and 10, respectively. For the total variants including SNPs and InDels, we used VCFtools (Danecek, et al. 2011) with argument “--mac 1 --minDP 1 --max-missing 1” to obtain a final no-missing data set including 44,035,924 biallelic SNPs and 4,214,076 InDels for 129 individuals.

Extraction of neutral regions

We extracted the neutral regions from genomes to infer demographic history. According to the annotation, we excluded autosomal SNPs in coding regions, repeated regions, as well as in flank regions of genes within a distance of 10kb. We only considered SNPs in the mappability mask using SNPable (http://lh3lh3.users.sourceforge.net/snpable.shtml).

PopSizeABC

The extracted neutral regions were used to calculate observed summary statistics. We selected 26 time windows and the oldest time was set to be 120,000 YBP using the same procedure reported before (Li and Durbin 2011; Schiffels and Durbin 2014). For data simulation, we set the bounds for the population size in each time window (in log10 scale) to be uniform distribution of [1, 5] and the ratio of population sizes between two consecutive time windows could not exceed 10. We simulated 350,000 samples for each HGF population with corresponding number of haploids using msprime (Kelleher, et al. 2016), and the parameters sampled from the above description. For the comparison of the simulated and observed summary statistics, we adopted R package abc (Csilléry, et al. 2012) with the accept tolerance rate to be 0.005. The neural network regression approach was used with the number of neural networks as 500 and units in the hidden layer as 10. The point estimate was obtained by taking the median of the posterior distribution for the parameter estimation.

Bao W, Kojima KK, Kohany O 2015. Repbase Update, a database of repetitive elements in eukaryotic genomes. Mobile DNA 6: 11. doi: 10.1186/s13100-015-0041-9

Bauer S, Grossmann S, Vingron M, Robinson PN 2008. Ontologizer 2.0--a multifunctional tool for GO term enrichment analysis and data exploration. Bioinformatics 24: 1650-1651. doi: 10.1093/bioinformatics/btn250

Benson G 1999. Tandem repeats finder: a program to analyze DNA sequences. Nucleic Acids Research 27: 573-580. doi: 10.1093/nar/27.2.573

Bolger AM, Lohse M, Usadel B 2014. Trimmomatic: a flexible trimmer for Illumina sequence data. Bioinformatics 30: 2114-2120. doi: 10.1093/bioinformatics/btu170

Burge SW, et al. 2013. Rfam 11.0: 10 years of RNA families. Nucleic Acids Research 41: D226-D232. doi: 10.1093/nar/gks1005

Chin CS, et al. 2013. Nonhybrid, finished microbial genome assemblies from long-read SMRT sequencing data. Nature Methods 10: 563-569. doi: 10.1038/nmeth.2474

Csilléry K, François O, Blum MGB 2012. abc: an R package for approximate Bayesian computation (ABC). Methods in Ecology and Evolution 3: 475-479. doi: 10.1111/j.2041-210X.2011.00179.x

Danecek P, et al. 2011. The variant call format and VCFtools. Bioinformatics 27: 2156-2158. doi: 10.1093/bioinformatics/btr330

Darris CE, et al. 2015. Molecular tools to support metabolic and immune function research in the Guinea Fowl (Numida meleagris). BMC Genomics 16: 358. doi: 10.1186/s12864-015-1520-6

Dudchenko O, et al. 2017. De novo assembly of the Aedes aegypti genome using Hi-C yields chromosome-length scaffolds. Science 356: 92-95. doi: 10.1126/science.aal3327

Durand NC, et al. 2016. Juicer Provides a One-Click System for Analyzing Loop-Resolution Hi-C Experiments. Cell Systems 3: 95-98. doi: 10.1016/j.cels.2016.07.002

Grabherr MG, et al. 2011. Full-length transcriptome assembly from RNA-Seq data without a reference genome. Nature Biotechnology 29: 644-652. doi: 10.1038/nbt.1883

Haas BJ, et al. 2003. Improving the Arabidopsis genome annotation using maximal transcript alignment assemblies. Nucleic Acids Research 31: 5654-5666. doi: 10.1093/nar/gkg770

Haas BJ, et al. 2008. Automated eukaryotic gene structure annotation using EVidenceModeler and the program to assemble spliced alignments. Genome Biology 9: 1-22. doi: 10.1186/gb-2008-9-1-r7

Kelleher J, Etheridge AM, McVean G 2016. Efficient Coalescent Simulation and Genealogical Analysis for Large Sample Sizes. PLoS Computational Biology 12: e1004842. doi: 10.1371/journal.pcbi.1004842

Kim D, Langmead B, Salzberg SL 2015. HISAT: a fast spliced aligner with low memory requirements. Nature Methods 12: 357-360. doi: 10.1038/nmeth.3317

Kim D, et al. 2013. TopHat2: accurate alignment of transcriptomes in the presence of insertions, deletions and gene fusions. Genome Biology 14: R36. doi: 10.1186/gb-2013-14-4-r36

Li H 2013. Aligning sequence reads, clone sequences and assembly contigs with BWA-MEM. arXiv:1303.3997v2 [q-bio.GN] 00: 1-3.

Li H, Durbin R 2011. Inference of human population history from individual whole-genome sequences. Nature 475: 493-496. doi: 10.1038/nature10231

Li H, et al. 2009. The Sequence Alignment/Map format and SAMtools. Bioinformatics 25: 2078-2079. doi: 10.1093/bioinformatics/btp352

Lowe TM, Eddy SR 1997. tRNAscan-SE: a program for improved detection of transfer RNA genes in genomic sequence. Nucleic Acids Research 25: 955-964. doi: 10.1093/nar/25.5.955
Lynn AM, et al. 2001. An automated annotation tool for genomic DNA sequences using GeneScan and BLAST. Journal of Genetics 80: 9-16. doi: 10.1007/BF02811413
Majoros WH, Pertea M, Salzberg SL 2004. TigrScan and GlimmerHMM: two open source ab initio eukaryotic gene-finders. Bioinformatics 20: 2878-2879. doi: 10.1093/bioinformatics/bth315
Marcais G, et al. 2018. MUMmer4: A fast and versatile genome alignment system. PLoS Computational Biology 14: e1005944. doi: 10.1371/journal.pcbi.1005944
McKenna A, et al. 2010. The Genome Analysis Toolkit: a MapReduce framework for analyzing next-generation DNA sequencing data. Genome Research 20: 1297-1303. doi: 10.1101/gr.107524.110
Nawrocki EP, Kolbe DL, Eddy SR 2009. Infernal 1.0: inference of RNA alignments. Bioinformatics 25: 1335-1337. doi: 10.1093/bioinformatics/btp157
Odonovan C, et al. 2002. High-quality protein knowledge resource: SWISS-PROT and TrEMBL. Briefings in Bioinformatics 3: 275-284.
Ou S, Chen J, Jiang N 2018. Assessing genome assembly quality using the LTR Assembly Index (LAI). Nucleic Acids Research 46: e126. doi: 10.1093/nar/gky730
Pruitt KD, et al. 2014. RefSeq: an update on mammalian reference sequences. Nucleic Acids Research 42: D756-D763. doi: 10.1093/nar/gkt1114
Qanbari S, et al. 2019. Genetics of adaptation in modern chicken. PLoS Genetics 15: e1007989. doi: 10.1371/journal.pgen.1007989
Schiffels S, Durbin R 2014. Inferring human population size and separation history from multiple genome sequences. Nature Genetics 46: 919-925. doi: 10.1038/ng.3015
Stanke M, et al. 2006. AUGUSTUS: ab initio prediction of alternative transcripts. Nucleic Acids Research 34: W435-W439. doi: 10.1093/nar/gkl200
Tanabe M, Kanehisa M 2005. Using the KEGG Database Resource. Current Protocols in Human Genetics 38: 1-12.
Trapnell C, et al. 2010. Transcript assembly and quantification by RNA-Seq reveals unannotated transcripts and isoform switching during cell differentiation. Nature Biotechnology 28: 511-515. doi: 10.1038/nbt.1621
Vignal A, et al. 2019. A guinea fowl genome assembly provides new evidence on evolution following domestication and selection in galliformes. Molecular Ecology Resources 19: 997-1014. doi: 10.1111/1755-0998.13017
Walker BJ, et al. 2014. Pilon: an integrated tool for comprehensive microbial variant detection and genome assembly improvement. PLoS ONE 9: e112963. doi: 10.1371/journal.pone.0112963
Waterhouse RM, et al. 2018. BUSCO Applications from Quality Assessments to Gene Prediction and Phylogenomics. Molecular Biology and Evolution 35: 543-548. doi: 10.1093/molbev/msx319
Wheeler TJ, et al. 2013. Dfam: a database of repetitive DNA based on profile hidden Markov models. Nucleic Acids Research 41: D70-D82. doi: 10.1093/nar/gks1265
Xiao CL, et al. 2017. MECAT: fast mapping, error correction, and de novo assembly for single-molecule sequencing reads. Nature Methods 14: 1072-1074. doi: 10.1038/nmeth.4432
Xu Z, Wang H 2007. LTR_FINDER: an efficient tool for the prediction of full-length LTR retrotransposons. Nucleic Acids Research 35: W265-W268. doi: 10.1093/nar/gkm286
Yuan Y, Bayer PE, Lee HT, Edwards D 2017. runBNG: a software package for BioNano genomic analysis on the command line. Bioinformatics 33: 3107-3109. doi: 10.1093/bioinformatics/btx366
Zdobnov EM, Apweiler R 2001. InterProScan - an integration platform for the signature-recognition methods in InterPro. Bioinformatics 17: 847-848. doi: DOI 10.1093/bioinformatics/17.9.847

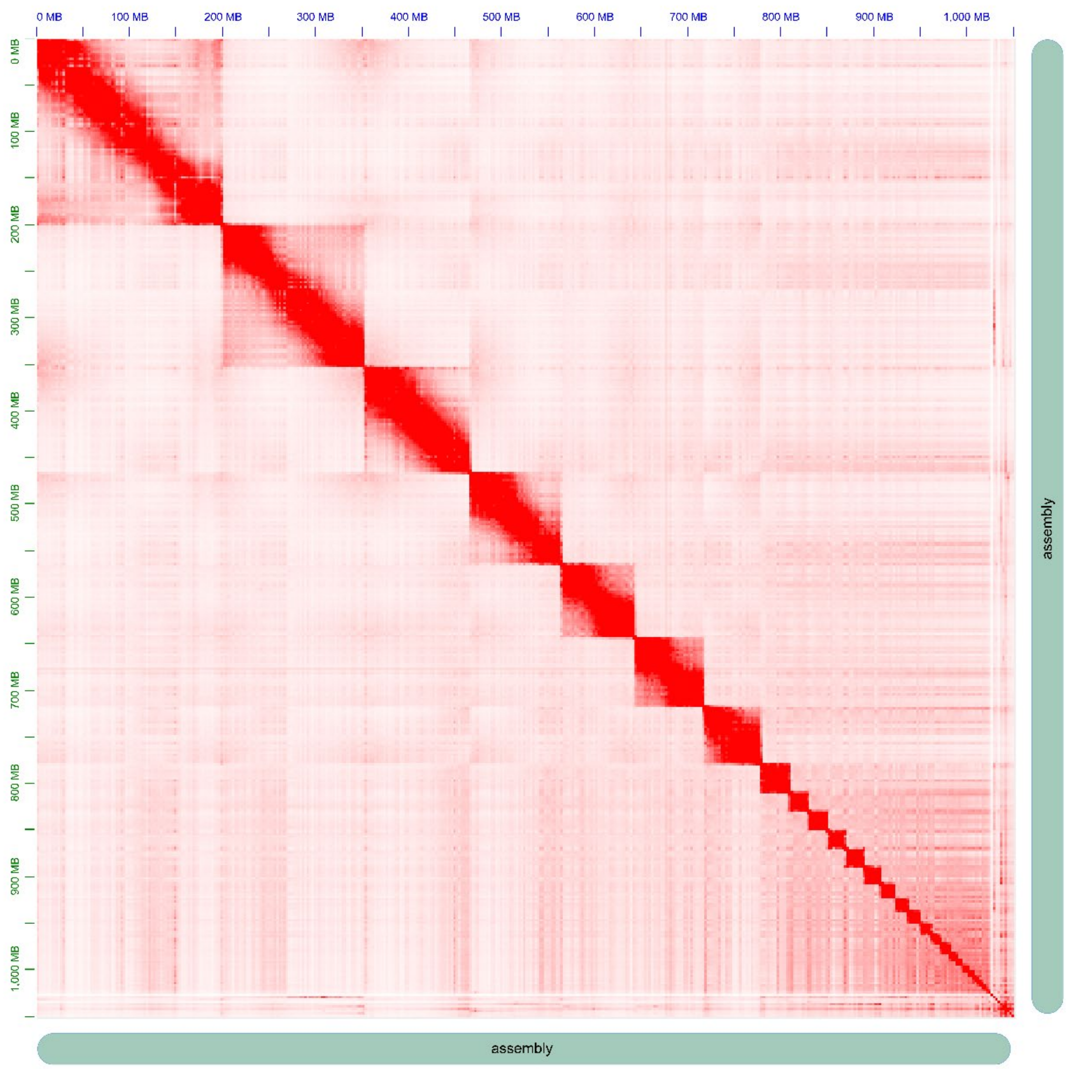

Figure S1. Hi-C interactions among all scaffolds for HGFv1 assembly.

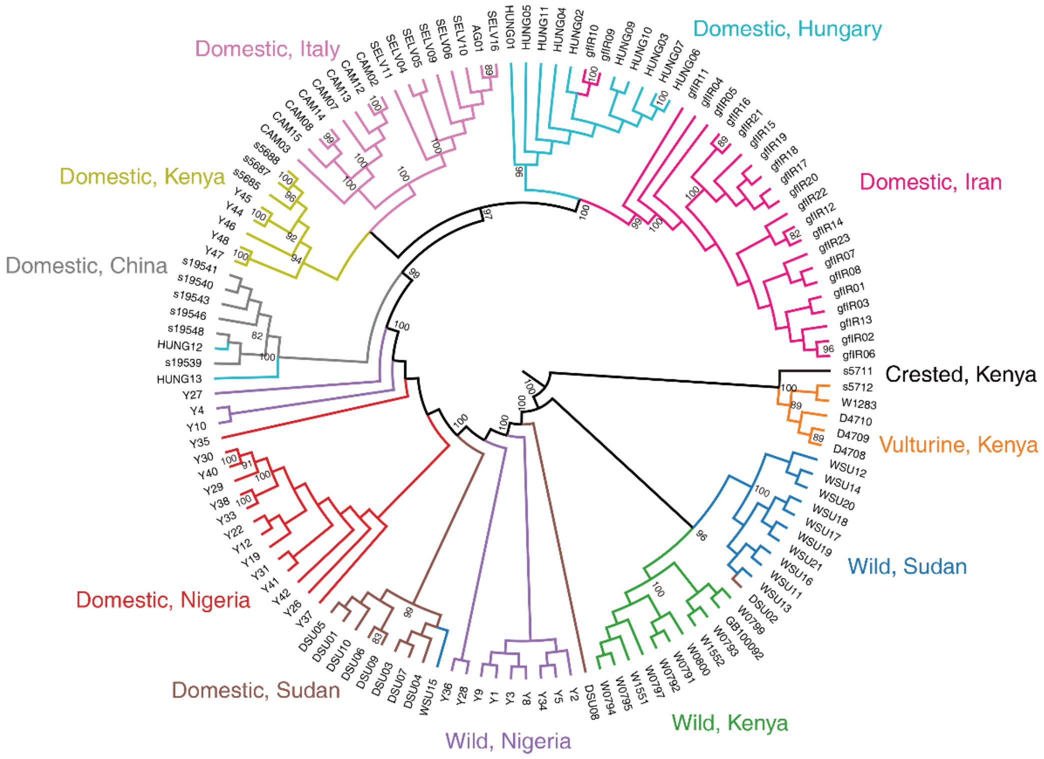

Figure S2. The phylogenetic tree constructed with RAxML. The nodes with support values more than 80% in 500 bootstraps were shown.

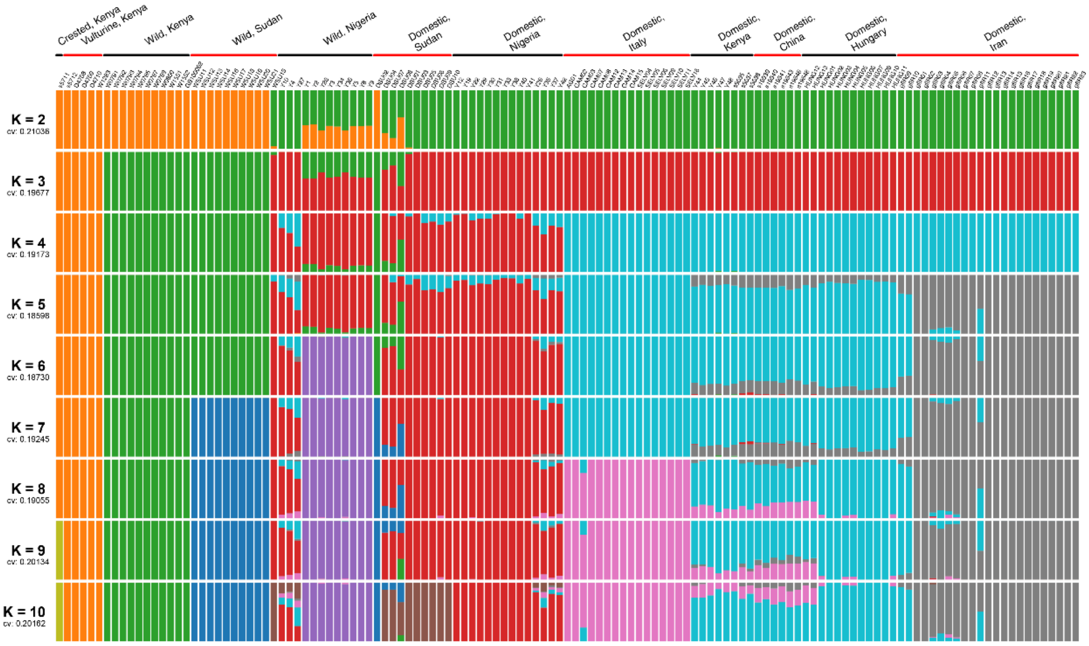

Figure S3. ADMIXTURE analysis with K=2 to K=10.

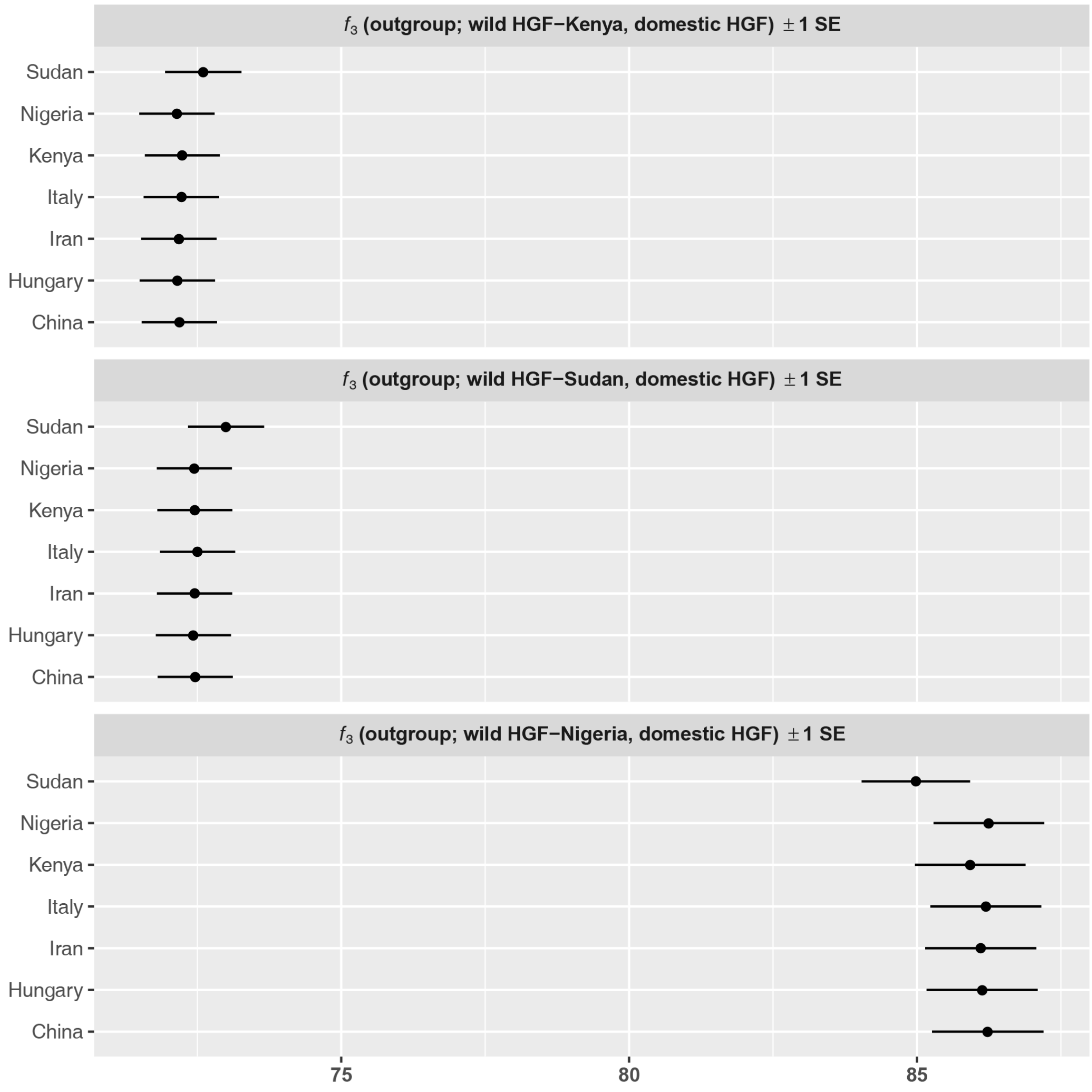

Figure S4. The outgroup f_3 statistics.

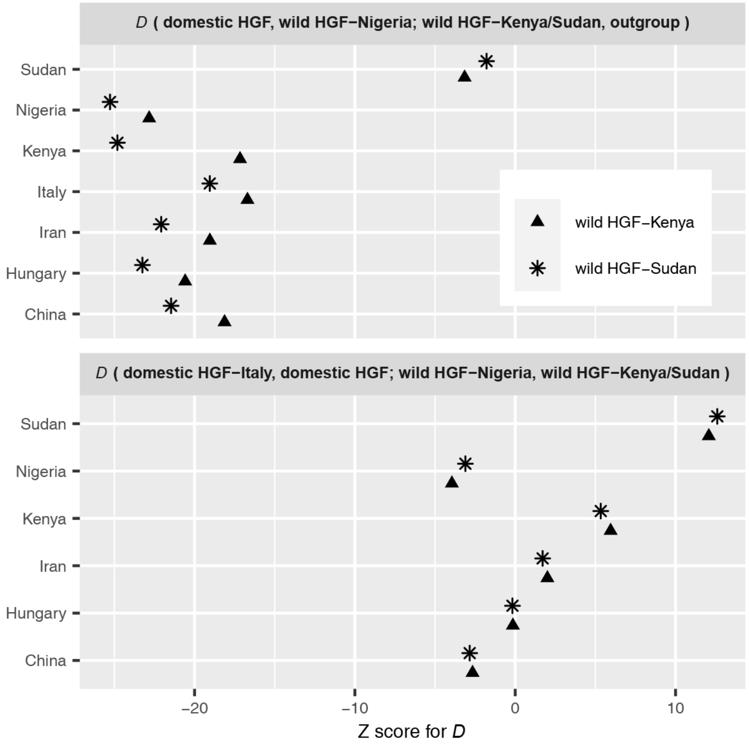

Figure S5. The D statistics.

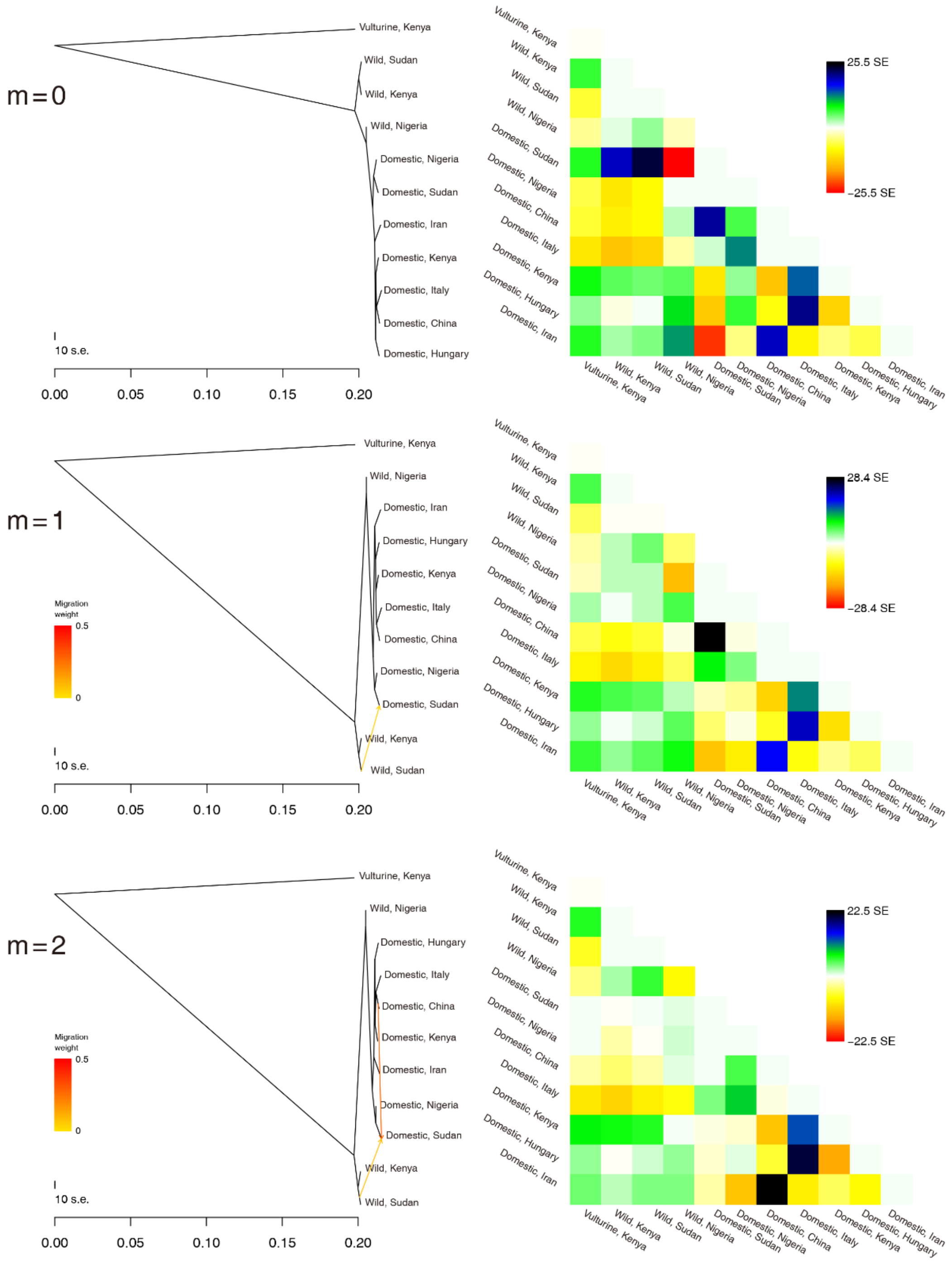

Figure S6. TreeMix analysis. When m=1 showing the gene flow from the wild to the domestic HGF in Sudan, the migration weight was 4.78% and the corresponding standard error was 6.85e-05. When m=2 indicating the additional gene flow from the domestic HGF of China to the domestic HGF of Sudan with the weight 28.64%, we thought this sort of overfitting.

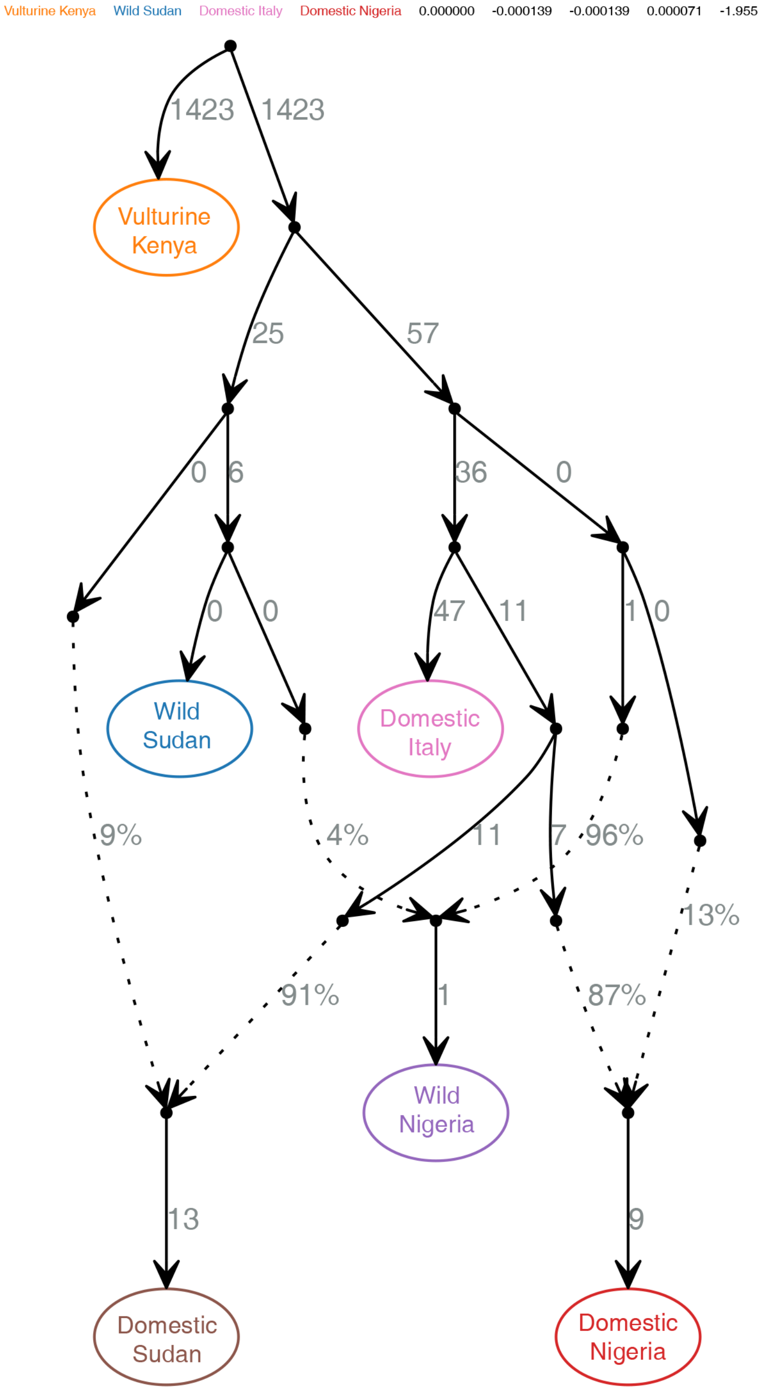

Figure S7. Admixture graph involving two wild HGF and three domestic HGF populations using qpGraph. The worst Z score was noted on the top.

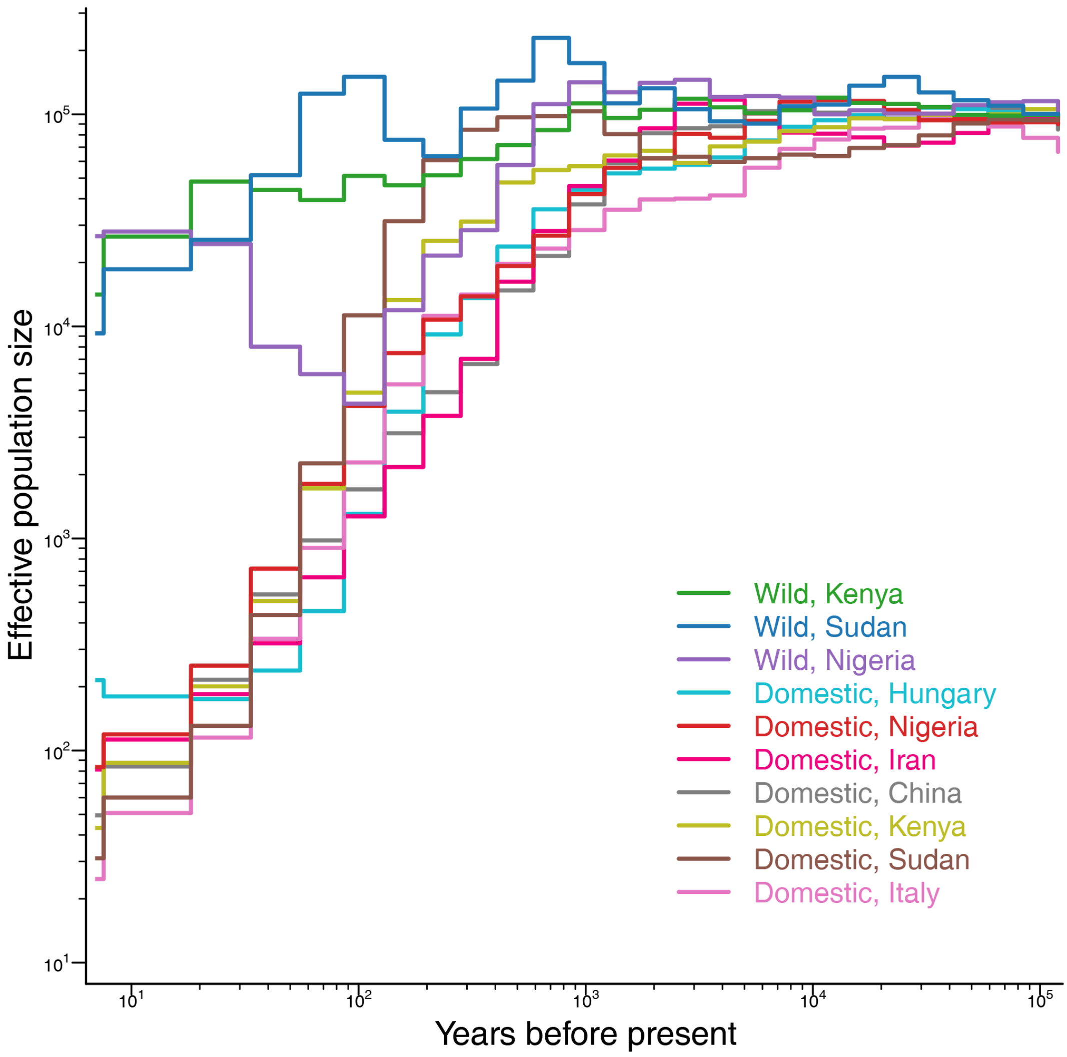

Figure S8. Dynamics of effective population sizes inferred by using PopSizeABC.

A

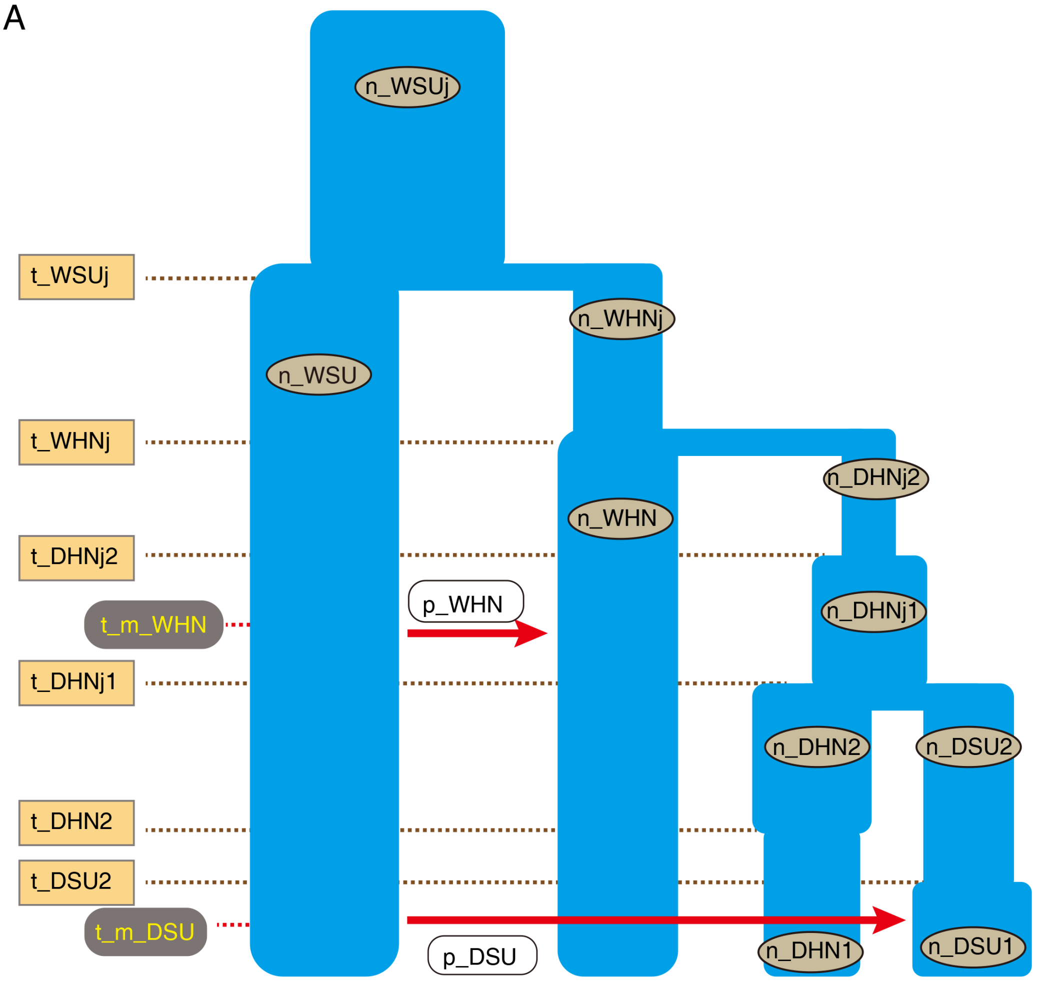

B

Parameter	Description	Estimation	Lower 95% CI	Upper 95% CI
n_WSUj	Ne common ancestor of all HGF	147099.966	85079.69	222987.47
n_WSU	Ne wild HGF from Sudan	221961.455	66796.09	396904.57
n_WHNj	Ne common ancestor of wild HGF from Nigeria and domestic HGF	115045.182	50000	225402.9
n_WHN	Ne wild HGF from Nigeria	61915.2408	50000	125896.04
n_DHNj2	Ne common ancestor of domestic HGF from Nigeria and Sudan before Ne change	9063.73428	5000	30033.95
n_DHNj1	Ne common ancestor of domestic HGF from Nigeria and Sudan after Ne change	17047.992	5000	29472.36
n_DHN2	Ne domestic HGF from Nigeria before Ne change	18438.26	1778.209	25341.167
n_DHN1	Ne domestic HGF from Nigeria after Ne change	3929.81482	1260.944	30134.694
n_DSU2	Ne domestic HGF from Sudan before Ne change	1972.4569	1130.466	17167.937
n_DSU1	Ne domestic HGF from Sudan after Ne change	38681.2766	1718.911	19489.815
p_WHN	Gene flow from wild HGF of Sudan lineage to wild HGF of Nigeria lineage	0.09316064	0.005940459	0.204160604
p_DSU	Gene flow from wild HGF of Sudan lineage to domestic HGF of Sudan lineage	0.05418048	0.0245547	0.14881277
t_WSUj	Time of split from common ancestors of all HGF	74413.1518	29672.27	168179.61
t_WHNj	Time of split from common ancestors of wild HGF from Nigeria and domestic HGF	5452.09546	2548.199	9916.37
t_DHNj2	Time of Ne change in common ancestor of domestic HGF from Nigeria and Sudan	1473.41937	253.4392	8927.9551
t_DHNj1	Time of split from common ancestors of domestic HGF from Nigeria and Sudan	1261.55217	162.4494	5180.9092
t_DHN2	Time of Ne change in domestic HGF of Nigeria lineage	723.622504	21.31706	2926.02646
t_DSU2	Time of Ne change in domestic HGF of Sudan lineage	100.933158	32.90332	2832.06223
t_m_WHN	Time of gene flow from wild HGF of Sudan lineage to wild HGF of Nigeria lineage	1448.91006	562.8476	8087.121
t_m_DSU	Time of gene flow from wild HGF of Sudan lineage to domestic HGF of Sudan lineage	154.126115	62.46897	3553.53764

Figure S9. Proposed model in momi2 analysis. *(A)*, Graph illustration for the topology and corresponding parameters. *(B)*, Estimation and 95% CI for each parameter in the model.

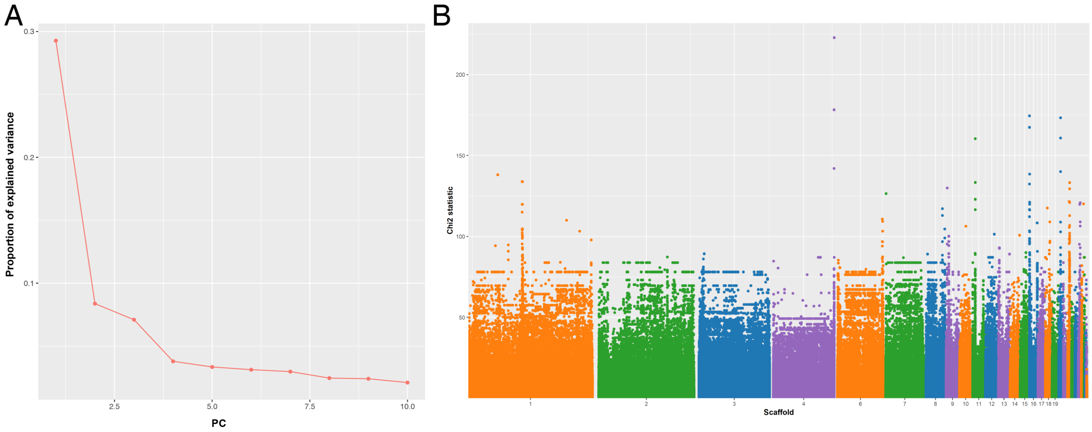

Figure S10. Scan of selective signals involved in HGF domestication and subsequent breeding. *(A)*, The first K=4 PCs that correspond to eigenvalues to the left of the straight line were selected in the subsequent analysis according to Cattell's rule. *(B)*, Manhattan plot for PCAdapt analysis.

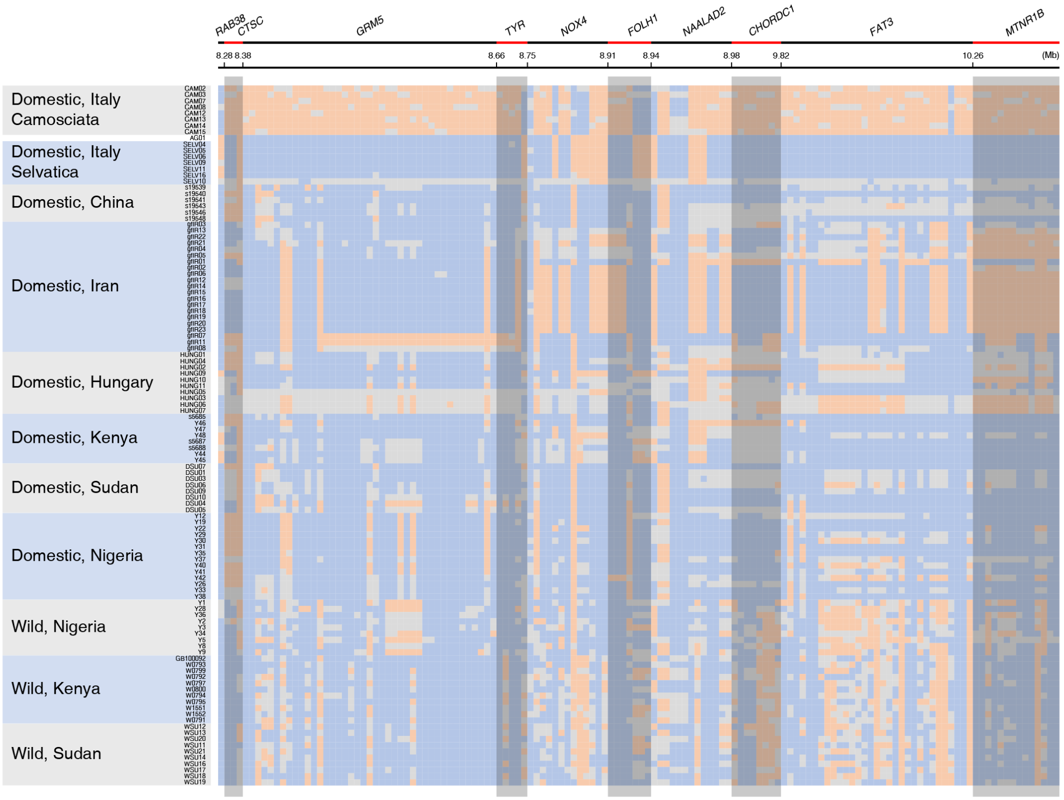

Figure S11. Genotype distribution for regions around *TYR* gene in the 9.5Mb LD block. The blue, grey and orange squares stand for the homozygous reference, heterozygous and homozygous alternative, respectively.

Table S1. Sampling and sequencing in genome assembly and annotation.

Sample ID (Sex)	Tissue	DNA-seq (PacBio)	DNA-seq (Illumina)	BioNano	Hi-C	RNA-seq (PacBio)	RNA-seq (Illumina)	Accession
YPT651 (male)	Cerebral cortex						7.0 Gb	SAMN15249096
YPT651 (male)	Heart						5.2 Gb	SAMN15249097
YPT651 (male)	Kidney						5.4 Gb	SAMN15249098
YPT651 (male)	Liver						5.4 Gb	SAMN15249099
YPT651 (male)	Lung						5.2 Gb	SAMN15249100
YPT651 (male)	Muscle						6.6 Gb	SAMN15249101
YPT651 (male)	Optic lobe						5.3 Gb	SAMN15249102
YPT651 (male)	Mixed brain						6.2 Gb	SAMN15249103
YPT651 (male)	Spleen						6.0 Gb	SAMN15249104
YPT651 (male)	Striatum						5.5 Gb	SAMN15249105
YPT651 (male)	Liver	128 Gb						SAMN15249094
YPT651 (male)	Liver		34.34 Gb					SAMN15249095
Bz-2 (male)	Testis					21.7 Gb		SAMN15249106
Dz-1 (male)	Testis					18.8 Gb		SAMN15249107
YP160303-001 (male)	Blood			200.8 Gb				SAMN15249325
YP160303-001 (male)	Blood				106 Gb			SAMN15249108

Table S2. Information of whole-genome re-sequencing for 129 samples.

SampleID	PopulationID	Mapping rate	Sequencing depth	Coverage by at least 1 base	Coverage by at least 5 bases	Longitude	Latitude	Species	Type/Breed	Description	Accession	Notes
s5711	WCK	91.44%	29.73	94.90%	93.80%	39.82	-3.27	*Guttera pucherani*	Outgroup	Wild-CGF, Arabuko-Sokoke Forest, Kenya	SAMN15249109	
s5712	WVK	84.55%	42.29	95.10%	94.10%	38.31	-3.45	*Acryllium vulturinum*	Outgroup	Wild-VGF-B, Taita-Taveta, Sagana Animal Sanctuary, Kenya	SAMN15249110	
D4708	WVK	96.90%	19.04	94.90%	92.90%	36.90	0.29	*Acryllium vulturinum*	Outgroup	Wild-VGF, Mpala, Kenya	SAMN15249111	
D4709	WVK	97.40%	25.36	94.90%	93.20%	36.90	0.29	*Acryllium vulturinum*	Outgroup	Wild-VGF, Mpala, Kenya	SAMN15249112	
D4710	WVK	97.07%	18.93	94.70%	92.20%	36.90	0.29	*Acryllium vulturinum*	Outgroup	Wild-VGF, Mpala, Kenya	SAMN15249113	
W1283	WVK	96.38%	21.86	95.10%	91.50%	36.90	0.29	*Acryllium vulturinum*	Outgroup	Wild-VGF, Mpala, Kenya	SAMN15249114	
W0791	WHK	97.18%	24.45	97.00%	95.70%	36.87	0.33	*Numida meleagris*	Wild	Wild-HGF, Mpala Ranch, Kenya	SAMN15249115	
W0792	WHK	96.83%	22.54	96.80%	92.00%	36.87	0.33	*Numida meleagris*	Wild	Wild-HGF, Mpala Ranch, Kenya	SAMN15249116	
W0793	WHK	96.81%	21.85	96.90%	92.50%	36.79	0.38	*Numida meleagris*	Wild	Wild-HGF, Laikipia, Kenya	SAMN15249117	
W0794	WHK	97.57%	19.50	96.90%	94.20%	36.90	0.29	*Numida meleagris*	Wild	Wild-HGF, Mpala, Kenya	SAMN15249118	
W0795	WHK	97.29%	22.85	96.60%	90.50%	36.87	0.33	*Numida meleagris*	Wild	Wild-HGF, Mpala, Kenya	SAMN15249119	
W0797	WHK	97.38%	24.75	97.10%	96.10%	37.91	0.02	*Numida meleagris*	Wild	Wild-HGF, Kenya	SAMN15249120	
W0799	WHK	97.16%	25.23	97.10%	94.20%	36.79	0.38	*Numida meleagris*	Wild	Wild-HGF, Laikipia, Kenya	SAMN15249121	
W0800	WHK	97.51%	24.69	97.00%	96.00%	36.90	0.29	*Numida meleagris*	Wild	Wild-HGF, Mpala, Kenya	SAMN15249122	
W1551	WHK	97.49%	23.80	97.00%	96.10%	37.91	0.02	*Numida meleagris*	Wild	Wild-HGF, Kenya	SAMN15249123	
W1552	WHK	96.34%	25.03	96.90%	92.40%	37.91	0.02	*Numida meleagris*	Wild	Wild-HGF, Kenya	SAMN15249124	
GB100092	WHK	97.79%	24.88	97.10%	93.50%	36.79	0.38	*Numida meleagris*	Wild	Wild-HGF, Laikipia, Kenya	SAMN15249125	
WSU11	WHSD	99.01%	18.82	97.20%	96.10%	32.53	15.60	*Numida meleagris*	Wild	Wild-HGF, Sudan	SAMN15249126	
WSU12	WHSD	98.92%	18.81	97.20%	96.30%	32.53	15.60	*Numida meleagris*	Wild	Wild-HGF, Sudan	SAMN15249127	
WSU13	WHSD	99.01%	18.68	97.20%	96.20%	32.53	15.60	*Numida meleagris*	Wild	Wild-HGF, Sudan	SAMN15249128	
WSU14	WHSD	98.89%	20.41	97.30%	96.30%	32.53	15.60	*Numida meleagris*	Wild	Wild-HGF, Sudan	SAMN15249129	
WSU16	WHSD	98.95%	18.42	97.20%	96.10%	32.53	15.60	*Numida meleagris*	Wild	Wild-HGF, Sudan	SAMN15249130	
WSU17	WHSD	98.98%	19.31	97.30%	95.90%	32.53	15.60	*Numida meleagris*	Wild	Wild-HGF, Sudan	SAMN15249131	
WSU18	WHSD	98.91%	19.32	97.30%	96.40%	32.53	15.60	*Numida meleagris*	Wild	Wild-HGF, Sudan	SAMN15249132	
WSU19	WHSD	98.95%	18.16	97.10%	95.90%	32.53	15.60	*Numida meleagris*	Wild	Wild-HGF, Sudan	SAMN15249133	
WSU20	WHSD	98.92%	18.50	97.20%	95.60%	32.53	15.60	*Numida meleagris*	Wild	Wild-HGF, Sudan	SAMN15249134	
WSU21	WHSD	99.06%	19.09	97.30%	96.20%	32.53	15.60	*Numida meleagris*	Wild	Wild-HGF, Sudan	SAMN15249135	
WSU15	WHSD	98.96%	19.17	97.40%	96.50%	32.53	15.60	*Numida meleagris*	Wild	Wild-HGF, Sudan	SAMN15249136	Going feral, removed in population grouping
Y10	WHN	88.49%	10.46	97.20%	91.40%	8.50	11.50	*Numida meleagris*	Wild	Wild-HGF, Kano, Nigeria	SAMN15249137	Going feral, removed in population grouping
Y4	WHN	87.22%	12.12	97.20%	93.70%	8.50	11.50	*Numida meleagris*	Wild	Wild-HGF, Kano, Nigeria	SAMN15249138	Going feral, removed in population grouping
Y27	WHN	91.96%	11.23	97.20%	91.10%	8.50	11.50	*Numida meleagris*	Wild	Wild-HGF, Kano, Nigeria	SAMN15249139	Going feral, removed in population grouping
Y1	WHN	90.04%	11.54	97.10%	91.00%	4.00	11.50	*Numida meleagris*	Wild	Wild-HGF, Kebbi, Nigeria	SAMN15249140	
Y2	WHN	95.37%	11.96	97.10%	92.60%	4.00	11.50	*Numida meleagris*	Wild	Wild-HGF, Kebbi, Nigeria	SAMN15249141	
Y28	WHN	94.12%	11.62	97.10%	92.10%	6.25	12.17	*Numida meleagris*	Wild	Wild-HGF, Zamfara, Nigeria	SAMN15249142	
Y3	WHN	97.53%	11.68	97.10%	91.70%	6.75	7.82	*Numida meleagris*	Wild	Wild-HGF, Lokoja, Kogi, Nigeria	SAMN15249143	
Y34	WHN	96.65%	11.91	97.10%	92.40%	5.25	13.08	*Numida meleagris*	Wild	Wild-HGF, Sokoto, Nigeria	SAMN15249144	
Y36	WHN	96.77%	10.91	97.10%	91.30%	6.25	12.17	*Numida meleagris*	Wild	Wild-HGF, Zamfara, Nigeria	SAMN15249145	
Y5	WHN	93.67%	11.47	97.10%	91.20%	6.75	7.82	*Numida meleagris*	Wild	Wild-HGF, Lokoja, Kogi, Nigeria	SAMN15249146	
Y8	WHN	92.29%	12.93	96.90%	91.20%	6.75	7.82	*Numida meleagris*	Wild	Wild-HGF, Lokoja, Kogi, Nigeria	SAMN15249147	
Y9	WHN	95.82%	12.37	97.10%	92.30%	5.25	13.08	*Numida meleagris*	Wild	Wild-HGF, Sokoto, Nigeria	SAMN15249148	
DSU02	DHSD	99.29%	17.55	97.20%	96.00%	32.53	15.60	*Numida meleagris*	Indigenous	Domestic-HGF, Sudan	SAMN15249149	Wild-caught, removed in population grouping
DSU04	DHSD	99.36%	17.88	97.30%	96.10%	32.53	15.60	*Numida meleagris*	Indigenous	Domestic-HGF, Sudan	SAMN15249150	
DSU07	DHSD	99.32%	16.63	97.30%	95.20%	32.53	15.60	*Numida meleagris*	Indigenous	Domestic-HGF, Sudan	SAMN15249151	
DSU08	DHSD	99.26%	16.44	97.20%	95.20%	32.53	15.60	*Numida meleagris*	Indigenous	Domestic-HGF, Sudan	SAMN15249152	Wild-caught, removed in population grouping
DSU01	DHSD	99.41%	17.53	97.30%	96.00%	32.53	15.60	*Numida meleagris*	Indigenous	Domestic-HGF, Sudan	SAMN15249153	
DSU03	DHSD	99.29%	16.76	97.20%	95.10%	32.53	15.60	*Numida meleagris*	Indigenous	Domestic-HGF, Sudan	SAMN15249154	
DSU05	DHSD	99.32%	17.82	97.30%	95.70%	32.53	15.60	*Numida meleagris*	Indigenous	Domestic-HGF, Sudan	SAMN15249155	
DSU06	DHSD	99.31%	17.38	97.40%	96.30%	32.53	15.60	*Numida meleagris*	Indigenous	Domestic-HGF, Sudan	SAMN15249156	
DSU09	DHSD	99.38%	16.88	97.30%	95.40%	32.53	15.60	*Numida meleagris*	Indigenous	Domestic-HGF, Sudan	SAMN15249157	
DSU10	DHSD	99.38%	18.03	97.30%	96.30%	32.53	15.60	*Numida meleagris*	Indigenous	Domestic-HGF, Sudan	SAMN15249158	
Y12	DHN	94.90%	11.27	97.20%	91.60%	4.00	11.50	*Numida meleagris*	Indigenous	Domestic-HGF, Kebbi, Nigeria	SAMN15249159	
Y19	DHN	89.88%	10.41	96.80%	85.50%	8.33	4.95	*Numida meleagris*	Indigenous	Domestic-HGF, Calabar, Nigeria	SAMN15249160	
Y22	DHN	94.55%	12.12	96.70%	88.60%	4.00	11.50	*Numida meleagris*	Indigenous	Domestic-HGF, Kebbi, Nigeria	SAMN15249161	
Y29	DHN	94.82%	10.88	97.20%	92.50%	10.50	8.00	*Numida meleagris*	Indigenous	Domestic-HGF, Taraba, Nigeria	SAMN15249162	
Y30	DHN	95.97%	11.59	97.10%	92.10%	8.50	11.50	*Numida meleagris*	Indigenous	Domestic-HGF, Kano, Nigeria	SAMN15249163	
Y31	DHN	96.14%	11.39	97.10%	90.60%	5.25	13.08	*Numida meleagris*	Indigenous	Domestic-HGF, Sokoto, Nigeria	SAMN15249164	
Y33	DHN	97.67%	10.98	97.00%	91.40%	5.25	13.08	*Numida meleagris*	Indigenous	Domestic-HGF, Sokoto, Nigeria	SAMN15249165	
Y38	DHN	88.59%	12.35	96.80%	90.30%	4.00	11.50	*Numida meleagris*	Indigenous	Domestic-HGF, Kebbi, Nigeria	SAMN15249166	
Y40	DHN	92.02%	11.54	97.00%	90.50%	7.00	6.33	*Numida meleagris*	Indigenous	Domestic-HGF, Anambra, Imo, Nigeria	SAMN15249167	
Y41	DHN	95.39%	12.12	97.10%	92.40%	6.25	12.17	*Numida meleagris*	Indigenous	Domestic-HGF, Zamfara, Nigeria	SAMN15249168	

Y26	DHN	95.83%	11.47	97.30%	94.20%	5.90	4.87	*Numida meleagris*	Indigenous	Domestic-HGF, Bayelsa, Nigeria	SAMN15249169	
Y35	DHN	98.21%	12.02	97.10%	92.70%	7.83	5.00	*Numida meleagris*	Indigenous	Domestic-HGF, Akwa Ibom, Nigeria	SAMN15249170	
Y37	DHN	88.56%	11.43	97.20%	93.50%	7.83	5.00	*Numida meleagris*	Indigenous	Domestic-HGF, Akwa Ibom, Nigeria	SAMN15249171	
Y42	DHN	91.94%	12.19	97.00%	91.10%	10.50	8.00	*Numida meleagris*	Indigenous	Domestic-HGF, Taraba, Nigeria	SAMN15249172	
AG01	DHIT	99.44%	19.93	97.30%	96.40%	11.55	45.57	*Numida meleagris*	Azzurra Ghigi	Domestic-HGF, Italy	SAMN15249173	
CAM02	DHIT	97.98%	25.60	97.30%	93.10%	13.83	42.17	*Numida meleagris*	Camosciata	Domestic-HGF, Italy	SAMN15249174	
CAM03	DHIT	98.72%	19.16	97.20%	95.30%	13.83	42.17	*Numida meleagris*	Camosciata	Domestic-HGF, Italy	SAMN15249175	
CAM07	DHIT	99.01%	26.29	97.30%	95.80%	13.83	42.17	*Numida meleagris*	Camosciata	Domestic-HGF, Italy	SAMN15249176	
CAM08	DHIT	98.18%	19.13	96.90%	88.30%	13.83	42.17	*Numida meleagris*	Camosciata	Domestic-HGF, Italy	SAMN15249177	
CAM12	DHIT	98.74%	18.48	97.10%	92.20%	13.83	42.17	*Numida meleagris*	Camosciata	Domestic-HGF, Italy	SAMN15249178	
CAM13	DHIT	98.35%	18.76	97.00%	89.40%	13.83	42.17	*Numida meleagris*	Camosciata	Domestic-HGF, Italy	SAMN15249179	
CAM14	DHIT	98.74%	16.04	97.10%	94.00%	13.83	42.17	*Numida meleagris*	Camosciata	Domestic-HGF, Italy	SAMN15249180	
CAM15	DHIT	98.80%	21.35	97.20%	93.50%	13.83	42.17	*Numida meleagris*	Camosciata	Domestic-HGF, Italy	SAMN15249181	
SELV04	DHIT	97.45%	19.32	97.10%	90.80%	12.55	43.19	*Numida meleagris*	Selvatica	Domestic-HGF, Italy	SAMN15249182	
SELV05	DHIT	98.30%	24.40	97.20%	93.20%	12.55	43.19	*Numida meleagris*	Selvatica	Domestic-HGF, Italy	SAMN15249183	
SELV06	DHIT	97.19%	25.53	97.40%	95.90%	12.55	43.19	*Numida meleagris*	Selvatica	Domestic-HGF, Italy	SAMN15249184	
SELV09	DHIT	97.89%	21.15	97.20%	92.70%	12.55	43.19	*Numida meleagris*	Selvatica	Domestic-HGF, Italy	SAMN15249185	
SELV10	DHIT	98.45%	22.43	97.10%	91.70%	12.55	43.19	*Numida meleagris*	Selvatica	Domestic-HGF, Italy	SAMN15249186	
SELV11	DHIT	97.92%	22.49	96.90%	89.60%	12.55	43.19	*Numida meleagris*	Selvatica	Domestic-HGF, Italy	SAMN15249187	
SELV16	DHIT	98.35%	21.48	97.30%	95.70%	12.55	43.19	*Numida meleagris*	Selvatica	Domestic-HGF, Italy	SAMN15249188	
Y44	DHK	91.82%	11.26	97.00%	90.40%	40.90	-2.27	*Numida meleagris*	Indigenous	Domestic-HGF, Lamu, Kenya	SAMN15249189	
Y45	DHK	90.39%	17.46	97.20%	94.20%	40.90	-2.27	*Numida meleagris*	Indigenous	Domestic-HGF, Lamu, Kenya	SAMN15249190	
Y46	DHK	90.38%	11.66	97.10%	92.90%	40.90	-2.27	*Numida meleagris*	Indigenous	Domestic-HGF, Lamu, Kenya	SAMN15249191	
Y47	DHK	93.16%	11.18	97.10%	91.30%	40.90	-2.27	*Numida meleagris*	Indigenous	Domestic-HGF, Lamu, Kenya	SAMN15249192	
Y48	DHK	98.09%	11.33	97.20%	91.20%	40.90	-2.27	*Numida meleagris*	Indigenous	Domestic-HGF, Lamu, Kenya	SAMN15249193	
s5685	DHK	95.61%	11.62	96.50%	86.70%	37.91	0.02	*Numida meleagris*	Indigenous	Domestic-HGF, Kenya	SAMN15249194	
s5687	DHK	86.85%	19.20	97.30%	95.60%	37.91	0.02	*Numida meleagris*	Indigenous	Domestic-HGF, Kenya	SAMN15249195	
s5688	DHK	84.83%	18.24	97.30%	95.20%	37.91	0.02	*Numida meleagris*	Indigenous	Domestic-HGF, Kenya	SAMN15249196	
s19539	DHC	85.99%	21.75	97.10%	95.20%	102.83	24.88	*Numida meleagris*	Indigenous	Domestic-HGF, China	SAMN15249197	
s19540	DHC	95.19%	13.58	96.80%	90.60%	102.83	24.88	*Numida meleagris*	Indigenous	Domestic-HGF, China	SAMN15249198	
s19541	DHC	89.18%	16.63	97.10%	94.30%	102.83	24.88	*Numida meleagris*	Indigenous	Domestic-HGF, China	SAMN15249199	
s19543	DHC	96.07%	19.74	97.30%	95.90%	102.83	24.88	*Numida meleagris*	Indigenous	Domestic-HGF, China	SAMN15249200	
s19546	DHC	85.74%	14.89	97.30%	94.80%	102.83	24.88	*Numida meleagris*	Indigenous	Domestic-HGF, China	SAMN15249201	
s19548	DHC	85.63%	24.17	97.30%	96.00%	102.83	24.88	*Numida meleagris*	Indigenous	Domestic-HGF, China	SAMN15249202	
HUNG12	DHH	97.69%	20.86	97.30%	91.60%	19.47	47.01	*Numida meleagris*	Indigenous	Domestic-HGF, Hungary - male	SAMN15249203	Removed in population grouping
HUNG13	DHH	97.38%	16.69	97.20%	90.60%	19.47	47.01	*Numida meleagris*	Indigenous	Domestic-HGF, Hungary - female	SAMN15249204	Removed in population grouping
HUNG01	DHH	99.20%	21.76	97.30%	93.90%	19.47	47.01	*Numida meleagris*	Indigenous	Domestic-HGF, Hungary	SAMN15249205	
HUNG02	DHH	99.12%	22.47	97.30%	96.30%	19.47	47.01	*Numida meleagris*	Indigenous	Domestic-HGF, Hungary	SAMN15249206	
HUNG03	DHH	99.29%	20.73	97.30%	95.30%	19.47	47.01	*Numida meleagris*	Indigenous	Domestic-HGF, Hungary	SAMN15249207	
HUNG04	DHH	99.16%	17.21	97.20%	95.70%	19.47	47.01	*Numida meleagris*	Indigenous	Domestic-HGF, Hungary	SAMN15249208	
HUNG05	DHH	99.24%	17.25	97.30%	96.10%	19.47	47.01	*Numida meleagris*	Indigenous	Domestic-HGF, Hungary	SAMN15249209	
HUNG06	DHH	99.12%	17.42	97.20%	94.30%	19.47	47.01	*Numida meleagris*	Indigenous	Domestic-HGF, Hungary	SAMN15249210	
HUNG07	DHH	98.91%	19.01	97.20%	95.00%	19.47	47.01	*Numida meleagris*	Indigenous	Domestic-HGF, Hungary	SAMN15249211	
HUNG09	DHH	99.03%	21.12	97.30%	96.10%	19.47	47.01	*Numida meleagris*	Indigenous	Domestic-HGF, Hungary	SAMN15249212	
HUNG10	DHH	99.06%	19.24	97.20%	94.50%	19.47	47.01	*Numida meleagris*	Indigenous	Domestic-HGF, Hungary	SAMN15249213	
HUNG11	DHH	99.04%	21.03	97.40%	96.30%	19.47	47.01	*Numida meleagris*	Indigenous	Domestic-HGF, Hungary	SAMN15249214	
gfIR09	DHIR	98.57%	21.45	97.20%	96.40%	46.27	38.10	*Numida meleagris*	Indigenous	Domestic-HGF, Tabriz, Iran	SAMN15249215	Removed in population grouping
gfIR10	DHIR	98.57%	22.66	97.20%	96.50%	46.27	38.10	*Numida meleagris*	Indigenous	Domestic-HGF, Tabriz, Iran	SAMN15249216	Removed in population grouping
gfIR01	DHIR	98.63%	18.76	97.20%	96.10%	52.58	29.59	*Numida meleagris*	Indigenous	Domestic-HGF, Shiraz, Iran	SAMN15249217	
gfIR02	DHIR	97.81%	19.82	97.30%	96.20%	52.58	29.59	*Numida meleagris*	Indigenous	Domestic-HGF, Shiraz, Iran	SAMN15249218	
gfIR03	DHIR	98.27%	24.78	97.30%	96.70%	57.75	28.03	*Numida meleagris*	Indigenous	Domestic-HGF, Kahnuj, Iran	SAMN15249219	
gfIR04	DHIR	98.18%	22.11	97.30%	96.50%	57.75	28.03	*Numida meleagris*	Indigenous	Domestic-HGF, Kahnuj, Iran	SAMN15249220	
gfIR05	DHIR	98.42%	27.41	97.30%	96.80%	57.75	28.03	*Numida meleagris*	Indigenous	Domestic-HGF, Kahnuj, Iran	SAMN15249221	
gfIR06	DHIR	98.72%	24.19	97.40%	96.80%	54.34	27.67	*Numida meleagris*	Indigenous	Domestic-HGF, Lar, Iran	SAMN15249222	
gfIR07	DHIR	98.23%	21.10	97.30%	96.00%	54.34	27.67	*Numida meleagris*	Indigenous	Domestic-HGF, Lar, Iran	SAMN15249223	
gfIR08	DHIR	98.47%	22.50	97.40%	96.70%	54.34	27.67	*Numida meleagris*	Indigenous	Domestic-HGF, Lar, Iran	SAMN15249224	
gfIR11	DHIR	98.65%	23.20	97.30%	96.60%	46.27	38.10	*Numida meleagris*	Indigenous	Domestic-HGF, Tabriz, Iran	SAMN15249225	
gfIR12	DHIR	98.68%	24.54	97.30%	96.70%	49.59	37.27	*Numida meleagris*	Indigenous	Domestic-HGF, Rasht, Iran	SAMN15249226	
gfIR13	DHIR	98.58%	24.61	97.30%	96.70%	49.59	37.27	*Numida meleagris*	Indigenous	Domestic-HGF, Rasht, Iran	SAMN15249227	
gfIR14	DHIR	98.48%	18.54	97.30%	96.10%	49.59	37.27	*Numida meleagris*	Indigenous	Domestic-HGF, Rasht, Iran	SAMN15249228	
gfIR15	DHIR	97.62%	19.07	97.30%	95.70%	46.27	38.10	*Numida meleagris*	Indigenous	Domestic-HGF, Tabriz, Iran	SAMN15249229	
gfIR16	DHIR	98.09%	23.57	97.40%	96.20%	46.27	38.10	*Numida meleagris*	Indigenous	Domestic-HGF, Tabriz, Iran	SAMN15249230	
gfIR17	DHIR	98.68%	22.76	97.30%	96.20%	46.27	38.10	*Numida meleagris*	Indigenous	Domestic-HGF, Tabriz, Iran	SAMN15249231	
gfIR18	DHIR	98.62%	22.41	97.30%	96.20%	46.27	38.10	*Numida meleagris*	Indigenous	Domestic-HGF, Tabriz, Iran	SAMN15249232	

gfIR19	DHIR	98.46%	22.28	97.30%	96.00%	46.27	38.10	*Numida meleagris*	Indigenous	Domestic-HGF, Tabriz, Iran	SAMN15249233
gfIR20	DHIR	98.13%	21.89	97.30%	96.00%	46.27	38.10	*Numida meleagris*	Indigenous	Domestic-HGF, Tabriz, Iran	SAMN15249234
gfIR21	DHIR	98.51%	24.24	97.20%	96.10%	46.27	38.10	*Numida meleagris*	Indigenous	Domestic-HGF, Tabriz, Iran	SAMN15249235
gfIR22	DHIR	98.33%	25.44	97.40%	96.80%	46.27	38.10	*Numida meleagris*	Indigenous	Domestic-HGF, Tabriz, Iran	SAMN15249236
gfIR23	DHIR	98.80%	20.78	97.30%	96.60%	46.27	38.10	*Numida meleagris*	Indigenous	Domestic-HGF, Tabriz, Iran	SAMN15249237

Table S3. Selective signals associated to PC1 and PC2 in PCAdapt analysis

sacffold	rsID	pos	Ref	Alt	PC associated	chi2.stat	description*
1	rs_1_14706432	14706432	G	A	PC1	77.99387	Intergenic;
1	rs_1_14708988	14708988	A	G	PC1	77.99387	Intergenic;
1	rs_1_14710825	14710825	G	A	PC1	77.99387	Intergenic;
1	rs_1_14714176	14714176	T	G	PC1	77.99387	Intergenic;
1	rs_1_14715235	14715235	G	T	PC1	77.99387	Intergenic;
1	rs_1_14719618	14719618	C	T	PC1	77.99387	Intergenic;
1	rs_1_14723224	14723224	A	G	PC1	77.99387	Intergenic;
1	rs_1_14723258	14723258	T	A	PC1	77.99387	Intergenic;
1	rs_1_14723387	14723387	T	G	PC1	77.99387	Intergenic;
1	rs_1_14723736	14723736	T	C	PC1	77.99387	Intergenic;
1	rs_1_14724293	14724293	T	C	PC1	77.99387	Intergenic;
1	rs_1_14724306	14724306	A	G	PC1	77.99387	Intergenic;
1	rs_1_14725988	14725988	A	G	PC1	77.99387	Intergenic;
1	rs_1_14726028	14726028	T	C	PC1	77.99387	Intergenic;
1	rs_1_14726477	14726477	A	G	PC1	77.99387	Intergenic;
1	rs_1_14726481	14726481	T	G	PC1	77.99387	Intergenic;
1	rs_1_14726820	14726820	G	C	PC1	77.99387	Intergenic;
1	rs_1_14727647	14727647	C	T	PC1	77.99387	Intergenic;
1	rs_1_15198864	15198864	A	G	PC1	77.99387	Intron;GRIA4;
1	rs_1_15199336	15199336	G	C	PC1	77.99387	Intron;GRIA4;
1	rs_1_15202116	15202116	T	C	PC1	77.99387	Intron;GRIA4;
1	rs_1_15203213	15203213	G	A	PC1	77.99387	Intron;GRIA4;
1	rs_1_15203214	15203214	C	A	PC1	77.99387	Intron;GRIA4;
1	rs_1_15203815	15203815	C	T	PC1	77.99387	Intron;GRIA4;
1	rs_1_15205795	15205795	T	G	PC1	77.99387	Intron;GRIA4;
1	rs_1_15205797	15205797	T	A	PC1	77.99387	Intron;GRIA4;
1	rs_1_15206210	15206210	A	G	PC1	77.99387	Intron;GRIA4;
1	rs_1_15206906	15206906	T	A	PC1	77.99387	Intron;GRIA4;
1	rs_1_15206907	15206907	C	A	PC1	77.99387	Intron;GRIA4;
1	rs_1_15207304	15207304	G	T	PC1	77.99387	Intron;GRIA4;
1	rs_1_15224849	15224849	C	T	PC1	77.99387	Intron;GRIA4;
1	rs_1_15225492	15225492	T	C	PC1	77.99387	Intron;GRIA4;
1	rs_1_15232820	15232820	C	T	PC1	77.99387	Intron;GRIA4;
1	rs_1_15248681	15248681	C	T	PC1	77.99387	Intron;GRIA4;
1	rs_1_15253266	15253266	A	G	PC1	77.99387	Intron;GRIA4;
1	rs_1_15254036	15254036	T	G	PC1	77.99387	Intron;GRIA4;
1	rs_1_15256950	15256950	C	T	PC1	77.99387	Intron;GRIA4;
1	rs_1_15257415	15257415	A	T	PC1	77.99387	Intron;GRIA4;
1	rs_1_15261010	15261010	A	G	PC1	77.99387	Intron;GRIA4;
1	rs_1_15415278	15415278	C	T	PC1	77.99387	Intergenic;
1	rs_1_15502611	15502611	C	T	PC1	77.99387	Intergenic;
1	rs_1_15502617	15502617	G	A	PC1	77.99387	Intergenic;
1	rs_1_15502810	15502810	A	C	PC1	77.99387	Intergenic;
1	rs_1_15502918	15502918	T	A	PC1	77.99387	Intergenic;
1	rs_1_15503886	15503886	T	C	PC1	77.99387	Intergenic;
1	rs_1_15504467	15504467	G	C	PC1	77.99387	Intergenic;
1	rs_1_15504766	15504766	C	T	PC1	77.99387	Intergenic;
1	rs_1_15505430	15505430	G	A	PC1	77.99387	Intergenic;
1	rs_1_15505511	15505511	A	G	PC1	77.99387	Intergenic;
1	rs_1_15505525	15505525	T	G	PC1	77.99387	Intergenic;
1	rs_1_15595580	15595580	A	G	PC1	77.99387	Intergenic;
1	rs_1_15596468	15596468	C	T	PC1	77.99387	Intergenic;
1	rs_1_15599512	15599512	T	G	PC1	77.99387	Intergenic;
1	rs_1_15719604	15719604	G	A	PC1	77.99387	Intron;GUCY1A2;
1	rs_1_15720969	15720969	C	T	PC1	77.99387	Intron;GUCY1A2;
1	rs_1_15721439	15721439	T	A	PC1	77.99387	Intron;GUCY1A2;
1	rs_1_15846438	15846438	T	G	PC1	77.99387	Intergenic;
1	rs_1_15975923	15975923	A	G	PC1	77.99387	Intron;ALKBH8;
1	rs_1_15975973	15975973	G	A	PC1	77.99387	Intron;ALKBH8;
1	rs_1_15976636	15976636	C	T	PC1	77.99387	Intron;ALKBH8;
1	rs_1_15977744	15977744	A	G	PC1	77.99387	Intron;ALKBH8;
1	rs_1_16751441	16751441	T	C	PC1	77.99387	Intergenic;
1	rs_1_16752011	16752011	A	G	PC1	77.99387	Intergenic;
1	rs_1_16785841	16785841	A	T	PC1	77.99387	Intergenic;
1	rs_1_16785910	16785910	T	C	PC1	77.99387	Intergenic;
1	rs_1_16785918	16785918	A	G	PC1	77.99387	Intergenic;
1	rs_1_16786458	16786458	G	A	PC1	77.99387	Intergenic;
1	rs_1_16786543	16786543	T	C	PC1	77.99387	Intergenic;
1	rs_1_16787246	16787246	A	G	PC1	77.99387	Intergenic;
1	rs_1_17723970	17723970	A	G	PC1	77.99387	Intergenic;
1	rs_1_17736137	17736137	C	T	PC1	77.99387	Intergenic;
1	rs_1_17747651	17747651	T	C	PC1	77.99387	CRYL1;downstream;
1	rs_1_17772155	17772155	T	C	PC1	77.99387	Intron;CRYL1;
1	rs_1_18381573	18381573	A	G	PC1	77.99387	Intergenic;
1	rs_1_18398814	18398814	A	G	PC1	77.99387	Intergenic;
1	rs_1_18399130	18399130	T	C	PC1	77.99387	Intergenic;
1	rs_1_18440451	18440451	G	A	PC1	77.99387	Intergenic;
1	rs_1_18441998	18441998	G	T	PC1	77.99387	Intergenic;
1	rs_1_18445026	18445026	T	C	PC1	77.99387	Intergenic;
1	rs_1_18502629	18502629	A	G	PC1	77.99387	Intergenic;
1	rs_1_18506329	18506329	A	G	PC1	77.99387	Intergenic;
1	rs_1_18509409	18509409	G	A	PC1	77.99387	Intergenic;
1	rs_1_18514941	18514941	C	A	PC1	77.99387	Intergenic;
1	rs_1_18515557	18515557	A	G	PC1	77.99387	Intergenic;
1	rs_1_18517319	18517319	C	T	PC1	77.99387	Intergenic;
1	rs_1_18542496	18542496	A	T	PC1	77.99387	Intergenic;

1	rs_1_18542497	18542497	G	C	PC1	77.99387	Intergenic;
1	rs_1_18549549	18549549	T	C	PC1	77.99387	Intergenic;
1	rs_1_18549600	18549600	G	A	PC1	77.99387	Intergenic;
1	rs_1_18551904	18551904	A	G	PC1	77.99387	Intergenic;
1	rs_1_18553261	18553261	C	G	PC1	77.99387	Intergenic;
1	rs_1_18553486	18553486	G	T	PC1	77.99387	Intergenic;
1	rs_1_18558241	18558241	T	C	PC1	77.99387	Intergenic;
1	rs_1_18559098	18559098	G	A	PC1	77.99387	Intergenic;
1	rs_1_18612323	18612323	G	A	PC1	77.99387	Intergenic;
1	rs_1_18617729	18617729	T	C	PC1	77.99387	Intergenic;
1	rs_1_18620058	18620058	T	C	PC1	77.99387	Intergenic;
1	rs_1_18621745	18621745	A	C	PC1	77.99387	Intergenic;
1	rs_1_18624341	18624341	A	G	PC1	77.99387	Intergenic;
1	rs_1_18624599	18624599	A	G	PC1	77.99387	Predicted;downstream;
1	rs_1_18625022	18625022	C	T	PC1	77.99387	Predicted;downstream;
1	rs_1_18649081	18649081	G	A	PC1	77.99387	Intergenic;
1	rs_1_18743944	18743944	A	G	PC1	77.99387	Intergenic;
1	rs_1_19196831	19196831	T	C	PC1	77.99387	TNFRSF19;downstream;
1	rs_1_20085635	20085635	C	T	PC1	77.99387	Intergenic;
1	rs_1_20086220	20086220	C	A	PC1	77.99387	Intergenic;
1	rs_1_20087804	20087804	T	G	PC1	77.99387	Intergenic;
1	rs_1_20131018	20131018	G	A	PC1	77.99387	WASF3;upstream;
1	rs_1_20964595	20964595	C	A	PC1	77.99387	Intergenic;
1	rs_1_20965504	20965504	A	G	PC1	77.99387	Intergenic;
1	rs_1_21525247	21525247	A	G	PC1	77.99387	Intergenic;
1	rs_1_22669381	22669381	G	A	PC1	77.99387	Exon;STARD13;STARD13;-;2;TAC;Synonymous;Tyr;Tyr;
1	rs_1_22677382	22677382	A	G	PC1	77.99387	Intron;STARD13;
1	rs_1_22677759	22677759	G	A	PC1	77.99387	Intron;STARD13;
1	rs_1_22682965	22682965	T	C	PC1	77.99387	Intron;STARD13;
1	rs_1_22683259	22683259	T	C	PC1	77.99387	Intron;STARD13;
1	rs_1_22684034	22684034	G	A	PC1	77.99387	Intron;STARD13;
1	rs_1_22684300	22684300	G	A	PC1	77.99387	Intron;STARD13;
1	rs_1_22684598	22684598	A	G	PC1	77.99387	Intron;STARD13;
1	rs_1_22686640	22686640	C	T	PC1	77.99387	Intron;STARD13;
1	rs_1_22688259	22688259	C	T	PC1	77.99387	Intron;STARD13;
1	rs_1_22688351	22688351	A	C	PC1	77.99387	Intron;STARD13;
1	rs_1_22735516	22735516	C	A	PC1	77.99387	Intron;STARD13;
1	rs_1_22783409	22783409	T	C	PC1	77.99387	Intergenic;
1	rs_1_22786627	22786627	G	C	PC1	77.99387	Intergenic;
1	rs_1_22832383	22832383	T	C	PC1	77.99387	Intergenic;
1	rs_1_22832722	22832722	G	T	PC1	77.99387	Intergenic;
1	rs_1_22832896	22832896	T	C	PC1	77.99387	Intergenic;
1	rs_1_22834506	22834506	G	A	PC1	77.99387	STARD13;downstream;
1	rs_1_25065923	25065923	G	T	PC1	77.99387	Intergenic;
1	rs_1_25067283	25067283	A	T	PC1	77.99387	Intergenic;
1	rs_1_25070128	25070128	T	G	PC1	77.99387	Intergenic;
1	rs_1_25081257	25081257	C	A	PC1	77.99387	Intergenic;
1	rs_1_25084400	25084400	A	C	PC1	77.99387	Intergenic;
1	rs_1_25093461	25093461	A	T	PC1	77.99387	Intergenic;
1	rs_1_25098031	25098031	G	C	PC1	77.99387	Intergenic;
1	rs_1_25186013	25186013	A	C	PC1	77.99387	Intergenic;
1	rs_1_25186380	25186380	C	T	PC1	77.99387	Intergenic;
1	rs_1_25186907	25186907	A	G	PC1	77.99387	Intergenic;
1	rs_1_25188387	25188387	T	C	PC1	77.99387	Intergenic;
1	rs_1_30404097	30404097	C	T	PC1	77.99387	RBBP6;upstream;
1	rs_1_37875333	37875333	G	A	PC1	77.99387	Intergenic;
1	rs_1_39304877	39304877	C	T	PC1	77.99387	Intergenic;
1	rs_1_41991171	41991171	A	C	PC1	77.99387	Intergenic;
1	rs_1_43435452	43435452	A	C	Others	94.18977	Intergenic;
1	rs_1_44177774	44177774	T	C	PC1	77.99387	Intergenic;
1	rs_1_44186787	44186787	A	G	PC1	77.99387	Intergenic;
1	rs_1_44330435	44330435	G	T	PC1	77.99387	Intergenic;
1	rs_1_44418231	44418231	G	T	PC1	77.99387	Intergenic;
1	rs_1_44443605	44443605	G	A	PC1	77.99387	Intergenic;
1	rs_1_45156699	45156699	C	T	PC1	77.99387	Intergenic;
1	rs_1_45161990	45161990	A	G	PC1	77.99387	Intergenic;
1	rs_1_45177214	45177214	G	A	PC1	77.99387	Intergenic;
1	rs_1_45187888	45187888	G	A	PC1	77.99387	Intergenic;
1	rs_1_45207358	45207358	G	C	PC1	77.99387	Intergenic;
1	rs_1_45216799	45216799	C	G	PC1	77.99387	Intergenic;
1	rs_1_45221728	45221728	A	T	PC1	77.99387	Intergenic;
1	rs_1_45233517	45233517	G	A	PC1	77.99387	Intergenic;
1	rs_1_45272092	45272092	G	A	PC1	77.99387	Intergenic;
1	rs_1_45274933	45274933	C	T	PC1	77.99387	Intergenic;
1	rs_1_45285570	45285570	G	A	PC1	77.99387	Intergenic;
1	rs_1_45302818	45302818	T	C	PC1	77.99387	Intergenic;
1	rs_1_45318292	45318292	C	T	PC1	77.99387	Intergenic;
1	rs_1_45324480	45324480	C	T	PC1	77.99387	Intergenic;
1	rs_1_45358862	45358862	T	C	PC1	77.99387	Intergenic;
1	rs_1_45359899	45359899	A	T	PC1	77.99387	Intergenic;
1	rs_1_45377052	45377052	T	A	PC1	77.99387	Intergenic;
1	rs_1_45394205	45394205	T	G	PC1	77.99387	Intergenic;
1	rs_1_45396872	45396872	C	T	PC1	77.99387	Intergenic;
1	rs_1_45398396	45398396	A	T	PC1	77.99387	Intergenic;
1	rs_1_45399416	45399416	G	A	PC1	77.99387	Intergenic;
1	rs_1_45399681	45399681	C	T	PC1	77.99387	Intergenic;
1	rs_1_45399792	45399792	C	T	PC1	77.99387	Intergenic;
1	rs_1_45404030	45404030	C	T	PC1	77.99387	Intergenic;

1	rs_1_45404517	45404517	A	G	PC1	77.99387		Intergenic;
1	rs_1_45407436	45407436	T	C	PC1	77.99387		Intergenic;
1	rs_1_45419218	45419218	C	T	PC1	77.99387		Intergenic;
1	rs_1_45419284	45419284	T	C	PC1	77.99387		Intergenic;
1	rs_1_45426880	45426880	A	C	PC1	77.99387		Intergenic;
1	rs_1_45427076	45427076	G	A	PC1	77.99387		Intergenic;
1	rs_1_45427327	45427327	A	G	PC1	77.99387		Intergenic;
1	rs_1_45428970	45428970	T	A	PC1	77.99387		Intergenic;
1	rs_1_45442425	45442425	T	A	PC1	77.99387		Intergenic;
1	rs_1_45442641	45442641	A	G	PC1	77.99387		Intergenic;
1	rs_1_45444734	45444734	A	G	PC1	77.99387		Intergenic;
1	rs_1_45448707	45448707	A	G	PC1	77.99387		Intergenic;
1	rs_1_45450185	45450185	T	C	PC1	77.99387		Intergenic;
1	rs_1_45452194	45452194	C	A	PC1	77.99387		Intergenic;
1	rs_1_45456897	45456897	G	A	PC1	77.99387		Intergenic;
1	rs_1_45460029	45460029	T	A	PC1	77.99387		Intergenic;
1	rs_1_45469290	45469290	C	T	PC1	77.99387		Intergenic;
1	rs_1_45472124	45472124	C	T	PC1	77.99387		Intergenic;
1	rs_1_45473425	45473425	A	G	PC1	77.99387		Intergenic;
1	rs_1_45474403	45474403	A	G	PC1	77.99387		Intergenic;
1	rs_1_45476578	45476578	T	C	PC1	77.99387		Intergenic;
1	rs_1_45476656	45476656	T	A	PC1	77.99387		Intergenic;
1	rs_1_45477195	45477195	G	A	PC1	77.99387		Intergenic;
1	rs_1_45485922	45485922	G	C	PC1	77.99387		Intergenic;
1	rs_1_45485990	45485990	T	C	PC1	77.99387		Intergenic;
1	rs_1_45487341	45487341	G	A	PC1	77.99387		Intergenic;
1	rs_1_45495662	45495662	C	G	PC1	77.99387		Intergenic;
1	rs_1_45496602	45496602	G	T	PC1	77.99387		Intergenic;
1	rs_1_45503466	45503466	C	T	PC1	77.99387		Intergenic;
1	rs_1_45504130	45504130	T	C	PC1	77.99387		Intergenic;
1	rs_1_45504990	45504990	C	T	PC1	77.99387		Intergenic;
1	rs_1_45508156	45508156	C	T	PC1	77.99387		SLIT1;downstream;
1	rs_1_45512033	45512033	A	C	PC1	77.99387		Intergenic;
1	rs_1_45514133	45514133	A	T	PC1	77.99387		Intergenic;
1	rs_1_45517008	45517008	T	C	PC1	77.99387		Exon;SLIT1;SLIT1;-;2;TCA;Synonymous;Ser;Ser;
1	rs_1_45517068	45517068	A	G	PC1	77.99387		Exon;SLIT1;SLIT1;-;2;AAT;Synonymous;Asn;Asn;
1	rs_1_45524410	45524410	T	C	PC1	77.99387		Intergenic;
1	rs_1_45524592	45524592	G	A	PC1	77.99387		Intergenic;
1	rs_1_45525893	45525893	G	A	PC1	77.99387		Intergenic;
1	rs_1_45525903	45525903	G	A	PC1	77.99387		Intergenic;
1	rs_1_45526096	45526096	C	G	PC1	77.99387		Intergenic;
1	rs_1_45526672	45526672	G	A	PC1	77.99387		Intergenic;
1	rs_1_45527751	45527751	T	C	PC1	77.99387		SLIT1;upstream;
1	rs_1_45528506	45528506	G	A	PC1	77.99387		SLIT1;upstream;
1	rs_1_45529105	45529105	A	T	PC1	77.99387		Intergenic;
1	rs_1_45529750	45529750	T	C	PC1	77.99387		Intergenic;
1	rs_1_45530676	45530676	T	C	PC1	77.99387		Intergenic;
1	rs_1_45530837	45530837	A	G	PC1	77.99387		Intergenic;
1	rs_1_45531205	45531205	G	A	PC1	77.99387		Intergenic;
1	rs_1_45537896	45537896	G	A	PC1	77.99387		Intergenic;
1	rs_1_45539291	45539291	A	G	PC1	77.99387		Intergenic;
1	rs_1_45542927	45542927	A	G	PC1	77.99387		Intergenic;
1	rs_1_45546608	45546608	G	A	PC1	77.99387		Intergenic;
1	rs_1_45551410	45551410	T	C	PC1	77.99387		Intergenic;
1	rs_1_45551628	45551628	A	G	PC1	77.99387		Intergenic;
1	rs_1_45552544	45552544	A	T	PC1	77.99387		Intergenic;
1	rs_1_45555328	45555328	G	A	PC1	77.99387		Intergenic;
1	rs_1_45562514	45562514	T	C	PC1	77.99387		Intergenic;
1	rs_1_45563716	45563716	A	T	PC1	77.99387		Intergenic;
1	rs_1_45565107	45565107	A	G	PC1	77.99387		Intergenic;
1	rs_1_45566129	45566129	T	C	PC1	77.99387		Intergenic;
1	rs_1_45568020	45568020	A	G	PC1	77.99387		Intergenic;
1	rs_1_45572483	45572483	T	C	PC1	77.99387		Intergenic;
1	rs_1_45574791	45574791	A	G	PC1	77.99387		Intergenic;
1	rs_1_45575140	45575140	A	G	PC1	77.99387		Intergenic;
1	rs_1_45578314	45578314	A	G	PC1	77.99387		Intergenic;
1	rs_1_45579053	45579053	G	A	PC1	77.99387		Intergenic;
1	rs_1_45579926	45579926	T	G	PC1	77.99387		Intergenic;
1	rs_1_45582003	45582003	A	T	PC1	77.99387		Intergenic;
1	rs_1_45583172	45583172	G	T	PC1	77.99387		Intergenic;
1	rs_1_45585686	45585686	T	A	PC1	77.99387		Intergenic;
1	rs_1_45588909	45588909	A	C	PC1	77.99387		Intergenic;
1	rs_1_45602240	45602240	T	C	PC1	77.99387		Intergenic;
1	rs_1_45602319	45602319	A	G	PC1	77.99387		Intergenic;
1	rs_1_45607526	45607526	G	A	PC1	77.99387		Intergenic;
1	rs_1_45608167	45608167	C	G	PC1	77.99387		Intergenic;
1	rs_1_45623455	45623455	G	C	PC1	77.99387		Intergenic;
1	rs_1_45632946	45632946	C	T	PC1	77.99387		Intergenic;
1	rs_1_45633500	45633500	A	C	PC1	77.99387		Intergenic;
1	rs_1_45633504	45633504	T	A	PC1	77.99387		Intergenic;
1	rs_1_45634763	45634763	T	C	PC1	77.99387		Intergenic;
1	rs_1_45639598	45639598	A	G	PC1	77.99387		Intergenic;
1	rs_1_45693521	45693521	A	G	PC1	77.99387		Intergenic;
1	rs_1_45773372	45773372	C	T	PC1	77.99387		Intergenic;
1	rs_1_46850726	46850726	G	C	PC1	77.99387		Intergenic;
1	rs_1_46871464	46871464	G	C	PC1	77.99387		Intergenic;
1	rs_1_46875250	46875250	C	G	PC1	77.99387		Intergenic;
1	rs_1_46882555	46882555	C	T	PC1	77.99387		Intergenic;

1	rs_1_46898840	46898840	G	A	PC1	77.99387	Intergenic;
1	rs_1_46899559	46899559	T	A	PC1	77.99387	Intergenic;
1	rs_1_46901771	46901771	A	G	PC1	77.99387	Intergenic;
1	rs_1_46904605	46904605	A	G	PC1	77.99387	Intergenic;
1	rs_1_46904916	46904916	C	A	PC1	77.99387	Intergenic;
1	rs_1_46904937	46904937	T	G	PC1	77.99387	Intergenic;
1	rs_1_46909680	46909680	G	A	PC1	77.99387	Intergenic;
1	rs_1_46909886	46909886	T	C	PC1	77.99387	Intergenic;
1	rs_1_46910841	46910841	G	A	PC1	77.99387	Intergenic;
1	rs_1_46911266	46911266	C	G	PC1	77.99387	Intergenic;
1	rs_1_47055402	47055402	T	A	PC1	77.99387	Intergenic;
1	rs_1_47057729	47057729	T	C	PC1	77.99387	Intergenic;
1	rs_1_47058101	47058101	T	C	PC1	77.99387	Intergenic;
1	rs_1_47058688	47058688	A	G	PC1	77.99387	Intergenic;
1	rs_1_47060136	47060136	G	T	PC1	77.99387	Intergenic;
1	rs_1_47064139	47064139	G	A	PC1	77.99387	Intergenic;
1	rs_1_47066665	47066665	T	C	PC1	77.99387	Intergenic;
1	rs_1_47067345	47067345	C	T	PC1	77.99387	Intergenic;
1	rs_1_47159926	47159926	G	A	PC1	77.99387	Intergenic;
1	rs_1_47160684	47160684	C	A	PC1	77.99387	Intergenic;
1	rs_1_47164108	47164108	G	A	PC1	77.99387	Intergenic;
1	rs_1_47164742	47164742	T	G	PC1	77.99387	Intergenic;
1	rs_1_47166534	47166534	G	T	PC1	77.99387	Intergenic;
1	rs_1_47166555	47166555	A	C	PC1	77.99387	Intergenic;
1	rs_1_47166581	47166581	A	G	PC1	77.99387	Intergenic;
1	rs_1_47168473	47168473	C	T	PC1	77.99387	Intergenic;
1	rs_1_47168627	47168627	A	G	PC1	77.99387	Intergenic;
1	rs_1_47168637	47168637	A	G	PC1	77.99387	Intergenic;
1	rs_1_47168643	47168643	T	C	PC1	77.99387	Intergenic;
1	rs_1_47168665	47168665	G	A	PC1	77.99387	Intergenic;
1	rs_1_47169497	47169497	G	A	PC1	77.99387	Intergenic;
1	rs_1_47169509	47169509	A	G	PC1	77.99387	Intergenic;
1	rs_1_47169737	47169737	G	T	PC1	77.99387	Intergenic;
1	rs_1_47170002	47170002	T	C	PC1	77.99387	Intergenic;
1	rs_1_47170525	47170525	C	T	PC1	77.99387	Intergenic;
1	rs_1_47170814	47170814	G	A	PC1	77.99387	Intergenic;
1	rs_1_47170902	47170902	G	T	PC1	77.99387	Intergenic;
1	rs_1_47170932	47170932	G	A	PC1	77.99387	Intergenic;
1	rs_1_47175485	47175485	C	T	PC1	77.99387	Intergenic;
1	rs_1_47179748	47179748	C	T	PC1	77.99387	Intergenic;
1	rs_1_47179775	47179775	A	G	PC1	77.99387	Intergenic;
1	rs_1_47180084	47180084	G	T	PC1	77.99387	Intergenic;
1	rs_1_47180743	47180743	A	G	PC1	77.99387	Intergenic;
1	rs_1_47180785	47180785	G	T	PC1	77.99387	Intergenic;
1	rs_1_47180852	47180852	G	A	PC1	77.99387	Intergenic;
1	rs_1_47181071	47181071	T	C	PC1	77.99387	Intergenic;
1	rs_1_47181354	47181354	T	A	PC1	77.99387	Intergenic;
1	rs_1_47181453	47181453	C	T	PC1	77.99387	Intergenic;
1	rs_1_47182252	47182252	C	T	PC1	77.99387	Intergenic;
1	rs_1_47182537	47182537	T	C	PC1	77.99387	Intergenic;
1	rs_1_47182575	47182575	A	G	PC1	77.99387	Intergenic;
1	rs_1_47182777	47182777	C	T	PC1	77.99387	Intergenic;
1	rs_1_47182830	47182830	A	G	PC1	77.99387	Intergenic;
1	rs_1_47183425	47183425	T	C	PC1	77.99387	Intergenic;
1	rs_1_47186137	47186137	G	T	PC1	77.99387	Intergenic;
1	rs_1_47186161	47186161	T	G	PC1	77.99387	Intergenic;
1	rs_1_47187513	47187513	A	G	PC1	77.99387	Intergenic;
1	rs_1_47188569	47188569	A	T	PC1	77.99387	Intergenic;
1	rs_1_47188662	47188662	A	G	PC1	77.99387	Intergenic;
1	rs_1_47190113	47190113	C	A	PC1	77.99387	Intergenic;
1	rs_1_47190497	47190497	C	G	PC1	77.99387	Intergenic;
1	rs_1_47191022	47191022	C	A	PC1	77.99387	Intergenic;
1	rs_1_47191117	47191117	C	T	PC1	77.99387	Intergenic;
1	rs_1_47191132	47191132	C	T	PC1	77.99387	Intergenic;
1	rs_1_47191186	47191186	T	C	PC1	77.99387	Intergenic;
1	rs_1_47191453	47191453	G	A	PC1	77.99387	Intergenic;
1	rs_1_47191922	47191922	A	T	PC1	77.99387	Intergenic;
1	rs_1_47192979	47192979	G	A	PC1	77.99387	Intergenic;
1	rs_1_47194017	47194017	C	T	PC1	77.99387	Intergenic;
1	rs_1_47194291	47194291	T	C	PC1	77.99387	Intergenic;
1	rs_1_47194380	47194380	A	G	PC1	77.99387	Intergenic;
1	rs_1_47194454	47194454	T	A	PC1	77.99387	Intergenic;
1	rs_1_47194961	47194961	T	C	PC1	77.99387	Intergenic;
1	rs_1_47195215	47195215	A	T	PC1	77.99387	Intergenic;
1	rs_1_47195295	47195295	G	A	PC1	77.99387	Intergenic;
1	rs_1_47195351	47195351	G	A	PC1	77.99387	Intergenic;
1	rs_1_47195917	47195917	G	A	PC1	77.99387	Intergenic;
1	rs_1_47199974	47199974	G	A	PC1	77.99387	Intergenic;
1	rs_1_47200256	47200256	C	T	PC1	77.99387	Intergenic;
1	rs_1_47201784	47201784	T	C	PC1	77.99387	Intergenic;
1	rs_1_47202210	47202210	G	A	PC1	77.99387	Intergenic;
1	rs_1_47202820	47202820	A	T	PC1	77.99387	Intergenic;
1	rs_1_47203790	47203790	C	A	PC1	77.99387	Intergenic;
1	rs_1_47204201	47204201	C	T	PC1	77.99387	Intergenic;
1	rs_1_47204366	47204366	T	A	PC1	77.99387	Intergenic;
1	rs_1_47205171	47205171	T	C	PC1	77.99387	Intergenic;
1	rs_1_47206960	47206960	A	G	PC1	77.99387	Intergenic;
1	rs_1_47206991	47206991	G	A	PC1	77.99387	Intergenic;

1	rs_1_47207119	47207119	T	C	PC1	77.99387	Intergenic;
1	rs_1_47207786	47207786	T	C	PC1	77.99387	Intergenic;
1	rs_1_47209261	47209261	C	T	PC1	77.99387	Intergenic;
1	rs_1_47210576	47210576	T	C	PC1	77.99387	Intergenic;
1	rs_1_47210696	47210696	T	C	PC1	77.99387	Intergenic;
1	rs_1_47210727	47210727	G	A	PC1	77.99387	Intergenic;
1	rs_1_47211068	47211068	T	C	PC1	77.99387	Intergenic;
1	rs_1_47211378	47211378	T	G	PC1	77.99387	Intergenic;
1	rs_1_47213486	47213486	G	T	PC1	77.99387	Intergenic;
1	rs_1_47213643	47213643	G	A	PC1	77.99387	Intergenic;
1	rs_1_47214016	47214016	G	A	PC1	77.99387	Intergenic;
1	rs_1_47214311	47214311	A	T	PC1	77.99387	Intergenic;
1	rs_1_47214334	47214334	A	C	PC1	77.99387	Intergenic;
1	rs_1_47214563	47214563	G	A	PC1	77.99387	Intergenic;
1	rs_1_47214627	47214627	G	A	PC1	77.99387	Intergenic;
1	rs_1_47214640	47214640	A	T	PC1	77.99387	Intergenic;
1	rs_1_47214659	47214659	A	G	PC1	77.99387	Intergenic;
1	rs_1_47214834	47214834	G	A	PC1	77.99387	Intergenic;
1	rs_1_47215445	47215445	T	A	PC1	77.99387	Intergenic;
1	rs_1_47215573	47215573	T	C	PC1	77.99387	Intergenic;
1	rs_1_47216131	47216131	G	A	PC1	77.99387	Intergenic;
1	rs_1_47216176	47216176	A	G	PC1	77.99387	Intergenic;
1	rs_1_47216270	47216270	C	T	PC1	77.99387	Intergenic;
1	rs_1_47216489	47216489	A	G	PC1	77.99387	Intergenic;
1	rs_1_47219050	47219050	C	T	PC1	77.99387	Intergenic;
1	rs_1_47219103	47219103	G	C	PC1	77.99387	Intergenic;
1	rs_1_47219107	47219107	G	T	PC1	77.99387	Intergenic;
1	rs_1_47219153	47219153	G	C	PC1	77.99387	Intergenic;
1	rs_1_47219234	47219234	T	C	PC1	77.99387	Intergenic;
1	rs_1_47219803	47219803	T	A	PC1	77.99387	Intergenic;
1	rs_1_47219912	47219912	C	T	PC1	77.99387	Intergenic;
1	rs_1_47220085	47220085	A	G	PC1	77.99387	Intergenic;
1	rs_1_47220301	47220301	C	G	PC1	77.99387	Intergenic;
1	rs_1_47220352	47220352	T	G	PC1	77.99387	Intergenic;
1	rs_1_47220426	47220426	T	G	PC1	77.99387	Intergenic;
1	rs_1_47220428	47220428	T	C	PC1	77.99387	Intergenic;
1	rs_1_47220787	47220787	G	A	PC1	77.99387	Intergenic;
1	rs_1_47220985	47220985	G	T	PC1	77.99387	Intergenic;
1	rs_1_47221455	47221455	C	T	PC1	77.99387	Intergenic;
1	rs_1_47221548	47221548	C	T	PC1	77.99387	Intergenic;
1	rs_1_47221763	47221763	A	C	PC1	77.99387	Intergenic;
1	rs_1_47221827	47221827	G	C	PC1	77.99387	Intergenic;
1	rs_1_47221842	47221842	A	G	PC1	77.99387	Intergenic;
1	rs_1_47221889	47221889	T	A	PC1	77.99387	Intergenic;
1	rs_1_47221928	47221928	G	A	PC1	77.99387	Intergenic;
1	rs_1_47222510	47222510	G	C	PC1	77.99387	Intergenic;
1	rs_1_47222550	47222550	G	T	PC1	77.99387	Intergenic;
1	rs_1_47222587	47222587	A	G	PC1	77.99387	Intergenic;
1	rs_1_47222598	47222598	G	A	PC1	77.99387	Intergenic;
1	rs_1_47222684	47222684	G	A	PC1	77.99387	Intergenic;
1	rs_1_47222768	47222768	G	A	PC1	77.99387	Intergenic;
1	rs_1_47222775	47222775	C	T	PC1	77.99387	Intergenic;
1	rs_1_47222785	47222785	T	C	PC1	77.99387	Intergenic;
1	rs_1_47222856	47222856	A	T	PC1	77.99387	Intergenic;
1	rs_1_47223144	47223144	G	A	PC1	77.99387	Intergenic;
1	rs_1_47223232	47223232	T	C	PC1	77.99387	Intergenic;
1	rs_1_47223251	47223251	C	A	PC1	77.99387	Intergenic;
1	rs_1_47223266	47223266	T	G	PC1	77.99387	Intergenic;
1	rs_1_47378037	47378037	A	G	Others	138.1093	Intergenic;
1	rs_1_48031178	48031178	A	T	PC1	77.99387	Intergenic;
1	rs_1_48082431	48082431	T	A	PC1	77.99387	Intergenic;
1	rs_1_48084924	48084924	A	G	PC1	77.99387	Intergenic;
1	rs_1_48087591	48087591	G	A	PC1	77.99387	Intergenic;
1	rs_1_48089774	48089774	A	G	PC1	77.99387	Intergenic;
1	rs_1_48090434	48090434	A	G	PC1	77.99387	Intergenic;
1	rs_1_48101996	48101996	T	C	PC1	77.99387	Intergenic;
1	rs_1_48103570	48103570	C	A	PC1	77.99387	Intergenic;
1	rs_1_48111175	48111175	G	A	PC1	77.99387	Intergenic;
1	rs_1_48113558	48113558	A	G	PC1	77.99387	Intergenic;
1	rs_1_48113605	48113605	A	G	PC1	77.99387	Intergenic;
1	rs_1_48113642	48113642	C	G	PC1	77.99387	Intergenic;
1	rs_1_48127853	48127853	T	C	PC1	77.99387	Intergenic;
1	rs_1_48133199	48133199	T	G	PC1	77.99387	Intergenic;
1	rs_1_48324764	48324764	T	C	PC1	77.99387	Intergenic;
1	rs_1_48343830	48343830	A	G	PC1	77.99387	Intergenic;
1	rs_1_48354260	48354260	T	G	PC1	77.99387	Intergenic;
1	rs_1_48357771	48357771	A	G	PC1	77.99387	Intergenic;
1	rs_1_48489473	48489473	A	G	PC1	77.99387	Intergenic;
1	rs_1_48576412	48576412	G	C	PC1	77.99387	Intergenic;
1	rs_1_48677699	48677699	T	C	PC1	77.99387	Intergenic;
1	rs_1_49486906	49486906	A	G	PC1	77.99387	Intergenic;
1	rs_1_49539956	49539956	A	G	PC1	77.99387	Intergenic;
1	rs_1_49539987	49539987	C	T	PC1	77.99387	Intergenic;
1	rs_1_49765232	49765232	T	G	PC1	77.99387	Intergenic;
1	rs_1_49768568	49768568	C	A	PC1	77.99387	Intergenic;
1	rs_1_49769065	49769065	T	C	PC1	77.99387	Intergenic;
1	rs_1_49773049	49773049	T	C	PC1	77.99387	Intergenic;
1	rs_1_49774374	49774374	A	G	PC1	77.99387	Intergenic;

1	rs_1_49774474	49774474	A	G	PC1	77.99387	Intergenic;
1	rs_1_49776655	49776655	A	C	PC1	77.99387	Intergenic;
1	rs_1_49776825	49776825	C	T	PC1	77.99387	Intergenic;
1	rs_1_49777803	49777803	T	A	PC1	77.99387	Intergenic;
1	rs_1_49778240	49778240	G	T	PC1	77.99387	Intergenic;
1	rs_1_49778486	49778486	G	T	PC1	77.99387	Intergenic;
1	rs_1_49778609	49778609	T	G	PC1	77.99387	Intergenic;
1	rs_1_49778613	49778613	T	G	PC1	77.99387	Intergenic;
1	rs_1_49779890	49779890	G	A	PC1	77.99387	Intergenic;
1	rs_1_49780010	49780010	A	T	PC1	77.99387	Intergenic;
1	rs_1_49780134	49780134	G	A	PC1	77.99387	Intergenic;
1	rs_1_49800970	49800970	A	G	PC1	77.99387	Intergenic;
1	rs_1_53915047	53915047	T	C	PC1	77.99387	Intron;FGF14;
1	rs_1_55846109	55846109	G	A	PC1	77.99387	Intergenic;
1	rs_1_55857486	55857486	T	C	PC1	77.99387	Intergenic;
1	rs_1_56389105	56389105	G	T	PC1	77.99387	Intergenic;
1	rs_1_56567039	56567039	G	A	PC1	77.99387	Intron;PAN1;
1	rs_1_56568489	56568489	G	C	PC1	77.99387	Intron;PAN1;
1	rs_1_56585484	56585484	G	A	PC1	77.99387	Intron;PAN1;
1	rs_1_56586009	56586009	G	A	PC1	77.99387	Intron;PAN1;
1	rs_1_56630115	56630115	C	T	PC1	77.99387	Intron;PAN1;
1	rs_1_56696050	56696050	G	A	PC1	77.99387	Intron;PAN1;
1	rs_1_56701495	56701495	C	A	PC1	77.99387	Intron;PAN1;
1	rs_1_56702328	56702328	T	G	PC1	77.99387	Intron;PAN1;
1	rs_1_56702641	56702641	T	C	PC1	77.99387	Intron;PAN1;
1	rs_1_64023025	64023025	T	G	PC2	85.47951	Intergenic;
1	rs_1_64023500	64023500	G	A	PC2	90.81798	Intergenic;
1	rs_1_64023580	64023580	A	T	PC2	85.47951	Intergenic;
1	rs_1_64025621	64025621	G	T	PC2	94.73898	H2AC21;downstream;
1	rs_1_69482719	69482719	A	T	PC1	77.99387	Intergenic;
1	rs_1_86291797	86291797	C	A	PC2	82.59497	Intron;KDM6A;
1	rs_1_86302173	86302173	G	A	PC2	103.8819	Intron;KDM6A;
1	rs_1_86314285	86314285	A	C	PC2	103.8819	Intron;KDM6A;
1	rs_1_86324997	86324997	C	G	PC2	103.8819	Intron;KDM6A;
1	rs_1_86331874	86331874	A	G	PC2	103.8819	Intron;KDM6A;
1	rs_1_86350620	86350620	T	C	PC2	95.34907	Intron;KDM6A;
1	rs_1_86353307	86353307	A	G	PC2	103.8819	Intron;KDM6A;
1	rs_1_86359255	86359255	C	T	PC2	103.8819	Intron;KDM6A;
1	rs_1_86367354	86367354	C	G	PC2	103.8819	Intron;KDM6A;
1	rs_1_86371089	86371089	A	G	PC2	103.8819	Intron;KDM6A;
1	rs_1_86373852	86373852	G	A	PC2	103.8819	Intron;KDM6A;
1	rs_1_86374304	86374304	A	T	PC2	103.8819	Intron;KDM6A;
1	rs_1_86376063	86376063	G	A	PC2	103.8819	Intron;KDM6A;
1	rs_1_86381156	86381156	C	G	PC2	119.8231	Intron;KDM6A;
1	rs_1_86384295	86384295	A	G	PC2	114.8938	Intron;KDM6A;
1	rs_1_86388003	86388003	G	A	PC2	104.5839	Intron;KDM6A;
1	rs_1_86396036	86396036	A	G	PC2	119.8231	Intron;KDM6A;
1	rs_1_86402876	86402876	G	A	PC2	79.59243	Intron;KDM6A;
1	rs_1_86420348	86420348	C	T	PC2	133.8701	Intron;DIPK2B;
1	rs_1_86425167	86425167	T	C	PC2	79.59243	Intron;DIPK2B;
1	rs_1_86426243	86426243	A	G	PC2	85.42286	Exon;DIPK2B;DIPK2B;-;1;TTG;Nonsynonymous;Leu;Ser;
1	rs_1_86427117	86427117	A	G	PC2	102.1175	DIPK2B;upstream;
1	rs_1_86427447	86427447	C	T	PC2	87.92252	DIPK2B;upstream;
1	rs_1_86427722	86427722	C	T	PC2	119.8231	DIPK2B;upstream;
1	rs_1_86437959	86437959	A	G	PC2	119.8231	Intergenic;
1	rs_1_86457754	86457754	C	G	PC2	119.8231	Intergenic;
1	rs_1_86477051	86477051	T	C	PC2	119.8231	Intergenic;
1	rs_1_86494020	86494020	A	G	PC2	119.8231	Intergenic;
1	rs_1_86494620	86494620	C	T	PC2	104.5655	Intergenic;
1	rs_1_86499555	86499555	C	T	PC2	119.8231	Intergenic;
1	rs_1_86499638	86499638	A	G	PC2	119.8231	Intergenic;
1	rs_1_86508610	86508610	C	T	PC2	119.8231	Intergenic;
1	rs_1_86518496	86518496	G	A	PC2	119.8231	Intergenic;
1	rs_1_86544539	86544539	G	A	PC2	119.8231	Intergenic;
1	rs_1_86557574	86557574	G	A	PC2	119.8231	Intergenic;
1	rs_1_86559483	86559483	G	A	PC2	100.678	Intergenic;
1	rs_1_86565160	86565160	T	C	PC2	119.8231	Intergenic;
1	rs_1_86572026	86572026	C	A	PC2	119.8231	Intergenic;
1	rs_1_86575788	86575788	C	T	PC2	100.678	Intergenic;
1	rs_1_86609444	86609444	C	T	PC2	104.1524	Intergenic;
1	rs_1_86627483	86627483	G	A	PC2	119.8231	Intergenic;
1	rs_1_86641538	86641538	A	G	PC2	119.8231	Intergenic;
1	rs_1_86643593	86643593	T	C	PC2	119.8231	Intergenic;
1	rs_1_86649336	86649336	G	A	PC2	104.5839	Intergenic;
1	rs_1_86650770	86650770	C	T	PC2	87.79358	Intergenic;
1	rs_1_86656329	86656329	G	A	PC2	119.8231	Intergenic;
1	rs_1_86657305	86657305	G	A	PC2	104.5839	Intergenic;
1	rs_1_86658079	86658079	T	A	PC2	98.78885	Intergenic;
1	rs_1_86658492	86658492	T	C	PC2	98.78885	Intergenic;
1	rs_1_86673442	86673442	C	T	PC2	92.78108	Intergenic;
1	rs_1_86680837	86680837	A	T	PC2	82.88914	Intergenic;
1	rs_1_86685903	86685903	C	G	PC2	92.78108	Intergenic;
1	rs_1_86686756	86686756	C	T	PC2	82.88914	Intergenic;
1	rs_1_86700301	86700301	G	A	PC2	119.8231	Intergenic;
1	rs_1_86703413	86703413	T	C	PC2	133.8701	Intergenic;
1	rs_1_86706289	86706289	C	T	PC2	119.8231	Intergenic;
1	rs_1_86707645	86707645	T	G	PC2	119.8231	Intergenic;
1	rs_1_86711604	86711604	A	G	PC2	119.8231	Intergenic;

1	rs_1_86714936	86714936	A	G	PC2	92.80534	Intergenic;
1	rs_1_86715205	86715205	C	A	PC2	92.05547	Intergenic;
1	rs_1_86716664	86716664	G	T	PC2	104.5839	Intergenic;
1	rs_1_86717718	86717718	G	C	PC2	119.8231	Intergenic;
1	rs_1_86720054	86720054	T	C	PC1	77.99387	Intergenic;
1	rs_1_86721339	86721339	A	G	PC2	119.8231	Intergenic;
1	rs_1_86725396	86725396	G	A	PC2	85.96172	Intergenic;
1	rs_1_86727626	86727626	G	A	PC2	119.8231	Intergenic;
1	rs_1_86734960	86734960	C	T	PC2	119.8231	Intergenic;
1	rs_1_86749514	86749514	G	A	PC2	93.05331	Intergenic;
1	rs_1_86751403	86751403	C	A	PC2	119.8231	Intergenic;
1	rs_1_86758643	86758643	G	T	PC2	85.6771	Intergenic;
1	rs_1_86762530	86762530	A	C	PC2	119.8231	Intergenic;
1	rs_1_86774719	86774719	A	G	PC2	119.8231	Intergenic;
1	rs_1_86784662	86784662	C	T	PC2	119.8231	Intergenic;
1	rs_1_86789394	86789394	G	A	PC2	119.8231	Intergenic;
1	rs_1_86794930	86794930	G	A	PC2	119.8231	Intergenic;
1	rs_1_86796101	86796101	A	G	PC2	119.8231	Intergenic;
1	rs_1_86798243	86798243	C	A	PC2	119.8231	Intergenic;
1	rs_1_86817804	86817804	T	A	PC2	119.8231	Intergenic;
1	rs_1_86828892	86828892	A	G	PC2	119.8231	ICOSLG;upstream;
1	rs_1_86831551	86831551	A	G	PC2	133.8701	ICOSLG;upstream;
1	rs_1_86843723	86843723	C	T	PC2	80.1227	Intron;ICOSLG;
1	rs_1_86846894	86846894	A	G	PC2	100.0227	Intron;ICOSLG;
1	rs_1_86847933	86847933	C	T	PC2	100.0227	ICOSLG;downstream;
1	rs_1_86847973	86847973	A	C	PC2	88.40088	ICOSLG;downstream;
1	rs_1_86856360	86856360	G	A	PC2	85.17102	ICOSLG;downstream;
1	rs_1_86862419	86862419	A	G	PC2	100.0227	Intergenic;
1	rs_1_86862617	86862617	C	T	PC2	100.0227	Intergenic;
1	rs_1_86864510	86864510	T	C	PC2	100.0227	Intergenic;
1	rs_1_86865354	86865354	A	G	PC2	76.96081	Intergenic;
1	rs_1_86865941	86865941	T	C	PC2	100.0227	Intergenic;
1	rs_1_86866844	86866844	G	A	PC2	100.0227	Intergenic;
1	rs_1_86868741	86868741	G	A	PC2	88.1183	Intergenic;
1	rs_1_86869800	86869800	T	C	PC2	119.8231	Intergenic;
1	rs_1_86879572	86879572	T	C	PC2	119.8231	VTCN1;upstream;
1	rs_1_86879981	86879981	T	C	PC2	119.8231	VTCN1;upstream;
1	rs_1_86880844	86880844	A	G	PC2	119.8231	Intergenic;
1	rs_1_86882159	86882159	T	C	PC2	119.8231	Intergenic;
1	rs_1_86884583	86884583	A	C	PC2	119.8231	Intergenic;
1	rs_1_86886621	86886621	C	T	PC2	119.8231	Intron;VTCN1;
1	rs_1_86886651	86886651	G	C	PC2	119.8231	Intron;VTCN1;
1	rs_1_86886980	86886980	A	T	PC2	119.8231	Intron;VTCN1;
1	rs_1_86887465	86887465	T	A	PC2	119.8231	Intron;VTCN1;
1	rs_1_86891104	86891104	G	A	PC2	119.8231	GATD3A;downstream;
1	rs_1_86896374	86896374	A	C	PC2	119.8231	VTCN1;downstream;PWP2;downstream;
1	rs_1_86899809	86899809	A	T	PC2	133.8701	Intron;GATD3A;
1	rs_1_87179309	87179309	G	C	PC2	88.51115	PDXK;upstream;
1	rs_1_87187727	87187727	T	A	PC2	80.70888	PDXK;upstream;RRP1B;downstream;
1	rs_1_87188686	87188686	C	G	PC2	80.70888	PDXK;upstream;RRP1B;downstream;
1	rs_1_87197715	87197715	C	T	PC2	80.70888	Intron;RRP1B;
1	rs_1_87199387	87199387	T	C	PC2	80.70888	Intron;RRP1B;
1	rs_1_87202680	87202680	A	G	PC2	76.56574	Intron;RRP1B;
1	rs_1_87203011	87203011	T	C	PC2	80.70888	Intron;RRP1B;
1	rs_1_87207951	87207951	C	A	PC2	80.70888	Intron;RRP1B;
1	rs_1_87209352	87209352	T	C	PC2	80.70888	Intron;RRP1B;
1	rs_1_87209543	87209543	G	T	PC2	80.70888	Intron;RRP1B;
1	rs_1_87218147	87218147	T	G	PC2	80.70888	Intron;HSF2BP;
1	rs_1_87226255	87226255	T	G	PC2	80.70888	Intron;HSF2BP;
1	rs_1_87227670	87227670	T	A	PC2	80.70888	Intron;HSF2BP;
1	rs_1_87238734	87238734	G	T	PC2	80.70888	Intron;HSF2BP;
1	rs_1_87242602	87242602	C	T	PC2	80.70888	Intron;HSF2BP;
1	rs_1_87249722	87249722	C	T	PC2	80.70888	HSF2BP;downstream;
1	rs_1_87279660	87279660	T	G	PC2	86.76771	Intergenic;
1	rs_1_87283911	87283911	A	G	PC2	80.70888	Intergenic;
1	rs_1_87286846	87286846	A	T	PC2	80.70888	Intergenic;
1	rs_1_87294047	87294047	C	T	PC2	80.70888	Intergenic;
1	rs_1_87296300	87296300	G	A	PC2	80.70888	Intergenic;
1	rs_1_87302319	87302319	A	T	PC2	80.70888	Intergenic;
1	rs_1_87329681	87329681	T	C	PC2	80.70888	Intron;SIK1;
1	rs_1_90775661	90775661	T	C	PC1	83.7223	Intergenic;
1	rs_1_93532189	93532189	A	G	PC1	77.99387	Intergenic;
1	rs_1_105124462	105124462	G	A	PC1	77.99387	Intron;POGLUT1;
1	rs_1_122740439	122740439	T	G	PC1	83.7223	Intergenic;
1	rs_1_122838054	122838054	C	T	PC1	77.99387	Intron;PRMT8;
1	rs_1_123361313	123361313	G	A	PC1	83.7223	Intron;VWF;
1	rs_1_123413506	123413506	C	T	PC1	83.7223	Intron;VWF;
1	rs_1_123438255	123438255	G	A	PC1	83.7223	Intron;VWF;
1	rs_1_123438303	123438303	C	T	PC1	83.7223	Intron;VWF;
1	rs_1_123738620	123738620	C	T	PC1	83.7223	Intergenic;
1	rs_1_123838566	123838566	G	A	PC1	83.7223	Intergenic;
1	rs_1_124031153	124031153	C	T	PC1	83.7223	Intergenic;
1	rs_1_124050562	124050562	T	C	PC1	83.7223	Intergenic;
1	rs_1_124430250	124430250	C	T	PC1	83.7223	Intron;CCND2;
1	rs_1_124670771	124670771	A	G	PC1	83.7223	Intron;FAR2;
1	rs_1_124674622	124674622	T	C	PC1	83.7223	Intron;FAR2;
1	rs_1_124872831	124872831	C	G	PC1	83.7223	Intergenic;
1	rs_1_124915682	124915682	G	C	PC1	83.7223	Intergenic;

1	rs_1_125693258	125693258	C	G	PC1	83.7223	Intergenic;
1	rs_1_126514930	126514930	G	T	PC1	83.7223	Intron;PARP12;
1	rs_1_126663012	126663012	T	C	PC1	83.7223	Intergenic;
1	rs_1_127092103	127092103	T	C	PC1	83.7223	WNT7B;upstream;
1	rs_1_127145837	127145837	C	T	PC1	83.7223	WNT7B;downstream;
1	rs_1_127156839	127156839	C	A	PC1	83.7223	Intergenic;
1	rs_1_127369394	127369394	G	A	PC1	83.7223	Intron;FBLN1;
1	rs_1_129134131	129134131	G	A	PC1	83.7223	Intron;MPPED1;
1	rs_1_129135168	129135168	G	A	PC1	83.7223	Intron;MPPED1;
1	rs_1_129135240	129135240	A	T	PC1	83.7223	Intron;MPPED1;
1	rs_1_129135576	129135576	A	T	PC1	83.7223	Intron;MPPED1;
1	rs_1_129136629	129136629	A	G	PC1	83.7223	Intron;MPPED1;
1	rs_1_129136747	129136747	C	T	PC1	83.7223	Intron;MPPED1;
1	rs_1_129138560	129138560	G	A	PC1	83.7223	Intron;MPPED1;
1	rs_1_131796190	131796190	A	G	PC1	77.99387	Intergenic;
1	rs_1_133834882	133834882	G	A	PC1	77.99387	Intergenic;
1	rs_1_134130710	134130710	C	G	PC1	77.99387	Intron;PIK3C2G;
1	rs_1_134130759	134130759	T	C	PC1	77.99387	Intron;PIK3C2G;
1	rs_1_134130817	134130817	G	A	PC1	77.99387	Intron;PIK3C2G;
1	rs_1_134130929	134130929	A	G	PC1	77.99387	Intron;PIK3C2G;
1	rs_1_134131521	134131521	C	T	PC1	77.99387	Intron;PIK3C2G;
1	rs_1_134131662	134131662	T	C	PC1	77.99387	Intron;PIK3C2G;
1	rs_1_134132000	134132000	C	T	PC1	77.99387	Intron;PIK3C2G;
1	rs_1_134132019	134132019	A	G	PC1	77.99387	Intron;PIK3C2G;
1	rs_1_134132055	134132055	A	G	PC1	77.99387	Intron;PIK3C2G;
1	rs_1_134132097	134132097	A	G	PC1	77.99387	Intron;PIK3C2G;
1	rs_1_134132198	134132198	G	T	PC1	77.99387	Intron;PIK3C2G;
1	rs_1_134132288	134132288	A	C	PC1	77.99387	Intron;PIK3C2G;
1	rs_1_142273865	142273865	G	C	PC1	77.99387	Intron;HIPK2;
1	rs_1_143075583	143075583	A	G	PC1	77.99387	Intergenic;
1	rs_1_143077217	143077217	G	C	PC1	77.99387	Intergenic;
1	rs_1_143077772	143077772	G	A	PC1	77.99387	Intergenic;
1	rs_1_143163390	143163390	T	C	PC1	77.99387	Intergenic;
1	rs_1_143168679	143168679	T	C	PC1	77.99387	Intergenic;
1	rs_1_143216266	143216266	T	C	PC1	77.99387	Intron;THL;
1	rs_1_143216397	143216397	T	C	PC1	77.99387	Intron;THL;
1	rs_1_145696489	145696489	G	A	PC1	77.99387	Intergenic;
1	rs_1_145741110	145741110	T	A	PC1	77.99387	Intergenic;
1	rs_1_145793312	145793312	A	G	PC1	77.99387	Intergenic;
1	rs_1_145871528	145871528	C	T	PC1	77.99387	Intergenic;
1	rs_1_147130368	147130368	T	C	PC1	77.99387	Intergenic;
1	rs_1_147176240	147176240	T	C	PC1	77.99387	Intron;CARD10;
1	rs_1_147650453	147650453	C	T	Others	84.01977	CBX6;upstream;CBX6;downstream;
1	rs_1_148811436	148811436	T	A	PC1	77.99387	PHF5A;downstream;
1	rs_1_154828167	154828167	G	A	PC1	77.99387	Intergenic;
1	rs_1_154836731	154836731	T	C	PC1	77.99387	Intergenic;
1	rs_1_155267281	155267281	T	A	PC1	77.99387	Intergenic;
1	rs_1_155267489	155267489	C	A	PC1	77.99387	Intergenic;
1	rs_1_155325400	155325400	A	G	Others	80.09584	Predicted;upstream;
1	rs_1_155383242	155383242	G	C	PC1	77.99387	Intergenic;
1	rs_1_155387930	155387930	T	C	PC1	77.99387	Intergenic;
1	rs_1_155389301	155389301	G	C	PC1	77.99387	Intergenic;
1	rs_1_155390934	155390934	T	G	PC1	77.99387	Intergenic;
1	rs_1_155391022	155391022	C	T	PC1	77.99387	Intergenic;
1	rs_1_155392645	155392645	T	C	PC1	77.99387	Intergenic;
1	rs_1_155511991	155511991	C	T	PC1	77.99387	Intron;KITLG;
1	rs_1_155524337	155524337	A	G	PC1	77.99387	Intron;KITLG;
1	rs_1_155529130	155529130	T	A	PC1	77.99387	Intron;KITLG;
1	rs_1_155558375	155558375	C	G	PC1	77.99387	Intergenic;
1	rs_1_155559099	155559099	A	C	PC1	77.99387	Intergenic;
1	rs_1_155559300	155559300	G	A	PC1	77.99387	Intergenic;
1	rs_1_155566518	155566518	C	T	PC1	77.99387	Intergenic;
1	rs_1_155567380	155567380	T	C	PC1	77.99387	Intergenic;
1	rs_1_155570325	155570325	C	T	PC1	77.99387	Intergenic;
1	rs_1_155577138	155577138	G	A	PC1	77.99387	Intergenic;
1	rs_1_155579096	155579096	C	T	PC1	77.99387	Intergenic;
1	rs_1_155580505	155580505	A	C	PC1	77.99387	Intergenic;
1	rs_1_155583565	155583565	T	C	PC1	77.99387	Intergenic;
1	rs_1_155586912	155586912	G	A	PC1	77.99387	Intergenic;
1	rs_1_155590232	155590232	C	T	PC1	77.99387	Intergenic;
1	rs_1_155594811	155594811	G	A	PC1	77.99387	Intergenic;
1	rs_1_155687856	155687856	A	C	PC1	77.99387	Intron;CEP290;
1	rs_1_155691232	155691232	G	A	PC1	77.99387	Intron;CEP290;
1	rs_1_155693423	155693423	A	G	PC1	77.99387	Intron;CEP290;
1	rs_1_155696670	155696670	T	C	PC1	77.99387	Intron;CEP290;
1	rs_1_156433622	156433622	C	T	PC1	77.99387	Intron;RASSF9;
1	rs_1_156445520	156445520	C	T	PC1	77.99387	Intergenic;
1	rs_1_156615558	156615558	T	C	PC1	77.99387	Intergenic;
1	rs_1_156647032	156647032	A	G	PC1	77.99387	Intergenic;
1	rs_1_156670205	156670205	T	C	PC1	77.99387	Intergenic;
1	rs_1_156682608	156682608	C	T	PC1	77.99387	ALX1;downstream;
1	rs_1_156689282	156689282	T	C	PC1	77.99387	Intron;ALX1;
1	rs_1_156794086	156794086	C	T	PC1	77.99387	Intron;LRRIQ1;
1	rs_1_156794452	156794452	A	C	PC1	77.99387	Intron;LRRIQ1;
1	rs_1_156796018	156796018	T	C	PC1	77.99387	Intron;LRRIQ1;
1	rs_1_156797687	156797687	G	A	PC1	77.99387	Intron;LRRIQ1;
1	rs_1_156799220	156799220	T	G	PC1	77.99387	Intron;LRRIQ1;
1	rs_1_156799613	156799613	A	G	PC1	77.99387	Intron;LRRIQ1;

1	rs_1_156799739	156799739	C	T	PC1	77.99387	Intron;LRRIQ1;
1	rs_1_15680378C	156803780	A	G	PC1	77.99387	Intron;LRRIQ1;
1	rs_1_15680652S	156806529	G	A	PC1	77.99387	Intron;LRRIQ1;
1	rs_1_156806741	156806741	C	T	PC1	77.99387	Intron;LRRIQ1;
1	rs_1_156898184	156898184	A	G	PC1	77.99387	Intergenic;
1	rs_1_156901633	156901633	T	G	PC1	77.99387	Intergenic;
1	rs_1_157544714	157544714	G	A	PC1	77.99387	Intergenic;
1	rs_1_157648002	157648002	T	C	Others	109.9409	Intron;SKI3;
1	rs_1_157681688	157681688	A	G	PC1	77.99387	Intron;SKI3;
1	rs_1_161369768	161369768	A	T	PC1	77.99387	Intergenic;
1	rs_1_161371605	161371605	C	T	PC1	77.99387	Intergenic;
1	rs_1_16137287€	161372876	T	C	PC1	77.99387	Intergenic;
1	rs_1_161817843	161817843	C	T	PC1	77.99387	Intergenic;
1	rs_1_16270145€	162701456	C	T	PC1	77.99387	Intron;PTPRR;
1	rs_1_162750125	162750125	C	T	PC1	77.99387	Intron;PTPRR;
1	rs_1_166553023	166553023	T	G	PC1	77.99387	Intergenic;
1	rs_1_166553573	166553573	C	T	PC1	77.99387	Intergenic;
1	rs_1_167539154	167539154	A	G	PC1	77.99387	Intergenic;
1	rs_1_167539164	167539164	C	G	PC1	77.99387	Intergenic;
1	rs_1_172278948	172278948	C	T	PC1	77.99387	Intron;NAMPT;
1	rs_1_172709595	172709595	G	C	PC1	77.99387	Intron;COG5;
1	rs_1_172711007	172711007	A	G	PC1	77.99387	Intron;COG5;
1	rs_1_174331077	174331077	G	T	PC1	77.99387	Intron;TBC1D22A;
1	rs_1_175762613	175762613	G	C	PC1	77.99387	Intergenic;
1	rs_1_175764423	175764423	A	T	PC1	77.99387	Intergenic;
1	rs_1_17577279€	175772796	A	G	PC1	77.99387	Intergenic;
1	rs_1_17648882€	176488826	T	C	PC1	77.99387	Intergenic;
1	rs_1_176497138	176497138	A	G	PC1	77.99387	Intergenic;
1	rs_1_176497903	176497903	T	G	PC1	77.99387	Intergenic;
1	rs_1_176498371	176498371	C	T	PC1	77.99387	Intergenic;
1	rs_1_176498827	176498827	C	T	PC1	77.99387	Intergenic;
1	rs_1_176500137	176500137	A	G	PC1	77.99387	Intergenic;
1	rs_1_176561798	176561798	G	A	PC1	77.99387	Intergenic;
1	rs_1_176583108	176583108	C	T	PC1	77.99387	Intergenic;
1	rs_1_176591383	176591383	C	G	PC1	77.99387	Intergenic;
1	rs_1_17659224€	176592246	A	G	PC1	77.99387	Intergenic;
1	rs_1_17659785C	176597850	A	C	PC1	77.99387	Intergenic;
1	rs_1_176598067	176598067	C	T	PC1	77.99387	Intergenic;
1	rs_1_176601141	176601141	C	T	PC1	77.99387	Intergenic;
1	rs_1_176605253	176605253	A	G	PC1	77.99387	Intergenic;
1	rs_1_176610674	176610674	A	G	PC1	77.99387	Intergenic;
1	rs_1_176611562	176611562	G	A	PC1	77.99387	Intergenic;
1	rs_1_176669634	176669634	T	C	PC1	77.99387	Intergenic;
1	rs_1_176673202	176673202	A	G	PC1	77.99387	Intergenic;
1	rs_1_176917022	176917022	T	C	PC1	77.99387	Intergenic;
1	rs_1_176920098	176920098	T	C	PC1	77.99387	BRD1;downstream;
1	rs_1_176981638	176981638	A	G	PC1	77.99387	BRD1;upstream;
1	rs_1_176994887	176994887	A	G	PC1	77.99387	Intergenic;
1	rs_1_176995638	176995638	T	C	PC1	77.99387	Intergenic;
1	rs_1_17700315€	177003156	G	A	PC1	77.99387	Intergenic;
1	rs_1_17700337S	177003379	G	A	PC1	77.99387	Intergenic;
1	rs_1_177003437	177003437	C	T	PC1	77.99387	Intergenic;
1	rs_1_177010771	177010771	G	A	PC1	77.99387	Intergenic;
1	rs_1_17701264€	177012646	A	G	PC1	77.99387	Intergenic;
1	rs_1_177012845	177012845	A	G	PC1	77.99387	Intergenic;
1	rs_1_17705238C	177052380	T	C	PC1	77.99387	CRELD2-A;downstream;
1	rs_1_177053453	177053453	G	T	PC1	77.99387	CRELD2-A;downstream;
1	rs_1_177119777	177119777	A	C	PC1	77.99387	Intergenic;
1	rs_1_177120177	177120177	A	C	PC1	77.99387	Intergenic;
1	rs_1_177166118	177166118	T	C	PC1	77.99387	Intergenic;
1	rs_1_17716714€	177167146	A	T	PC1	77.99387	Intergenic;
1	rs_1_177174071	177174071	T	C	PC1	77.99387	Intergenic;
1	rs_1_17718499S	177184999	C	A	PC1	77.99387	Intergenic;
1	rs_1_177195261	177195261	G	A	PC1	77.99387	Intergenic;
1	rs_1_177195942	177195942	G	A	PC1	77.99387	Intergenic;
1	rs_1_17721979€	177219796	T	C	PC1	77.99387	Intergenic;
1	rs_1_177219803	177219803	A	G	PC1	77.99387	Intergenic;
1	rs_1_177221315	177221315	G	A	PC1	77.99387	Intergenic;
1	rs_1_177227123	177227123	A	C	PC1	77.99387	Intergenic;
1	rs_1_177229418	177229418	T	C	PC1	77.99387	Intergenic;
1	rs_1_177230758	177230758	C	T	PC1	77.99387	Intergenic;
1	rs_1_177231602	177231602	T	C	PC1	77.99387	Intergenic;
1	rs_1_177234421	177234421	G	C	PC1	77.99387	Intergenic;
1	rs_1_177234843	177234843	C	A	PC1	77.99387	Intergenic;
1	rs_1_177234872	177234872	A	C	PC1	77.99387	Intergenic;
1	rs_1_177236104	177236104	C	T	PC1	77.99387	Intergenic;
1	rs_1_177239777	177239777	T	C	PC1	77.99387	Intergenic;
1	rs_1_177243103	177243103	T	C	PC1	77.99387	Intergenic;
1	rs_1_177244127	177244127	A	T	PC1	77.99387	Intergenic;
1	rs_1_177245351	177245351	A	G	PC1	77.99387	Intergenic;
1	rs_1_17724630C	177246300	T	A	PC1	77.99387	Intergenic;
1	rs_1_177246878	177246878	G	C	PC1	77.99387	Intergenic;
1	rs_1_177247602	177247602	A	C	PC1	77.99387	Intergenic;
1	rs_1_177248672	177248672	A	G	PC1	77.99387	Intergenic;
1	rs_1_17724940S	177249409	A	G	PC1	77.99387	Intergenic;
1	rs_1_177249683	177249683	C	T	PC1	77.99387	Intergenic;
1	rs_1_177250358	177250358	A	G	PC1	77.99387	Intergenic;
1	rs_1_177250695	177250695	A	G	PC1	77.99387	Intergenic;

1	rs_1_177252127	177252127	T	A	PC1	77.99387	Intergenic;
1	rs_1_177252214	177252214	A	G	PC1	77.99387	Intergenic;
1	rs_1_177252357	177252357	C	T	PC1	77.99387	Intergenic;
1	rs_1_177253080	177253080	T	C	PC1	77.99387	Intergenic;
1	rs_1_177255144	177255144	T	G	PC1	77.99387	Intergenic;
1	rs_1_177256120	177256120	G	A	PC1	77.99387	Intergenic;
1	rs_1_177256199	177256199	T	G	PC1	77.99387	Intergenic;
1	rs_1_177261414	177261414	G	C	PC1	77.99387	Intergenic;
1	rs_1_177265258	177265258	C	T	PC1	77.99387	Intergenic;
1	rs_1_177268516	177268516	A	T	PC1	77.99387	Intergenic;
1	rs_1_177275525	177275525	T	C	PC1	77.99387	Intergenic;
1	rs_1_177275638	177275638	C	T	PC1	77.99387	Intergenic;
1	rs_1_177279197	177279197	T	C	PC1	77.99387	Intergenic;
1	rs_1_177280761	177280761	A	G	PC1	77.99387	Intergenic;
1	rs_1_177281472	177281472	T	C	PC1	77.99387	Intergenic;
1	rs_1_177282394	177282394	A	G	PC1	77.99387	Intergenic;
1	rs_1_177283214	177283214	G	A	PC1	77.99387	Intergenic;
1	rs_1_177284153	177284153	G	A	PC1	77.99387	Intergenic;
1	rs_1_177285107	177285107	C	T	PC1	77.99387	Intergenic;
1	rs_1_177286896	177286896	A	T	PC1	77.99387	Intergenic;
1	rs_1_177287158	177287158	A	G	PC1	77.99387	Intergenic;
1	rs_1_177287726	177287726	A	G	PC1	77.99387	Intergenic;
1	rs_1_177287865	177287865	C	G	PC1	77.99387	Intergenic;
1	rs_1_177288744	177288744	C	T	PC1	77.99387	Intergenic;
1	rs_1_177294729	177294729	T	G	PC1	77.99387	Intergenic;
1	rs_1_177297779	177297779	T	C	PC1	77.99387	Intergenic;
1	rs_1_177313789	177313789	G	A	PC1	77.99387	Intergenic;
1	rs_1_177313912	177313912	A	G	PC1	77.99387	Intergenic;
1	rs_1_177321804	177321804	A	G	PC1	77.99387	Intergenic;
1	rs_1_177323333	177323333	T	C	PC1	77.99387	Intergenic;
1	rs_1_177327502	177327502	C	T	PC1	77.99387	Intergenic;
1	rs_1_177328252	177328252	G	C	PC1	77.99387	Intergenic;
1	rs_1_177328667	177328667	A	T	PC1	77.99387	Intergenic;
1	rs_1_177332878	177332878	T	G	PC1	77.99387	Intergenic;
1	rs_1_177332972	177332972	A	C	PC1	77.99387	Intergenic;
1	rs_1_177339199	177339199	C	G	PC1	77.99387	Intergenic;
1	rs_1_177345809	177345809	T	G	PC1	77.99387	PIM3;upstream;
1	rs_1_177349700	177349700	G	A	PC1	77.99387	Intergenic;
1	rs_1_177349982	177349982	T	C	PC1	77.99387	Intergenic;
1	rs_1_177353766	177353766	C	A	PC1	77.99387	Intergenic;
1	rs_1_177353768	177353768	A	G	PC1	77.99387	Intergenic;
1	rs_1_177357752	177357752	G	A	PC1	77.99387	Intergenic;
1	rs_1_177361597	177361597	T	C	PC1	77.99387	Intergenic;
1	rs_1_177364586	177364586	T	C	PC1	77.99387	Intergenic;
1	rs_1_177364631	177364631	C	T	PC1	77.99387	Intergenic;
1	rs_1_177366602	177366602	T	C	PC1	77.99387	Intergenic;
1	rs_1_177368882	177368882	T	G	PC1	77.99387	Intergenic;
1	rs_1_177369119	177369119	G	A	PC1	77.99387	Intergenic;
1	rs_1_177370555	177370555	T	C	PC1	77.99387	Intergenic;
1	rs_1_177370584	177370584	C	T	PC1	77.99387	Intergenic;
1	rs_1_177377603	177377603	T	C	PC1	77.99387	Intergenic;
1	rs_1_177377833	177377833	T	C	PC1	77.99387	Intergenic;
1	rs_1_177378247	177378247	G	C	PC1	77.99387	Intergenic;
1	rs_1_177384703	177384703	A	T	PC1	77.99387	Intergenic;
1	rs_1_177385926	177385926	A	G	PC1	77.99387	Intergenic;
1	rs_1_177386767	177386767	A	G	PC1	77.99387	Intergenic;
1	rs_1_177387920	177387920	G	A	PC1	77.99387	Intergenic;
1	rs_1_177402671	177402671	T	C	PC1	77.99387	Intergenic;
1	rs_1_177406035	177406035	A	G	PC1	77.99387	Intergenic;
1	rs_1_177408739	177408739	A	C	PC1	77.99387	Intergenic;
1	rs_1_177414181	177414181	C	G	PC1	77.99387	Intergenic;
1	rs_1_177418636	177418636	G	A	PC1	77.99387	Intergenic;
1	rs_1_177422284	177422284	C	G	PC1	77.99387	Intergenic;
1	rs_1_177422903	177422903	C	A	PC1	77.99387	Intergenic;
1	rs_1_177423282	177423282	G	A	PC1	77.99387	Intergenic;
1	rs_1_177430679	177430679	A	G	PC1	77.99387	Intergenic;
1	rs_1_177462194	177462194	A	G	PC1	77.99387	Intergenic;
1	rs_1_177466061	177466061	T	C	PC1	77.99387	Intergenic;
1	rs_1_177469506	177469506	G	A	PC1	77.99387	Intergenic;
1	rs_1_177937495	177937495	T	G	PC1	77.99387	Intergenic;
1	rs_1_177938995	177938995	T	C	PC1	77.99387	Intergenic;
1	rs_1_177939883	177939883	C	T	PC1	77.99387	Intergenic;
1	rs_1_178021688	178021688	T	C	PC1	77.99387	Intergenic;
1	rs_1_178030934	178030934	G	A	PC1	77.99387	Intergenic;
1	rs_1_178031226	178031226	G	A	PC1	77.99387	Intergenic;
1	rs_1_178036457	178036457	T	C	PC1	77.99387	Intergenic;
1	rs_1_178036810	178036810	A	C	PC1	77.99387	Intergenic;
1	rs_1_178040397	178040397	G	A	PC1	77.99387	Intergenic;
1	rs_1_178043997	178043997	T	A	PC1	77.99387	Intergenic;
1	rs_1_178044844	178044844	T	C	PC1	77.99387	Intergenic;
1	rs_1_178045998	178045998	A	G	PC1	77.99387	Intergenic;
1	rs_1_178048487	178048487	T	C	PC1	77.99387	Intergenic;
1	rs_1_178050237	178050237	T	C	PC1	77.99387	Intergenic;
1	rs_1_178055759	178055759	A	C	PC1	77.99387	Intergenic;
1	rs_1_178072431	178072431	A	G	PC1	77.99387	Intergenic;
1	rs_1_178592488	178592488	T	C	PC1	77.99387	Intron;TUBGCP6;
1	rs_1_178597696	178597696	T	G	PC2	103.2241	TUBGCP6;upstream;HDAC10;downstream;
1	rs_1_180939691	180939691	A	G	PC1	77.99387	Intron;CADPS2;

1	rs_1_18093976	180939766	T	G	PC1	77.99387	Intron;CADPS2;
1	rs_1_181399807	181399807	A	G	PC1	77.99387	Intergenic;
1	rs_1_183257843	183257843	A	T	PC1	77.99387	Intergenic;
1	rs_1_18498309	184983099	T	G	PC1	77.99387	Intergenic;
1	rs_1_185331593	185331593	C	T	PC1	77.99387	BMT2;downstream;TMEM168;upstream;
1	rs_1_187155445	187155445	A	C	PC1	77.99387	Intron;MAGI2;
1	rs_1_187158391	187158391	A	G	PC1	77.99387	Intron;MAGI2;
1	rs_1_187158647	187158647	C	G	PC1	77.99387	Intron;MAGI2;
1	rs_1_18889326	188893266	C	T	PC1	77.99387	Intron;HGF;
1	rs_1_189587077	189587077	G	A	PC1	77.99387	Intron;PCLO;
1	rs_1_19029309	190293096	C	T	PC1	77.99387	Intron;SEMA3A;
1	rs_1_190294345	190294345	C	T	PC1	77.99387	Intron;SEMA3A;
1	rs_1_19030546	190305466	T	C	PC1	77.99387	Intron;SEMA3A;
1	rs_1_19692094	196920946	C	A	PC2	97.79576	Intron;PLXNA4;
1	rs_1_19692480	196924809	T	C	PC2	97.79576	Intron;PLXNA4;
1	rs_1_19693065	196930656	T	A	PC2	97.79576	Intron;PLXNA4;
1	rs_1_196931547	196931547	C	T	PC2	82.21736	Intron;PLXNA4;
1	rs_1_196936431	196936431	T	C	PC2	97.79576	Intron;PLXNA4;
1	rs_1_196941603	196941603	T	A	PC2	82.21736	Intron;PLXNA4;
1	rs_1_196942962	196942962	G	A	PC2	97.79576	Intron;PLXNA4;
1	rs_1_196943393	196943393	T	A	PC2	97.79576	Intron;PLXNA4;
1	rs_1_196945501	196945501	G	C	PC2	83.0765	Intron;PLXNA4;
1	rs_1_196949711	196949711	G	C	PC2	83.02867	Intron;PLXNA4;
1	rs_1_19695197	196951979	T	C	PC2	83.71623	Intron;PLXNA4;
2	rs_2_2567848	2567848	T	C	PC1	77.99387	Exon;OBSCN;OBSCN;-;2;GCA;Synonymous;Ala;Ala;
2	rs_2_4856570	4856570	A	G	PC1	77.99387	Intron;Predicted;
2	rs_2_6959704	6959704	A	G	PC1	83.7223	Intron;PRKAG2;
2	rs_2_7777177	7777177	T	C	PC1	83.7223	Intergenic;
2	rs_2_7777278	7777278	T	C	PC1	83.7223	Intergenic;
2	rs_2_8434971	8434971	G	A	PC1	83.7223	EN2;upstream;
2	rs_2_12536081	12536081	T	C	PC1	83.7223	Intergenic;
2	rs_2_12539747	12539747	G	A	PC1	83.7223	Intergenic;
2	rs_2_12540394	12540394	C	G	PC1	83.7223	Intergenic;
2	rs_2_12540967	12540967	T	G	PC1	83.7223	Intergenic;
2	rs_2_12541463	12541463	C	T	PC1	83.7223	Intergenic;
2	rs_2_12541621	12541621	T	C	PC1	83.7223	Intergenic;
2	rs_2_12542418	12542418	C	T	PC1	83.7223	Intergenic;
2	rs_2_12542714	12542714	G	T	PC1	83.7223	Intergenic;
2	rs_2_12542752	12542752	T	C	PC1	83.7223	Intergenic;
2	rs_2_12544650	12544650	G	A	PC1	83.7223	Intergenic;
2	rs_2_12545366	12545366	C	T	PC1	83.7223	Intergenic;
2	rs_2_12545665	12545665	T	C	PC1	83.7223	Intergenic;
2	rs_2_12547604	12547604	T	C	PC1	83.7223	Intergenic;
2	rs_2_12734142	12734142	G	A	PC1	83.7223	Intergenic;
2	rs_2_12734285	12734285	A	G	PC1	83.7223	Intergenic;
2	rs_2_12744530	12744530	A	C	PC1	83.7223	Intergenic;
2	rs_2_12744857	12744857	A	G	PC1	83.7223	Intergenic;
2	rs_2_12744930	12744930	T	C	PC1	83.7223	Intergenic;
2	rs_2_12748455	12748455	A	G	PC1	83.7223	Intergenic;
2	rs_2_12748719	12748719	G	A	PC1	83.7223	Intergenic;
2	rs_2_12753472	12753472	C	T	PC1	83.7223	Intergenic;
2	rs_2_12754647	12754647	C	T	PC1	83.7223	Intergenic;
2	rs_2_12756291	12756291	T	C	PC1	83.7223	Intergenic;
2	rs_2_12765791	12765791	G	A	PC1	83.7223	Intergenic;
2	rs_2_12771374	12771374	G	C	PC1	83.7223	Intergenic;
2	rs_2_12777974	12777974	G	A	PC1	83.7223	Intergenic;
2	rs_2_12778773	12778773	C	T	PC1	83.7223	Intergenic;
2	rs_2_12784135	12784135	T	C	PC1	83.7223	Intergenic;
2	rs_2_12784248	12784248	A	G	PC1	83.7223	Intergenic;
2	rs_2_12784535	12784535	G	A	PC1	77.99387	Intergenic;
2	rs_2_12788037	12788037	A	G	PC1	83.7223	Intergenic;
2	rs_2_12792790	12792790	A	T	PC1	83.7223	Intergenic;
2	rs_2_12793553	12793553	C	T	PC1	83.7223	Intergenic;
2	rs_2_12793573	12793573	A	G	PC1	83.7223	Intergenic;
2	rs_2_12826043	12826043	A	T	PC1	83.7223	Intergenic;
2	rs_2_12826104	12826104	A	T	PC1	83.7223	Intergenic;
2	rs_2_12911446	12911446	A	T	PC1	83.7223	Intergenic;
2	rs_2_14065882	14065882	C	T	PC1	77.99387	Intron;PARD3;
2	rs_2_14668604	14668604	C	T	PC1	83.7223	Predicted;upstream;
2	rs_2_14676514	14676514	G	A	PC1	83.7223	Intergenic;
2	rs_2_39846331	39846331	A	G	PC1	83.7223	Intergenic;
2	rs_2_40819144	40819144	C	G	PC1	83.7223	Intron;RBMS3;
2	rs_2_45247687	45247687	A	G	PC1	77.99387	Predicted;upstream;
2	rs_2_46673567	46673567	G	A	PC1	83.7223	Intergenic;
2	rs_2_46674585	46674585	C	T	PC1	83.7223	Intergenic;
2	rs_2_46706710	46706710	T	C	PC1	83.7223	Intergenic;
2	rs_2_46707563	46707563	A	G	PC1	83.7223	Intergenic;
2	rs_2_46707620	46707620	G	C	PC1	77.99387	Intergenic;
2	rs_2_46708995	46708995	A	G	PC1	83.7223	Intergenic;
2	rs_2_46709709	46709709	T	G	PC1	83.7223	Intergenic;
2	rs_2_46710239	46710239	T	A	PC1	83.7223	Intergenic;
2	rs_2_46712424	46712424	G	A	PC1	83.7223	Intergenic;
2	rs_2_46713426	46713426	A	T	PC1	83.7223	Intergenic;
2	rs_2_46728715	46728715	C	T	PC1	83.7223	Intergenic;
2	rs_2_46738877	46738877	T	C	PC1	83.7223	Intergenic;
2	rs_2_46740083	46740083	C	A	PC1	83.7223	Intergenic;
2	rs_2_46743833	46743833	C	T	PC1	83.7223	Intergenic;
2	rs_2_47502276	47502276	C	T	PC1	83.7223	Intergenic;

2	rs_2_47503131	47503131	T	C	PC1	83.7223	Intergenic;
2	rs_2_47503543	47503543	C	T	PC1	83.7223	Intergenic;
2	rs_2_47505737	47505737	T	C	PC1	83.7223	Intergenic;
2	rs_2_47508370	47508370	C	T	PC1	83.7223	Intergenic;
2	rs_2_47510064	47510064	T	C	PC1	83.7223	Intergenic;
2	rs_2_47511570	47511570	T	C	PC1	83.7223	Intergenic;
2	rs_2_47515246	47515246	C	T	PC1	83.7223	Intergenic;
2	rs_2_47917777	47917777	C	T	PC1	83.7223	Intron;ELMO1;
2	rs_2_47926276	47926276	T	G	PC1	83.7223	Intron;ELMO1;
2	rs_2_47928692	47928692	A	T	PC1	83.7223	Intron;ELMO1;
2	rs_2_47937681	47937681	T	G	PC1	83.7223	Intron;ELMO1;
2	rs_2_47942027	47942027	G	C	PC1	83.7223	Intron;ELMO1;
2	rs_2_47944196	47944196	C	A	PC1	83.7223	Intron;ELMO1;
2	rs_2_47984060	47984060	A	C	PC1	83.7223	Intron;ELMO1;
2	rs_2_48009178	48009178	G	A	PC1	83.7223	Intron;ELMO1;
2	rs_2_48061161	48061161	G	A	PC1	83.7223	ELMO1;downstream;AOAH;upstream;
2	rs_2_48063158	48063158	T	C	PC1	83.7223	Predicted;downstream;AOAH;upstream;
2	rs_2_49692953	49692953	A	G	PC1	83.7223	Intron;PDE1C;
2	rs_2_49815756	49815756	A	G	PC1	83.7223	Intron;PDE1C;
2	rs_2_49827645	49827645	A	G	PC1	83.7223	Intron;PDE1C;
2	rs_2_49830991	49830991	T	C	PC1	83.7223	Intron;PDE1C;
2	rs_2_49834620	49834620	C	T	PC1	83.7223	Intron;PDE1C;
2	rs_2_49835589	49835589	C	G	PC1	83.7223	Intron;PDE1C;
2	rs_2_49838465	49838465	A	G	PC1	83.7223	Intron;PDE1C;
2	rs_2_49841013	49841013	T	C	PC1	83.7223	Intron;PDE1C;
2	rs_2_49841452	49841452	C	T	PC1	83.7223	Intron;PDE1C;
2	rs_2_49842297	49842297	G	C	PC1	83.7223	Intron;PDE1C;
2	rs_2_49843423	49843423	G	A	PC1	83.7223	Intron;PDE1C;
2	rs_2_49846285	49846285	A	G	PC1	83.7223	Intron;PDE1C;
2	rs_2_49846794	49846794	G	A	PC1	83.7223	Intron;PDE1C;
2	rs_2_49848816	49848816	A	G	PC1	83.7223	Intron;PDE1C;
2	rs_2_60540124	60540124	A	G	PC1	83.7223	Intergenic;
2	rs_2_60727110	60727110	T	C	PC1	83.7223	Intron;CDKAL1;
2	rs_2_61093913	61093913	T	G	PC1	83.7223	Intron;E2F3;
2	rs_2_61094291	61094291	A	G	PC1	83.7223	Intron;E2F3;
2	rs_2_61094414	61094414	G	A	PC1	83.7223	Intron;E2F3;
2	rs_2_61095128	61095128	C	T	PC1	83.7223	Intron;E2F3;
2	rs_2_61096250	61096250	G	A	PC1	83.7223	Intron;E2F3;
2	rs_2_61097266	61097266	C	T	PC1	83.7223	Intron;E2F3;
2	rs_2_64032665	64032665	A	G	PC1	83.7223	Intron;PHACTR3;
2	rs_2_64033178	64033178	C	T	PC1	83.7223	Intron;PHACTR3;
2	rs_2_68162094	68162094	A	G	PC1	83.7223	IRF4;upstream;
2	rs_2_68199248	68199248	A	G	PC1	77.99387	EXOC2;downstream;
2	rs_2_68200548	68200548	T	C	PC1	77.99387	EXOC2;downstream;
2	rs_2_68200816	68200816	T	C	PC1	77.99387	EXOC2;downstream;
2	rs_2_68201426	68201426	T	C	PC1	77.99387	EXOC2;downstream;
2	rs_2_68396437	68396437	T	C	PC1	83.7223	Intergenic;
2	rs_2_68396489	68396489	C	T	PC1	83.7223	Intergenic;
2	rs_2_68397993	68397993	T	C	PC1	83.7223	Intergenic;
2	rs_2_68410013	68410013	T	C	PC1	83.7223	Intergenic;
2	rs_2_68451504	68451504	C	T	PC1	83.7223	Intergenic;
2	rs_2_68453212	68453212	G	C	PC1	83.7223	Intergenic;
2	rs_2_68453853	68453853	C	A	PC1	77.99387	Intergenic;
2	rs_2_68457484	68457484	T	C	PC1	83.7223	Intergenic;
2	rs_2_68470979	68470979	G	A	PC1	83.7223	Intergenic;
2	rs_2_68473351	68473351	A	T	PC1	83.7223	Intergenic;
2	rs_2_68510920	68510920	T	C	PC1	83.7223	Intergenic;
2	rs_2_69189458	69189458	A	T	PC1	83.7223	Intergenic;
2	rs_2_69189714	69189714	A	C	PC1	83.7223	Intergenic;
2	rs_2_69191815	69191815	C	T	PC1	83.7223	Intergenic;
2	rs_2_70211833	70211833	A	G	PC1	83.7223	Intergenic;
2	rs_2_70515725	70515725	T	C	PC1	83.7223	Intergenic;
2	rs_2_70527044	70527044	C	G	PC1	83.7223	Intergenic;
2	rs_2_70555885	70555885	G	A	PC1	83.7223	Intergenic;
2	rs_2_70559958	70559958	T	A	PC1	83.7223	Intergenic;
2	rs_2_70573524	70573524	A	T	PC1	83.7223	Intergenic;
2	rs_2_70573525	70573525	T	C	PC1	83.7223	Intergenic;
2	rs_2_70780248	70780248	C	T	PC1	83.7223	Intergenic;
2	rs_2_76236511	76236511	A	G	PC1	83.7223	Intergenic;
2	rs_2_76240824	76240824	G	A	PC1	83.7223	Intergenic;
2	rs_2_76242327	76242327	T	C	PC1	83.7223	Intergenic;
2	rs_2_76244013	76244013	A	G	PC1	83.7223	Intergenic;
2	rs_2_76248918	76248918	G	A	PC1	83.7223	RBBP6;upstream;
2	rs_2_76250082	76250082	C	T	PC1	83.7223	Intergenic;
2	rs_2_76258501	76258501	T	A	PC1	83.7223	Intergenic;
2	rs_2_76260035	76260035	A	G	PC1	83.7223	Intergenic;
2	rs_2_76267594	76267594	C	G	PC1	83.7223	Intergenic;
2	rs_2_76268386	76268386	T	A	PC1	83.7223	Intergenic;
2	rs_2_76271073	76271073	G	A	PC1	83.7223	Intergenic;
2	rs_2_76271329	76271329	A	G	PC1	83.7223	Intergenic;
2	rs_2_76272893	76272893	T	C	PC1	83.7223	Intergenic;
2	rs_2_76306337	76306337	T	G	PC1	77.99387	Intron;CDH18;
2	rs_2_76308341	76308341	C	T	PC1	83.7223	Intron;CDH18;
2	rs_2_76312616	76312616	C	A	PC1	83.7223	Intron;CDH18;
2	rs_2_76312685	76312685	T	C	PC1	83.7223	Intron;CDH18;
2	rs_2_76316372	76316372	G	A	PC1	83.7223	Intron;CDH18;
2	rs_2_76340997	76340997	A	T	PC1	83.7223	Intron;CDH18;
2	rs_2_76341082	76341082	A	T	PC1	83.7223	Intron;CDH18;

2	rs_2_76341360	76341360	G	C	PC1	83.7223	Intron;CDH18;
2	rs_2_76342924	76342924	T	C	PC1	83.7223	Intron;CDH18;
2	rs_2_76343072	76343072	T	C	PC1	83.7223	Intron;CDH18;
2	rs_2_76343076	76343076	C	T	PC1	83.7223	Intron;CDH18;
2	rs_2_76343147	76343147	A	G	PC1	83.7223	Intron;CDH18;
2	rs_2_76420187	76420187	G	C	PC1	83.7223	Intron;CDH18;
2	rs_2_76420343	76420343	T	C	PC1	83.7223	Intron;CDH18;
2	rs_2_76420344	76420344	G	A	PC1	83.7223	Intron;CDH18;
2	rs_2_76423257	76423257	C	A	PC1	83.7223	Exon;CDH18;CDH18;+;0;CTC;Nonsynonymous;Leu;Ile;
2	rs_2_76424429	76424429	T	C	PC1	83.7223	Intron;CDH18;
2	rs_2_76425567	76425567	C	T	PC1	83.7223	Intron;CDH18;
2	rs_2_76513453	76513453	A	C	PC1	83.7223	Intergenic;
2	rs_2_76516120	76516120	A	G	PC1	83.7223	Intergenic;
2	rs_2_76518876	76518876	C	T	PC1	83.7223	Intergenic;
2	rs_2_76535884	76535884	G	A	PC1	77.99387	Intergenic;
2	rs_2_76537491	76537491	C	G	PC1	77.99387	Intergenic;
2	rs_2_77202169	77202169	A	G	PC1	83.7223	Intergenic;
2	rs_2_77204493	77204493	A	G	PC1	83.7223	Intergenic;
2	rs_2_77210494	77210494	T	C	PC1	77.99387	Intergenic;
2	rs_2_77212640	77212640	A	G	PC1	83.7223	Intergenic;
2	rs_2_77223942	77223942	C	T	PC1	83.7223	Intergenic;
2	rs_2_77223957	77223957	C	T	PC1	83.7223	Intergenic;
2	rs_2_77223990	77223990	G	A	PC1	83.7223	Intergenic;
2	rs_2_77224530	77224530	A	C	PC1	83.7223	Intergenic;
2	rs_2_77224925	77224925	C	T	PC1	83.7223	Intergenic;
2	rs_2_77225417	77225417	A	G	PC1	83.7223	Intergenic;
2	rs_2_77225511	77225511	A	C	PC1	83.7223	Intergenic;
2	rs_2_77226325	77226325	T	C	PC1	83.7223	Intergenic;
2	rs_2_77226988	77226988	T	C	PC1	83.7223	Intergenic;
2	rs_2_77227080	77227080	G	A	PC1	83.7223	Intergenic;
2	rs_2_77227260	77227260	G	A	PC1	83.7223	Intergenic;
2	rs_2_77227354	77227354	T	C	PC1	83.7223	Intergenic;
2	rs_2_77228404	77228404	T	C	PC1	83.7223	Intergenic;
2	rs_2_77228920	77228920	T	G	PC1	83.7223	Intergenic;
2	rs_2_77229904	77229904	C	T	PC1	83.7223	Intergenic;
2	rs_2_77230199	77230199	T	C	PC1	83.7223	Intergenic;
2	rs_2_77230582	77230582	C	G	PC1	83.7223	Intergenic;
2	rs_2_77230933	77230933	A	G	PC1	83.7223	Intergenic;
2	rs_2_77231199	77231199	T	C	PC1	83.7223	Intergenic;
2	rs_2_77787404	77787404	T	C	PC1	83.7223	Intergenic;
2	rs_2_77787636	77787636	T	C	PC1	83.7223	Intergenic;
2	rs_2_77787806	77787806	A	G	PC1	83.7223	Intergenic;
2	rs_2_79164333	79164333	G	T	PC1	77.99387	Intergenic;
2	rs_2_79165303	79165303	C	A	PC1	83.7223	Intergenic;
2	rs_2_79167597	79167597	G	A	PC1	83.7223	Intergenic;
2	rs_2_79175048	79175048	A	G	PC1	83.7223	Intergenic;
2	rs_2_79183255	79183255	C	T	PC1	83.7223	Intergenic;
2	rs_2_79183323	79183323	G	C	PC1	83.7223	Intergenic;
2	rs_2_79185256	79185256	G	A	PC1	83.7223	Intergenic;
2	rs_2_79188285	79188285	C	T	PC1	83.7223	Intergenic;
2	rs_2_79207054	79207054	T	G	PC1	83.7223	Intergenic;
2	rs_2_79209117	79209117	G	A	PC1	83.7223	Intergenic;
2	rs_2_79211780	79211780	C	T	PC1	83.7223	Intergenic;
2	rs_2_79306311	79306311	G	A	PC1	83.7223	Intergenic;
2	rs_2_79316087	79316087	C	T	PC1	83.7223	Intergenic;
2	rs_2_79383700	79383700	A	G	PC1	83.7223	Intergenic;
2	rs_2_81803785	81803785	C	T	PC1	83.7223	Intergenic;
2	rs_2_81809223	81809223	G	A	PC1	83.7223	Intergenic;
2	rs_2_81812041	81812041	T	A	PC1	83.7223	Intergenic;
2	rs_2_81889417	81889417	A	G	PC1	83.7223	Intergenic;
2	rs_2_82928325	82928325	G	A	PC1	77.99387	Intron;IKZF1;
2	rs_2_83177256	83177256	T	C	PC1	77.99387	Intron;GRB10;
2	rs_2_83178701	83178701	G	T	PC1	77.99387	Intron;GRB10;
2	rs_2_83178967	83178967	T	C	PC1	77.99387	Intron;GRB10;
2	rs_2_83179116	83179116	T	C	PC1	77.99387	Intron;GRB10;
2	rs_2_83180312	83180312	G	A	PC1	77.99387	Intron;GRB10;
2	rs_2_83705354	83705354	C	T	PC1	77.99387	Intergenic;
2	rs_2_83705696	83705696	A	G	PC1	77.99387	Intergenic;
2	rs_2_83709816	83709816	T	C	PC1	77.99387	Intergenic;
2	rs_2_83733098	83733098	T	C	PC1	77.99387	Intergenic;
2	rs_2_83747492	83747492	T	C	PC1	77.99387	Intergenic;
2	rs_2_83775708	83775708	A	G	PC1	77.99387	Intergenic;
2	rs_2_84192768	84192768	T	C	PC1	77.99387	Intergenic;
2	rs_2_85997278	85997278	T	C	PC1	77.99387	Intergenic;
2	rs_2_85997279	85997279	A	C	PC1	77.99387	Intergenic;
2	rs_2_85998790	85998790	T	A	PC1	77.99387	Intergenic;
2	rs_2_86002337	86002337	C	T	PC1	77.99387	Intergenic;
2	rs_2_86005721	86005721	T	C	PC1	77.99387	Intergenic;
2	rs_2_86006658	86006658	T	C	PC1	77.99387	Intergenic;
2	rs_2_86011087	86011087	A	G	PC1	77.99387	Intergenic;
2	rs_2_86011777	86011777	A	G	PC1	77.99387	Intergenic;
2	rs_2_86012045	86012045	C	T	PC1	77.99387	Intergenic;
2	rs_2_86012600	86012600	A	T	PC1	77.99387	Intergenic;
2	rs_2_86012787	86012787	A	G	PC1	77.99387	Intergenic;
2	rs_2_86013079	86013079	G	C	PC1	77.99387	Intergenic;
2	rs_2_86013218	86013218	G	A	PC1	77.99387	Intergenic;
2	rs_2_86015135	86015135	A	G	PC1	77.99387	Intergenic;
2	rs_2_86016626	86016626	T	C	PC1	77.99387	Intergenic;

2	rs_2_86017545	86017545	A	C	PC1	77.99387	Intergenic;
2	rs_2_86017677	86017677	T	C	PC1	77.99387	Intergenic;
2	rs_2_86017681	86017681	C	T	PC1	77.99387	Intergenic;
2	rs_2_86020221	86020221	C	T	PC1	77.99387	Intergenic;
2	rs_2_86020293	86020293	A	G	PC1	77.99387	Intergenic;
2	rs_2_86020294	86020294	T	A	PC1	77.99387	Intergenic;
2	rs_2_86020708	86020708	T	C	PC1	77.99387	Intergenic;
2	rs_2_86021314	86021314	A	C	PC1	77.99387	Intergenic;
2	rs_2_86022702	86022702	T	C	PC1	77.99387	Intergenic;
2	rs_2_86025403	86025403	A	G	PC1	77.99387	Intergenic;
2	rs_2_86027029	86027029	A	G	PC1	77.99387	Intergenic;
2	rs_2_86028017	86028017	G	A	PC1	77.99387	Intergenic;
2	rs_2_86029269	86029269	T	C	PC1	77.99387	Intergenic;
2	rs_2_86029645	86029645	T	A	PC1	77.99387	Intergenic;
2	rs_2_86029896	86029896	T	C	PC1	77.99387	Intergenic;
2	rs_2_86030239	86030239	T	C	PC1	77.99387	Intergenic;
2	rs_2_86030453	86030453	T	C	PC1	77.99387	Intergenic;
2	rs_2_86031078	86031078	T	C	PC1	77.99387	Intergenic;
2	rs_2_86032211	86032211	A	C	PC1	77.99387	Intergenic;
2	rs_2_86032239	86032239	C	T	PC1	77.99387	Intergenic;
2	rs_2_86032573	86032573	G	A	PC1	77.99387	Intergenic;
2	rs_2_86032860	86032860	G	T	PC1	77.99387	Intergenic;
2	rs_2_86033144	86033144	C	T	PC1	77.99387	Intergenic;
2	rs_2_86033671	86033671	G	C	PC1	77.99387	Intergenic;
2	rs_2_86034243	86034243	T	C	PC1	77.99387	Intergenic;
2	rs_2_86034318	86034318	G	A	PC1	77.99387	Intergenic;
2	rs_2_86037040	86037040	T	C	PC1	77.99387	Intergenic;
2	rs_2_88921020	88921020	G	A	PC1	77.99387	Intergenic;
2	rs_2_88975189	88975189	A	T	PC1	77.99387	Intergenic;
2	rs_2_89141930	89141930	C	A	PC1	77.99387	Intergenic;
2	rs_2_89223288	89223288	T	G	PC1	77.99387	Intergenic;
2	rs_2_89232209	89232209	A	G	PC1	77.99387	Intergenic;
2	rs_2_89236120	89236120	A	G	PC1	77.99387	Intergenic;
2	rs_2_89237183	89237183	G	C	PC1	77.99387	Intergenic;
2	rs_2_89238375	89238375	G	A	PC1	77.99387	Intergenic;
2	rs_2_89261146	89261146	G	C	PC1	77.99387	Intergenic;
2	rs_2_89273676	89273676	C	T	PC1	77.99387	Intergenic;
2	rs_2_89282939	89282939	A	G	PC1	77.99387	Intergenic;
2	rs_2_89284064	89284064	T	G	PC1	77.99387	Intergenic;
2	rs_2_89312296	89312296	C	T	PC1	77.99387	Intergenic;
2	rs_2_89315009	89315009	T	C	PC1	77.99387	Intergenic;
2	rs_2_89317320	89317320	T	C	PC1	77.99387	Intergenic;
2	rs_2_89328083	89328083	C	A	PC1	77.99387	Intergenic;
2	rs_2_89333286	89333286	T	C	PC1	77.99387	Intergenic;
2	rs_2_89335167	89335167	A	G	PC1	77.99387	Intergenic;
2	rs_2_89338409	89338409	C	A	PC1	77.99387	Intergenic;
2	rs_2_89339656	89339656	C	T	PC1	77.99387	Intergenic;
2	rs_2_89341819	89341819	A	G	PC1	77.99387	Intergenic;
2	rs_2_89347677	89347677	T	C	PC1	77.99387	Intergenic;
2	rs_2_89349266	89349266	C	T	PC1	77.99387	Intergenic;
2	rs_2_89349345	89349345	T	C	PC1	77.99387	Intergenic;
2	rs_2_89360607	89360607	G	A	PC1	77.99387	Intergenic;
2	rs_2_89374008	89374008	G	A	PC1	77.99387	Intergenic;
2	rs_2_89378654	89378654	C	T	PC1	77.99387	Intergenic;
2	rs_2_89379209	89379209	T	A	PC1	77.99387	Intergenic;
2	rs_2_89379366	89379366	G	A	PC1	77.99387	Intergenic;
2	rs_2_89381855	89381855	C	G	PC1	77.99387	Intergenic;
2	rs_2_89381927	89381927	A	G	PC1	77.99387	Intergenic;
2	rs_2_89382863	89382863	T	C	PC1	77.99387	Intergenic;
2	rs_2_89382883	89382883	T	C	PC1	77.99387	Intergenic;
2	rs_2_89387816	89387816	G	C	PC1	77.99387	Intergenic;
2	rs_2_89388009	89388009	T	C	PC1	77.99387	Intergenic;
2	rs_2_89394656	89394656	G	C	PC1	77.99387	Intergenic;
2	rs_2_89395697	89395697	C	T	PC1	77.99387	Intergenic;
2	rs_2_89396559	89396559	G	A	PC1	77.99387	Intergenic;
2	rs_2_89400846	89400846	T	C	PC1	77.99387	Intergenic;
2	rs_2_89406720	89406720	C	G	PC1	77.99387	Intergenic;
2	rs_2_89406944	89406944	C	G	PC1	77.99387	Intergenic;
2	rs_2_89407487	89407487	A	G	PC1	77.99387	Intergenic;
2	rs_2_89407858	89407858	C	G	PC1	77.99387	Intergenic;
2	rs_2_89413319	89413319	T	C	PC1	77.99387	Intergenic;
2	rs_2_89441320	89441320	T	G	PC1	77.99387	Intergenic;
2	rs_2_89464173	89464173	T	C	PC1	77.99387	Intergenic;
2	rs_2_89473443	89473443	A	T	PC1	77.99387	Intergenic;
2	rs_2_89479802	89479802	G	A	PC1	77.99387	Intergenic;
2	rs_2_89480600	89480600	C	T	PC1	77.99387	Intergenic;
2	rs_2_89496616	89496616	G	A	PC1	77.99387	Intergenic;
2	rs_2_89498858	89498858	A	G	PC1	77.99387	Intergenic;
2	rs_2_89517641	89517641	A	G	PC1	77.99387	Intergenic;
2	rs_2_89519406	89519406	C	G	PC1	77.99387	Intergenic;
2	rs_2_89579428	89579428	T	C	PC1	77.99387	Intergenic;
2	rs_2_89586501	89586501	C	T	PC1	77.99387	Intergenic;
2	rs_2_89599571	89599571	C	T	PC1	77.99387	Predicted;upstream;
2	rs_2_89600269	89600269	C	A	PC1	77.99387	Intergenic;
2	rs_2_89605402	89605402	T	C	PC1	77.99387	Intron;IRX1-A;
2	rs_2_89608710	89608710	A	G	PC1	77.99387	Intron;IRX1-A;
2	rs_2_90056508	90056508	T	C	Others	77.97479	Intergenic;
2	rs_2_90059928	90059928	G	A	Others	77.97479	Intergenic;

2	rs_2_92352313	92352313	T	C	PC1	77.99387	Intron;PARD6G;
2	rs_2_93655599	93655599	T	A	PC1	77.99387	Intergenic;
2	rs_2_94080060	94080060	T	C	PC1	77.99387	Intron;ZNF407;
2	rs_2_94080216	94080216	A	G	PC1	77.99387	Intron;ZNF407;
2	rs_2_94080638	94080638	G	A	PC1	77.99387	Intron;ZNF407;
2	rs_2_94080659	94080659	G	A	PC1	77.99387	Intron;ZNF407;
2	rs_2_94082302	94082302	A	G	PC1	77.99387	Intron;ZNF407;
2	rs_2_94082400	94082400	T	G	PC1	77.99387	Intron;ZNF407;
2	rs_2_94082418	94082418	T	C	PC1	77.99387	Intron;ZNF407;
2	rs_2_94082429	94082429	T	C	PC1	77.99387	Intron;ZNF407;
2	rs_2_94082492	94082492	T	G	PC1	77.99387	Intron;ZNF407;
2	rs_2_94082614	94082614	G	C	PC1	77.99387	Intron;ZNF407;
2	rs_2_94082656	94082656	A	G	PC1	77.99387	Intron;ZNF407;
2	rs_2_94082728	94082728	T	C	PC1	77.99387	Intron;ZNF407;
2	rs_2_94654077	94654077	T	A	PC1	77.99387	Intergenic;
2	rs_2_94654901	94654901	T	C	PC1	77.99387	Intergenic;
2	rs_2_94773615	94773615	T	C	PC1	77.99387	Intergenic;
2	rs_2_94828808	94828808	T	A	PC1	77.99387	Intergenic;
2	rs_2_94831688	94831688	C	T	PC1	77.99387	Intergenic;
2	rs_2_94835764	94835764	A	T	PC1	77.99387	Intergenic;
2	rs_2_95002876	95002876	G	A	PC1	77.99387	Intergenic;
2	rs_2_95004203	95004203	A	C	PC1	77.99387	Intergenic;
2	rs_2_95005470	95005470	T	C	PC1	77.99387	Intergenic;
2	rs_2_95006116	95006116	C	G	PC1	77.99387	Intergenic;
2	rs_2_95007042	95007042	C	T	PC1	77.99387	Intergenic;
2	rs_2_95009719	95009719	G	A	PC1	77.99387	Intergenic;
2	rs_2_95009764	95009764	T	C	PC1	77.99387	Intergenic;
2	rs_2_95011720	95011720	C	T	PC1	77.99387	Intergenic;
2	rs_2_95019929	95019929	T	C	PC1	77.99387	Intergenic;
2	rs_2_95029969	95029969	T	C	PC1	77.99387	CUBN;upstream;
2	rs_2_95030005	95030005	C	G	PC1	77.99387	CUBN;upstream;
2	rs_2_95030007	95030007	G	A	PC1	77.99387	CUBN;upstream;
2	rs_2_95033294	95033294	C	G	PC1	77.99387	CUBN;upstream;
2	rs_2_95033351	95033351	G	C	PC1	77.99387	CUBN;upstream;
2	rs_2_95033420	95033420	G	C	PC1	77.99387	CUBN;upstream;
2	rs_2_95033519	95033519	A	G	PC1	77.99387	CUBN;upstream;
2	rs_2_95033725	95033725	A	G	PC1	77.99387	CUBN;upstream;
2	rs_2_95034168	95034168	T	C	PC1	77.99387	CUBN;upstream;
2	rs_2_95034781	95034781	A	G	PC1	77.99387	CUBN;upstream;
2	rs_2_95069723	95069723	G	T	PC1	77.99387	Intron;CUBN;
2	rs_2_95078718	95078718	C	T	PC1	77.99387	Intron;CUBN;
2	rs_2_95079083	95079083	G	A	PC1	77.99387	Intron;CUBN;
2	rs_2_95095531	95095531	G	T	PC1	77.99387	Intron;CUBN;
2	rs_2_95116706	95116706	G	C	PC1	77.99387	Intergenic;
2	rs_2_95117826	95117826	A	T	PC1	77.99387	Intergenic;
2	rs_2_95348047	95348047	A	G	PC1	77.99387	Intergenic;
2	rs_2_95474423	95474423	C	T	PC1	77.99387	Intergenic;
2	rs_2_95481769	95481769	G	C	PC1	77.99387	Intergenic;
2	rs_2_95483581	95483581	T	C	PC1	77.99387	Intergenic;
2	rs_2_95483756	95483756	T	G	PC1	77.99387	Intergenic;
2	rs_2_95709801	95709801	G	T	PC1	77.99387	Intergenic;
2	rs_2_95710003	95710003	T	C	PC1	77.99387	Intergenic;
2	rs_2_95870718	95870718	A	C	PC1	77.99387	Intergenic;
2	rs_2_95872423	95872423	C	G	PC1	77.99387	Intergenic;
2	rs_2_95882141	95882141	A	G	PC1	77.99387	Intergenic;
2	rs_2_98065388	98065388	T	A	Others	80.68132	Intergenic;
2	rs_2_98065393	98065393	A	C	Others	80.68132	Intergenic;
2	rs_2_98778988	98778988	T	G	PC1	77.99387	Intergenic;
2	rs_2_98779283	98779283	G	C	PC1	77.99387	Intergenic;
2	rs_2_98787749	98787749	T	A	PC1	77.99387	Intergenic;
2	rs_2_98848527	98848527	T	C	PC1	77.99387	Intergenic;
2	rs_2_98848565	98848565	G	A	PC1	77.99387	Intergenic;
2	rs_2_98848793	98848793	G	A	PC1	77.99387	Intergenic;
2	rs_2_98876659	98876659	T	A	PC1	77.99387	Intergenic;
2	rs_2_98879212	98879212	A	G	PC1	77.99387	Intergenic;
2	rs_2_98882804	98882804	T	C	PC1	77.99387	Intergenic;
2	rs_2_98893162	98893162	A	G	PC1	77.99387	Intergenic;
2	rs_2_98896367	98896367	G	C	PC1	77.99387	Intergenic;
2	rs_2_98902724	98902724	T	C	PC1	77.99387	Intergenic;
2	rs_2_100017065	100017065	G	T	PC1	77.99387	Intron;GNAL;
2	rs_2_100038961	100038961	C	T	PC1	77.99387	Intron;GNAL;
2	rs_2_100040382	100040382	T	A	PC1	77.99387	Intron;GNAL;
2	rs_2_100040407	100040407	T	A	PC1	77.99387	Intron;GNAL;
2	rs_2_100141290	100141290	T	C	PC1	77.99387	Intergenic;
2	rs_2_100143869	100143869	T	G	PC1	77.99387	Intergenic;
2	rs_2_100145674	100145674	G	A	PC1	77.99387	Intergenic;
2	rs_2_100146418	100146418	A	G	PC1	77.99387	Intergenic;
2	rs_2_100147236	100147236	C	T	PC1	77.99387	Intergenic;
2	rs_2_100367563	100367563	A	G	PC1	77.99387	Intron;PIEZO2;
2	rs_2_100371385	100371385	G	A	PC1	77.99387	Intron;PIEZO2;
2	rs_2_100563622	100563622	A	G	PC1	77.99387	Intron;PIEZO2;
2	rs_2_100564871	100564871	C	A	PC1	77.99387	Intron;PIEZO2;
2	rs_2_100565237	100565237	T	C	PC1	77.99387	Intron;PIEZO2;
2	rs_2_100565580	100565580	G	T	PC1	77.99387	Intron;PIEZO2;
2	rs_2_100570211	100570211	T	C	PC1	77.99387	Intron;PIEZO2;
2	rs_2_100570659	100570659	T	C	PC1	77.99387	Intron;PIEZO2;
2	rs_2_100571673	100571673	G	A	PC1	77.99387	Intron;PIEZO2;
2	rs_2_101029871	101029871	A	G	PC1	77.99387	Intergenic;

2	rs_2_105934152	105934152	T	C	PC2	79.32426	LAMA3;downstream;TTC39C;upstream;
2	rs_2_110172831	110172831	T	C	PC2	87.30961	Intergenic;
2	rs_2_110174805	110174805	C	T	PC2	87.30961	Intergenic;
2	rs_2_110184826	110184826	C	G	PC2	87.30961	Intergenic;
2	rs_2_110192566	110192566	A	C	PC2	87.30961	Intergenic;
2	rs_2_110193339	110193339	A	G	PC2	87.30961	Intergenic;
2	rs_2_110194158	110194158	A	T	PC2	87.30961	Intergenic;
2	rs_2_110194257	110194257	A	G	PC2	87.30961	Intergenic;
2	rs_2_110194286	110194286	A	T	PC2	87.30961	Intergenic;
2	rs_2_110199396	110199396	A	G	PC2	87.30961	Intergenic;
2	rs_2_110201868	110201868	G	C	PC2	87.30961	Intergenic;
2	rs_2_110220828	110220828	G	A	PC2	87.30961	Intergenic;
2	rs_2_110226050	110226050	G	A	PC2	78.26123	DTNA;upstream;
2	rs_2_110228318	110228318	A	T	PC2	78.26123	DTNA;upstream;
2	rs_2_110230391	110230391	A	G	PC2	78.26123	DTNA;upstream;
2	rs_2_110243299	110243299	G	T	PC2	78.26123	Intron;DTNA;
2	rs_2_110245308	110245308	T	C	PC2	78.26123	Intron;DTNA;
2	rs_2_110249164	110249164	C	T	PC2	87.30961	Intron;DTNA;
2	rs_2_110254086	110254086	A	T	PC2	87.30961	Intron;DTNA;
2	rs_2_110257795	110257795	C	A	PC2	87.30961	Intron;DTNA;
2	rs_2_110274265	110274265	T	G	PC2	87.30961	Intron;DTNA;
2	rs_2_110280027	110280027	G	A	PC2	87.30961	Intron;DTNA;
2	rs_2_110294034	110294034	G	A	PC2	87.30961	Intron;DTNA;
2	rs_2_110311260	110311260	C	T	PC2	78.0371	Intron;DTNA;
2	rs_2_110311502	110311502	T	C	PC2	78.0371	Exon;DTNA;DTNA;+;1;ATT;Nonsynonymous;Ile;Thr;
2	rs_2_110314052	110314052	C	T	PC2	78.0371	DTNA;downstream;DTNA;upstream;
2	rs_2_110319231	110319231	T	C	PC2	79.1455	DTNA;downstream;DTNA;upstream;
2	rs_2_110321162	110321162	C	G	PC2	87.30961	Intron;DTNA;
2	rs_2_110321261	110321261	T	C	PC2	87.30961	Intron;DTNA;
2	rs_2_118678083	118678083	G	A	PC1	83.7223	Intron;PREX2;
2	rs_2_118678090	118678090	G	A	PC1	83.7223	Intron;PREX2;
2	rs_2_120629489	120629489	A	G	PC1	83.7223	Intron;KCNB2;
2	rs_2_120630510	120630510	A	C	PC1	83.7223	Intron;KCNB2;
2	rs_2_120631142	120631142	A	G	PC1	83.7223	Intron;KCNB2;
2	rs_2_120631679	120631679	C	T	PC1	83.7223	Intron;KCNB2;
2	rs_2_120631694	120631694	G	A	PC1	83.7223	Intron;KCNB2;
2	rs_2_120631771	120631771	C	T	PC1	83.7223	Intron;KCNB2;
2	rs_2_120631807	120631807	T	A	PC1	83.7223	Intron;KCNB2;
2	rs_2_120631934	120631934	T	C	PC1	83.7223	Intron;KCNB2;
2	rs_2_120632512	120632512	C	T	PC1	83.7223	Intron;KCNB2;
2	rs_2_120632909	120632909	T	C	PC1	83.7223	Intron;KCNB2;
2	rs_2_120632974	120632974	G	A	PC1	83.7223	Intron;KCNB2;
2	rs_2_120804242	120804242	T	C	PC1	83.7223	RDH10;downstream;
2	rs_2_120804331	120804331	A	G	PC1	83.7223	RDH10;downstream;
2	rs_2_120805258	120805258	A	G	PC1	83.7223	RDH10;downstream;
2	rs_2_120832673	120832673	T	C	PC1	83.7223	STAU2;downstream;
2	rs_2_121334694	121334694	C	T	PC1	83.7223	Intergenic;
2	rs_2_121336240	121336240	C	T	PC1	83.7223	Intergenic;
2	rs_2_121336573	121336573	T	C	PC1	83.7223	Intergenic;
2	rs_2_121341020	121341020	G	A	PC1	83.7223	Intergenic;
2	rs_2_121342758	121342758	T	G	PC1	83.7223	Intergenic;
2	rs_2_121344010	121344010	T	G	PC1	83.7223	Intergenic;
2	rs_2_121346146	121346146	T	C	PC1	83.7223	Intergenic;
2	rs_2_121762112	121762112	T	C	PC1	83.7223	Intergenic;
2	rs_2_121763329	121763329	C	T	PC1	83.7223	Intergenic;
2	rs_2_121764018	121764018	T	A	PC1	83.7223	Intergenic;
2	rs_2_121764200	121764200	C	G	PC1	83.7223	Intergenic;
2	rs_2_121870021	121870021	T	C	PC1	83.7223	Intergenic;
2	rs_2_121870297	121870297	A	G	PC1	83.7223	Intergenic;
2	rs_2_121870596	121870596	G	A	PC1	83.7223	Intergenic;
2	rs_2_121873891	121873891	T	A	PC1	83.7223	Intergenic;
2	rs_2_122293040	122293040	C	T	PC1	83.7223	Intergenic;
2	rs_2_122346745	122346745	G	T	PC1	83.7223	Intergenic;
2	rs_2_122348888	122348888	T	C	PC1	83.7223	Intergenic;
2	rs_2_122375846	122375846	T	C	PC1	83.7223	Intergenic;
2	rs_2_122396393	122396393	G	A	PC1	83.7223	Intron;ZFHX4;
2	rs_2_122424471	122424471	G	A	PC1	83.7223	Intron;ZFHX4;
2	rs_2_122425351	122425351	A	G	PC1	83.7223	Intron;ZFHX4;
2	rs_2_122426238	122426238	A	C	PC1	83.7223	Intron;ZFHX4;
2	rs_2_122426299	122426299	C	T	PC1	83.7223	Intron;ZFHX4;
2	rs_2_122427703	122427703	C	A	PC1	83.7223	Intron;ZFHX4;
2	rs_2_122428242	122428242	T	C	PC1	83.7223	Intron;ZFHX4;
2	rs_2_122428654	122428654	A	G	PC1	83.7223	Intron;ZFHX4;
2	rs_2_122429159	122429159	C	T	PC1	77.99387	Intron;ZFHX4;
2	rs_2_122430132	122430132	T	A	PC1	83.7223	Intron;ZFHX4;
2	rs_2_122431192	122431192	G	A	PC1	83.7223	Intron;ZFHX4;
2	rs_2_122432042	122432042	G	A	PC1	83.7223	Intron;ZFHX4;
2	rs_2_122432335	122432335	A	G	PC1	83.7223	Intron;ZFHX4;
2	rs_2_122434761	122434761	C	T	PC1	83.7223	Intron;ZFHX4;
2	rs_2_122438276	122438276	G	A	PC1	83.7223	Intron;ZFHX4;
2	rs_2_122439581	122439581	A	G	PC1	83.7223	Intron;ZFHX4;
2	rs_2_122442423	122442423	G	T	PC1	83.7223	Intron;ZFHX4;
2	rs_2_122453406	122453406	A	G	PC1	83.7223	Intron;ZFHX4;
2	rs_2_122457067	122457067	T	C	PC1	83.7223	Intron;ZFHX4;
2	rs_2_122461036	122461036	G	T	PC1	83.7223	Intron;ZFHX4;
2	rs_2_122467484	122467484	C	A	PC1	83.7223	Intron;ZFHX4;
2	rs_2_122537407	122537407	G	A	PC1	83.7223	Intergenic;
2	rs_2_122555148	122555148	A	G	PC1	83.7223	PEX2;downstream;

2	rs_2_122577601	122577601	A	G	PC1	83.7223	Intergenic;
2	rs_2_122585386	122585386	T	C	PC1	83.7223	Intergenic;
2	rs_2_123038422	123038422	C	T	PC1	83.7223	Intergenic;
2	rs_2_123038919	123038919	C	A	PC1	83.7223	Intergenic;
2	rs_2_123039303	123039303	C	T	PC1	83.7223	Intergenic;
2	rs_2_123039437	123039437	G	T	PC1	83.7223	Intergenic;
2	rs_2_123040244	123040244	T	A	PC1	83.7223	Intergenic;
2	rs_2_123040377	123040377	C	A	PC1	83.7223	Intergenic;
2	rs_2_123041735	123041735	C	T	PC1	77.99387	Intergenic;
2	rs_2_123043119	123043119	C	T	PC1	83.7223	Intergenic;
2	rs_2_123162609	123162609	G	A	PC1	83.7223	Intergenic;
2	rs_2_123168410	123168410	C	A	PC1	83.7223	Intergenic;
2	rs_2_123169635	123169635	A	G	PC1	83.7223	Intergenic;
2	rs_2_123169956	123169956	A	G	PC1	83.7223	Intergenic;
2	rs_2_123170480	123170480	C	T	PC1	83.7223	Intergenic;
2	rs_2_123173021	123173021	C	T	PC1	83.7223	Intergenic;
2	rs_2_123205442	123205442	A	G	PC1	83.7223	Intergenic;
2	rs_2_123206259	123206259	C	G	PC1	83.7223	Intergenic;
2	rs_2_123244531	123244531	A	G	PC1	83.7223	Intergenic;
2	rs_2_126518531	126518531	A	T	PC1	83.7223	Intergenic;
2	rs_2_134576827	134576827	A	T	PC1	77.99387	Intron;RSPO1;
2	rs_2_134578958	134578958	G	A	PC1	77.99387	Intron;RSPO1;
2	rs_2_134862384	134862384	A	T	PC1	77.99387	Intergenic;
2	rs_2_135229057	135229057	A	G	PC1	77.99387	Intergenic;
2	rs_2_135915365	135915365	C	T	PC1	77.99387	Intergenic;
2	rs_2_135927357	135927357	C	T	PC1	77.99387	Intergenic;
2	rs_2_135991475	135991475	C	T	PC1	77.99387	Intergenic;
2	rs_2_135996660	135996660	G	A	PC1	77.99387	Intergenic;
2	rs_2_135998010	135998010	G	A	PC1	77.99387	Intergenic;
2	rs_2_135998096	135998096	T	A	PC1	77.99387	Intergenic;
2	rs_2_135998177	135998177	G	A	PC1	77.99387	Intergenic;
2	rs_2_136002249	136002249	T	C	PC1	77.99387	Intergenic;
2	rs_2_136004458	136004458	A	T	PC1	77.99387	Intergenic;
2	rs_2_136012479	136012479	G	A	PC1	77.99387	Intergenic;
2	rs_2_136012907	136012907	T	C	PC1	77.99387	Intergenic;
2	rs_2_136013558	136013558	G	A	PC1	77.99387	Intergenic;
2	rs_2_136022652	136022652	C	T	PC1	77.99387	Intergenic;
2	rs_2_136022708	136022708	T	C	PC1	77.99387	Intergenic;
2	rs_2_136024760	136024760	C	T	PC1	77.99387	Intergenic;
2	rs_2_136060227	136060227	A	C	PC1	77.99387	Intergenic;
2	rs_2_136154670	136154670	G	T	PC1	77.99387	CSMD3;downstream;
2	rs_2_136262629	136262629	T	C	PC1	77.99387	Intron;CSMD3;
2	rs_2_136275778	136275778	G	A	PC1	77.99387	Intron;CSMD3;
2	rs_2_136278987	136278987	G	C	PC1	77.99387	Intron;CSMD3;
2	rs_2_136283061	136283061	G	A	PC1	77.99387	Intron;CSMD3;
2	rs_2_136552818	136552818	T	C	PC1	77.99387	Intron;CSMD3;
2	rs_2_136567316	136567316	T	C	PC1	77.99387	Intron;CSMD3;
2	rs_2_137129312	137129312	A	C	PC1	77.99387	Intergenic;
2	rs_2_137132399	137132399	A	C	PC1	77.99387	Intergenic;
2	rs_2_137132487	137132487	T	G	PC1	77.99387	Intergenic;
2	rs_2_137135414	137135414	T	C	PC1	77.99387	Intergenic;
2	rs_2_137137214	137137214	T	C	PC1	77.99387	Intergenic;
2	rs_2_137147567	137147567	C	A	PC1	77.99387	Intergenic;
2	rs_2_137261561	137261561	T	G	PC1	77.99387	Intergenic;
2	rs_2_137278236	137278236	T	C	PC1	77.99387	Intergenic;
2	rs_2_137558253	137558253	A	G	PC1	77.99387	Intergenic;
2	rs_2_137558983	137558983	T	C	PC1	77.99387	Intergenic;
2	rs_2_137575764	137575764	A	C	PC1	77.99387	Intergenic;
2	rs_2_137576282	137576282	G	A	PC1	77.99387	Intergenic;
2	rs_2_137595901	137595901	C	T	PC1	77.99387	Intergenic;
2	rs_2_137597471	137597471	T	C	PC1	77.99387	Intergenic;
2	rs_2_137597665	137597665	A	C	PC1	77.99387	Intergenic;
2	rs_2_137598797	137598797	C	T	PC1	77.99387	Intergenic;
2	rs_2_137598884	137598884	T	C	PC1	77.99387	Intergenic;
2	rs_2_137599141	137599141	A	C	PC1	77.99387	Intergenic;
2	rs_2_137599418	137599418	T	C	PC1	77.99387	Intergenic;
2	rs_2_137599483	137599483	G	A	PC1	77.99387	Intergenic;
2	rs_2_137599541	137599541	T	C	PC1	77.99387	Intergenic;
2	rs_2_137600637	137600637	A	G	PC1	77.99387	Intergenic;
2	rs_2_137641309	137641309	A	G	PC1	77.99387	Intergenic;
2	rs_2_137730305	137730305	A	G	PC1	77.99387	Intergenic;
2	rs_2_137730422	137730422	G	A	PC1	77.99387	Intergenic;
2	rs_2_137737700	137737700	T	C	PC1	77.99387	Intergenic;
2	rs_2_138326531	138326531	T	C	PC1	77.99387	Intron;Predicted;
2	rs_2_138660498	138660498	T	C	PC1	77.99387	Intron;EXT1;
2	rs_2_139060057	139060057	C	G	PC1	77.99387	Intergenic;
2	rs_2_139707883	139707883	C	A	PC1	77.99387	Intron;VPS4A;
2	rs_2_141912868	141912868	C	G	PC1	77.99387	Intergenic;
2	rs_2_142439410	142439410	A	T	PC1	77.99387	Intergenic;
2	rs_2_142447579	142447579	T	G	PC1	77.99387	Intergenic;
2	rs_2_142479085	142479085	T	C	PC1	77.99387	Intron;Predicted;
2	rs_2_142481050	142481050	A	G	PC1	77.99387	Intron;Predicted;
2	rs_2_142484067	142484067	A	G	PC1	77.99387	Intron;Predicted;
2	rs_2_142486328	142486328	A	G	PC1	77.99387	Predicted;upstream;
2	rs_2_142487194	142487194	T	G	PC1	77.99387	Predicted;upstream;
2	rs_2_143641246	143641246	C	T	PC1	77.99387	Intergenic;
2	rs_2_144563394	144563394	A	G	PC1	77.99387	Intron;PHF20L1;
2	rs_2_144563950	144563950	C	G	PC1	77.99387	Intron;PHF20L1;

2	rs_2_144564165	144564165	C	G	PC1	77.99387	Intron;PHF20L1;
2	rs_2_144571894	144571894	T	A	PC1	77.99387	Intron;PHF20L1;
2	rs_2_144572008	144572008	A	G	PC1	77.99387	Exon;PHF20L1;PHF20L1;+;2;GGA;Synonymous;Gly;Gly;
2	rs_2_144934084	144934084	G	A	PC1	77.99387	Intron;ST3GAL1;
2	rs_2_145024122	145024122	G	T	PC1	77.99387	Intergenic;
2	rs_2_14915235€	149152356	T	C	PC1	77.99387	Intergenic;
2	rs_2_14915245C	149152450	C	T	PC1	77.99387	Intergenic;
2	rs_2_14915261€	149152616	G	A	PC1	77.99387	Intergenic;
2	rs_2_149152621	149152621	G	A	PC1	77.99387	Intergenic;
2	rs_2_14953878S	149538789	T	C	PC1	77.99387	Intergenic;
2	rs_2_14953904S	149539049	T	G	PC1	77.99387	Intergenic;
2	rs_2_14953920S	149539209	G	A	PC1	77.99387	Intergenic;
2	rs_2_14953970€	149539706	G	A	PC1	77.99387	Intergenic;
2	rs_2_149539981	149539981	C	T	PC1	77.99387	Intergenic;
2	rs_2_149540022	149540022	C	G	PC1	77.99387	Intergenic;
2	rs_2_14963498S	149634989	A	T	PC1	77.99387	Intergenic;
2	rs_2_149635115	149635115	C	A	PC1	77.99387	Intergenic;
2	rs_2_149635804	149635804	G	C	PC1	77.99387	Intergenic;
2	rs_2_151659648	151659648	T	C	PC1	77.99387	Intergenic;
2	rs_2_151659991	151659991	A	G	PC1	77.99387	Intergenic;
2	rs_2_151660255	151660255	T	C	PC1	77.99387	Intergenic;
2	rs_2_15166110€	151661106	A	G	PC1	77.99387	Intergenic;
3	rs_3_4594228	4594228	A	G	PC1	77.99387	Intergenic;
3	rs_3_4595366	4595366	C	T	PC1	77.99387	Intergenic;
3	rs_3_4595538	4595538	C	T	PC1	77.99387	Intergenic;
3	rs_3_4610241	4610241	G	T	PC1	77.99387	Intergenic;
3	rs_3_4614007	4614007	T	C	PC1	77.99387	Intergenic;
3	rs_3_4616109	4616109	C	T	PC1	77.99387	Intergenic;
3	rs_3_4616352	4616352	T	C	PC1	77.99387	Intergenic;
3	rs_3_4619009	4619009	C	G	PC1	77.99387	Intergenic;
3	rs_3_4856726	4856726	C	T	PC1	77.99387	Intergenic;
3	rs_3_4857206	4857206	G	A	PC1	77.99387	Intergenic;
3	rs_3_5805447	5805447	G	A	PC2	76.66582	Intron;PUM2;
3	rs_3_5815412	5815412	A	C	PC2	79.01227	Intron;PUM2;
3	rs_3_5817898	5817898	T	A	PC2	79.01227	Intron;PUM2;
3	rs_3_5977786	5977786	G	C	Others	78.48173	Intergenic;
3	rs_3_6999046	6999046	C	A	PC2	80.34186	Intergenic;
3	rs_3_7003630	7003630	C	T	PC2	86.03218	Intergenic;
3	rs_3_7006220	7006220	G	A	PC2	86.03218	Intergenic;
3	rs_3_7011934	7011934	C	T	PC2	86.03218	Intergenic;
3	rs_3_7013517	7013517	G	T	PC2	86.03218	Intergenic;
3	rs_3_7018766	7018766	T	C	PC2	86.03218	Intergenic;
3	rs_3_7020673	7020673	T	G	PC2	86.03218	Intergenic;
3	rs_3_7026415	7026415	G	A	PC2	86.03218	Intergenic;
3	rs_3_7029898	7029898	A	G	PC2	86.03218	Intergenic;
3	rs_3_7034112	7034112	A	G	PC2	81.12781	Intergenic;
3	rs_3_7038836	7038836	G	T	PC2	77.10355	Intergenic;
3	rs_3_7040172	7040172	C	G	PC2	86.03218	Intergenic;
3	rs_3_7044244	7044244	C	A	PC2	86.03218	Intergenic;
3	rs_3_7047034	7047034	G	A	PC2	86.03218	Intergenic;
3	rs_3_7051152	7051152	A	G	PC2	86.03218	Intergenic;
3	rs_3_7051310	7051310	T	C	PC2	86.03218	Intergenic;
3	rs_3_7104682	7104682	C	A	PC2	79.33795	Intergenic;
3	rs_3_8421094	8421094	G	A	PC2	89.24531	Intron;CYP39A1;
3	rs_3_10320914	10320914	C	A	PC1	77.99387	Intergenic;
3	rs_3_10409017	10409017	A	T	PC1	77.99387	Intergenic;
3	rs_3_10506477	10506477	A	G	PC1	77.99387	Intergenic;
3	rs_3_12893108	12893108	G	A	PC1	77.99387	Intergenic;
3	rs_3_14546185	14546185	T	G	PC1	77.99387	Intergenic;
3	rs_3_15649745	15649745	G	A	PC1	77.99387	Intergenic;
3	rs_3_16819872	16819872	T	C	PC1	77.99387	Intergenic;
3	rs_3_17492911	17492911	C	T	PC1	77.99387	Intergenic;
3	rs_3_18051664	18051664	A	C	PC1	77.99387	Intergenic;
3	rs_3_18131411	18131411	C	T	PC1	77.99387	Intergenic;
3	rs_3_22236508	22236508	C	T	PC1	77.99387	Intergenic;
3	rs_3_22255735	22255735	C	T	PC1	77.99387	Intergenic;
3	rs_3_22662467	22662467	T	C	PC1	77.99387	Intergenic;
3	rs_3_23944460	23944460	G	A	PC1	77.99387	Intron;FAM83D;
3	rs_3_24109754	24109754	T	C	PC1	77.99387	Intron;GFRA1;
3	rs_3_25340128	25340128	A	T	PC1	77.99387	Intron;KHDRBS2;
3	rs_3_29895855	29895855	A	G	PC1	77.99387	Intron;RIMS1;
3	rs_3_30052723	30052723	A	G	PC1	77.99387	Intergenic;
3	rs_3_30113619	30113619	G	A	PC1	77.99387	Intergenic;
3	rs_3_31189646	31189646	C	T	PC1	77.99387	Intron;COL12A1;
3	rs_3_31198351	31198351	T	C	PC1	77.99387	COL12A1;upstream;
3	rs_3_39448117	39448117	T	C	PC1	77.99387	Intergenic;
3	rs_3_39448440	39448440	C	A	PC1	77.99387	Intergenic;
3	rs_3_40535134	40535134	A	G	PC1	77.99387	Intergenic;
3	rs_3_40552635	40552635	T	C	PC1	77.99387	Intergenic;
3	rs_3_41238668	41238668	C	A	PC1	77.99387	Intron;ASCC3;
3	rs_3_41301003	41301003	A	G	PC1	77.99387	Intron;ASCC3;
3	rs_3_41423252	41423252	T	C	PC1	77.99387	Intron;ASCC3;
3	rs_3_43586713	43586713	T	C	PC1	77.99387	Intergenic;
3	rs_3_43609079	43609079	G	A	PC1	77.99387	Intergenic;
3	rs_3_43609587	43609587	T	G	PC1	77.99387	Intergenic;
3	rs_3_43646560	43646560	A	G	PC1	77.99387	Intergenic;
3	rs_3_43668766	43668766	C	A	PC1	77.99387	Intergenic;
3	rs_3_43695280	43695280	T	G	PC1	77.99387	Intergenic;

3	rs_3_43696302	43696302	G	A	PC1	77.99387	Intergenic;
3	rs_3_43696326	43696326	G	A	PC1	77.99387	Intergenic;
3	rs_3_43696395	43696395	T	C	PC1	77.99387	Intergenic;
3	rs_3_43696570	43696570	T	G	PC1	77.99387	Intergenic;
3	rs_3_43696683	43696683	T	C	PC1	77.99387	Intergenic;
3	rs_3_43698302	43698302	A	G	PC1	77.99387	Intergenic;
3	rs_3_43700263	43700263	A	T	PC1	77.99387	Intergenic;
3	rs_3_43714479	43714479	T	C	PC1	77.99387	Intergenic;
3	rs_3_43715290	43715290	G	T	PC1	77.99387	Intergenic;
3	rs_3_43717244	43717244	A	T	PC1	77.99387	Intergenic;
3	rs_3_43718473	43718473	G	A	PC1	77.99387	Intergenic;
3	rs_3_43718541	43718541	T	C	PC1	77.99387	Intergenic;
3	rs_3_43719713	43719713	A	G	PC1	77.99387	Intergenic;
3	rs_3_43722244	43722244	A	G	PC1	77.99387	Intergenic;
3	rs_3_43722543	43722543	T	A	PC1	77.99387	Intergenic;
3	rs_3_43750234	43750234	C	T	PC1	77.99387	Intergenic;
3	rs_3_43755393	43755393	C	G	PC1	77.99387	Intergenic;
3	rs_3_43755658	43755658	T	C	PC1	77.99387	Intergenic;
3	rs_3_44007783	44007783	C	A	PC1	77.99387	Intergenic;
3	rs_3_44012487	44012487	T	C	PC1	77.99387	Intergenic;
3	rs_3_44552706	44552706	C	G	PC1	77.99387	Intergenic;
3	rs_3_44554695	44554695	A	G	PC1	77.99387	SCML4;downstream;
3	rs_3_44561937	44561937	T	A	PC1	77.99387	SCML4;downstream;
3	rs_3_44562108	44562108	A	G	PC1	77.99387	SCML4;downstream;
3	rs_3_45965621	45965621	A	G	PC1	77.99387	AMD1;downstream;
3	rs_3_46579525	46579525	C	G	PC1	77.99387	Intron;LAMA4;
3	rs_3_46580236	46580236	T	C	PC1	77.99387	Intron;LAMA4;
3	rs_3_46581407	46581407	T	C	PC1	77.99387	Intron;LAMA4;
3	rs_3_46582147	46582147	G	C	PC1	77.99387	Intron;LAMA4;
3	rs_3_46604101	46604101	T	C	PC1	77.99387	Intron;LAMA4;
3	rs_3_46677313	46677313	T	C	PC1	77.99387	Intergenic;
3	rs_3_48796059	48796059	T	C	PC1	77.99387	Intergenic;
3	rs_3_48818794	48818794	A	G	PC1	77.99387	Exon;VGLL2;VGLL2;+;1;AAG;Nonsynonymous;Lys;Arg;
3	rs_3_48829956	48829956	A	G	PC1	77.99387	Intron;ROS1;
3	rs_3_48849114	48849114	T	C	PC1	77.99387	Intron;ROS1;
3	rs_3_48863780	48863780	C	T	PC1	77.99387	Intron;ROS1;
3	rs_3_48870052	48870052	C	A	PC1	77.99387	Intron;ROS1;
3	rs_3_48870441	48870441	T	C	PC1	77.99387	Exon;ROS1;ROS1;-;0;ATA;Nonsynonymous;Ile;Val;
3	rs_3_48898779	48898779	G	A	PC1	77.99387	Intron;ROS1;
3	rs_3_49191687	49191687	C	T	PC1	77.99387	Intron;SLC35F1;
3	rs_3_49195362	49195362	A	T	PC1	77.99387	Intron;SLC35F1;
3	rs_3_49226004	49226004	G	A	PC1	77.99387	Intron;SLC35F1;
3	rs_3_49230574	49230574	A	G	PC1	77.99387	Intron;SLC35F1;
3	rs_3_49237649	49237649	G	C	PC1	77.99387	Intron;SLC35F1;
3	rs_3_49248201	49248201	T	C	PC1	77.99387	Intron;SLC35F1;
3	rs_3_49260024	49260024	A	T	PC1	77.99387	Intron;SLC35F1;
3	rs_3_49316151	49316151	A	T	PC1	77.99387	Intron;SLC35F1;
3	rs_3_49502634	49502634	A	C	PC1	77.99387	CEP85L;upstream;
3	rs_3_49897018	49897018	T	C	PC1	77.99387	Intron;MAN1A1;
3	rs_3_49898064	49898064	G	A	PC1	77.99387	Intron;MAN1A1;
3	rs_3_49909478	49909478	T	A	PC1	77.99387	Intergenic;
3	rs_3_49909496	49909496	A	G	PC1	77.99387	Intergenic;
3	rs_3_49941949	49941949	G	A	PC1	77.99387	Intergenic;
3	rs_3_49956741	49956741	C	T	PC1	77.99387	Intergenic;
3	rs_3_49959922	49959922	T	C	PC1	77.99387	Intergenic;
3	rs_3_49998245	49998245	T	C	PC1	77.99387	Intergenic;
3	rs_3_50022747	50022747	T	G	PC1	77.99387	Intergenic;
3	rs_3_50839889	50839889	G	C	PC1	77.99387	Intergenic;
3	rs_3_51696636	51696636	T	A	PC1	77.99387	Intergenic;
3	rs_3_51721328	51721328	C	T	PC1	77.99387	Intergenic;
3	rs_3_51821374	51821374	A	C	PC1	77.99387	Intergenic;
3	rs_3_52147978	52147978	T	A	PC1	77.99387	Intron;NKAIN2;
3	rs_3_52204540	52204540	G	C	PC1	77.99387	Intron;NKAIN2;
3	rs_3_52210936	52210936	A	T	PC1	77.99387	Intron;NKAIN2;
3	rs_3_52602675	52602675	T	C	PC1	77.99387	HEY2;upstream;
3	rs_3_52602770	52602770	A	T	PC1	77.99387	HEY2;upstream;
3	rs_3_52602980	52602980	T	G	PC1	77.99387	HEY2;upstream;
3	rs_3_52797739	52797739	A	G	PC1	77.99387	Intron;Predicted;
3	rs_3_52888729	52888729	G	A	PC1	77.99387	Intron;CENPW;
3	rs_3_52900932	52900932	C	T	PC1	77.99387	CENPW;downstream;
3	rs_3_52906774	52906774	G	A	PC1	77.99387	CENPW;downstream;
3	rs_3_52912811	52912811	T	C	PC1	77.99387	Intergenic;
3	rs_3_52914700	52914700	A	G	PC1	77.99387	Intergenic;
3	rs_3_52925086	52925086	T	C	PC1	77.99387	Intergenic;
3	rs_3_52947516	52947516	T	C	PC1	77.99387	Intergenic;
3	rs_3_52963118	52963118	G	T	PC1	77.99387	Intergenic;
3	rs_3_52972053	52972053	A	G	PC1	77.99387	Intergenic;
3	rs_3_52973054	52973054	G	A	PC1	77.99387	Intergenic;
3	rs_3_52986846	52986846	C	G	PC1	77.99387	Intergenic;
3	rs_3_52994981	52994981	G	A	PC1	77.99387	Intergenic;
3	rs_3_52995211	52995211	C	T	PC1	77.99387	Intergenic;
3	rs_3_53027301	53027301	G	A	PC1	77.99387	Intergenic;
3	rs_3_53027550	53027550	A	G	PC1	77.99387	Intergenic;
3	rs_3_53044334	53044334	A	G	PC1	77.99387	Intergenic;
3	rs_3_53048594	53048594	A	C	PC1	77.99387	Intergenic;
3	rs_3_53090269	53090269	A	G	PC1	77.99387	Intergenic;
3	rs_3_53097824	53097824	A	G	PC1	77.99387	Intergenic;
3	rs_3_53101844	53101844	G	A	PC1	77.99387	Intergenic;

3	rs_3_53119890	53119890	T	A	PC1	77.99387	Intergenic;
3	rs_3_53127739	53127739	A	C	PC1	77.99387	Intergenic;
3	rs_3_53163518	53163518	T	C	PC1	77.99387	Intergenic;
3	rs_3_53195456	53195456	A	T	PC1	77.99387	RSPO1;upstream;
3	rs_3_53795846	53795846	T	C	PC1	77.99387	Intron;PTPRK;
3	rs_3_53796163	53796163	T	C	PC1	77.99387	Intron;PTPRK;
3	rs_3_53803723	53803723	T	A	PC1	77.99387	Intron;PTPRK;
3	rs_3_53808822	53808822	A	G	PC1	77.99387	Intron;PTPRK;
3	rs_3_53850328	53850328	G	T	PC1	77.99387	Intron;PTPRK;
3	rs_3_53882415	53882415	A	C	PC1	77.99387	Intergenic;
3	rs_3_53905410	53905410	G	C	PC1	77.99387	Predicted;downstream;
3	rs_3_53919926	53919926	G	A	PC1	77.99387	Intron;Predicted;
3	rs_3_53927184	53927184	A	G	PC1	77.99387	Intron;Predicted;
3	rs_3_54937152	54937152	C	T	PC1	77.99387	EPB41L2;downstream;
3	rs_3_54937905	54937905	T	C	PC1	77.99387	Intron;EPB41L2;
3	rs_3_54937954	54937954	T	G	PC1	77.99387	Intron;EPB41L2;
3	rs_3_54942205	54942205	C	T	PC1	77.99387	Intron;EPB41L2;
3	rs_3_54943746	54943746	A	G	PC1	77.99387	Intron;EPB41L2;
3	rs_3_54944367	54944367	A	G	PC1	77.99387	Intron;EPB41L2;
3	rs_3_54944687	54944687	T	C	PC1	77.99387	Intron;EPB41L2;
3	rs_3_54946994	54946994	C	T	PC1	77.99387	Exon;EPB41L2;EPB41L2;-;2;TCG;Synonymous;Ser;Ser;
3	rs_3_54949144	54949144	G	A	PC1	77.99387	Intron;EPB41L2;
3	rs_3_54972205	54972205	T	A	PC1	77.99387	Intron;EPB41L2;
3	rs_3_54974378	54974378	G	C	PC1	77.99387	Intron;EPB41L2;
3	rs_3_54975747	54975747	C	T	PC1	77.99387	Intron;EPB41L2;
3	rs_3_54977510	54977510	T	A	PC1	77.99387	Intron;EPB41L2;
3	rs_3_55035037	55035037	C	T	PC1	77.99387	Intergenic;
3	rs_3_55239797	55239797	A	C	PC1	77.99387	Intron;MED23;
3	rs_3_55494052	55494052	G	A	PC1	77.99387	Intergenic;
3	rs_3_55595723	55595723	C	T	PC1	77.99387	Intergenic;
3	rs_3_55714273	55714273	T	C	PC1	77.99387	STX7;upstream;
3	rs_3_55715988	55715988	G	A	PC1	77.99387	STX7;upstream;
3	rs_3_55732164	55732164	G	A	PC1	77.99387	Intergenic;
3	rs_3_55733109	55733109	A	C	PC1	77.99387	Intergenic;
3	rs_3_55736506	55736506	G	C	PC1	77.99387	Intergenic;
3	rs_3_55741239	55741239	T	C	PC1	77.99387	Intergenic;
3	rs_3_55787094	55787094	A	G	PC1	77.99387	Exon;VNN1;VNN1;-;1;CTA;Nonsynonymous;Leu;Pro;
3	rs_3_55790901	55790901	T	A	PC1	77.99387	Intron;VNN1;
3	rs_3_55808307	55808307	T	C	PC1	77.99387	Intron;VNN1;
3	rs_3_55822237	55822237	T	C	PC1	77.99387	Intron;SLC18B1;
3	rs_3_55824104	55824104	G	A	PC1	77.99387	Intron;SLC18B1;
3	rs_3_55843502	55843502	G	T	PC1	77.99387	SLC18B1;upstream;
3	rs_3_55899264	55899264	C	T	PC1	77.99387	Intergenic;
3	rs_3_55920036	55920036	A	G	PC1	77.99387	Intergenic;
3	rs_3_55976200	55976200	T	A	PC1	77.99387	Intergenic;
3	rs_3_55983157	55983157	C	T	PC1	77.99387	Intergenic;
3	rs_3_55983513	55983513	A	T	PC1	77.99387	Intergenic;
3	rs_3_55985688	55985688	C	G	PC1	77.99387	Intergenic;
3	rs_3_55986535	55986535	G	A	PC1	77.99387	Intergenic;
3	rs_3_55999995	55999995	A	G	PC1	77.99387	Intergenic;
3	rs_3_56005179	56005179	C	T	PC1	77.99387	Intergenic;
3	rs_3_56007077	56007077	C	T	PC1	77.99387	Intergenic;
3	rs_3_56008620	56008620	C	T	PC1	77.99387	Intergenic;
3	rs_3_56010259	56010259	C	T	PC1	77.99387	Intergenic;
3	rs_3_56011635	56011635	A	G	PC1	77.99387	Intergenic;
3	rs_3_56012355	56012355	C	T	PC1	77.99387	Intergenic;
3	rs_3_56013747	56013747	T	C	PC1	77.99387	Intergenic;
3	rs_3_56013758	56013758	A	T	PC1	77.99387	Intergenic;
3	rs_3_56015663	56015663	T	C	PC1	77.99387	Intergenic;
3	rs_3_56017071	56017071	T	C	PC1	77.99387	Intergenic;
3	rs_3_56021903	56021903	A	T	PC1	77.99387	Intergenic;
3	rs_3_56028818	56028818	A	G	PC1	77.99387	Intergenic;
3	rs_3_56045941	56045941	A	G	PC1	77.99387	Intergenic;
3	rs_3_56047671	56047671	G	A	PC1	77.99387	Intergenic;
3	rs_3_57277730	57277730	A	T	PC1	77.99387	Intergenic;
3	rs_3_57586251	57586251	T	C	PC1	77.99387	Intron;MAP7;
3	rs_3_57586280	57586280	A	G	PC1	77.99387	Intron;MAP7;
3	rs_3_57590268	57590268	A	G	PC1	77.99387	Intron;MAP7;
3	rs_3_58224366	58224366	C	T	PC1	77.99387	ARFGEF3;upstream;
3	rs_3_58454109	58454109	G	A	PC1	77.99387	Intron;NHSL1;
3	rs_3_58944116	58944116	T	C	PC1	77.99387	Intergenic;
3	rs_3_58979196	58979196	A	G	PC1	77.99387	Intergenic;
3	rs_3_59019466	59019466	A	T	PC1	77.99387	Intergenic;
3	rs_3_59068487	59068487	G	C	PC1	77.99387	Intergenic;
3	rs_3_59078893	59078893	T	C	PC1	77.99387	Intergenic;
3	rs_3_59090674	59090674	G	A	PC1	77.99387	Intergenic;
3	rs_3_59406137	59406137	A	G	PC1	77.99387	Intergenic;
3	rs_3_59412567	59412567	A	G	PC1	77.99387	Intergenic;
3	rs_3_59414143	59414143	T	G	PC1	77.99387	Intergenic;
3	rs_3_59421758	59421758	T	A	PC1	77.99387	Intergenic;
3	rs_3_59469941	59469941	C	T	PC1	77.99387	Intergenic;
3	rs_3_59475207	59475207	G	A	PC1	77.99387	Intergenic;
3	rs_3_59505759	59505759	A	G	PC1	77.99387	Intergenic;
3	rs_3_59582869	59582869	T	C	PC1	77.99387	Intergenic;
3	rs_3_59620818	59620818	C	T	PC1	77.99387	Intergenic;
3	rs_3_59627263	59627263	A	C	PC1	77.99387	Intergenic;
3	rs_3_59627302	59627302	A	T	PC1	77.99387	Intergenic;
3	rs_3_59636047	59636047	G	A	PC1	77.99387	Intergenic;

3	rs_3_59645204	59645204	G	A	PC1	77.99387	Intergenic;
3	rs_3_59657338	59657338	A	C	PC1	77.99387	Intergenic;
3	rs_3_59684997	59684997	G	A	PC1	77.99387	Intron;NMBR;
3	rs_3_59736941	59736941	G	A	PC1	77.99387	Intron;VTA1;
3	rs_3_59859658	59859658	C	A	PC1	77.99387	Intron;ADGRG6;
3	rs_3_59906093	59906093	A	C	PC1	77.99387	Intergenic;
3	rs_3_60009203	60009203	G	A	PC1	77.99387	HIVEP2;upstream;
3	rs_3_60022081	60022081	G	A	PC1	77.99387	Intergenic;
3	rs_3_60022082	60022082	T	A	PC1	77.99387	Intergenic;
3	rs_3_60026580	60026580	A	G	PC1	77.99387	Intergenic;
3	rs_3_60032128	60032128	G	A	PC1	77.99387	Intergenic;
3	rs_3_60057294	60057294	T	G	PC1	77.99387	Intergenic;
3	rs_3_60066029	60066029	G	T	PC1	77.99387	Intergenic;
3	rs_3_60066906	60066906	A	G	PC1	77.99387	Intergenic;
3	rs_3_62119867	62119867	A	G	PC1	77.99387	NOX3;upstream;
3	rs_3_62706170	62706170	A	C	PC1	77.99387	Intron;CNKSR3;
3	rs_3_62707912	62707912	C	T	PC1	77.99387	Intron;CNKSR3;
3	rs_3_71188644	71188644	C	T	PC1	77.99387	Intergenic;
3	rs_3_88472569	88472569	G	T	PC1	77.99387	Intron;THADA;
3	rs_3_88473231	88473231	A	C	PC1	77.99387	Intron;THADA;
3	rs_3_94626251	94626251	C	T	PC1	77.99387	Intergenic;
3	rs_3_98159972	98159972	T	C	PC1	77.99387	Exon;RRBP1;RRBP1;-;2;GCA;Synonymous;Ala;Ala;
3	rs_3_98286255	98286255	T	G	PC1	77.99387	KAT14;downstream;DZANK1;downstream;
3	rs_3_10104779ε	101047798	T	G	PC1	77.99387	Intron;ISM1;
4	rs_4_535384	535384	G	A	PC2	84.68457	Intron;NOT2;
4	rs_4_7861812	7861812	A	G	PC2	80.36519	Intron;Predicted;
4	rs_4_7862596	7862596	A	G	PC2	80.36519	Intron;Predicted;
4	rs_4_7866444	7866444	G	A	PC2	80.36519	Intron;Predicted;
4	rs_4_7866483	7866483	C	T	PC2	80.36519	Intron;Predicted;
4	rs_4_72274829	72274829	T	G	PC1	87.09052	Intergenic;
4	rs_4_73984175	73984175	A	G	PC1	87.09052	Intron;GPC2;
4	rs_4_76142825	76142825	A	C	PC1	87.09052	Intron;NEU2;
4	rs_4_97685526	97685526	A	G	PC2	80.05914	Intron;MED12L;
4	rs_4_97693394	97693394	C	T	PC2	80.05914	Intron;MED12L;
4	rs_4_97694590	97694590	C	G	PC2	80.05914	Intron;MED12L;
4	rs_4_97709495	97709495	G	C	PC2	80.05914	Intron;MED12L;
4	rs_4_97714949	97714949	A	G	PC2	80.05914	Intron;MED12L;
4	rs_4_97716949	97716949	G	A	PC2	80.05914	Intron;MED12L;
4	rs_4_97725727	97725727	A	G	PC2	80.05914	Intron;MED12L;
4	rs_4_97726964	97726964	G	A	PC2	80.05914	Intron;MED12L;
4	rs_4_97734275	97734275	G	A	PC2	80.05914	Intron;MED12L;
4	rs_4_97734560	97734560	C	T	PC2	80.05914	Intron;MED12L;
4	rs_4_97735205	97735205	C	T	PC2	80.05914	Intron;MED12L;
4	rs_4_97735562	97735562	C	T	PC2	80.05914	Intron;MED12L;
4	rs_4_97736022	97736022	C	T	PC2	80.05914	Intron;MED12L;
4	rs_4_97738003	97738003	C	T	PC2	80.05914	Intron;MED12L;
4	rs_4_97738179	97738179	T	C	PC2	80.05914	Intron;MED12L;
4	rs_4_97823453	97823453	A	G	PC2	178.1954	Intron;AADAC;
4	rs_4_97825084	97825084	T	G	PC2	142.0226	Intergenic;
4	rs_4_97825096	97825096	G	A	PC2	142.0226	Intergenic;
4	rs_4_97827422	97827422	A	G	PC2	79.50003	AADAC;downstream;
4	rs_4_97827764	97827764	G	A	PC2	79.50003	AADAC;downstream;
4	rs_4_97905279	97905279	C	T	PC2	222.8195	Intergenic;
4	rs_4_97986176	97986176	C	T	PC1	87.09052	Intergenic;
6	rs_6_1133765	1133765	C	T	PC2	77.02905	GALNT13;upstream;
6	rs_6_1134094	1134094	T	C	PC2	77.02905	GALNT13;upstream;
6	rs_6_1134600	1134600	G	A	PC2	77.02905	GALNT13;upstream;
6	rs_6_1139626	1139626	C	T	PC2	77.02905	GALNT13;upstream;
6	rs_6_1143101	1143101	A	G	PC2	77.02905	Intergenic;
6	rs_6_1143616	1143616	G	A	PC2	77.02905	Intergenic;
6	rs_6_1145203	1145203	C	T	PC2	77.02905	Intergenic;
6	rs_6_1145331	1145331	C	T	PC2	77.02905	Intergenic;
6	rs_6_1146167	1146167	G	A	PC2	77.02905	Intergenic;
6	rs_6_1176986	1176986	A	G	PC2	77.02905	Intergenic;
6	rs_6_1177137	1177137	C	T	PC2	77.02905	Intergenic;
6	rs_6_1649189	1649189	T	G	PC2	85.28884	Intron;NEB;
6	rs_6_1668517	1668517	T	A	PC2	76.67661	Intron;NEB;
6	rs_6_1677909	1677909	G	A	PC2	83.50386	Intron;NEB;
6	rs_6_3796115	3796115	T	C	PC2	80.76628	Intergenic;
6	rs_6_4172546	4172546	T	A	PC2	80.59031	Intron;ARHGAP15;
6	rs_6_14235218	14235218	A	G	PC1	77.99387	Intron;IGFBP3;
6	rs_6_14806925	14806925	G	A	PC1	77.99387	Predicted;downstream;
6	rs_6_17703627	17703627	C	T	PC1	77.99387	Intron;SCN2A;
6	rs_6_23461205	23461205	C	T	PC1	77.99387	CERKL;upstream;
6	rs_6_23461542	23461542	A	G	PC1	77.99387	CERKL;upstream;
6	rs_6_23581619	23581619	A	G	PC1	77.99387	Intergenic;
6	rs_6_24947842	24947842	G	A	PC1	77.99387	PARD3;downstream;
6	rs_6_24991864	24991864	G	A	PC1	77.99387	Intron;PARD3B;
6	rs_6_25469092	25469092	A	C	PC1	77.99387	Intergenic;
6	rs_6_25479271	25479271	A	G	PC1	77.99387	KLF7;downstream;
6	rs_6_27043059	27043059	T	C	PC1	77.99387	Intron;SATB2;
6	rs_6_30282852	30282852	T	C	PC1	77.99387	PMS1;downstream;
6	rs_6_35047241	35047241	A	G	PC1	77.99387	Intron;UBE2F;
6	rs_6_36004173	36004173	G	C	PC1	77.99387	Intergenic;
6	rs_6_37168039	37168039	C	T	PC1	77.99387	Intron;NDUFA10;
6	rs_6_37676351	37676351	T	G	PC1	77.99387	Intron;PCNT;
6	rs_6_37684009	37684009	A	G	PC1	77.99387	Intron;PCNT;
6	rs_6_38545948	38545948	G	A	PC1	77.99387	PTEN;downstream;

6	rs_6_38762131	38762131	G	T	PC1	77.99387	Intron;ASAH2;
6	rs_6_38775785	38775785	A	C	PC1	77.99387	Intron;ASAH2;
6	rs_6_38904322	38904322	A	G	PC1	77.99387	Intron;PRKG1;
6	rs_6_39048209	39048209	T	C	PC1	77.99387	Intron;PRKG1;
6	rs_6_42307102	42307102	A	G	PC1	77.99387	Intron;MYPN;
6	rs_6_42361708	42361708	T	C	PC1	77.99387	Intergenic;
6	rs_6_43885331	43885331	C	T	PC1	77.99387	Intron;NRBF2;
6	rs_6_44473136	44473136	T	C	PC1	77.99387	Intergenic;
6	rs_6_44506829	44506829	T	G	PC1	77.99387	Intergenic;
6	rs_6_44532576	44532576	G	A	PC1	77.99387	Intergenic;
6	rs_6_44532648	44532648	C	T	PC1	77.99387	Intergenic;
6	rs_6_44536057	44536057	T	G	PC1	77.99387	Intergenic;
6	rs_6_44536999	44536999	C	T	PC1	77.99387	Intergenic;
6	rs_6_44717560	44717560	C	T	PC1	77.99387	Intergenic;
6	rs_6_45762375	45762375	A	T	Others	79.60398	Intergenic;
6	rs_6_51131127	51131127	G	A	PC1	77.99387	Intron;DLG5;
6	rs_6_52475693	52475693	C	T	PC1	77.99387	LRMDA;downstream;
6	rs_6_52663511	52663511	T	C	PC1	77.99387	Intergenic;
6	rs_6_52702833	52702833	C	T	PC1	77.99387	Intergenic;
6	rs_6_52736145	52736145	T	C	PC1	77.99387	Intergenic;
6	rs_6_52976924	52976924	T	C	PC1	77.99387	Intron;KAT6B;
6	rs_6_53574656	53574656	G	T	PC1	77.99387	Intron;CAMK2G;
6	rs_6_55222395	55222395	C	T	PC1	77.99387	Intergenic;
6	rs_6_55258004	55258004	C	T	PC1	77.99387	Intergenic;
6	rs_6_56039641	56039641	G	A	PC1	77.99387	GDF10;downstream;
6	rs_6_58052001	58052001	C	T	PC1	77.99387	TNKS2;downstream;
6	rs_6_60797981	60797981	A	G	PC1	77.99387	Intron;FGF8;
6	rs_6_71728986	71728986	G	C	PC2	77.54163	Intergenic;
6	rs_6_71909869	71909869	G	A	PC2	93.97475	Intergenic;
6	rs_6_71921549	71921549	T	C	PC2	110.6834	Intergenic;
6	rs_6_71924943	71924943	G	A	PC2	86.03377	Intergenic;
6	rs_6_71925623	71925623	C	A	PC2	96.76057	Intergenic;
6	rs_6_71925984	71925984	T	A	PC2	79.47646	Intergenic;
6	rs_6_71926007	71926007	A	G	PC2	80.04118	Intergenic;
6	rs_6_71926018	71926018	C	T	PC2	86.61728	Intergenic;
6	rs_6_71926165	71926165	A	G	PC2	86.61728	Intergenic;
6	rs_6_71926304	71926304	G	A	PC2	86.61728	Intergenic;
6	rs_6_72570817	72570817	T	C	PC2	109.3025	Intron;EBF3;
6	rs_6_72571693	72571693	G	C	PC2	109.3025	Intron;EBF3;
6	rs_6_72575296	72575296	C	T	PC2	96.71756	Intron;EBF3;
6	rs_6_72580407	72580407	G	T	PC2	96.71756	Intron;EBF3;
6	rs_6_72580794	72580794	C	T	PC2	103.1367	Intron;EBF3;
6	rs_6_72582041	72582041	C	T	PC2	96.71756	Intron;EBF3;
6	rs_6_72582088	72582088	C	T	PC2	84.34933	Intron;EBF3;
6	rs_6_72582250	72582250	G	A	PC2	96.71756	Intron;EBF3;
6	rs_6_72669741	72669741	C	T	PC2	76.98302	GLRX3;downstream;
6	rs_6_72675174	72675174	T	C	PC2	87.43001	GLRX3;downstream;
6	rs_6_73311357	73311357	G	A	PC2	81.96061	Intergenic;
6	rs_6_73318034	73318034	C	T	PC2	81.96061	Intergenic;
6	rs_6_73331620	73331620	C	T	PC2	81.96061	PWWP2B;upstream;
6	rs_6_73331830	73331830	G	A	PC2	81.96061	PWWP2B;upstream;
6	rs_6_73332446	73332446	C	T	PC2	78.19662	PWWP2B;upstream;
7	rs_7_816851	816851	C	G	PC2	126.3475	Intergenic;
7	rs_7_944973	944973	T	C	Others	82.90595	Intron;ANO3;
7	rs_7_3540652	3540652	A	G	PC1	83.7223	PRMT3;upstream;
7	rs_7_3561602	3561602	G	A	PC1	83.7223	Intron;PRMT3;
7	rs_7_5224463	5224463	T	C	PC1	83.7223	Intron;Q5REB1;
7	rs_7_5225794	5225794	C	G	PC1	83.7223	Intron;Q5REB1;
7	rs_7_5226253	5226253	A	G	PC1	83.7223	Intron;Q5REB1;
7	rs_7_5228263	5228263	T	C	PC1	83.7223	Intron;Q5REB1;
7	rs_7_5229146	5229146	G	A	PC1	83.7223	Intron;Q5REB1;
7	rs_7_5726888	5726888	C	T	PC1	83.7223	Intron;ELP4;
7	rs_7_5726969	5726969	G	A	PC1	83.7223	Intron;ELP4;
7	rs_7_5730252	5730252	T	C	PC1	83.7223	Intron;ELP4;
7	rs_7_5741819	5741819	T	G	PC1	83.7223	Intron;ELP4;
7	rs_7_5745044	5745044	T	A	PC1	83.7223	Intron;ELP4;
7	rs_7_5751039	5751039	A	G	PC1	83.7223	Intron;ELP4;
7	rs_7_5752420	5752420	G	A	PC1	83.7223	Intron;ELP4;
7	rs_7_5755315	5755315	C	T	PC1	83.7223	Intron;ELP4;
7	rs_7_5756779	5756779	C	T	PC1	83.7223	Intron;ELP4;
7	rs_7_5799045	5799045	C	A	PC1	83.7223	ELP4;downstream;PAX6;downstream;
7	rs_7_5799132	5799132	C	T	PC1	83.7223	ELP4;downstream;PAX6;downstream;
7	rs_7_6232063	6232063	C	T	PC1	83.7223	Intron;PRR12;
7	rs_7_10195488	10195488	T	C	PC1	83.7223	Intergenic;
7	rs_7_15514259	15514259	C	A	PC1	83.7223	Intron;TSPAN4;
7	rs_7_17385879	17385879	T	C	PC1	83.7223	Predicted;upstream;
7	rs_7_17386662	17386662	G	A	PC1	83.7223	Predicted;upstream;
7	rs_7_17408105	17408105	T	C	PC1	83.7223	Predicted;downstream;
7	rs_7_17410139	17410139	C	T	PC1	83.7223	Intergenic;
7	rs_7_17430043	17430043	T	C	PC1	83.7223	Intergenic;
7	rs_7_17532943	17532943	G	T	PC1	83.7223	Intergenic;
7	rs_7_18241163	18241163	C	T	PC1	83.7223	Intron;SHANK2;
7	rs_7_18241392	18241392	A	T	PC1	83.7223	Intron;SHANK2;
7	rs_7_18245506	18245506	A	G	PC1	83.7223	Intron;SHANK2;
7	rs_7_18249232	18249232	G	A	PC1	83.7223	Intron;SHANK2;
7	rs_7_18250977	18250977	T	C	PC1	83.7223	Intron;SHANK2;
7	rs_7_18254166	18254166	G	A	PC1	83.7223	Intron;SHANK2;
7	rs_7_18362384	18362384	A	G	PC1	83.7223	Intron;SHANK2;

7	rs_7_18365521	18365521	C	T	PC1	83.7223	Intron;SHANK2;
7	rs_7_19035776	19035776	C	A	PC1	77.99387	EHF;downstream;
7	rs_7_20136248	20136248	C	T	PC1	83.7223	Intergenic;
7	rs_7_20137288	20137288	G	A	PC1	83.7223	Intergenic;
7	rs_7_20702266	20702266	G	A	PC1	83.7223	Intergenic;
7	rs_7_20708230	20708230	A	G	PC1	83.7223	Intergenic;
7	rs_7_20709212	20709212	T	C	PC1	83.7223	Intergenic;
7	rs_7_20709654	20709654	A	G	PC1	83.7223	Intergenic;
7	rs_7_20709916	20709916	A	G	PC1	77.99387	Intergenic;
7	rs_7_20710570	20710570	C	T	PC1	83.7223	Intergenic;
7	rs_7_20710988	20710988	T	A	PC1	83.7223	Intergenic;
7	rs_7_20711461	20711461	G	A	PC1	83.7223	Intergenic;
7	rs_7_20717971	20717971	T	G	PC1	83.7223	Intergenic;
7	rs_7_20719484	20719484	C	T	PC1	83.7223	Intergenic;
7	rs_7_20720439	20720439	T	C	PC1	83.7223	Intergenic;
7	rs_7_20733628	20733628	T	C	PC1	83.7223	Intergenic;
7	rs_7_20738304	20738304	A	G	PC1	83.7223	Intergenic;
7	rs_7_20741325	20741325	G	A	PC1	83.7223	Intergenic;
7	rs_7_20744407	20744407	G	A	PC1	83.7223	Intergenic;
7	rs_7_20772350	20772350	C	G	PC1	83.7223	Intergenic;
7	rs_7_21365488	21365488	A	C	PC1	83.7223	Intergenic;
7	rs_7_21367410	21367410	C	T	PC1	83.7223	Intergenic;
7	rs_7_21372346	21372346	T	A	PC1	83.7223	Intergenic;
7	rs_7_21376461	21376461	G	C	PC1	83.7223	Intergenic;
7	rs_7_21378050	21378050	A	C	PC1	83.7223	Intergenic;
7	rs_7_21381246	21381246	T	G	PC1	83.7223	Intergenic;
7	rs_7_21383752	21383752	G	A	PC1	83.7223	Predicted;upstream;
7	rs_7_21391825	21391825	T	G	PC1	83.7223	Predicted;upstream;
7	rs_7_21393116	21393116	A	C	PC1	83.7223	Intron;Predicted;
7	rs_7_21414777	21414777	T	C	PC1	83.7223	Intergenic;
7	rs_7_22390437	22390437	G	A	PC1	83.7223	Intergenic;
7	rs_7_22399648	22399648	G	T	PC1	83.7223	Intergenic;
7	rs_7_22406679	22406679	C	T	PC1	83.7223	Intergenic;
7	rs_7_22409179	22409179	T	C	PC1	83.7223	Intergenic;
7	rs_7_22409611	22409611	T	C	PC1	83.7223	Intergenic;
7	rs_7_22416291	22416291	C	T	PC1	83.7223	Intergenic;
7	rs_7_22454019	22454019	T	A	PC1	83.7223	Intron;CD82;
7	rs_7_22489620	22489620	T	C	PC1	83.7223	Intergenic;
7	rs_7_22489648	22489648	T	C	PC1	83.7223	Intergenic;
7	rs_7_23027959	23027959	T	G	PC1	83.7223	Intergenic;
7	rs_7_23031219	23031219	C	T	PC1	83.7223	Intergenic;
7	rs_7_23031576	23031576	T	C	PC1	83.7223	Intergenic;
7	rs_7_23035985	23035985	T	G	PC1	83.7223	Intergenic;
7	rs_7_23825737	23825737	G	A	PC1	83.7223	LRP4;upstream;
7	rs_7_24249056	24249056	A	G	PC1	83.7223	Intergenic;
7	rs_7_24566684	24566684	G	A	PC1	83.7223	Intergenic;
7	rs_7_24567413	24567413	A	C	PC1	83.7223	Intergenic;
7	rs_7_24765338	24765338	G	A	PC1	77.99387	Intergenic;
7	rs_7_24775084	24775084	A	T	PC1	77.99387	VPS18;upstream;
7	rs_7_24847994	24847994	T	C	PC1	83.7223	Intergenic;
7	rs_7_24851689	24851689	T	C	PC1	83.7223	Intergenic;
7	rs_7_24853736	24853736	T	C	PC1	83.7223	Intergenic;
7	rs_7_24856188	24856188	T	G	PC1	83.7223	Intergenic;
7	rs_7_24860063	24860063	T	C	PC1	83.7223	Intergenic;
7	rs_7_24860623	24860623	G	A	PC1	83.7223	Intergenic;
7	rs_7_24866805	24866805	A	T	PC1	83.7223	Intergenic;
7	rs_7_24867333	24867333	A	G	PC1	83.7223	Intergenic;
7	rs_7_24867408	24867408	T	C	PC1	83.7223	Intergenic;
7	rs_7_24871699	24871699	T	C	PC1	77.99387	Intergenic;
7	rs_7_24872897	24872897	T	C	PC1	83.7223	Intergenic;
7	rs_7_24874047	24874047	A	G	PC1	83.7223	Intergenic;
7	rs_7_24890671	24890671	T	C	PC1	83.7223	DLL4;upstream;
7	rs_7_24891919	24891919	A	G	PC1	83.7223	DLL4;upstream;
7	rs_7_24892082	24892082	A	G	PC1	83.7223	DLL4;upstream;
7	rs_7_24892617	24892617	T	A	PC1	83.7223	DLL4;upstream;
7	rs_7_24903428	24903428	T	C	PC1	83.7223	Intron;DLL4;
7	rs_7_26324530	26324530	A	G	PC1	83.7223	Intron;STARD9;
7	rs_7_26414855	26414855	G	A	PC1	83.7223	CDAN1;upstream;
7	rs_7_27189237	27189237	T	C	PC1	83.7223	DPF3;downstream;
7	rs_7_27189829	27189829	A	G	PC1	83.7223	DPF3;downstream;
7	rs_7_27424037	27424037	A	T	PC1	83.7223	Predicted;downstream;
7	rs_7_27426796	27426796	A	T	PC1	83.7223	Intergenic;
7	rs_7_27428846	27428846	G	A	PC1	83.7223	Intergenic;
7	rs_7_27750042	27750042	T	C	PC1	83.7223	Intergenic;
7	rs_7_28188136	28188136	T	A	PC1	83.7223	SLC8A3;upstream;
7	rs_7_28206799	28206799	A	G	PC1	83.7223	Intron;SLC8A3;
7	rs_7_28208703	28208703	G	A	PC1	83.7223	Intron;SLC8A3;
7	rs_7_28210680	28210680	C	T	PC1	83.7223	Intron;SLC8A3;
7	rs_7_28215802	28215802	T	C	PC1	83.7223	Intron;SLC8A3;
7	rs_7_28234140	28234140	T	C	PC1	83.7223	Intron;SLC8A3;
7	rs_7_28262888	28262888	T	C	PC1	83.7223	Intron;SLC8A3;
7	rs_7_28361050	28361050	C	T	PC1	83.7223	Intron;SMOC1;
7	rs_7_28367025	28367025	A	G	PC1	83.7223	Intron;SMOC1;
7	rs_7_28611814	28611814	T	C	PC2	86.90625	SLC39A9;upstream;
7	rs_7_28857417	28857417	A	G	PC1	83.7223	Intron;ACTN1;
7	rs_7_28859613	28859613	T	C	PC1	83.7223	Intron;ACTN1;
7	rs_7_28994198	28994198	G	A	PC1	83.7223	Intergenic;
7	rs_7_29724558	29724558	G	C	PC1	83.7223	Intron;GPHN;

7	rs_7_29724834	29724834	C	T	PC1	83.7223	Intron;GPHN;
7	rs_7_29727446	29727446	A	G	PC1	83.7223	Intron;GPHN;
7	rs_7_29922983	29922983	T	C	PC1	83.7223	Intergenic;
7	rs_7_29996309	29996309	G	T	PC1	77.99387	Intergenic;
7	rs_7_30351298	30351298	T	G	PC1	83.7223	Intergenic;
7	rs_7_30352283	30352283	T	C	PC1	83.7223	Intergenic;
7	rs_7_30352983	30352983	A	G	PC1	83.7223	Intergenic;
7	rs_7_30353220	30353220	T	C	PC1	83.7223	Intergenic;
7	rs_7_30360965	30360965	A	G	PC1	83.7223	Intergenic;
7	rs_7_30363425	30363425	A	C	PC1	83.7223	Intergenic;
7	rs_7_30364525	30364525	C	A	PC1	83.7223	Intergenic;
7	rs_7_30376480	30376480	C	T	PC1	83.7223	Intergenic;
7	rs_7_30381404	30381404	T	C	PC1	83.7223	Intergenic;
7	rs_7_30398891	30398891	G	C	PC1	83.7223	Intergenic;
7	rs_7_30406978	30406978	G	A	PC1	83.7223	Intergenic;
7	rs_7_30409282	30409282	C	T	PC1	83.7223	Intergenic;
7	rs_7_30411035	30411035	T	G	PC1	83.7223	Intergenic;
7	rs_7_30640193	30640193	C	G	PC1	83.7223	Intergenic;
7	rs_7_30640970	30640970	A	G	PC1	83.7223	Intergenic;
7	rs_7_31256269	31256269	T	C	PC1	83.7223	Intergenic;
7	rs_7_31256360	31256360	T	C	PC1	83.7223	Intergenic;
7	rs_7_31256361	31256361	C	A	PC1	83.7223	Intergenic;
7	rs_7_32252076	32252076	T	G	PC1	83.7223	Intron;RCJMB04_2G23;
7	rs_7_32255335	32255335	C	T	PC1	83.7223	Intron;RCJMB04_2G23;
7	rs_7_32255764	32255764	A	C	PC1	83.7223	Intron;RCJMB04_2G23;
7	rs_7_32301740	32301740	A	G	PC1	83.7223	Intergenic;
7	rs_7_32303013	32303013	C	G	PC1	77.99387	Intergenic;
7	rs_7_32310675	32310675	A	G	PC1	83.7223	Intergenic;
7	rs_7_32332220	32332220	A	G	PC1	83.7223	Intergenic;
7	rs_7_32341894	32341894	G	C	PC1	83.7223	Intergenic;
7	rs_7_32350522	32350522	C	T	PC1	83.7223	Intergenic;
7	rs_7_32351068	32351068	G	A	PC1	83.7223	Intergenic;
7	rs_7_32365100	32365100	T	C	PC1	83.7223	Intergenic;
7	rs_7_32367026	32367026	T	C	PC1	83.7223	Intergenic;
7	rs_7_32374075	32374075	G	A	PC1	83.7223	Intergenic;
7	rs_7_32387007	32387007	T	C	PC1	83.7223	Intergenic;
7	rs_7_32387969	32387969	G	A	PC1	83.7223	Intergenic;
7	rs_7_32391850	32391850	T	C	PC1	83.7223	Intergenic;
7	rs_7_32398689	32398689	G	A	PC1	83.7223	Intergenic;
7	rs_7_32403926	32403926	G	A	PC1	83.7223	Intergenic;
7	rs_7_32409506	32409506	A	G	PC1	83.7223	Intergenic;
7	rs_7_32409803	32409803	G	A	PC1	83.7223	Intergenic;
7	rs_7_32423667	32423667	T	C	PC1	83.7223	Intergenic;
7	rs_7_32424928	32424928	A	T	PC1	83.7223	Intergenic;
7	rs_7_32433537	32433537	T	C	PC1	83.7223	Intergenic;
7	rs_7_32433722	32433722	A	G	PC1	83.7223	Intergenic;
7	rs_7_32433736	32433736	G	A	PC1	83.7223	Intergenic;
7	rs_7_32452065	32452065	T	C	PC1	83.7223	Intergenic;
7	rs_7_32462222	32462222	A	G	PC1	83.7223	Intergenic;
7	rs_7_32463237	32463237	G	A	PC1	83.7223	Intergenic;
7	rs_7_32471292	32471292	C	T	PC1	83.7223	Intergenic;
7	rs_7_32474446	32474446	C	T	PC1	83.7223	Intergenic;
7	rs_7_32479221	32479221	T	C	PC1	83.7223	Intergenic;
7	rs_7_32489734	32489734	G	A	PC1	83.7223	Intergenic;
7	rs_7_32506948	32506948	T	C	PC1	83.7223	Intergenic;
7	rs_7_32515709	32515709	C	T	PC1	83.7223	Intergenic;
7	rs_7_32520345	32520345	A	G	PC1	83.7223	Intergenic;
7	rs_7_32521019	32521019	G	A	PC1	83.7223	Intergenic;
7	rs_7_32521768	32521768	T	C	PC1	83.7223	Intergenic;
7	rs_7_32522312	32522312	T	A	PC1	83.7223	Intergenic;
7	rs_7_32522713	32522713	G	A	PC1	83.7223	Intergenic;
7	rs_7_32524036	32524036	T	C	PC1	83.7223	Intergenic;
7	rs_7_32530827	32530827	C	A	PC1	83.7223	Intergenic;
7	rs_7_32531178	32531178	C	T	PC1	83.7223	Intergenic;
7	rs_7_32536542	32536542	C	G	PC1	83.7223	Intergenic;
7	rs_7_32537659	32537659	A	C	PC1	83.7223	Intergenic;
7	rs_7_32542066	32542066	A	G	PC1	83.7223	Intergenic;
7	rs_7_32552969	32552969	G	A	PC1	83.7223	Intergenic;
7	rs_7_32554740	32554740	G	A	PC1	83.7223	Intergenic;
7	rs_7_32555633	32555633	T	C	PC1	83.7223	Intergenic;
7	rs_7_32565077	32565077	A	G	PC1	83.7223	Intergenic;
7	rs_7_32565083	32565083	C	T	PC1	83.7223	Intergenic;
7	rs_7_32575495	32575495	A	G	PC1	83.7223	Intergenic;
7	rs_7_32582793	32582793	T	C	PC1	83.7223	Intergenic;
7	rs_7_32586885	32586885	A	C	PC1	83.7223	Intergenic;
7	rs_7_32588090	32588090	G	A	PC1	83.7223	Intergenic;
7	rs_7_32596858	32596858	A	C	PC1	83.7223	Intergenic;
7	rs_7_32600513	32600513	A	G	PC1	83.7223	Intergenic;
7	rs_7_32600883	32600883	C	A	PC1	83.7223	Intergenic;
7	rs_7_32607895	32607895	T	C	PC1	83.7223	Intergenic;
7	rs_7_32618795	32618795	A	T	PC1	83.7223	Intergenic;
7	rs_7_32622778	32622778	G	A	PC1	83.7223	Intergenic;
7	rs_7_32622929	32622929	A	G	PC1	83.7223	Intergenic;
7	rs_7_32635134	32635134	A	G	PC1	83.7223	Intergenic;
7	rs_7_32638490	32638490	C	G	PC1	83.7223	Intergenic;
7	rs_7_32640726	32640726	C	T	PC1	83.7223	Intergenic;
7	rs_7_32646788	32646788	G	A	PC1	83.7223	Intergenic;
7	rs_7_32648101	32648101	T	G	PC1	83.7223	Intergenic;

7	rs_7_32652501	32652501	G	A	PC1	83.7223	Intergenic;
7	rs_7_32654966	32654966	T	C	PC1	83.7223	Intergenic;
7	rs_7_32670379	32670379	C	T	PC1	83.7223	Intergenic;
7	rs_7_32676318	32676318	T	C	PC1	83.7223	Intergenic;
7	rs_7_32691018	32691018	T	C	PC1	83.7223	Intergenic;
7	rs_7_32693159	32693159	C	G	PC1	83.7223	Intergenic;
7	rs_7_32693482	32693482	C	T	PC1	83.7223	Intergenic;
7	rs_7_32696581	32696581	C	T	PC1	83.7223	Intergenic;
7	rs_7_32700539	32700539	T	A	PC1	83.7223	Intergenic;
7	rs_7_32703159	32703159	G	A	PC1	83.7223	Intergenic;
7	rs_7_32711828	32711828	G	C	PC1	83.7223	Intergenic;
7	rs_7_32712765	32712765	C	T	PC1	83.7223	Intergenic;
7	rs_7_32718265	32718265	T	C	PC1	83.7223	Intergenic;
7	rs_7_32720758	32720758	G	A	PC1	83.7223	Intergenic;
7	rs_7_32726812	32726812	A	G	PC1	83.7223	DPH6;upstream;
7	rs_7_32726853	32726853	T	C	PC1	83.7223	DPH6;upstream;
7	rs_7_32728792	32728792	A	G	PC1	83.7223	DPH6;upstream;
7	rs_7_32735225	32735225	C	G	PC1	83.7223	Intron;DPH6;
7	rs_7_32737394	32737394	A	G	PC1	83.7223	Intron;DPH6;
7	rs_7_32737397	32737397	A	G	PC1	83.7223	Intron;DPH6;
7	rs_7_32737889	32737889	C	T	PC1	83.7223	Intron;DPH6;
7	rs_7_32744880	32744880	G	A	PC1	83.7223	Intron;DPH6;
7	rs_7_32748295	32748295	A	G	PC1	83.7223	Intron;DPH6;
7	rs_7_32751204	32751204	A	G	PC1	83.7223	Intron;DPH6;
7	rs_7_32755110	32755110	A	G	PC1	83.7223	Intron;DPH6;
7	rs_7_32773136	32773136	T	G	PC1	83.7223	Intron;DPH6;
7	rs_7_32783707	32783707	C	T	PC1	83.7223	Intron;DPH6;
7	rs_7_32786510	32786510	C	T	PC1	83.7223	Intron;DPH6;
7	rs_7_32788388	32788388	G	A	PC1	83.7223	Intron;DPH6;
7	rs_7_32794844	32794844	T	C	PC1	83.7223	Intron;DPH6;
7	rs_7_32796044	32796044	T	A	PC1	83.7223	Intron;DPH6;
7	rs_7_32799392	32799392	G	A	PC1	83.7223	Intron;DPH6;
7	rs_7_32802236	32802236	A	G	PC1	83.7223	Intron;DPH6;
7	rs_7_32809027	32809027	A	T	PC1	83.7223	Intron;DPH6;
7	rs_7_32814416	32814416	T	A	PC1	83.7223	Intron;DPH6;
7	rs_7_32820810	32820810	A	T	PC1	83.7223	Intron;DPH6;
7	rs_7_32822964	32822964	C	T	PC1	83.7223	Intron;DPH6;
7	rs_7_32839183	32839183	T	G	PC1	83.7223	Intron;DPH6;
7	rs_7_32841761	32841761	C	A	PC1	83.7223	Intron;DPH6;
7	rs_7_32848132	32848132	G	A	PC1	83.7223	Intron;DPH6;
7	rs_7_32849262	32849262	T	C	PC1	83.7223	Intron;DPH6;
7	rs_7_32864251	32864251	C	T	PC1	83.7223	Intron;DPH6;
7	rs_7_32877369	32877369	C	T	PC1	83.7223	Intron;DPH6;
7	rs_7_32880838	32880838	C	T	PC1	83.7223	Intron;DPH6;
7	rs_7_32881286	32881286	A	G	PC1	83.7223	Intron;DPH6;
7	rs_7_32885690	32885690	T	C	PC1	83.7223	Intron;DPH6;
7	rs_7_33065361	33065361	T	C	PC1	83.7223	Intergenic;
7	rs_7_33084882	33084882	A	T	PC1	83.7223	Intergenic;
7	rs_7_33099555	33099555	A	G	PC1	83.7223	Intergenic;
7	rs_7_33111487	33111487	G	A	PC1	83.7223	Intergenic;
7	rs_7_33249628	33249628	A	G	PC1	83.7223	Intergenic;
7	rs_7_33267247	33267247	G	A	PC1	83.7223	Intergenic;
7	rs_7_33268914	33268914	A	T	PC1	83.7223	Intergenic;
7	rs_7_33273266	33273266	T	C	PC1	83.7223	Intergenic;
7	rs_7_33283102	33283102	T	C	PC1	83.7223	Intergenic;
7	rs_7_33290807	33290807	T	C	PC1	83.7223	Intergenic;
7	rs_7_33299045	33299045	T	C	PC1	83.7223	Intergenic;
7	rs_7_33303868	33303868	T	A	PC1	83.7223	Intergenic;
7	rs_7_33315713	33315713	A	C	PC1	83.7223	Intergenic;
7	rs_7_33316190	33316190	T	C	PC1	83.7223	Intergenic;
7	rs_7_33316191	33316191	G	A	PC1	83.7223	Intergenic;
7	rs_7_33317797	33317797	A	G	PC1	83.7223	Intergenic;
7	rs_7_33319788	33319788	C	T	PC1	83.7223	Intergenic;
7	rs_7_33341514	33341514	T	A	PC1	83.7223	Intergenic;
7	rs_7_33342972	33342972	C	A	PC1	83.7223	Intergenic;
7	rs_7_33344704	33344704	T	A	PC1	83.7223	Intergenic;
7	rs_7_33359984	33359984	A	G	PC1	83.7223	Intergenic;
7	rs_7_33364540	33364540	A	G	PC1	83.7223	Intergenic;
7	rs_7_33377174	33377174	A	G	PC1	83.7223	Intergenic;
7	rs_7_33377703	33377703	A	G	PC1	83.7223	Intergenic;
7	rs_7_33379575	33379575	C	A	PC1	83.7223	Intergenic;
7	rs_7_33388026	33388026	A	G	PC1	83.7223	Intergenic;
7	rs_7_33400884	33400884	C	A	PC1	83.7223	Intergenic;
7	rs_7_33401605	33401605	G	T	PC1	83.7223	Intergenic;
7	rs_7_33407298	33407298	C	A	PC1	83.7223	Intergenic;
7	rs_7_33412105	33412105	C	A	PC1	83.7223	Intergenic;
7	rs_7_33418849	33418849	A	T	PC1	83.7223	Intergenic;
7	rs_7_33430588	33430588	T	C	PC1	83.7223	Intergenic;
7	rs_7_33432171	33432171	A	G	PC1	83.7223	Intergenic;
7	rs_7_33435137	33435137	T	C	PC1	83.7223	Intergenic;
7	rs_7_33437921	33437921	T	C	PC1	83.7223	Intergenic;
7	rs_7_33438167	33438167	C	A	PC1	83.7223	Intergenic;
7	rs_7_33457294	33457294	A	G	PC1	83.7223	Intergenic;
7	rs_7_33472356	33472356	G	A	PC1	83.7223	Intergenic;
7	rs_7_33472530	33472530	A	G	PC1	83.7223	Intergenic;
7	rs_7_33474404	33474404	C	T	PC1	83.7223	Intergenic;
7	rs_7_33486921	33486921	A	G	PC1	83.7223	Intergenic;
7	rs_7_33490435	33490435	A	G	PC1	83.7223	Intergenic;

7	rs_7_33491269	33491269	T	C	PC1	83.7223	Intergenic;
7	rs_7_33497419	33497419	T	G	PC1	83.7223	Intergenic;
7	rs_7_33499014	33499014	A	C	PC1	83.7223	Intergenic;
7	rs_7_33500926	33500926	A	G	PC1	83.7223	Intergenic;
7	rs_7_33502579	33502579	T	C	PC1	83.7223	Intergenic;
7	rs_7_33512950	33512950	A	C	PC1	83.7223	Intergenic;
7	rs_7_33515708	33515708	C	T	PC1	83.7223	Intergenic;
7	rs_7_33516288	33516288	G	A	PC1	83.7223	Intergenic;
7	rs_7_33522240	33522240	G	A	PC1	83.7223	Intergenic;
7	rs_7_33522519	33522519	A	T	PC1	83.7223	Intergenic;
7	rs_7_33529789	33529789	T	C	PC1	83.7223	Intergenic;
7	rs_7_33544370	33544370	T	C	PC1	83.7223	Intergenic;
7	rs_7_33547902	33547902	A	T	PC1	83.7223	Intergenic;
7	rs_7_33549999	33549999	G	A	PC1	83.7223	Intergenic;
7	rs_7_33553105	33553105	C	T	PC1	83.7223	Intergenic;
7	rs_7_33591225	33591225	G	A	PC1	83.7223	Intergenic;
7	rs_7_33601668	33601668	G	A	PC1	83.7223	Intergenic;
7	rs_7_33604582	33604582	T	G	PC1	83.7223	Intergenic;
7	rs_7_33609178	33609178	C	A	PC1	83.7223	Intergenic;
7	rs_7_33609184	33609184	T	C	PC1	83.7223	Intergenic;
7	rs_7_33614414	33614414	T	C	PC1	83.7223	Intergenic;
7	rs_7_33629450	33629450	G	A	PC1	83.7223	Intergenic;
7	rs_7_33638036	33638036	T	A	PC1	83.7223	Intergenic;
7	rs_7_33638922	33638922	C	G	PC1	83.7223	Intergenic;
7	rs_7_33648655	33648655	A	G	PC1	83.7223	Intergenic;
7	rs_7_33657200	33657200	G	A	PC1	83.7223	Intergenic;
7	rs_7_33661119	33661119	A	C	PC1	83.7223	Intergenic;
7	rs_7_33662791	33662791	G	A	PC1	83.7223	Intergenic;
7	rs_7_33666331	33666331	A	G	PC1	83.7223	Intergenic;
7	rs_7_33679669	33679669	T	A	PC1	83.7223	Intergenic;
7	rs_7_33687067	33687067	A	T	PC1	83.7223	Intergenic;
7	rs_7_33695702	33695702	C	T	PC1	83.7223	Intergenic;
7	rs_7_33760673	33760673	T	C	PC1	83.7223	Intron;NOVA1;
7	rs_7_33761021	33761021	C	T	PC1	83.7223	Intron;NOVA1;
7	rs_7_33762084	33762084	T	C	PC1	83.7223	Intron;NOVA1;
7	rs_7_33769513	33769513	C	G	PC1	83.7223	Intron;NOVA1;
7	rs_7_33772425	33772425	A	G	PC1	83.7223	Intron;NOVA1;
7	rs_7_33781259	33781259	T	C	PC1	83.7223	Intron;NOVA1;
7	rs_7_33781533	33781533	C	T	PC1	83.7223	Intron;NOVA1;
7	rs_7_33786560	33786560	C	T	PC1	83.7223	Intron;NOVA1;
7	rs_7_33800151	33800151	T	C	PC1	83.7223	Intron;NOVA1;
7	rs_7_33803680	33803680	G	T	PC1	83.7223	Intron;NOVA1;
7	rs_7_33812438	33812438	A	G	PC1	83.7223	Intron;NOVA1;
7	rs_7_33817547	33817547	T	C	PC1	83.7223	Intron;NOVA1;
7	rs_7_33886202	33886202	T	C	PC1	83.7223	Intergenic;
7	rs_7_33890059	33890059	T	C	PC1	83.7223	Intergenic;
7	rs_7_33895698	33895698	C	T	PC1	83.7223	Intergenic;
7	rs_7_33895775	33895775	T	G	PC1	83.7223	Intergenic;
7	rs_7_33899475	33899475	G	A	PC1	83.7223	Intergenic;
7	rs_7_33911882	33911882	G	A	PC1	83.7223	Intergenic;
7	rs_7_33929551	33929551	A	G	PC1	83.7223	Intergenic;
7	rs_7_33954394	33954394	G	T	PC1	83.7223	Intergenic;
7	rs_7_33954685	33954685	T	C	PC1	83.7223	Intergenic;
7	rs_7_33954693	33954693	T	G	PC1	83.7223	Intergenic;
7	rs_7_33965748	33965748	T	C	PC1	83.7223	Intergenic;
7	rs_7_33985876	33985876	T	C	PC1	83.7223	Intergenic;
7	rs_7_33987224	33987224	C	T	PC1	83.7223	Intergenic;
7	rs_7_34007487	34007487	G	C	PC1	83.7223	Intergenic;
7	rs_7_34486534	34486534	T	C	PC1	83.7223	Intergenic;
7	rs_7_35738373	35738373	G	A	PC1	83.7223	Intergenic;
7	rs_7_35744626	35744626	A	G	PC1	83.7223	Intergenic;
7	rs_7_35747019	35747019	A	G	PC1	83.7223	Intergenic;
7	rs_7_35754521	35754521	G	A	PC1	83.7223	Intergenic;
7	rs_7_35754617	35754617	G	A	PC1	83.7223	Intergenic;
7	rs_7_35756378	35756378	A	T	PC1	83.7223	Intergenic;
7	rs_7_35757068	35757068	C	A	PC1	83.7223	Intergenic;
7	rs_7_35758696	35758696	C	T	PC1	83.7223	Intergenic;
7	rs_7_35759068	35759068	C	A	PC1	83.7223	Intergenic;
7	rs_7_35760656	35760656	G	C	PC1	83.7223	Predicted;downstream;
7	rs_7_35763722	35763722	T	G	PC1	83.7223	Predicted;downstream;
7	rs_7_35764918	35764918	A	G	PC1	83.7223	Predicted;downstream;
7	rs_7_35766116	35766116	T	C	PC1	83.7223	Predicted;downstream;
7	rs_7_35768946	35768946	C	G	PC1	83.7223	Intergenic;
7	rs_7_35769711	35769711	C	G	PC1	83.7223	Intergenic;
7	rs_7_35770720	35770720	A	G	PC1	83.7223	Intron;Predicted;
7	rs_7_35772267	35772267	C	T	PC1	83.7223	Intron;Predicted;
7	rs_7_35775515	35775515	C	A	PC1	83.7223	Intron;Predicted;
7	rs_7_35779509	35779509	G	A	PC1	83.7223	Intergenic;
7	rs_7_35782212	35782212	C	T	PC1	83.7223	Predicted;upstream;
7	rs_7_35805926	35805926	G	A	PC1	83.7223	Intergenic;
7	rs_7_35843931	35843931	G	T	PC1	83.7223	Intron;AKAP6;
7	rs_7_35850108	35850108	T	C	PC1	83.7223	Intron;AKAP6;
7	rs_7_35851388	35851388	C	T	PC1	83.7223	Intron;AKAP6;
7	rs_7_35852174	35852174	A	G	PC1	83.7223	Intron;AKAP6;
7	rs_7_35852784	35852784	A	G	PC1	83.7223	Intron;AKAP6;
7	rs_7_35854488	35854488	G	A	PC1	83.7223	Intron;AKAP6;
7	rs_7_35855772	35855772	T	G	PC1	83.7223	Intron;AKAP6;
7	rs_7_35855883	35855883	G	C	PC1	83.7223	Intron;AKAP6;

7	rs_7_35856290	35856290	C	G	PC1	83.7223	Intron;AKAP6;
7	rs_7_35857458	35857458	G	A	PC1	83.7223	Intron;AKAP6;
7	rs_7_35890042	35890042	A	G	PC1	83.7223	Intron;AKAP6;
7	rs_7_35890631	35890631	T	G	PC1	83.7223	Intron;AKAP6;
7	rs_7_35891955	35891955	A	G	PC1	83.7223	Intron;AKAP6;
7	rs_7_35895124	35895124	A	C	PC1	83.7223	Intron;AKAP6;
7	rs_7_35908581	35908581	C	T	PC1	83.7223	Intron;AKAP6;
7	rs_7_35920276	35920276	A	G	PC1	83.7223	Intron;AKAP6;
7	rs_7_35921872	35921872	A	G	PC1	83.7223	Intron;AKAP6;
7	rs_7_35923451	35923451	A	C	PC1	83.7223	Intron;AKAP6;
7	rs_7_35924564	35924564	A	C	PC1	83.7223	Intron;AKAP6;
7	rs_7_35926646	35926646	A	G	PC1	83.7223	Intron;AKAP6;
7	rs_7_35930644	35930644	A	G	PC1	83.7223	Intron;AKAP6;
7	rs_7_35931049	35931049	C	A	PC1	83.7223	Intron;AKAP6;
7	rs_7_35931607	35931607	T	C	PC1	83.7223	Intron;AKAP6;
7	rs_7_35933713	35933713	C	T	PC1	83.7223	Intron;AKAP6;
7	rs_7_35936385	35936385	T	G	PC1	83.7223	Intron;AKAP6;
7	rs_7_35936477	35936477	T	C	PC1	83.7223	Intron;AKAP6;
7	rs_7_35937197	35937197	C	T	PC1	83.7223	Intron;AKAP6;
7	rs_7_35937660	35937660	T	C	PC1	83.7223	Intron;AKAP6;
7	rs_7_35939100	35939100	C	T	PC1	83.7223	Intron;AKAP6;
7	rs_7_35939723	35939723	G	A	PC1	83.7223	Intron;AKAP6;
7	rs_7_35942006	35942006	G	A	PC1	83.7223	Intron;AKAP6;
7	rs_7_35945382	35945382	C	T	PC1	83.7223	Intron;AKAP6;
7	rs_7_35946039	35946039	T	C	PC1	83.7223	Intron;AKAP6;
7	rs_7_35953029	35953029	G	T	PC1	83.7223	Intron;AKAP6;
7	rs_7_35955954	35955954	G	T	PC1	83.7223	Intron;AKAP6;
7	rs_7_36003989	36003989	C	T	PC1	83.7223	Intron;AKAP6;
7	rs_7_36004478	36004478	G	A	PC1	83.7223	Intron;AKAP6;
7	rs_7_36006465	36006465	C	A	PC1	83.7223	Intron;AKAP6;
7	rs_7_36010860	36010860	G	A	PC1	83.7223	Intron;AKAP6;
7	rs_7_36019839	36019839	G	A	PC1	83.7223	Intron;AKAP6;
7	rs_7_36028945	36028945	T	A	PC1	83.7223	Intron;AKAP6;
7	rs_7_36040777	36040777	C	T	PC1	83.7223	Intron;AKAP6;
7	rs_7_36042125	36042125	A	G	PC1	83.7223	Intron;AKAP6;
7	rs_7_36043713	36043713	G	T	PC1	83.7223	Intron;AKAP6;
7	rs_7_36048298	36048298	G	C	PC1	83.7223	Exon;AKAP6;AKAP6;+;1;CGA;Nonsynonymous;Arg;Pro;
7	rs_7_36048361	36048361	A	G	PC1	83.7223	Exon;AKAP6;AKAP6;+;1;AAG;Nonsynonymous;Lys;Arg;
7	rs_7_36056451	36056451	G	A	PC1	83.7223	AKAP6;downstream;
7	rs_7_36075484	36075484	T	G	PC1	83.7223	Intergenic;
7	rs_7_36079713	36079713	C	T	PC1	83.7223	Intergenic;
7	rs_7_36081383	36081383	T	C	PC1	83.7223	Intergenic;
7	rs_7_36082647	36082647	C	A	PC1	83.7223	Intergenic;
7	rs_7_36090099	36090099	A	G	PC1	83.7223	Intergenic;
7	rs_7_36098604	36098604	C	T	PC1	83.7223	Intergenic;
7	rs_7_36100276	36100276	A	G	PC1	83.7223	Intergenic;
7	rs_7_36111639	36111639	C	T	PC1	83.7223	Predicted;upstream;
7	rs_7_36118327	36118327	T	A	PC1	83.7223	Intergenic;
7	rs_7_36119536	36119536	A	T	PC1	83.7223	Intergenic;
7	rs_7_36120640	36120640	T	C	PC1	83.7223	Intergenic;
7	rs_7_36120729	36120729	A	G	PC1	83.7223	Intergenic;
7	rs_7_36120814	36120814	A	G	PC1	83.7223	Intergenic;
7	rs_7_36122567	36122567	A	G	PC1	83.7223	Intergenic;
7	rs_7_36123467	36123467	C	T	PC1	83.7223	Intergenic;
7	rs_7_36123638	36123638	T	C	PC1	83.7223	Intergenic;
7	rs_7_36123790	36123790	C	T	PC1	83.7223	Intergenic;
7	rs_7_36126260	36126260	A	G	PC1	83.7223	Intergenic;
7	rs_7_36130771	36130771	T	C	PC1	83.7223	Intergenic;
7	rs_7_36132282	36132282	T	C	PC1	83.7223	Intergenic;
7	rs_7_36132791	36132791	A	G	PC1	83.7223	Intergenic;
7	rs_7_36133452	36133452	A	T	PC1	83.7223	Intergenic;
7	rs_7_36134460	36134460	G	T	PC1	83.7223	Intergenic;
7	rs_7_36134704	36134704	A	G	PC1	83.7223	Intergenic;
7	rs_7_36136136	36136136	A	G	PC1	83.7223	Predicted;upstream;
7	rs_7_36137192	36137192	A	G	PC1	83.7223	Predicted;upstream;
7	rs_7_36138074	36138074	T	A	PC1	83.7223	Predicted;upstream;
7	rs_7_36138999	36138999	A	G	PC1	83.7223	Predicted;upstream;
7	rs_7_36141333	36141333	A	G	PC1	83.7223	Predicted;upstream;
7	rs_7_36146429	36146429	A	G	PC1	83.7223	Intron;Predicted;
7	rs_7_36146757	36146757	A	G	PC1	83.7223	Intron;Predicted;
7	rs_7_36147853	36147853	C	A	PC1	83.7223	Intron;Predicted;
7	rs_7_36148682	36148682	A	C	PC1	83.7223	Intron;Predicted;
7	rs_7_36148803	36148803	C	A	PC1	83.7223	Intron;Predicted;
7	rs_7_36149666	36149666	A	G	PC1	83.7223	Intron;Predicted;
7	rs_7_36149802	36149802	A	G	PC1	83.7223	Intron;Predicted;
7	rs_7_36152272	36152272	A	C	PC1	83.7223	Intron;Predicted;
7	rs_7_36152329	36152329	G	A	PC1	83.7223	Intron;Predicted;
7	rs_7_36154806	36154806	C	T	PC1	83.7223	Intergenic;
7	rs_7_36155999	36155999	G	A	PC1	83.7223	Predicted;downstream;
7	rs_7_36157275	36157275	C	A	PC1	83.7223	Predicted;downstream;
7	rs_7_36157539	36157539	A	G	PC1	83.7223	Predicted;downstream;
7	rs_7_36157586	36157586	C	T	PC1	83.7223	Predicted;downstream;
7	rs_7_36157767	36157767	A	G	PC1	83.7223	Predicted;downstream;
7	rs_7_36160732	36160732	T	C	PC1	83.7223	Predicted;downstream;
7	rs_7_36160924	36160924	T	C	PC1	83.7223	Predicted;downstream;
7	rs_7_36162233	36162233	C	T	PC1	83.7223	Predicted;downstream;
7	rs_7_36162960	36162960	G	A	PC1	83.7223	Predicted;downstream;
7	rs_7_36163189	36163189	T	C	PC1	83.7223	Predicted;downstream;

7	rs_7_36165036	36165036	A	G	PC1	83.7223	Intergenic;
7	rs_7_36165260	36165260	C	T	PC1	83.7223	Intergenic;
7	rs_7_36165529	36165529	G	A	PC1	83.7223	Intergenic;
7	rs_7_36167837	36167837	C	T	PC1	83.7223	Intergenic;
7	rs_7_36168720	36168720	C	T	PC1	83.7223	Intergenic;
7	rs_7_36169846	36169846	T	G	PC1	83.7223	Intergenic;
7	rs_7_36170030	36170030	A	G	PC1	83.7223	Intergenic;
7	rs_7_36171370	36171370	T	C	PC1	83.7223	Intergenic;
7	rs_7_36171371	36171371	G	A	PC1	83.7223	Intergenic;
7	rs_7_36171610	36171610	G	A	PC1	83.7223	Intergenic;
7	rs_7_36171699	36171699	A	G	PC1	83.7223	Intergenic;
7	rs_7_36175469	36175469	C	G	PC1	83.7223	Intergenic;
7	rs_7_36175851	36175851	G	A	PC1	83.7223	Intergenic;
7	rs_7_36178418	36178418	C	T	PC1	83.7223	Intergenic;
7	rs_7_36178835	36178835	G	A	PC1	83.7223	Intergenic;
7	rs_7_36179494	36179494	A	G	PC1	83.7223	Intergenic;
7	rs_7_36179610	36179610	C	A	PC1	83.7223	Intergenic;
7	rs_7_36180579	36180579	A	G	PC1	83.7223	Intergenic;
7	rs_7_36180624	36180624	G	C	PC1	83.7223	Intergenic;
7	rs_7_36180858	36180858	G	A	PC1	83.7223	Intergenic;
7	rs_7_36184200	36184200	C	G	PC1	83.7223	Intergenic;
7	rs_7_36186862	36186862	T	C	PC1	83.7223	Intergenic;
7	rs_7_36187478	36187478	A	G	PC1	83.7223	Intergenic;
7	rs_7_36187576	36187576	C	T	PC1	83.7223	Intergenic;
7	rs_7_36188023	36188023	A	G	PC1	83.7223	Intergenic;
7	rs_7_36188646	36188646	A	G	PC1	83.7223	Intergenic;
7	rs_7_36189049	36189049	T	A	PC1	83.7223	Intergenic;
7	rs_7_36189300	36189300	G	A	PC1	83.7223	Intergenic;
7	rs_7_36191625	36191625	A	G	PC1	83.7223	Intergenic;
7	rs_7_36192210	36192210	T	G	PC1	83.7223	Intergenic;
7	rs_7_36193352	36193352	T	C	PC1	83.7223	Intergenic;
7	rs_7_36193396	36193396	C	A	PC1	83.7223	Intergenic;
7	rs_7_36193442	36193442	C	A	PC1	83.7223	Intergenic;
7	rs_7_36194924	36194924	C	A	PC1	83.7223	Intergenic;
7	rs_7_36198124	36198124	A	G	PC1	83.7223	Intergenic;
7	rs_7_36198425	36198425	T	G	PC1	83.7223	Intergenic;
7	rs_7_36201807	36201807	A	T	PC1	83.7223	Intergenic;
7	rs_7_36204887	36204887	G	T	PC1	83.7223	Intergenic;
7	rs_7_36207233	36207233	C	T	PC1	83.7223	Intergenic;
7	rs_7_36212018	36212018	G	C	PC1	83.7223	Intergenic;
7	rs_7_36212969	36212969	T	A	PC1	83.7223	Intergenic;
7	rs_7_36213536	36213536	C	T	PC1	83.7223	Intergenic;
7	rs_7_36215100	36215100	T	A	PC1	83.7223	Intergenic;
7	rs_7_36218035	36218035	C	T	PC1	83.7223	Intergenic;
7	rs_7_36225059	36225059	A	G	PC1	83.7223	Intergenic;
7	rs_7_36225231	36225231	T	C	PC1	83.7223	Intergenic;
7	rs_7_36225294	36225294	T	A	PC1	83.7223	Intergenic;
7	rs_7_36226747	36226747	A	G	PC1	83.7223	Intergenic;
7	rs_7_36227361	36227361	A	G	PC1	83.7223	Intergenic;
7	rs_7_36227572	36227572	T	C	PC1	83.7223	Intergenic;
7	rs_7_36228386	36228386	A	G	PC1	83.7223	Intergenic;
7	rs_7_36228706	36228706	T	C	PC1	83.7223	Intergenic;
7	rs_7_36229163	36229163	C	G	PC1	83.7223	Intergenic;
7	rs_7_36230569	36230569	G	A	PC1	83.7223	Intergenic;
7	rs_7_36231622	36231622	G	A	PC1	83.7223	Intergenic;
7	rs_7_36235266	36235266	A	G	PC1	83.7223	Intergenic;
7	rs_7_36240371	36240371	C	T	PC1	83.7223	Intergenic;
7	rs_7_36242055	36242055	G	T	PC1	83.7223	Intergenic;
7	rs_7_36242468	36242468	G	A	PC1	83.7223	Intergenic;
7	rs_7_36244870	36244870	C	T	PC1	83.7223	Intergenic;
7	rs_7_36247839	36247839	G	A	PC1	83.7223	Intergenic;
7	rs_7_36247938	36247938	G	A	PC1	83.7223	Intergenic;
7	rs_7_36249967	36249967	A	G	PC1	83.7223	Intergenic;
7	rs_7_36250185	36250185	G	A	PC1	83.7223	Intergenic;
7	rs_7_36250436	36250436	T	G	PC1	83.7223	Intergenic;
7	rs_7_36250500	36250500	T	C	PC1	83.7223	Intergenic;
7	rs_7_36250858	36250858	C	T	PC1	83.7223	Intergenic;
7	rs_7_36250971	36250971	G	A	PC1	83.7223	Intergenic;
7	rs_7_36252881	36252881	G	C	PC1	83.7223	Intergenic;
7	rs_7_36255333	36255333	C	T	PC1	83.7223	Intergenic;
7	rs_7_36255510	36255510	A	G	PC1	83.7223	Intergenic;
7	rs_7_36260224	36260224	G	T	PC1	83.7223	Intergenic;
7	rs_7_36260304	36260304	G	A	PC1	83.7223	Intergenic;
7	rs_7_36260582	36260582	A	G	PC1	83.7223	Intergenic;
7	rs_7_36262425	36262425	G	C	PC1	83.7223	Intergenic;
7	rs_7_36263311	36263311	A	T	PC1	83.7223	Intergenic;
7	rs_7_36263705	36263705	G	A	PC1	83.7223	Intergenic;
7	rs_7_36264119	36264119	G	T	PC1	83.7223	Intergenic;
7	rs_7_36265058	36265058	T	G	PC1	83.7223	Intergenic;
7	rs_7_36265512	36265512	G	A	PC1	83.7223	Intergenic;
7	rs_7_36266560	36266560	A	G	PC1	83.7223	Intergenic;
7	rs_7_36267738	36267738	A	C	PC1	83.7223	Intergenic;
7	rs_7_36272629	36272629	G	A	PC1	83.7223	Intergenic;
7	rs_7_36274298	36274298	A	G	PC1	83.7223	Intergenic;
7	rs_7_36281191	36281191	A	G	PC1	83.7223	NPAS3;upstream;
7	rs_7_36293987	36293987	T	C	PC1	83.7223	Intron;NPAS3;
7	rs_7_36297072	36297072	T	C	PC1	83.7223	Intron;NPAS3;
7	rs_7_36297299	36297299	G	A	PC1	83.7223	Intron;NPAS3;

7	rs_7_36299130	36299130	C	T	PC1	83.7223	Intron;NPAS3;
7	rs_7_36301817	36301817	A	G	PC1	83.7223	Intron;NPAS3;
7	rs_7_36308743	36308743	T	A	PC1	83.7223	Intron;NPAS3;
7	rs_7_36327588	36327588	T	A	PC1	83.7223	Intron;NPAS3;
7	rs_7_36333930	36333930	T	C	PC1	83.7223	Intron;NPAS3;
7	rs_7_36340553	36340553	C	T	PC1	83.7223	Intron;NPAS3;
7	rs_7_36349714	36349714	A	C	PC1	83.7223	Intron;NPAS3;
7	rs_7_36351943	36351943	G	C	PC1	83.7223	Intron;NPAS3;
7	rs_7_36352326	36352326	C	T	PC1	83.7223	Intron;NPAS3;
7	rs_7_36355681	36355681	A	C	PC1	83.7223	Intron;NPAS3;
7	rs_7_36364122	36364122	C	T	PC1	83.7223	Intron;NPAS3;
7	rs_7_36366808	36366808	G	C	PC1	83.7223	Intron;NPAS3;
7	rs_7_36369436	36369436	A	C	PC1	83.7223	Intron;NPAS3;
7	rs_7_36380623	36380623	A	G	PC1	83.7223	Intron;NPAS3;
7	rs_7_36381567	36381567	C	G	PC1	83.7223	Intron;NPAS3;
7	rs_7_36383145	36383145	T	A	PC1	83.7223	Intron;NPAS3;
7	rs_7_36391491	36391491	G	A	PC1	83.7223	Intron;NPAS3;
7	rs_7_36398078	36398078	A	G	PC1	83.7223	Intron;NPAS3;
7	rs_7_36400053	36400053	C	G	PC1	83.7223	Intron;NPAS3;
7	rs_7_36419184	36419184	A	G	PC1	83.7223	Intron;NPAS3;
7	rs_7_36423841	36423841	G	A	PC1	83.7223	Intron;NPAS3;
7	rs_7_36433762	36433762	T	C	PC1	83.7223	Intron;NPAS3;
7	rs_7_36434082	36434082	A	G	PC1	83.7223	Intron;NPAS3;
7	rs_7_36435039	36435039	T	C	PC1	83.7223	Intron;NPAS3;
7	rs_7_36435201	36435201	C	G	PC1	83.7223	Intron;NPAS3;
7	rs_7_36439193	36439193	G	A	PC1	83.7223	Intron;NPAS3;
7	rs_7_36439653	36439653	T	C	PC1	83.7223	Intron;NPAS3;
7	rs_7_36440235	36440235	A	G	PC1	77.99387	Intron;NPAS3;
7	rs_7_36447341	36447341	C	T	PC1	83.7223	Intron;NPAS3;
7	rs_7_36447476	36447476	A	G	PC1	83.7223	Intron;NPAS3;
7	rs_7_36447807	36447807	G	A	PC1	83.7223	Intron;NPAS3;
7	rs_7_36449503	36449503	G	A	PC1	83.7223	Intron;NPAS3;
7	rs_7_36453604	36453604	G	A	PC1	83.7223	Intron;NPAS3;
7	rs_7_36455254	36455254	G	A	PC1	83.7223	Intron;NPAS3;
7	rs_7_36455313	36455313	T	C	PC1	83.7223	Intron;NPAS3;
7	rs_7_36455521	36455521	C	T	PC1	83.7223	Intron;NPAS3;
7	rs_7_36456156	36456156	C	G	PC1	83.7223	Intron;NPAS3;
7	rs_7_36457238	36457238	A	G	PC1	83.7223	Intron;NPAS3;
7	rs_7_36457349	36457349	T	G	PC1	83.7223	Intron;NPAS3;
7	rs_7_36457666	36457666	T	A	PC1	83.7223	Intron;NPAS3;
7	rs_7_36457717	36457717	T	C	PC1	83.7223	Intron;NPAS3;
7	rs_7_36458577	36458577	A	G	PC1	83.7223	Intron;NPAS3;
7	rs_7_36459019	36459019	C	T	PC1	83.7223	Intron;NPAS3;
7	rs_7_36459534	36459534	T	C	PC1	83.7223	Intron;NPAS3;
7	rs_7_36462430	36462430	G	A	PC1	83.7223	Intron;NPAS3;
7	rs_7_36462876	36462876	A	G	PC1	83.7223	Intron;NPAS3;
7	rs_7_36463554	36463554	G	T	PC1	83.7223	Intron;NPAS3;
7	rs_7_36466746	36466746	G	A	PC1	83.7223	Intron;NPAS3;
7	rs_7_36467909	36467909	C	T	PC1	83.7223	Intron;NPAS3;
7	rs_7_36471992	36471992	G	A	PC1	83.7223	Intron;NPAS3;
7	rs_7_36472014	36472014	G	A	PC1	83.7223	Intron;NPAS3;
7	rs_7_36472239	36472239	A	G	PC1	83.7223	Intron;NPAS3;
7	rs_7_36478456	36478456	T	C	PC1	83.7223	Intron;NPAS3;
7	rs_7_36481186	36481186	T	C	PC1	83.7223	Intron;NPAS3;
7	rs_7_36482662	36482662	A	G	PC1	83.7223	Intron;NPAS3;
7	rs_7_36484697	36484697	G	A	PC1	83.7223	Intron;NPAS3;
7	rs_7_36487681	36487681	G	A	PC1	83.7223	Intron;NPAS3;
7	rs_7_36487936	36487936	C	T	PC1	83.7223	Intron;NPAS3;
7	rs_7_36491989	36491989	G	A	PC1	83.7223	Intron;NPAS3;
7	rs_7_36492628	36492628	A	G	PC1	83.7223	Intron;NPAS3;
7	rs_7_36493641	36493641	C	T	PC1	83.7223	Intron;NPAS3;
7	rs_7_36496332	36496332	G	A	PC1	83.7223	Intron;NPAS3;
7	rs_7_36497573	36497573	A	G	PC1	83.7223	Intron;NPAS3;
7	rs_7_36500494	36500494	T	G	PC1	83.7223	Intron;NPAS3;
7	rs_7_36509036	36509036	G	T	PC1	83.7223	Intron;NPAS3;
7	rs_7_36509100	36509100	T	C	PC1	83.7223	Intron;NPAS3;
7	rs_7_36514047	36514047	C	G	PC1	83.7223	Intron;NPAS3;
7	rs_7_36514164	36514164	A	G	PC1	83.7223	Intron;NPAS3;
7	rs_7_36523342	36523342	G	A	PC1	83.7223	Intron;NPAS3;
7	rs_7_36525471	36525471	G	A	PC1	83.7223	Intron;NPAS3;
7	rs_7_36527361	36527361	A	G	PC1	83.7223	Intron;NPAS3;
7	rs_7_36528503	36528503	A	C	PC1	83.7223	Intron;NPAS3;
7	rs_7_36528980	36528980	A	G	PC1	83.7223	Intron;NPAS3;
7	rs_7_36532419	36532419	T	A	PC1	83.7223	Intron;NPAS3;
7	rs_7_36534879	36534879	G	C	PC1	83.7223	Intron;NPAS3;
7	rs_7_36537796	36537796	C	G	PC1	83.7223	Intron;NPAS3;
7	rs_7_36541001	36541001	C	T	PC1	83.7223	Intron;NPAS3;
7	rs_7_36541031	36541031	T	C	PC1	83.7223	Intron;NPAS3;
7	rs_7_36545126	36545126	A	T	PC1	83.7223	Intron;NPAS3;
7	rs_7_36552172	36552172	C	T	PC1	83.7223	Intron;NPAS3;
7	rs_7_36558221	36558221	A	G	PC1	83.7223	Intron;NPAS3;
7	rs_7_36560251	36560251	G	A	PC1	83.7223	Intron;NPAS3;
7	rs_7_36561966	36561966	T	A	PC1	83.7223	Intron;NPAS3;
7	rs_7_36563591	36563591	C	T	PC1	83.7223	Intron;NPAS3;
7	rs_7_36563635	36563635	C	G	PC1	83.7223	Intron;NPAS3;
7	rs_7_36565128	36565128	C	A	PC1	83.7223	Intron;NPAS3;
7	rs_7_36570364	36570364	A	G	PC1	83.7223	Intron;NPAS3;
7	rs_7_36573519	36573519	G	A	PC1	83.7223	Intron;NPAS3;

7	rs_7_36576012	36576012	A	G	PC1	83.7223	Intron;NPAS3;
7	rs_7_36577013	36577013	T	A	PC1	83.7223	Intron;NPAS3;
7	rs_7_36578986	36578986	T	C	PC1	83.7223	Intron;NPAS3;
7	rs_7_36579849	36579849	T	C	PC1	83.7223	Intron;NPAS3;
7	rs_7_36581029	36581029	G	A	PC1	83.7223	Intron;NPAS3;
7	rs_7_36587711	36587711	A	T	PC1	83.7223	Intron;NPAS3;
7	rs_7_36588776	36588776	T	C	PC1	83.7223	Intron;NPAS3;
7	rs_7_36594507	36594507	A	T	PC1	83.7223	Intron;NPAS3;
7	rs_7_36596332	36596332	C	T	PC1	83.7223	Intron;NPAS3;
7	rs_7_36598749	36598749	G	A	PC1	83.7223	Intron;NPAS3;
7	rs_7_36601543	36601543	A	G	PC1	83.7223	Intron;NPAS3;
7	rs_7_36607051	36607051	T	G	PC1	83.7223	Intron;NPAS3;
7	rs_7_36611685	36611685	A	G	PC1	83.7223	Intron;NPAS3;
7	rs_7_36612288	36612288	A	C	PC1	83.7223	Intron;NPAS3;
7	rs_7_36612950	36612950	C	T	PC1	83.7223	Intron;NPAS3;
7	rs_7_36615220	36615220	G	A	PC1	83.7223	Intron;NPAS3;
7	rs_7_36617672	36617672	G	C	PC1	83.7223	Intron;NPAS3;
7	rs_7_36622190	36622190	A	G	PC1	83.7223	Intron;NPAS3;
7	rs_7_36622620	36622620	T	A	PC1	83.7223	Intron;NPAS3;
7	rs_7_36624795	36624795	T	C	PC1	83.7223	Intron;NPAS3;
7	rs_7_36628272	36628272	G	C	PC1	83.7223	Intron;NPAS3;
7	rs_7_36632851	36632851	T	C	PC1	83.7223	Intron;NPAS3;
7	rs_7_36643040	36643040	G	A	PC1	83.7223	Intron;NPAS3;
7	rs_7_36643514	36643514	T	A	PC1	83.7223	Intron;NPAS3;
7	rs_7_36643666	36643666	T	C	PC1	83.7223	Intron;NPAS3;
7	rs_7_36654745	36654745	C	G	PC1	83.7223	Intron;NPAS3;
7	rs_7_36667214	36667214	T	C	PC1	83.7223	Intron;NPAS3;
7	rs_7_36683015	36683015	G	A	PC1	83.7223	Intron;NPAS3;
7	rs_7_36701169	36701169	T	C	PC1	83.7223	Intron;NPAS3;
7	rs_7_36713380	36713380	A	T	PC1	83.7223	Intron;NPAS3;
7	rs_7_36715876	36715876	A	C	PC1	83.7223	Intron;NPAS3;
7	rs_7_36718120	36718120	T	C	PC1	83.7223	Intron;NPAS3;
7	rs_7_36720432	36720432	A	G	PC1	83.7223	Intron;NPAS3;
7	rs_7_36733907	36733907	A	G	PC1	83.7223	Intron;EGLN3;
7	rs_7_36736180	36736180	C	A	PC1	83.7223	Intron;EGLN3;
7	rs_7_36738353	36738353	G	A	PC1	83.7223	Exon;EGLN3;EGLN3;-;1;GCC;Nonsynonymous;Ala;Val;
7	rs_7_36750809	36750809	A	G	PC1	83.7223	Intron;EGLN3;
7	rs_7_36751975	36751975	G	C	PC1	83.7223	Intron;EGLN3;
7	rs_7_36767051	36767051	C	T	PC1	83.7223	Intron;EGLN3;
7	rs_7_36768838	36768838	T	C	PC1	83.7223	Intron;EGLN3;
7	rs_7_36777274	36777274	G	A	PC1	83.7223	Intron;EGLN3;
7	rs_7_36780433	36780433	G	A	PC1	83.7223	EGLN3;upstream;
7	rs_7_36782334	36782334	A	G	PC1	83.7223	EGLN3;upstream;
7	rs_7_36785902	36785902	C	T	PC1	83.7223	EGLN3;upstream;
7	rs_7_36786183	36786183	G	A	PC1	83.7223	EGLN3;upstream;
7	rs_7_36793526	36793526	A	G	PC1	83.7223	Intergenic;
7	rs_7_36796942	36796942	A	G	PC1	83.7223	Intergenic;
7	rs_7_36796994	36796994	C	T	PC1	83.7223	Intergenic;
7	rs_7_36801402	36801402	G	A	PC1	83.7223	Intergenic;
7	rs_7_36813645	36813645	T	C	PC1	83.7223	Intergenic;
7	rs_7_36813733	36813733	C	G	PC1	83.7223	Intergenic;
7	rs_7_36815417	36815417	C	G	PC1	83.7223	Intergenic;
7	rs_7_36815762	36815762	T	C	PC1	83.7223	Intergenic;
7	rs_7_36819570	36819570	T	C	PC1	83.7223	Intergenic;
7	rs_7_36820089	36820089	C	T	PC1	83.7223	Intergenic;
7	rs_7_36820845	36820845	A	G	PC1	83.7223	Intergenic;
7	rs_7_36825823	36825823	C	T	PC1	83.7223	Intergenic;
7	rs_7_36827536	36827536	T	C	PC1	83.7223	Intergenic;
7	rs_7_36827688	36827688	T	C	PC1	77.99387	Intergenic;
7	rs_7_36829213	36829213	T	A	PC1	83.7223	Intergenic;
7	rs_7_36831451	36831451	A	G	PC1	83.7223	Intergenic;
7	rs_7_36832343	36832343	C	T	PC1	83.7223	Intergenic;
7	rs_7_36833284	36833284	G	A	PC1	83.7223	Intergenic;
7	rs_7_36833913	36833913	A	G	PC1	83.7223	Intergenic;
7	rs_7_36834722	36834722	G	C	PC1	83.7223	Intergenic;
7	rs_7_36838162	36838162	G	A	PC1	83.7223	Intergenic;
7	rs_7_36838789	36838789	A	G	PC1	83.7223	Intergenic;
7	rs_7_36839610	36839610	T	C	PC1	83.7223	Intergenic;
7	rs_7_36839653	36839653	A	G	PC1	83.7223	Intergenic;
7	rs_7_36843204	36843204	T	A	PC1	83.7223	Intergenic;
7	rs_7_36845514	36845514	G	A	PC1	83.7223	Intergenic;
7	rs_7_36846991	36846991	A	C	PC1	83.7223	Intergenic;
7	rs_7_36923991	36923991	G	A	PC1	83.7223	Intergenic;
7	rs_7_36967597	36967597	A	T	PC1	83.7223	Intron;EAPP;
7	rs_7_36989543	36989543	A	G	PC1	83.7223	Intron;SNX6;
7	rs_7_37185498	37185498	G	A	PC1	83.7223	Intron;PPP2R3C;
7	rs_7_37418907	37418907	C	T	PC1	83.7223	Intron;RALGAPA1;
7	rs_7_37419402	37419402	A	G	PC1	83.7223	Intron;RALGAPA1;
7	rs_7_37419689	37419689	G	A	PC1	83.7223	Intron;RALGAPA1;
7	rs_7_37422282	37422282	T	C	PC1	83.7223	RALGAPA1;upstream;BRMS1L;upstream;
7	rs_7_37426225	37426225	A	C	PC1	83.7223	RALGAPA1;upstream;BRMS1L;upstream;
7	rs_7_37485271	37485271	T	A	PC1	83.7223	Intergenic;
7	rs_7_37511903	37511903	T	A	PC1	83.7223	Intergenic;
7	rs_7_37533336	37533336	C	T	PC1	83.7223	Intergenic;
7	rs_7_37533338	37533338	G	T	PC1	83.7223	Intergenic;
7	rs_7_37533570	37533570	A	G	PC1	83.7223	Intergenic;
7	rs_7_37534565	37534565	G	A	PC1	83.7223	Intergenic;
7	rs_7_37536874	37536874	A	G	PC1	83.7223	Intergenic;

7	rs_7_37537509	37537509	C	T	PC1	83.7223	Intergenic;
7	rs_7_37537796	37537796	G	A	PC1	83.7223	Intergenic;
7	rs_7_37538180	37538180	A	G	PC1	83.7223	Intergenic;
7	rs_7_37538368	37538368	G	A	PC1	83.7223	Intergenic;
7	rs_7_37538629	37538629	G	A	PC1	83.7223	Intergenic;
7	rs_7_37539081	37539081	C	T	PC1	83.7223	Intergenic;
7	rs_7_37541870	37541870	A	C	PC1	83.7223	Intergenic;
7	rs_7_37542172	37542172	A	G	PC1	83.7223	Intergenic;
7	rs_7_37547474	37547474	T	G	PC1	83.7223	Intergenic;
7	rs_7_37548821	37548821	T	C	PC1	83.7223	Intergenic;
7	rs_7_37552948	37552948	G	C	PC1	83.7223	Intergenic;
7	rs_7_37553829	37553829	G	A	PC1	83.7223	Intergenic;
7	rs_7_37554337	37554337	A	G	PC1	83.7223	Intergenic;
7	rs_7_37556426	37556426	C	T	PC1	83.7223	Intergenic;
7	rs_7_37558091	37558091	T	C	PC1	83.7223	Intergenic;
7	rs_7_37558423	37558423	T	G	PC1	83.7223	Intergenic;
7	rs_7_37560921	37560921	T	C	PC1	83.7223	Intergenic;
7	rs_7_37571953	37571953	C	T	PC1	83.7223	Intergenic;
7	rs_7_37577059	37577059	T	C	PC1	83.7223	Intergenic;
7	rs_7_37577093	37577093	C	T	PC1	83.7223	Intergenic;
7	rs_7_37586618	37586618	A	G	PC1	83.7223	Intergenic;
7	rs_7_37586795	37586795	C	G	PC1	83.7223	Intergenic;
7	rs_7_37586819	37586819	A	G	PC1	83.7223	Intergenic;
7	rs_7_37588577	37588577	A	G	PC1	83.7223	Intergenic;
7	rs_7_37596437	37596437	T	C	PC1	83.7223	Intergenic;
7	rs_7_37599662	37599662	G	T	PC1	83.7223	Intergenic;
7	rs_7_37600110	37600110	T	C	PC1	83.7223	MBIP;downstream;
7	rs_7_37600226	37600226	C	T	PC1	83.7223	MBIP;downstream;
7	rs_7_37601334	37601334	G	C	PC1	83.7223	MBIP;downstream;
7	rs_7_37603020	37603020	C	T	PC1	83.7223	MBIP;downstream;
7	rs_7_37604374	37604374	T	C	PC1	83.7223	MBIP;downstream;
7	rs_7_37604410	37604410	G	A	PC1	83.7223	MBIP;downstream;
7	rs_7_37605250	37605250	G	A	PC1	83.7223	MBIP;downstream;
7	rs_7_37605506	37605506	A	G	PC1	83.7223	MBIP;downstream;
7	rs_7_37605852	37605852	T	C	PC1	83.7223	MBIP;downstream;
7	rs_7_37606261	37606261	A	T	PC1	83.7223	MBIP;downstream;
7	rs_7_37606301	37606301	A	C	PC1	83.7223	MBIP;downstream;
7	rs_7_37606362	37606362	C	G	PC1	83.7223	MBIP;downstream;
7	rs_7_37606665	37606665	C	G	PC1	83.7223	MBIP;downstream;
7	rs_7_37606687	37606687	A	G	PC1	83.7223	MBIP;downstream;
7	rs_7_37606772	37606772	C	T	PC1	83.7223	MBIP;downstream;
7	rs_7_37614803	37614803	C	T	PC1	83.7223	Intron;MBIP;
7	rs_7_37615189	37615189	G	A	PC1	83.7223	Intron;MBIP;
7	rs_7_37617863	37617863	T	G	PC1	83.7223	Intron;MBIP;
7	rs_7_37618698	37618698	T	C	PC1	83.7223	Intron;MBIP;
7	rs_7_37618985	37618985	C	A	PC1	83.7223	Intron;MBIP;
7	rs_7_37618987	37618987	C	T	PC1	83.7223	Intron;MBIP;
7	rs_7_37619021	37619021	T	A	PC1	83.7223	Intron;MBIP;
7	rs_7_37619286	37619286	C	T	PC1	83.7223	Intron;MBIP;
7	rs_7_37623165	37623165	G	A	PC1	83.7223	MBIP;upstream;
7	rs_7_37624700	37624700	T	C	PC1	83.7223	MBIP;upstream;
7	rs_7_37624791	37624791	A	G	PC1	83.7223	MBIP;upstream;
7	rs_7_37624802	37624802	C	T	PC1	83.7223	MBIP;upstream;
7	rs_7_37625580	37625580	A	G	PC1	77.99387	MBIP;upstream;
7	rs_7_37627391	37627391	T	G	PC1	83.7223	MBIP;upstream;
7	rs_7_37628131	37628131	A	G	PC1	83.7223	MBIP;upstream;
7	rs_7_37628408	37628408	T	C	PC1	83.7223	MBIP;upstream;
7	rs_7_37630247	37630247	C	T	PC1	83.7223	MBIP;upstream;
7	rs_7_37630663	37630663	G	A	PC1	83.7223	MBIP;upstream;
7	rs_7_37631338	37631338	T	C	PC1	83.7223	MBIP;upstream;
7	rs_7_37631404	37631404	G	A	PC1	83.7223	MBIP;upstream;
7	rs_7_37631791	37631791	T	C	PC1	83.7223	MBIP;upstream;
7	rs_7_37633264	37633264	A	T	PC1	83.7223	Intergenic;
7	rs_7_37633850	37633850	C	T	PC1	83.7223	Intergenic;
7	rs_7_37636224	37636224	A	T	PC1	83.7223	Intergenic;
7	rs_7_37638430	37638430	A	G	PC1	83.7223	Intergenic;
7	rs_7_37642296	37642296	T	C	PC1	83.7223	Intergenic;
7	rs_7_37644969	37644969	C	A	PC1	83.7223	Intergenic;
7	rs_7_37652058	37652058	G	A	PC1	83.7223	Intergenic;
7	rs_7_37652525	37652525	A	G	PC1	83.7223	Intergenic;
7	rs_7_37654518	37654518	C	T	PC1	77.99387	Intergenic;
7	rs_7_37654924	37654924	A	C	PC1	83.7223	Intergenic;
7	rs_7_37655547	37655547	C	T	PC1	83.7223	Intergenic;
7	rs_7_37661311	37661311	T	G	PC1	83.7223	Intergenic;
7	rs_7_37663454	37663454	T	A	PC1	83.7223	Intergenic;
7	rs_7_37667738	37667738	T	C	PC1	83.7223	Intergenic;
7	rs_7_37670316	37670316	A	G	PC1	83.7223	Intergenic;
7	rs_7_37671512	37671512	C	G	PC1	83.7223	Intergenic;
7	rs_7_37673653	37673653	A	G	PC1	83.7223	Intergenic;
7	rs_7_37674601	37674601	A	C	PC1	83.7223	Intergenic;
7	rs_7_37676112	37676112	T	C	PC1	83.7223	Intergenic;
7	rs_7_37676456	37676456	A	G	PC1	83.7223	Intergenic;
7	rs_7_37679421	37679421	C	G	PC1	83.7223	Intergenic;
7	rs_7_37681324	37681324	G	C	PC1	83.7223	Intergenic;
7	rs_7_37681596	37681596	C	T	PC1	83.7223	Intergenic;
7	rs_7_37685504	37685504	T	C	PC1	83.7223	Intergenic;
7	rs_7_37686945	37686945	C	G	PC1	83.7223	Intergenic;
7	rs_7_37687729	37687729	C	T	PC1	83.7223	Intergenic;

7	rs_7_37687960	37687960	T	A	PC1	83.7223		Intergenic;
7	rs_7_37687970	37687970	G	C	PC1	83.7223		Intergenic;
7	rs_7_37689783	37689783	A	C	PC1	83.7223		Intergenic;
7	rs_7_37690155	37690155	A	T	PC1	83.7223		Intergenic;
7	rs_7_37690916	37690916	G	A	PC1	83.7223		Intergenic;
7	rs_7_37691527	37691527	G	A	PC1	83.7223		Intergenic;
7	rs_7_37694393	37694393	C	T	PC1	83.7223		Intergenic;
7	rs_7_37694985	37694985	G	T	PC1	83.7223		Intergenic;
7	rs_7_37695225	37695225	A	G	PC1	83.7223		Intergenic;
7	rs_7_37696068	37696068	T	G	PC1	83.7223		Intergenic;
7	rs_7_37696597	37696597	C	T	PC1	83.7223		NKX2-1;downstream;
7	rs_7_37697321	37697321	G	A	PC1	83.7223		NKX2-1;downstream;
7	rs_7_37697447	37697447	T	C	PC1	83.7223		NKX2-1;downstream;
7	rs_7_37701502	37701502	A	G	PC1	83.7223		NKX2-1;downstream;
7	rs_7_37702803	37702803	C	G	PC1	83.7223		NKX2-1;downstream;
7	rs_7_37703893	37703893	G	A	PC1	83.7223		NKX2-1;downstream;
7	rs_7_37704128	37704128	T	C	PC1	83.7223		NKX2-1;downstream;
7	rs_7_37704258	37704258	A	G	PC1	83.7223		NKX2-1;downstream;
7	rs_7_37704858	37704858	T	C	PC1	83.7223		NKX2-1;downstream;
7	rs_7_37705029	37705029	T	C	PC1	83.7223		NKX2-1;downstream;
7	rs_7_37706020	37706020	A	G	PC1	83.7223		NKX2-1;downstream;
7	rs_7_37706696	37706696	C	T	PC1	83.7223		Intron;NKX2-1;
7	rs_7_37710013	37710013	C	T	PC1	83.7223		Intron;NKX2-1;
7	rs_7_37710697	37710697	A	C	PC1	77.99387		Intron;NKX2-1;
7	rs_7_37714027	37714027	T	C	PC1	83.7223		Intron;NKX2-1;
7	rs_7_37714033	37714033	G	C	PC1	83.7223		Intron;NKX2-1;
7	rs_7_37714360	37714360	T	G	PC1	83.7223		Intron;NKX2-1;
7	rs_7_37719861	37719861	T	C	PC1	83.7223		Intron;NKX2-1;
7	rs_7_37724562	37724562	A	C	PC1	83.7223		NKX2-1;upstream;
7	rs_7_37727278	37727278	G	A	PC1	83.7223		NKX2-1;upstream;
7	rs_7_37727666	37727666	G	A	PC1	83.7223		NKX2-1;upstream;
7	rs_7_37733024	37733024	A	C	PC1	83.7223		NKX2-1;upstream;
7	rs_7_37733169	37733169	C	T	PC1	83.7223		NKX2-1;upstream;
7	rs_7_37733945	37733945	G	A	PC1	83.7223		Intergenic;
7	rs_7_37734249	37734249	C	A	PC1	83.7223		Intergenic;
7	rs_7_37735169	37735169	G	A	PC1	83.7223		Intergenic;
7	rs_7_37736156	37736156	A	C	PC1	83.7223		Intergenic;
7	rs_7_37736661	37736661	G	C	PC1	83.7223		Intergenic;
7	rs_7_37737586	37737586	G	A	PC1	83.7223		Intergenic;
7	rs_7_37738405	37738405	T	C	PC1	83.7223		Intergenic;
7	rs_7_37739279	37739279	A	C	PC1	83.7223		Intergenic;
7	rs_7_37739817	37739817	T	C	PC1	83.7223		Intergenic;
7	rs_7_37739917	37739917	T	C	PC1	83.7223		Intergenic;
7	rs_7_37740388	37740388	G	A	PC1	83.7223		Intergenic;
7	rs_7_37742505	37742505	G	A	PC1	83.7223		Intergenic;
7	rs_7_37742581	37742581	T	C	PC1	83.7223		Intergenic;
7	rs_7_37742702	37742702	T	C	PC1	83.7223		Intergenic;
7	rs_7_37742713	37742713	G	A	PC1	83.7223		Intergenic;
7	rs_7_37743270	37743270	T	C	PC1	83.7223		Intergenic;
7	rs_7_37744589	37744589	G	A	PC1	83.7223		Intergenic;
7	rs_7_37751960	37751960	A	G	PC1	83.7223		Intergenic;
7	rs_7_37752075	37752075	A	G	PC1	83.7223		Intergenic;
7	rs_7_37752393	37752393	T	C	PC1	83.7223		Intergenic;
7	rs_7_37756196	37756196	G	C	PC1	83.7223		NKX2-8;downstream;
7	rs_7_37758295	37758295	T	A	PC1	83.7223		Intergenic;
7	rs_7_37758445	37758445	T	G	PC1	83.7223		Intergenic;
7	rs_7_37759480	37759480	A	G	PC1	83.7223		Intergenic;
7	rs_7_37759837	37759837	G	A	PC1	83.7223		Intergenic;
7	rs_7_37762365	37762365	C	A	PC1	83.7223		Intergenic;
7	rs_7_37765067	37765067	G	A	PC1	83.7223		Intergenic;
7	rs_7_37765461	37765461	G	T	PC1	83.7223		Intergenic;
7	rs_7_37767349	37767349	T	C	PC1	83.7223		Exon;NKX2-8;NKX2-8;-;0;AGC;Nonsynonymous;Ser;Gly;
7	rs_7_37772764	37772764	C	G	PC1	83.7223		Intergenic;
7	rs_7_37772910	37772910	C	T	PC1	83.7223		Intergenic;
7	rs_7_37774279	37774279	C	G	PC1	83.7223		Intergenic;
7	rs_7_37774589	37774589	A	C	PC1	83.7223		Intergenic;
7	rs_7_37774603	37774603	G	A	PC1	83.7223		Intergenic;
7	rs_7_37777528	37777528	A	C	PC1	83.7223		Intergenic;
7	rs_7_37790635	37790635	T	C	PC1	83.7223		Intergenic;
7	rs_7_37793458	37793458	G	T	PC1	83.7223		Intergenic;
7	rs_7_37793645	37793645	G	A	PC1	83.7223		Intergenic;
7	rs_7_37806336	37806336	T	C	PC1	83.7223		PAX9;upstream;
7	rs_7_37814290	37814290	C	A	PC1	83.7223		Intron;PAX9;
7	rs_7_37819069	37819069	A	C	PC1	83.7223		Intron;PAX9;
7	rs_7_37904004	37904004	G	A	PC1	83.7223		Intergenic;
7	rs_7_37921825	37921825	G	A	PC1	83.7223		Intergenic;
7	rs_7_37935965	37935965	G	A	PC1	83.7223		Intergenic;
7	rs_7_37950365	37950365	T	C	PC1	83.7223		Intergenic;
7	rs_7_38024014	38024014	C	A	PC1	83.7223		Intergenic;
7	rs_7_38026976	38026976	A	G	PC1	83.7223		Intergenic;
7	rs_7_38034367	38034367	G	A	PC1	83.7223		Intergenic;
7	rs_7_38180566	38180566	T	A	PC1	83.7223		Intron;GCC2;
7	rs_7_38182090	38182090	G	A	PC1	83.7223		Intron;GCC2;
7	rs_7_38187910	38187910	A	G	PC1	83.7223		Intron;GCC2;
7	rs_7_38193441	38193441	A	G	PC1	83.7223		Intron;GCC2;
7	rs_7_38211517	38211517	G	A	PC1	83.7223		Intron;GCC2;
7	rs_7_38224768	38224768	A	G	PC1	83.7223		Intron;GCC2;
7	rs_7_38240573	38240573	T	G	PC1	83.7223		Intergenic;

7	rs_7_38255593	38255593	A	G	PC1	83.7223	Intergenic;
7	rs_7_38263160	38263160	T	C	PC1	83.7223	Intergenic;
7	rs_7_38270635	38270635	T	A	PC1	83.7223	Intergenic;
7	rs_7_38276982	38276982	T	C	PC1	83.7223	Intergenic;
7	rs_7_38279671	38279671	T	C	PC1	83.7223	TTC6;upstream;
7	rs_7_38280006	38280006	C	T	PC1	83.7223	TTC6;upstream;
7	rs_7_38281264	38281264	A	G	PC1	83.7223	TTC6;upstream;
7	rs_7_38287858	38287858	T	C	PC1	83.7223	TTC6;upstream;
7	rs_7_38288331	38288331	A	C	PC1	83.7223	TTC6;upstream;
7	rs_7_38288695	38288695	T	C	PC1	83.7223	TTC6;upstream;
7	rs_7_38289308	38289308	T	C	PC1	83.7223	Intron;TTC6;
7	rs_7_38297087	38297087	C	T	PC1	83.7223	Intron;TTC6;
7	rs_7_38298566	38298566	T	C	PC1	83.7223	Intron;TTC6;
7	rs_7_38299350	38299350	A	T	PC1	83.7223	Intron;TTC6;
7	rs_7_38299649	38299649	G	A	PC1	83.7223	Intron;TTC6;
7	rs_7_38300075	38300075	T	C	PC1	83.7223	Intron;TTC6;
7	rs_7_38393407	38393407	A	T	PC1	83.7223	Intergenic;
7	rs_7_38400055	38400055	A	G	PC1	83.7223	Intergenic;
7	rs_7_38400810	38400810	G	A	PC1	83.7223	Intergenic;
7	rs_7_38405504	38405504	C	T	PC1	83.7223	Intergenic;
7	rs_7_38409128	38409128	A	C	PC1	83.7223	Intergenic;
7	rs_7_38414459	38414459	G	T	PC1	83.7223	Intergenic;
7	rs_7_38442612	38442612	T	C	PC1	83.7223	Intergenic;
7	rs_7_38444798	38444798	A	C	PC1	83.7223	Intergenic;
7	rs_7_38449233	38449233	G	A	PC1	83.7223	Intergenic;
7	rs_7_38459002	38459002	C	T	PC1	83.7223	Intergenic;
7	rs_7_38461328	38461328	T	C	PC1	83.7223	Intergenic;
7	rs_7_38806607	38806607	G	C	PC1	83.7223	Intron;ZNF410;
7	rs_7_38921430	38921430	A	G	PC1	83.7223	Intergenic;
7	rs_7_38923755	38923755	A	G	PC1	83.7223	Intergenic;
7	rs_7_38941596	38941596	T	A	PC1	83.7223	Intergenic;
7	rs_7_38943813	38943813	A	C	PC1	83.7223	VSX2;upstream;
7	rs_7_39496182	39496182	G	A	PC1	83.7223	Intron;TTLL5;
7	rs_7_39498769	39498769	A	T	PC1	83.7223	Intron;TTLL5;
7	rs_7_39579614	39579614	T	C	PC1	83.7223	Intron;TTLL5;
7	rs_7_39593037	39593037	A	G	PC1	83.7223	Intron;TTLL5;
7	rs_7_39593404	39593404	T	C	PC1	83.7223	Intron;TTLL5;
7	rs_7_39599452	39599452	G	A	PC1	83.7223	Intron;TTLL5;
7	rs_7_39601690	39601690	T	C	PC1	83.7223	TTLL5;downstream;TGFB3;downstream;
7	rs_7_39808081	39808081	G	A	PC1	83.7223	Intergenic;
7	rs_7_40064078	40064078	C	T	PC1	83.7223	Intron;LRRC74B;
7	rs_7_40064482	40064482	G	A	PC1	83.7223	Intron;LRRC74B;
7	rs_7_40066607	40066607	T	C	PC1	83.7223	Intron;LRRC74B;
7	rs_7_40066788	40066788	A	T	PC1	83.7223	Intron;LRRC74B;
7	rs_7_40072849	40072849	C	A	PC1	83.7223	LRRC74B;downstream;
7	rs_7_40072948	40072948	C	T	PC1	83.7223	LRRC74B;downstream;
7	rs_7_40072966	40072966	A	G	PC1	83.7223	LRRC74B;downstream;
7	rs_7_40073937	40073937	T	C	PC1	83.7223	LRRC74B;downstream;
7	rs_7_40075217	40075217	T	C	PC1	83.7223	LRRC74B;downstream;
7	rs_7_40077194	40077194	T	C	PC1	83.7223	LRRC74B;downstream;
7	rs_7_40100109	40100109	C	T	PC1	83.7223	Intergenic;
7	rs_7_40361622	40361622	G	A	PC1	83.7223	ACBP4;upstream;SAMD15;upstream;
7	rs_7_40362788	40362788	A	G	PC1	83.7223	Intron;TMED8;
7	rs_7_40364318	40364318	T	A	PC1	83.7223	Intron;TMED8;
7	rs_7_40364858	40364858	T	C	PC1	83.7223	Intron;TMED8;
7	rs_7_40365194	40365194	G	A	PC1	83.7223	Exon;TMED8;TMED8;-;1;GCG;Nonsynonymous;Ala;Val;
7	rs_7_40365843	40365843	G	A	PC1	83.7223	VIPAS39;downstream;
7	rs_7_40377753	40377753	G	A	PC1	83.7223	Intron;VIPAS39;
7	rs_7_40426585	40426585	A	G	PC1	83.7223	DAXX;upstream;SPTLC2;downstream;
7	rs_7_40430231	40430231	C	T	PC1	83.7223	Exon;SPTLC2;SPTLC2;-;0;GAT;Nonsynonymous;Asp;Asn;
7	rs_7_40435145	40435145	A	G	PC1	83.7223	Intron;SPTLC2;
7	rs_7_40461553	40461553	C	T	PC1	83.7223	Intron;SPTLC2;
7	rs_7_40472867	40472867	C	T	PC1	83.7223	Exon;SPTLC2;SPTLC2;-;0;GAA;Nonsynonymous;Glu;Lys;
7	rs_7_40474999	40474999	T	A	PC1	83.7223	Intron;SPTLC2;
7	rs_7_40475388	40475388	C	T	PC1	83.7223	Intron;SPTLC2;
7	rs_7_40475414	40475414	C	T	PC1	83.7223	Intron;SPTLC2;
7	rs_7_40477156	40477156	C	T	PC1	83.7223	Intron;SPTLC2;
7	rs_7_40478280	40478280	C	T	PC1	83.7223	Intron;SPTLC2;
7	rs_7_40494068	40494068	C	A	PC1	83.7223	SPTLC2;upstream;
7	rs_7_40494390	40494390	A	G	PC1	83.7223	SPTLC2;upstream;
7	rs_7_40601986	40601986	T	C	PC1	83.7223	Intron;ADCK1;
7	rs_7_40731497	40731497	A	T	PC1	83.7223	TCHH;downstream;
7	rs_7_40833675	40833675	A	G	PC1	83.7223	Intron;NRXN3;
7	rs_7_40838855	40838855	C	G	PC1	83.7223	Intron;NRXN3;
7	rs_7_40839417	40839417	T	A	PC1	83.7223	Intron;NRXN3;
7	rs_7_40841536	40841536	T	C	PC1	83.7223	Intron;NRXN3;
7	rs_7_40841546	40841546	G	C	PC1	83.7223	Intron;NRXN3;
7	rs_7_40841722	40841722	G	C	PC1	83.7223	Intron;NRXN3;
7	rs_7_40842655	40842655	T	C	PC1	83.7223	Intron;NRXN3;
7	rs_7_40843276	40843276	A	C	PC1	83.7223	Intron;NRXN3;
7	rs_7_40843362	40843362	A	G	PC1	83.7223	Intron;NRXN3;
7	rs_7_40850843	40850843	C	T	PC1	83.7223	Intron;NRXN3;
7	rs_7_40856729	40856729	G	A	PC1	83.7223	Intron;NRXN3;
7	rs_7_40857511	40857511	T	C	PC1	77.99387	Intron;NRXN3;
7	rs_7_40859642	40859642	G	A	PC1	83.7223	Intron;NRXN3;
7	rs_7_40888375	40888375	C	T	PC1	83.7223	Intron;NRXN3;
7	rs_7_40889571	40889571	A	G	PC1	83.7223	Intron;NRXN3;
7	rs_7_40897405	40897405	A	G	PC1	83.7223	Intron;NRXN3;

7	rs_7_40899872	40899872	G	C	PC1	83.7223	Intron;NRXN3;
7	rs_7_40908704	40908704	T	C	PC1	83.7223	Intron;NRXN3;
7	rs_7_40916882	40916882	G	C	PC1	83.7223	Intron;NRXN3;
7	rs_7_40919641	40919641	G	C	PC1	83.7223	Intron;NRXN3;
7	rs_7_40920847	40920847	A	T	PC1	83.7223	Intron;NRXN3;
7	rs_7_40928722	40928722	A	G	PC1	83.7223	Intron;NRXN3;
7	rs_7_40931120	40931120	T	C	PC1	83.7223	Intron;NRXN3;
7	rs_7_40933633	40933633	A	T	PC1	83.7223	Intron;NRXN3;
7	rs_7_40935164	40935164	C	T	PC1	83.7223	Intron;NRXN3;
7	rs_7_40943122	40943122	A	T	PC1	83.7223	Intron;NRXN3;
7	rs_7_40946564	40946564	A	G	PC1	83.7223	Intron;NRXN3;
7	rs_7_40952806	40952806	G	T	PC1	83.7223	Intron;NRXN3;
7	rs_7_40954342	40954342	T	C	PC1	83.7223	Intron;NRXN3;
7	rs_7_40954602	40954602	T	C	PC1	83.7223	Intron;NRXN3;
7	rs_7_40957269	40957269	T	C	PC1	83.7223	Intron;NRXN3;
7	rs_7_40961790	40961790	G	A	PC1	83.7223	Intron;NRXN3;
7	rs_7_40965204	40965204	T	A	PC1	83.7223	Intron;NRXN3;
7	rs_7_40967370	40967370	G	A	PC1	83.7223	Intron;NRXN3;
7	rs_7_40978454	40978454	T	C	PC1	83.7223	Intron;NRXN3;
7	rs_7_41009044	41009044	C	A	PC1	83.7223	Intron;NRXN3;
7	rs_7_41016730	41016730	T	C	PC1	83.7223	Intron;NRXN3;
7	rs_7_41081771	41081771	C	T	PC1	83.7223	Intron;NRXN3;
7	rs_7_41088733	41088733	C	T	PC1	83.7223	Intron;NRXN3;
7	rs_7_41096284	41096284	C	G	PC1	83.7223	Intron;NRXN3;
7	rs_7_41101938	41101938	C	T	PC1	83.7223	Intron;NRXN3;
7	rs_7_41103431	41103431	T	A	PC1	83.7223	Intron;NRXN3;
7	rs_7_41104819	41104819	T	C	PC1	83.7223	Intron;NRXN3;
7	rs_7_41105084	41105084	T	C	PC1	83.7223	Intron;NRXN3;
7	rs_7_41105114	41105114	G	T	PC1	83.7223	Intron;NRXN3;
7	rs_7_41110984	41110984	G	T	PC1	83.7223	Intron;NRXN3;
7	rs_7_41113029	41113029	T	C	PC1	83.7223	Intron;NRXN3;
7	rs_7_41115253	41115253	C	T	PC1	83.7223	Intron;NRXN3;
7	rs_7_41116650	41116650	T	C	PC1	83.7223	Intron;NRXN3;
7	rs_7_41120374	41120374	G	A	PC1	83.7223	Intron;NRXN3;
7	rs_7_41128794	41128794	G	A	PC1	83.7223	Intron;NRXN3;
7	rs_7_41135007	41135007	A	G	PC1	83.7223	Intron;NRXN3;
7	rs_7_41135285	41135285	G	A	PC1	83.7223	Intron;NRXN3;
7	rs_7_41136437	41136437	G	A	PC1	83.7223	Intron;NRXN3;
7	rs_7_41138508	41138508	G	A	PC1	83.7223	Intron;NRXN3;
7	rs_7_41142111	41142111	A	G	PC1	83.7223	Intron;NRXN3;
7	rs_7_41142963	41142963	A	G	PC1	83.7223	Intron;NRXN3;
7	rs_7_41146303	41146303	C	T	PC1	83.7223	Intron;NRXN3;
7	rs_7_41160612	41160612	C	G	PC1	83.7223	Intron;NRXN3;
7	rs_7_41165796	41165796	G	A	PC1	83.7223	Intron;NRXN3;
7	rs_7_41167375	41167375	T	G	PC1	83.7223	Intron;NRXN3;
7	rs_7_41167890	41167890	A	C	PC1	83.7223	Intron;NRXN3;
7	rs_7_41173231	41173231	T	C	PC1	83.7223	Intron;NRXN3;
7	rs_7_41174465	41174465	T	C	PC1	83.7223	Intron;NRXN3;
7	rs_7_41174945	41174945	A	C	PC1	83.7223	Intron;NRXN3;
7	rs_7_41209896	41209896	A	G	PC1	83.7223	Intron;NRXN3;
7	rs_7_41213769	41213769	T	C	PC1	83.7223	Intron;NRXN3;
7	rs_7_41218324	41218324	G	A	PC1	83.7223	Intron;NRXN3;
7	rs_7_41224327	41224327	G	A	PC1	83.7223	Intron;NRXN3;
7	rs_7_41242599	41242599	A	C	PC1	77.99387	Intron;NRXN3;
7	rs_7_41300224	41300224	G	A	PC1	83.7223	Intron;NRXN3;
7	rs_7_41330137	41330137	C	G	PC1	83.7223	Intron;NRXN3;
7	rs_7_41541825	41541825	C	A	PC1	83.7223	Intron;NRXN3;
7	rs_7_41581887	41581887	A	G	PC1	83.7223	Intron;NRXN3;
7	rs_7_41581889	41581889	C	G	PC1	83.7223	Intron;NRXN3;
7	rs_7_41587080	41587080	C	T	PC1	83.7223	Intron;NRXN3;
7	rs_7_41590965	41590965	A	G	PC1	83.7223	Intron;NRXN3;
7	rs_7_41594769	41594769	A	G	PC1	83.7223	Intron;NRXN3;
7	rs_7_41595258	41595258	A	T	PC1	83.7223	Intron;NRXN3;
7	rs_7_41602807	41602807	C	T	PC1	77.99387	Intron;NRXN3;
7	rs_7_41608029	41608029	G	A	PC1	83.7223	Intron;NRXN3;
7	rs_7_41609015	41609015	A	T	PC1	83.7223	Intron;NRXN3;
7	rs_7_41609921	41609921	T	C	PC1	83.7223	Intron;NRXN3;
7	rs_7_41609941	41609941	T	C	PC1	83.7223	Intron;NRXN3;
7	rs_7_41610211	41610211	G	T	PC1	83.7223	Intron;NRXN3;
7	rs_7_41610492	41610492	A	G	PC1	83.7223	Intron;NRXN3;
7	rs_7_41611159	41611159	G	T	PC1	83.7223	Intron;NRXN3;
7	rs_7_41612457	41612457	A	G	PC1	83.7223	Intron;NRXN3;
7	rs_7_41612707	41612707	T	C	PC1	83.7223	Intron;NRXN3;
7	rs_7_41613323	41613323	C	A	PC1	83.7223	Intron;NRXN3;
7	rs_7_41613750	41613750	A	C	PC1	83.7223	Intron;NRXN3;
7	rs_7_41614643	41614643	G	A	PC1	83.7223	Intron;NRXN3;
7	rs_7_41617422	41617422	A	C	PC1	83.7223	Intron;NRXN3;
7	rs_7_41617438	41617438	A	C	PC1	83.7223	Intron;NRXN3;
7	rs_7_41618270	41618270	G	A	PC1	83.7223	Intron;NRXN3;
7	rs_7_41621316	41621316	T	A	PC1	83.7223	Intron;NRXN3;
7	rs_7_41621545	41621545	T	C	PC1	83.7223	Intron;NRXN3;
7	rs_7_41623338	41623338	C	T	PC1	83.7223	Intron;NRXN3;
7	rs_7_41624165	41624165	T	C	PC1	83.7223	Intron;NRXN3;
7	rs_7_41624708	41624708	C	A	PC1	83.7223	Intron;NRXN3;
7	rs_7_41625156	41625156	G	T	PC1	83.7223	Intron;NRXN3;
7	rs_7_41626063	41626063	G	A	PC1	83.7223	Intron;NRXN3;
7	rs_7_41626257	41626257	A	G	PC1	83.7223	Intron;NRXN3;
7	rs_7_41627221	41627221	A	G	PC1	83.7223	Intron;NRXN3;

7	rs_7_41628773	41628773	A	C	PC1	83.7223	Intron;NRXN3;
7	rs_7_41632754	41632754	C	A	PC1	83.7223	Intron;NRXN3;
7	rs_7_41634932	41634932	A	G	PC1	83.7223	Intron;NRXN3;
7	rs_7_41637538	41637538	C	T	PC1	83.7223	Intron;NRXN3;
7	rs_7_41637769	41637769	G	A	PC1	83.7223	Intron;NRXN3;
7	rs_7_41638002	41638002	G	A	PC1	83.7223	Intron;NRXN3;
7	rs_7_41638625	41638625	T	G	PC1	83.7223	Intron;NRXN3;
7	rs_7_41641659	41641659	G	A	PC1	83.7223	Intron;NRXN3;
7	rs_7_41642101	41642101	A	G	PC1	83.7223	Intron;NRXN3;
7	rs_7_41643765	41643765	C	A	PC1	83.7223	Intron;NRXN3;
7	rs_7_41643785	41643785	T	G	PC1	83.7223	Intron;NRXN3;
7	rs_7_41712634	41712634	A	G	PC1	83.7223	Intergenic;
7	rs_7_41712797	41712797	G	C	PC1	83.7223	Intergenic;
7	rs_7_41716446	41716446	A	G	PC1	83.7223	Intergenic;
7	rs_7_41722301	41722301	G	T	PC1	83.7223	Intergenic;
7	rs_7_41723762	41723762	T	C	PC1	83.7223	Intergenic;
7	rs_7_41725569	41725569	C	T	PC1	83.7223	Intergenic;
7	rs_7_41728481	41728481	G	T	PC1	83.7223	Intergenic;
7	rs_7_41730243	41730243	T	C	PC1	83.7223	Intergenic;
7	rs_7_41730758	41730758	T	C	PC1	83.7223	Intergenic;
7	rs_7_41732599	41732599	G	C	PC1	83.7223	Intergenic;
7	rs_7_41741825	41741825	G	A	PC1	83.7223	Intergenic;
7	rs_7_41748151	41748151	G	A	PC1	83.7223	DIO2;downstream;
7	rs_7_41902828	41902828	T	G	PC1	83.7223	Intron;CEP128;
7	rs_7_43950410	43950410	G	A	PC1	83.7223	Intergenic;
7	rs_7_44536858	44536858	C	T	PC1	83.7223	Intron;PTPN21;
7	rs_7_44591634	44591634	C	T	PC1	83.7223	Intron;PTPN21;
7	rs_7_44938901	44938901	C	G	PC1	83.7223	FOXN3;downstream;
7	rs_7_45708862	45708862	T	C	PC1	83.7223	Exon;C5H14ORF159;C5H14ORF159;+;2;ATT;Synonymous;Ile;Ile;
7	rs_7_45737535	45737535	T	C	PC1	83.7223	GPR68;upstream;
7	rs_7_46600523	46600523	A	T	PC1	83.7223	Intron;BTBD7;
7	rs_7_46606943	46606943	G	A	PC1	83.7223	Intron;BTBD7;
7	rs_7_46607539	46607539	C	G	PC1	83.7223	Intron;BTBD7;
7	rs_7_46784645	46784645	C	G	PC1	83.7223	FORMIN;downstream;
7	rs_7_46794818	46794818	G	A	PC1	83.7223	Intron;FORMIN;
7	rs_7_46800287	46800287	A	G	PC1	83.7223	Intron;FORMIN;
7	rs_7_46813212	46813212	A	G	PC1	83.7223	Intron;FORMIN;
7	rs_7_46813517	46813517	A	G	PC1	83.7223	Intron;FORMIN;
7	rs_7_46814947	46814947	G	A	PC1	83.7223	Intron;FORMIN;
7	rs_7_46816452	46816452	T	C	PC1	83.7223	Intron;FORMIN;
7	rs_7_46818479	46818479	G	A	PC1	83.7223	Intron;FORMIN;
7	rs_7_46832106	46832106	C	T	PC1	83.7223	Intron;FORMIN;
7	rs_7_46874521	46874521	A	G	PC1	83.7223	Intergenic;
7	rs_7_46970936	46970936	T	A	PC1	83.7223	PPP4R4;upstream;
7	rs_7_46971032	46971032	G	A	PC1	83.7223	PPP4R4;upstream;
7	rs_7_46994508	46994508	T	C	PC1	83.7223	Intron;PPP4R4;
7	rs_7_46997053	46997053	T	C	PC1	83.7223	Exon;PPP4R4;PPP4R4;+;2;CAT;Synonymous;His;His;
7	rs_7_47059878	47059878	A	G	PC1	83.7223	Intergenic;
7	rs_7_47060015	47060015	A	G	PC1	83.7223	Intergenic;
7	rs_7_47099848	47099848	A	T	PC1	83.7223	Intergenic;
7	rs_7_47099972	47099972	A	G	PC1	83.7223	Intergenic;
7	rs_7_47103279	47103279	A	C	PC1	83.7223	SERPINA1;upstream;
7	rs_7_47131476	47131476	A	C	PC1	83.7223	Intron;SPIA9;
7	rs_7_47146568	47146568	T	C	PC1	83.7223	SPIA9;upstream;SERPINA3;downstream;
7	rs_7_47168724	47168724	A	G	PC1	83.7223	SERPINA3;upstream;
7	rs_7_47169244	47169244	T	A	PC1	83.7223	SERPINA3;upstream;
7	rs_7_47169852	47169852	G	A	PC1	83.7223	SERPINA3;upstream;
7	rs_7_47172358	47172358	C	A	PC1	83.7223	SERPINA3;upstream;
7	rs_7_47173377	47173377	G	A	PC1	83.7223	SERPINA3;upstream;
7	rs_7_47185314	47185314	A	G	PC1	83.7223	Intergenic;
7	rs_7_47188738	47188738	T	C	PC1	83.7223	Intergenic;
7	rs_7_47189361	47189361	C	G	PC1	83.7223	Intergenic;
7	rs_7_47190684	47190684	T	G	PC1	83.7223	Intergenic;
7	rs_7_47197829	47197829	A	C	PC1	83.7223	Intergenic;
7	rs_7_47199251	47199251	T	C	PC1	83.7223	Intergenic;
7	rs_7_47202942	47202942	G	A	PC1	83.7223	Intergenic;
7	rs_7_47208565	47208565	T	A	PC1	83.7223	Intergenic;
7	rs_7_47213674	47213674	A	G	PC1	83.7223	Intergenic;
7	rs_7_47217961	47217961	C	T	PC1	83.7223	Intergenic;
7	rs_7_47221510	47221510	A	T	PC1	83.7223	Intergenic;
7	rs_7_47221623	47221623	T	C	PC1	83.7223	Intergenic;
7	rs_7_47227884	47227884	A	T	PC1	83.7223	Intergenic;
7	rs_7_47228945	47228945	T	A	PC1	83.7223	Intergenic;
7	rs_7_47243280	47243280	G	A	PC1	83.7223	GSC;downstream;
7	rs_7_47300687	47300687	C	G	PC1	83.7223	Intergenic;
7	rs_7_47352959	47352959	T	G	PC1	77.99387	Intergenic;
7	rs_7_47401520	47401520	T	C	PC1	83.7223	Intron;DICER1;
7	rs_7_47401930	47401930	T	A	PC1	83.7223	Intron;DICER1;
7	rs_7_47458274	47458274	G	A	PC1	83.7223	Intron;SYNE1B;
7	rs_7_47467907	47467907	T	A	PC1	83.7223	Intron;SYNE1B;
7	rs_7_47467957	47467957	C	T	PC1	83.7223	Intron;SYNE1B;
7	rs_7_47468236	47468236	C	A	PC1	83.7223	Intron;SYNE1B;
7	rs_7_47468329	47468329	A	G	PC1	83.7223	Intron;SYNE1B;
7	rs_7_47471134	47471134	C	A	PC1	83.7223	Intron;SYNE1B;
7	rs_7_47472451	47472451	A	G	PC1	77.99387	Intron;SYNE1B;
7	rs_7_47607475	47607475	G	C	PC1	77.99387	Intergenic;
7	rs_7_47607476	47607476	G	A	PC1	77.99387	Intergenic;
7	rs_7_47636726	47636726	A	G	PC1	83.7223	Intergenic;

7	rs_7_47638556	47638556	A	G	PC1	83.7223	Intergenic;
7	rs_7_47641150	47641150	T	C	PC1	83.7223	Intergenic;
7	rs_7_47646227	47646227	T	C	PC1	83.7223	Intergenic;
7	rs_7_47647121	47647121	T	C	PC1	83.7223	Intergenic;
7	rs_7_47647344	47647344	G	A	PC1	83.7223	Intergenic;
7	rs_7_47649936	47649936	A	C	PC1	83.7223	Intergenic;
7	rs_7_47651128	47651128	C	T	PC1	83.7223	Intergenic;
7	rs_7_47663319	47663319	G	C	PC1	83.7223	Intergenic;
7	rs_7_47776589	47776589	T	C	PC1	77.99387	Intergenic;
7	rs_7_47777083	47777083	C	T	PC1	83.7223	Intergenic;
7	rs_7_47777486	47777486	T	C	PC1	83.7223	Predicted;downstream;
7	rs_7_47778908	47778908	G	A	PC1	83.7223	Predicted;downstream;
7	rs_7_47779214	47779214	T	C	PC1	83.7223	Predicted;downstream;
7	rs_7_47779398	47779398	A	G	PC1	83.7223	Predicted;downstream;
7	rs_7_47780080	47780080	T	G	PC1	83.7223	Predicted;downstream;
7	rs_7_47780215	47780215	T	C	PC1	83.7223	Predicted;downstream;
7	rs_7_48306623	48306623	G	A	PC1	83.7223	Intergenic;
7	rs_7_48309246	48309246	G	A	PC1	83.7223	Intergenic;
7	rs_7_48310616	48310616	A	C	PC1	83.7223	Intergenic;
7	rs_7_48318114	48318114	A	T	PC1	83.7223	Intergenic;
7	rs_7_48326940	48326940	T	C	PC1	83.7223	Intergenic;
7	rs_7_48330722	48330722	T	C	PC1	83.7223	Intergenic;
7	rs_7_48335374	48335374	T	C	PC1	83.7223	Intergenic;
7	rs_7_48354904	48354904	T	A	PC1	83.7223	Intergenic;
7	rs_7_48356293	48356293	A	C	PC1	83.7223	Intergenic;
7	rs_7_48356452	48356452	T	C	PC1	83.7223	Intergenic;
7	rs_7_48994959	48994959	G	A	PC1	83.7223	Intergenic;
7	rs_7_49011572	49011572	G	A	PC1	83.7223	Intergenic;
7	rs_7_49013010	49013010	T	C	PC1	83.7223	Intergenic;
7	rs_7_49018982	49018982	G	A	PC1	83.7223	Intergenic;
7	rs_7_49027820	49027820	C	T	PC1	83.7223	Intergenic;
7	rs_7_49033801	49033801	T	C	PC1	83.7223	Intergenic;
7	rs_7_49040168	49040168	C	T	PC1	83.7223	Intergenic;
7	rs_7_49040186	49040186	A	G	PC1	83.7223	Intergenic;
7	rs_7_49042945	49042945	C	T	PC1	83.7223	Intergenic;
7	rs_7_49043537	49043537	T	C	PC1	83.7223	Intergenic;
7	rs_7_49044143	49044143	A	G	PC1	83.7223	Intergenic;
7	rs_7_49044770	49044770	G	C	PC1	83.7223	Intergenic;
7	rs_7_49047428	49047428	C	T	PC1	83.7223	Intergenic;
7	rs_7_49073049	49073049	A	G	PC1	83.7223	Intergenic;
7	rs_7_49079234	49079234	T	C	PC1	83.7223	Intergenic;
7	rs_7_49079504	49079504	T	C	PC1	83.7223	Intergenic;
7	rs_7_49079795	49079795	A	G	PC1	83.7223	Intergenic;
7	rs_7_49085351	49085351	T	C	PC1	83.7223	Intergenic;
7	rs_7_49087071	49087071	T	C	PC1	83.7223	Intergenic;
7	rs_7_49087344	49087344	T	C	PC1	83.7223	Intergenic;
7	rs_7_49087462	49087462	G	A	PC1	83.7223	Intergenic;
7	rs_7_49087513	49087513	A	G	PC1	83.7223	Intergenic;
7	rs_7_49091575	49091575	T	C	PC1	83.7223	Intergenic;
7	rs_7_49091826	49091826	C	G	PC1	83.7223	Intergenic;
7	rs_7_49092241	49092241	C	A	PC1	83.7223	Intergenic;
7	rs_7_49092325	49092325	A	C	PC1	83.7223	Intergenic;
7	rs_7_49093275	49093275	G	T	PC1	83.7223	Intergenic;
7	rs_7_49095448	49095448	A	C	PC1	83.7223	Intergenic;
7	rs_7_49099080	49099080	C	T	PC1	83.7223	Intergenic;
7	rs_7_49109196	49109196	A	C	PC1	83.7223	Intergenic;
7	rs_7_49110557	49110557	C	A	PC1	83.7223	Intergenic;
7	rs_7_49112563	49112563	C	G	PC1	83.7223	Intergenic;
7	rs_7_49112630	49112630	C	G	PC1	83.7223	Intergenic;
7	rs_7_49115110	49115110	G	A	PC1	83.7223	Intergenic;
7	rs_7_49145036	49145036	T	G	PC1	83.7223	Intergenic;
7	rs_7_49150834	49150834	T	A	PC1	83.7223	Intergenic;
7	rs_7_49173814	49173814	G	C	PC1	83.7223	Intergenic;
7	rs_7_49175620	49175620	A	G	PC1	83.7223	Intergenic;
7	rs_7_49182136	49182136	T	C	PC1	83.7223	Intergenic;
7	rs_7_49182255	49182255	G	A	PC1	83.7223	Intergenic;
7	rs_7_49184979	49184979	G	A	PC1	83.7223	Intergenic;
7	rs_7_49186778	49186778	A	G	PC1	83.7223	Intergenic;
7	rs_7_49190638	49190638	C	G	PC1	83.7223	Intergenic;
7	rs_7_49190885	49190885	C	T	PC1	83.7223	Intergenic;
7	rs_7_49209163	49209163	C	T	PC1	83.7223	Intergenic;
7	rs_7_49212370	49212370	T	C	PC1	83.7223	Intergenic;
7	rs_7_49238632	49238632	G	T	PC1	83.7223	Intergenic;
7	rs_7_49239960	49239960	T	C	PC1	83.7223	Intergenic;
7	rs_7_49241328	49241328	C	T	PC1	83.7223	Intergenic;
7	rs_7_49241363	49241363	T	C	PC1	83.7223	Intergenic;
7	rs_7_49248096	49248096	T	G	PC1	83.7223	Intergenic;
7	rs_7_49255088	49255088	A	T	PC1	83.7223	Intergenic;
7	rs_7_49256398	49256398	C	A	PC1	83.7223	Intergenic;
7	rs_7_49259896	49259896	T	C	PC1	83.7223	Intergenic;
7	rs_7_49266935	49266935	T	C	PC1	83.7223	Intergenic;
7	rs_7_49269208	49269208	A	G	PC1	83.7223	Intergenic;
7	rs_7_50674418	50674418	G	A	PC1	83.7223	Intergenic;
7	rs_7_50860972	50860972	A	G	PC1	83.7223	Intergenic;
7	rs_7_50861170	50861170	G	A	PC1	83.7223	Intergenic;
7	rs_7_50867567	50867567	T	C	PC1	83.7223	Intergenic;
7	rs_7_50870914	50870914	A	G	PC1	83.7223	Intergenic;
7	rs_7_50871287	50871287	T	C	PC1	83.7223	Intergenic;

7	rs_7_50879663	50879663	G	T	PC1	83.7223	Intergenic;
7	rs_7_50890519	50890519	A	G	PC1	83.7223	Intergenic;
7	rs_7_51824960	51824960	G	A	PC1	83.7223	Intergenic;
7	rs_7_51828744	51828744	A	G	PC1	83.7223	Intergenic;
7	rs_7_51852651	51852651	A	G	PC1	83.7223	MARK3;upstream;
7	rs_7_52874925	52874925	A	G	PC1	83.7223	Intergenic;
7	rs_7_54225705	54225705	C	G	PC1	83.7223	Intergenic;
7	rs_7_55692493	55692493	G	A	PC1	83.7223	Intergenic;
7	rs_7_55692496	55692496	C	T	PC1	83.7223	Intergenic;
7	rs_7_56873192	56873192	A	T	Others	78.95076	Intergenic;
8	rs_8_2745949	2745949	C	T	Others	89.02837	Intergenic;
8	rs_8_3394566	3394566	T	C	PC1	83.7223	Intergenic;
8	rs_8_3511552	3511552	T	C	PC1	83.7223	Intergenic;
8	rs_8_3610207	3610207	G	T	PC1	83.7223	Intergenic;
8	rs_8_3715504	3715504	T	C	PC1	83.7223	Intron;NTNG1;
8	rs_8_3809107	3809107	T	C	PC1	77.99387	Intron;VAV3;
8	rs_8_3809200	3809200	C	T	PC1	77.99387	Intron;VAV3;
8	rs_8_3809946	3809946	T	A	PC1	77.99387	Intron;VAV3;
8	rs_8_3811437	3811437	G	T	PC1	83.7223	Intron;VAV3;
8	rs_8_4019497	4019497	C	T	PC1	83.7223	Intron;SLC25A24;
8	rs_8_4235301	4235301	G	A	PC1	83.7223	Intron;CAMSAP2;
8	rs_8_4235453	4235453	C	T	PC1	83.7223	Intron;CAMSAP2;
8	rs_8_4236668	4236668	T	C	PC1	83.7223	Intron;CAMSAP2;
8	rs_8_4369517	4369517	T	A	PC1	83.7223	Intergenic;
8	rs_8_4374820	4374820	C	A	PC1	83.7223	Intergenic;
8	rs_8_4444803	4444803	C	T	PC1	83.7223	Intergenic;
8	rs_8_4445413	4445413	G	A	PC1	83.7223	Intergenic;
8	rs_8_4536901	4536901	G	C	PC1	83.7223	Intron;NR5A2;
8	rs_8_4536902	4536902	C	T	PC1	83.7223	Intron;NR5A2;
8	rs_8_4537132	4537132	C	T	PC1	83.7223	Intron;NR5A2;
8	rs_8_4561440	4561440	C	T	PC1	83.7223	Intergenic;
8	rs_8_4563024	4563024	T	C	PC1	83.7223	Intergenic;
8	rs_8_4649526	4649526	C	G	PC1	83.7223	Intergenic;
8	rs_8_4649846	4649846	G	A	PC1	83.7223	Intergenic;
8	rs_8_4651501	4651501	G	A	PC1	83.7223	Intergenic;
8	rs_8_4652336	4652336	C	A	PC1	83.7223	Intergenic;
8	rs_8_4652808	4652808	C	G	PC1	83.7223	Intergenic;
8	rs_8_4653204	4653204	A	T	PC1	83.7223	Intergenic;
8	rs_8_4793563	4793563	A	T	PC1	83.7223	Intergenic;
8	rs_8_4794064	4794064	T	A	PC1	83.7223	Intergenic;
8	rs_8_4795594	4795594	A	G	PC1	83.7223	Intergenic;
8	rs_8_4802064	4802064	A	T	PC1	83.7223	Intergenic;
8	rs_8_4802609	4802609	A	G	PC1	83.7223	Intergenic;
8	rs_8_4802746	4802746	T	C	PC1	83.7223	Intergenic;
8	rs_8_4804530	4804530	G	A	PC1	83.7223	Intergenic;
8	rs_8_4805073	4805073	C	G	PC1	83.7223	Intergenic;
8	rs_8_4809395	4809395	A	G	PC1	83.7223	Intergenic;
8	rs_8_4810087	4810087	G	C	PC1	83.7223	Intergenic;
8	rs_8_4811069	4811069	C	T	PC1	83.7223	Intergenic;
8	rs_8_4823114	4823114	G	A	PC1	83.7223	Intergenic;
8	rs_8_4831356	4831356	C	T	PC1	83.7223	Intergenic;
8	rs_8_4836457	4836457	A	G	PC1	83.7223	Intergenic;
8	rs_8_4847646	4847646	C	A	PC1	83.7223	Intergenic;
8	rs_8_4853240	4853240	C	A	PC1	83.7223	Intergenic;
8	rs_8_4853395	4853395	G	A	PC1	83.7223	Intergenic;
8	rs_8_4854031	4854031	C	T	PC1	83.7223	Intergenic;
8	rs_8_4854368	4854368	A	C	PC1	83.7223	Intergenic;
8	rs_8_4854716	4854716	C	A	PC1	83.7223	Intergenic;
8	rs_8_4855210	4855210	G	A	PC1	83.7223	Intergenic;
8	rs_8_4855900	4855900	G	A	PC1	83.7223	Intergenic;
8	rs_8_4857064	4857064	A	G	PC1	83.7223	Intergenic;
8	rs_8_4857359	4857359	A	G	PC1	83.7223	Intergenic;
8	rs_8_4857420	4857420	T	C	PC1	83.7223	Intergenic;
8	rs_8_4857752	4857752	A	C	PC1	83.7223	Intergenic;
8	rs_8_4857826	4857826	T	C	PC1	83.7223	Intergenic;
8	rs_8_4858063	4858063	A	G	PC1	83.7223	Intergenic;
8	rs_8_4858080	4858080	T	G	PC1	83.7223	Intergenic;
8	rs_8_4859253	4859253	T	C	PC1	83.7223	Intergenic;
8	rs_8_4873507	4873507	G	A	PC1	83.7223	Intergenic;
8	rs_8_4874623	4874623	T	C	PC1	83.7223	Intergenic;
8	rs_8_4876200	4876200	A	G	PC1	83.7223	Intergenic;
8	rs_8_4878562	4878562	A	T	PC1	83.7223	Intergenic;
8	rs_8_4879668	4879668	G	A	PC1	83.7223	Intergenic;
8	rs_8_4884101	4884101	T	C	PC1	83.7223	Intergenic;
8	rs_8_4884571	4884571	G	A	PC1	83.7223	Intergenic;
8	rs_8_4887715	4887715	G	A	PC1	83.7223	Intergenic;
8	rs_8_4895977	4895977	C	T	PC1	83.7223	Intergenic;
8	rs_8_4896133	4896133	A	T	PC1	83.7223	Intergenic;
8	rs_8_4896839	4896839	T	C	PC1	83.7223	Intergenic;
8	rs_8_4898163	4898163	G	A	PC1	83.7223	Intergenic;
8	rs_8_4898254	4898254	G	A	PC1	83.7223	Intergenic;
8	rs_8_4946455	4946455	A	C	PC1	83.7223	Intron;PTPRC;
8	rs_8_4946836	4946836	C	A	PC1	83.7223	Exon;PTPRC;PTPRC;-;1;TGG;Nonsynonymous;Trp;Leu;
8	rs_8_4949027	4949027	T	C	PC1	77.99387	PTPRC;upstream;
8	rs_8_4951969	4951969	A	G	PC1	83.7223	PTPRC;upstream;
8	rs_8_4953879	4953879	G	A	PC1	77.99387	PTPRC;upstream;
8	rs_8_4954148	4954148	A	T	PC1	77.99387	PTPRC;upstream;
8	rs_8_4954248	4954248	C	T	PC1	77.99387	PTPRC;upstream;

8	rs_8_5019009	5019009	C	T	PC1	83.7223	ATP6V1G3;downstream;
8	rs_8_5032197	5032197	C	T	PC1	83.7223	Intergenic;
8	rs_8_5050525	5050525	G	A	PC1	83.7223	Intergenic;
8	rs_8_5139969	5139969	A	G	PC1	83.7223	Intergenic;
8	rs_8_5160595	5160595	A	G	PC1	83.7223	Intergenic;
8	rs_8_5163056	5163056	T	C	PC1	83.7223	Intergenic;
8	rs_8_5183646	5183646	G	A	PC1	83.7223	Intron;LHX9;
8	rs_8_5185702	5185702	G	T	PC1	83.7223	Intron;LHX9;
8	rs_8_5196124	5196124	C	G	PC1	83.7223	LHX9;upstream;
8	rs_8_5196841	5196841	C	T	PC1	83.7223	LHX9;upstream;
8	rs_8_5197779	5197779	C	G	PC1	83.7223	LHX9;upstream;
8	rs_8_5204809	5204809	A	G	PC1	83.7223	Intergenic;
8	rs_8_5215481	5215481	G	T	PC1	83.7223	Intergenic;
8	rs_8_5217704	5217704	G	A	PC1	83.7223	Intergenic;
8	rs_8_5221475	5221475	G	T	PC1	83.7223	Intergenic;
8	rs_8_5222293	5222293	T	C	PC1	83.7223	Intergenic;
8	rs_8_5222952	5222952	C	T	PC1	83.7223	Intergenic;
8	rs_8_5225194	5225194	T	C	PC1	83.7223	Intergenic;
8	rs_8_5226818	5226818	A	G	PC1	83.7223	Intergenic;
8	rs_8_5226861	5226861	C	T	PC1	83.7223	Intergenic;
8	rs_8_5227283	5227283	G	T	PC1	83.7223	Intergenic;
8	rs_8_5227360	5227360	C	T	PC1	83.7223	Intergenic;
8	rs_8_5227532	5227532	T	C	PC1	83.7223	Intergenic;
8	rs_8_5227784	5227784	A	T	PC1	83.7223	Intergenic;
8	rs_8_5227787	5227787	G	A	PC1	83.7223	Intergenic;
8	rs_8_5227994	5227994	C	T	PC1	83.7223	Intergenic;
8	rs_8_5228965	5228965	G	C	PC1	83.7223	Intergenic;
8	rs_8_5229057	5229057	C	A	PC1	83.7223	Intergenic;
8	rs_8_5231492	5231492	A	G	PC1	83.7223	Intergenic;
8	rs_8_5231599	5231599	G	A	PC1	83.7223	Intergenic;
8	rs_8_5234868	5234868	C	A	PC1	83.7223	Intergenic;
8	rs_8_5237341	5237341	G	A	PC1	83.7223	Intergenic;
8	rs_8_5237840	5237840	T	C	PC1	83.7223	Intergenic;
8	rs_8_5241308	5241308	A	T	PC1	83.7223	Intergenic;
8	rs_8_5242047	5242047	A	C	PC1	83.7223	Intergenic;
8	rs_8_5242888	5242888	C	T	PC1	83.7223	Intergenic;
8	rs_8_5251666	5251666	G	A	PC1	83.7223	Intergenic;
8	rs_8_5381570	5381570	C	T	PC1	83.7223	DENND1B;downstream;
8	rs_8_5384909	5384909	A	G	PC1	83.7223	DENND1B;downstream;
8	rs_8_5385008	5385008	G	A	PC1	83.7223	DENND1B;downstream;
8	rs_8_5389076	5389076	C	T	PC1	83.7223	Intergenic;
8	rs_8_5422723	5422723	T	A	PC1	83.7223	Intron;CRB1;
8	rs_8_5422797	5422797	G	A	PC1	83.7223	Intron;CRB1;
8	rs_8_5720999	5720999	C	T	PC1	83.7223	Intron;KCNT2;
8	rs_8_5951329	5951329	G	A	PC1	83.7223	Intergenic;
8	rs_8_6253677	6253677	G	A	PC1	83.7223	Intergenic;
8	rs_8_6261306	6261306	T	A	PC1	83.7223	Intergenic;
8	rs_8_6262171	6262171	A	G	PC1	83.7223	Intergenic;
8	rs_8_6269782	6269782	G	A	PC1	83.7223	Intergenic;
8	rs_8_6271007	6271007	G	A	PC1	83.7223	Intergenic;
8	rs_8_6277030	6277030	C	T	PC1	83.7223	Intergenic;
8	rs_8_6296360	6296360	C	T	PC1	83.7223	Intergenic;
8	rs_8_6298654	6298654	A	G	PC1	83.7223	Intergenic;
8	rs_8_6300788	6300788	C	T	PC1	83.7223	Intergenic;
8	rs_8_6301049	6301049	C	G	PC1	83.7223	Intergenic;
8	rs_8_6301497	6301497	T	C	PC1	83.7223	Intergenic;
8	rs_8_6302375	6302375	G	A	PC1	83.7223	Intergenic;
8	rs_8_6323422	6323422	A	G	PC1	83.7223	Intergenic;
8	rs_8_6324139	6324139	T	A	PC1	83.7223	Intergenic;
8	rs_8_6459436	6459436	A	G	PC1	83.7223	Intergenic;
8	rs_8_6460470	6460470	A	C	PC1	83.7223	Intergenic;
8	rs_8_6460568	6460568	C	T	PC1	83.7223	Intergenic;
8	rs_8_6497116	6497116	T	C	PC1	83.7223	CDC73;downstream;
8	rs_8_6501460	6501460	C	A	PC1	83.7223	Intron;CDC73;
8	rs_8_6512528	6512528	T	C	PC1	83.7223	Intron;CDC73;
8	rs_8_6515770	6515770	G	A	PC1	83.7223	Intron;CDC73;
8	rs_8_6517263	6517263	T	C	PC1	83.7223	Intron;CDC73;
8	rs_8_6521614	6521614	A	C	PC1	83.7223	Intron;CDC73;
8	rs_8_6525667	6525667	G	C	PC1	83.7223	Intron;CDC73;
8	rs_8_6533527	6533527	T	A	PC1	83.7223	Intron;CDC73;
8	rs_8_6539623	6539623	A	C	PC1	83.7223	Intron;CDC73;
8	rs_8_6541361	6541361	T	C	PC1	83.7223	Intron;CDC73;
8	rs_8_6541522	6541522	G	C	PC1	83.7223	Intron;CDC73;
8	rs_8_6541822	6541822	T	A	PC1	83.7223	Intron;CDC73;
8	rs_8_6542357	6542357	A	G	PC1	83.7223	Intron;CDC73;
8	rs_8_6544635	6544635	A	G	PC1	83.7223	Intron;CDC73;
8	rs_8_6544754	6544754	G	A	PC1	83.7223	Intron;CDC73;
8	rs_8_6545685	6545685	C	T	PC1	83.7223	Intron;CDC73;
8	rs_8_6549559	6549559	G	A	PC1	83.7223	Intron;CDC73;
8	rs_8_6550774	6550774	A	T	PC1	83.7223	Intron;CDC73;
8	rs_8_6837536	6837536	A	T	PC1	83.7223	Exon;HTATIP2;HTATIP2;-;0;TCA;Nonsynonymous;Ser;Thr;
8	rs_8_7032040	7032040	A	G	PC1	83.7223	Intergenic;
8	rs_8_7077096	7077096	G	T	PC1	83.7223	Intron;TEDC1;
8	rs_8_7078475	7078475	A	G	PC1	83.7223	Intron;TEDC1;
8	rs_8_7079097	7079097	A	C	PC1	83.7223	Intron;TEDC1;
8	rs_8_7080120	7080120	T	G	PC1	83.7223	Intron;TEDC1;
8	rs_8_7347811	7347811	A	G	PC1	83.7223	Intron;NOTCH2;
8	rs_8_7350001	7350001	A	G	PC1	83.7223	Intron;NOTCH2;

8	rs_8_7461727	7461727	T	C	PC1	83.7223	Exon;FMO5;FMO5;-;2;ACA;Synonymous;Thr;Thr;
8	rs_8_8057029	8057029	A	G	PC1	83.7223	Intron;COL11A1;
8	rs_8_8091653	8091653	G	A	PC1	83.7223	Intron;COL11A1;
8	rs_8_8112154	8112154	C	T	PC1	83.7223	Intron;COL11A1;
8	rs_8_8124801	8124801	T	C	PC1	83.7223	Intron;COL11A1;
8	rs_8_8688967	8688967	G	T	PC1	83.7223	Intron;DBT;
8	rs_8_8692709	8692709	T	C	PC1	83.7223	DBT;downstream;
8	rs_8_8693651	8693651	T	C	PC1	77.99387	DBT;downstream;LRRC39;upstream;
8	rs_8_8693792	8693792	G	A	PC1	83.7223	DBT;downstream;TRMT13;downstream;
8	rs_8_8701393	8701393	G	A	PC1	83.7223	Intron;LRRC39;
8	rs_8_8702332	8702332	G	A	PC1	83.7223	Exon;LRRC39;LRRC39;+;2;GAG;Synonymous;Glu;Glu;
8	rs_8_8702961	8702961	A	C	PC1	83.7223	Intron;LRRC39;
8	rs_8_8703923	8703923	T	C	PC1	83.7223	Intron;TRMT13;
8	rs_8_8703942	8703942	T	G	PC1	83.7223	Intron;TRMT13;
8	rs_8_8706909	8706909	A	C	PC1	83.7223	Exon;TRMT13;TRMT13;-;0;TTT;Nonsynonymous;Phe;Val;
8	rs_8_8717036	8717036	C	T	PC1	83.7223	Exon;SASS6;SASS6;+;1;GCG;Nonsynonymous;Ala;Val;
8	rs_8_8718034	8718034	G	T	PC1	83.7223	Intron;SASS6;
8	rs_8_8718060	8718060	C	T	PC1	83.7223	Intron;SASS6;
8	rs_8_8718793	8718793	C	T	PC1	83.7223	Intron;SASS6;
8	rs_8_8719018	8719018	T	C	PC1	83.7223	Intron;SASS6;
8	rs_8_8719816	8719816	A	G	PC1	83.7223	Intron;SASS6;
8	rs_8_8722454	8722454	C	T	PC1	83.7223	SASS6;downstream;MFSD14A;downstream;
8	rs_8_8724610	8724610	T	C	PC1	83.7223	Exon;MFSD14A;MFSD14A;-;2;CCA;Synonymous;Pro;Pro;
8	rs_8_8729741	8729741	G	C	PC1	83.7223	Intron;MFSD14A;
8	rs_8_8753800	8753800	T	G	PC1	83.7223	Intron;SLC35A3;
8	rs_8_8753803	8753803	T	A	PC1	83.7223	Intron;SLC35A3;
8	rs_8_8841968	8841968	C	T	PC1	83.7223	AGL;downstream;
8	rs_8_8842415	8842415	T	C	PC1	83.7223	AGL;downstream;
8	rs_8_8843293	8843293	G	T	PC1	83.7223	AGL;downstream;
8	rs_8_8844227	8844227	A	C	PC1	83.7223	AGL;downstream;
8	rs_8_8844771	8844771	G	T	PC1	83.7223	AGL;downstream;
8	rs_8_8844854	8844854	C	T	PC1	83.7223	AGL;downstream;
8	rs_8_8851065	8851065	T	A	PC1	83.7223	Intron;AGL;
8	rs_8_8858654	8858654	T	C	PC1	83.7223	Intron;AGL;
8	rs_8_8858955	8858955	C	T	PC1	83.7223	Intron;AGL;
8	rs_8_8862919	8862919	C	T	PC1	83.7223	Intron;AGL;
8	rs_8_8864239	8864239	A	G	PC1	83.7223	Exon;AGL;AGL;-;2;TAT;Synonymous;Tyr;Tyr;
8	rs_8_8867055	8867055	A	G	PC1	83.7223	Intron;AGL;
8	rs_8_8869631	8869631	C	T	PC1	83.7223	Intron;AGL;
8	rs_8_8873577	8873577	G	A	PC1	83.7223	Intron;AGL;
8	rs_8_8875403	8875403	A	G	PC1	83.7223	Intron;AGL;
8	rs_8_8875899	8875899	C	T	PC1	83.7223	Intron;AGL;
8	rs_8_8879574	8879574	C	T	PC1	83.7223	AGL;upstream;
8	rs_8_8879618	8879618	T	C	PC1	83.7223	AGL;upstream;
8	rs_8_8879990	8879990	C	T	PC1	83.7223	AGL;upstream;
8	rs_8_8880248	8880248	A	G	PC1	83.7223	AGL;upstream;
8	rs_8_8881985	8881985	A	C	PC1	83.7223	AGL;upstream;
8	rs_8_8884894	8884894	T	C	PC1	83.7223	AGL;upstream;FRRS1;upstream;
8	rs_8_8885396	8885396	G	T	PC1	83.7223	AGL;upstream;FRRS1;upstream;
8	rs_8_8886125	8886125	T	A	PC1	83.7223	AGL;upstream;FRRS1;upstream;
8	rs_8_8886127	8886127	T	G	PC1	83.7223	AGL;upstream;FRRS1;upstream;
8	rs_8_8886979	8886979	T	C	PC1	83.7223	FRRS1;upstream;
8	rs_8_8888489	8888489	A	T	PC1	83.7223	FRRS1;upstream;
8	rs_8_8889174	8889174	A	G	PC1	83.7223	FRRS1;upstream;
8	rs_8_8913914	8913914	A	G	PC1	83.7223	Intron;PALMD;
8	rs_8_8914758	8914758	G	C	PC1	83.7223	Exon;PALMD;PALMD;-;2;CAC;Nonsynonymous;His;Gln;
8	rs_8_8919710	8919710	T	A	PC1	83.7223	Intron;PALMD;
8	rs_8_8920732	8920732	A	G	PC1	83.7223	Intron;PALMD;
8	rs_8_8929273	8929273	C	T	PC1	83.7223	Intron;PALMD;
8	rs_8_8930164	8930164	G	A	PC1	83.7223	Intron;PALMD;
8	rs_8_8931198	8931198	A	G	PC1	83.7223	Intron;PALMD;
8	rs_8_8931775	8931775	A	G	PC1	83.7223	Intron;PALMD;
8	rs_8_8935179	8935179	G	C	PC1	83.7223	PALMD;upstream;
8	rs_8_8938454	8938454	G	T	PC1	83.7223	PALMD;upstream;
8	rs_8_8938461	8938461	G	C	PC1	83.7223	PALMD;upstream;
8	rs_8_8939052	8939052	T	C	PC1	83.7223	PALMD;upstream;
8	rs_8_8939120	8939120	T	C	PC1	83.7223	PALMD;upstream;
8	rs_8_8942180	8942180	C	G	PC1	83.7223	PALMD;upstream;
8	rs_8_8942761	8942761	G	A	PC1	83.7223	PALMD;upstream;
8	rs_8_8944554	8944554	G	A	PC1	83.7223	Intergenic;
8	rs_8_8948253	8948253	T	C	PC1	83.7223	Intergenic;
8	rs_8_8949790	8949790	A	T	PC1	83.7223	Intergenic;
8	rs_8_8950167	8950167	C	T	PC1	83.7223	Intergenic;
8	rs_8_8951254	8951254	A	G	PC1	83.7223	Intergenic;
8	rs_8_8960755	8960755	C	T	PC1	83.7223	Intergenic;
8	rs_8_8960817	8960817	A	G	PC1	83.7223	Intergenic;
8	rs_8_8960833	8960833	T	C	PC1	83.7223	Intergenic;
8	rs_8_8961047	8961047	A	T	PC1	83.7223	Intergenic;
8	rs_8_8961130	8961130	A	G	PC1	83.7223	Intergenic;
8	rs_8_8961221	8961221	G	A	PC1	83.7223	Intergenic;
8	rs_8_8961300	8961300	A	G	PC1	83.7223	Intergenic;
8	rs_8_8961512	8961512	G	C	PC1	83.7223	Intergenic;
8	rs_8_8961537	8961537	A	G	PC1	83.7223	Intergenic;
8	rs_8_8962610	8962610	G	T	PC1	83.7223	Intergenic;
8	rs_8_8964881	8964881	C	T	PC1	83.7223	Intergenic;
8	rs_8_8968147	8968147	G	A	PC1	83.7223	Intergenic;
8	rs_8_8968626	8968626	G	A	PC1	83.7223	Intergenic;
8	rs_8_8969713	8969713	A	G	PC1	83.7223	Intergenic;

8	rs_8_8969993	8969993	T	C	PC1	83.7223	Intergenic;
8	rs_8_8970027	8970027	T	A	PC1	83.7223	Intergenic;
8	rs_8_8970238	8970238	G	A	PC1	83.7223	Intergenic;
8	rs_8_8971121	8971121	C	T	PC1	83.7223	Intergenic;
8	rs_8_8972743	8972743	G	A	PC1	83.7223	Intergenic;
8	rs_8_8973858	8973858	C	T	PC1	83.7223	Intergenic;
8	rs_8_8974879	8974879	A	G	PC1	83.7223	Intergenic;
8	rs_8_8974909	8974909	T	A	PC1	83.7223	Intergenic;
8	rs_8_8976361	8976361	G	A	PC1	83.7223	Intergenic;
8	rs_8_8976863	8976863	C	G	PC1	83.7223	Intergenic;
8	rs_8_8976988	8976988	G	A	PC1	83.7223	Intergenic;
8	rs_8_8977003	8977003	A	C	PC1	83.7223	Intergenic;
8	rs_8_8977619	8977619	A	G	PC1	83.7223	Intergenic;
8	rs_8_8977680	8977680	C	T	PC1	83.7223	Intergenic;
8	rs_8_8977692	8977692	C	T	PC1	83.7223	Intergenic;
8	rs_8_8977927	8977927	G	A	PC1	83.7223	Intergenic;
8	rs_8_8978362	8978362	C	T	PC1	83.7223	Intergenic;
8	rs_8_8980185	8980185	C	T	PC1	83.7223	Intergenic;
8	rs_8_8984727	8984727	A	G	PC1	83.7223	Intergenic;
8	rs_8_8992547	8992547	T	C	PC1	83.7223	Intergenic;
8	rs_8_8996672	8996672	G	C	PC1	83.7223	Intergenic;
8	rs_8_9003402	9003402	T	G	PC1	83.7223	Intergenic;
8	rs_8_9007968	9007968	T	G	PC1	83.7223	PLPPR4;downstream;
8	rs_8_9029661	9029661	G	A	PC1	83.7223	Intron;PLPPR4;
8	rs_8_9031069	9031069	A	G	PC1	83.7223	Intron;PLPPR4;
8	rs_8_9032326	9032326	C	G	PC1	83.7223	Intron;PLPPR4;
8	rs_8_9036839	9036839	T	C	PC1	83.7223	Intron;PLPPR4;
8	rs_8_9037159	9037159	G	T	PC1	83.7223	Intron;PLPPR4;
8	rs_8_9038873	9038873	T	C	PC1	83.7223	Intron;PLPPR4;
8	rs_8_9040129	9040129	T	C	PC1	83.7223	Intron;PLPPR4;
8	rs_8_9046665	9046665	T	C	PC1	83.7223	PLPPR4;upstream;
8	rs_8_9047857	9047857	T	C	PC1	83.7223	PLPPR4;upstream;
8	rs_8_9050348	9050348	G	A	PC1	83.7223	PLPPR4;upstream;
8	rs_8_9051847	9051847	G	A	PC1	83.7223	PLPPR4;upstream;
8	rs_8_9055364	9055364	C	T	PC1	83.7223	Intergenic;
8	rs_8_9057909	9057909	T	C	PC1	83.7223	Intergenic;
8	rs_8_9058184	9058184	G	A	PC1	83.7223	Intergenic;
8	rs_8_9058599	9058599	T	A	PC1	83.7223	Intergenic;
8	rs_8_9060405	9060405	G	A	PC1	83.7223	Intergenic;
8	rs_8_9061888	9061888	C	T	PC1	83.7223	Intergenic;
8	rs_8_9062343	9062343	C	G	PC1	83.7223	Intergenic;
8	rs_8_9062523	9062523	G	A	PC1	83.7223	Intergenic;
8	rs_8_9064643	9064643	T	G	PC1	83.7223	Intergenic;
8	rs_8_9066156	9066156	G	A	PC1	83.7223	Intergenic;
8	rs_8_9067024	9067024	C	T	PC1	83.7223	Intergenic;
8	rs_8_9069598	9069598	A	G	PC1	83.7223	Intergenic;
8	rs_8_9075273	9075273	T	G	PC1	83.7223	Intergenic;
8	rs_8_9078517	9078517	T	G	PC1	83.7223	Intergenic;
8	rs_8_9080480	9080480	A	G	PC1	83.7223	Intergenic;
8	rs_8_9085735	9085735	T	C	PC1	83.7223	Intergenic;
8	rs_8_9087430	9087430	G	A	PC1	83.7223	Intergenic;
8	rs_8_9089587	9089587	T	G	PC1	83.7223	Intergenic;
8	rs_8_9095266	9095266	C	T	PC1	83.7223	PLPPR5;upstream;
8	rs_8_9096848	9096848	G	A	PC1	83.7223	PLPPR5;upstream;
8	rs_8_9096895	9096895	C	T	PC1	83.7223	PLPPR5;upstream;
8	rs_8_9099319	9099319	A	G	PC1	83.7223	PLPPR5;upstream;
8	rs_8_9102613	9102613	T	C	PC1	83.7223	Intron;PLPPR5;
8	rs_8_9110237	9110237	A	G	PC1	83.7223	Intron;PLPPR5;
8	rs_8_9118248	9118248	T	G	PC1	83.7223	Intron;PLPPR5;
8	rs_8_9119951	9119951	A	G	PC1	83.7223	Intron;PLPPR5;
8	rs_8_9120980	9120980	G	A	PC1	83.7223	Intron;PLPPR5;
8	rs_8_9121459	9121459	T	C	PC1	83.7223	Intron;PLPPR5;
8	rs_8_9124523	9124523	A	C	PC1	83.7223	Intron;PLPPR5;
8	rs_8_9132667	9132667	G	A	PC1	83.7223	Intron;PLPPR5;
8	rs_8_9136838	9136838	C	T	PC1	83.7223	Intron;PLPPR5;
8	rs_8_9137612	9137612	T	C	PC1	83.7223	Intron;PLPPR5;
8	rs_8_9201646	9201646	T	C	PC1	83.7223	SNX7;upstream;
8	rs_8_9312039	9312039	C	T	PC1	83.7223	Intergenic;
8	rs_8_9326280	9326280	T	C	PC1	83.7223	Intergenic;
8	rs_8_9688247	9688247	T	C	PC1	83.7223	DPYD;downstream;
8	rs_8_9698261	9698261	C	T	PC1	83.7223	Intergenic;
8	rs_8_9698402	9698402	C	T	PC1	83.7223	Intergenic;
8	rs_8_9699156	9699156	A	G	PC1	83.7223	Intergenic;
8	rs_8_9700057	9700057	A	C	PC1	83.7223	Intergenic;
8	rs_8_9700342	9700342	C	T	PC1	83.7223	Intergenic;
8	rs_8_9700996	9700996	A	G	PC1	83.7223	Intergenic;
8	rs_8_9701192	9701192	G	A	PC1	83.7223	Intergenic;
8	rs_8_9701604	9701604	G	A	PC1	83.7223	Intergenic;
8	rs_8_9701757	9701757	C	T	PC1	83.7223	Intergenic;
8	rs_8_9703549	9703549	T	G	PC1	83.7223	Intergenic;
8	rs_8_9704687	9704687	G	A	PC1	83.7223	Intergenic;
8	rs_8_9704926	9704926	C	T	PC1	83.7223	Intergenic;
8	rs_8_9705273	9705273	A	G	PC1	83.7223	Intergenic;
8	rs_8_9705613	9705613	G	A	PC1	83.7223	Intergenic;
8	rs_8_9706193	9706193	G	C	PC1	83.7223	Intergenic;
8	rs_8_9706195	9706195	A	G	PC1	83.7223	Intergenic;
8	rs_8_9707195	9707195	G	A	PC1	83.7223	Intergenic;
8	rs_8_9710912	9710912	G	T	PC1	83.7223	Intergenic;

8	rs_8_9714412	9714412	A	G	PC1	83.7223	Intergenic;
8	rs_8_9722350	9722350	T	C	PC1	83.7223	Intergenic;
8	rs_8_9722895	9722895	G	A	PC1	83.7223	Intergenic;
8	rs_8_9725497	9725497	A	G	PC1	83.7223	Intergenic;
8	rs_8_9726548	9726548	T	G	PC1	83.7223	Intergenic;
8	rs_8_9743345	9743345	C	T	PC1	83.7223	PTBP2;downstream;
8	rs_8_10121150	10121150	C	T	PC1	83.7223	Intergenic;
8	rs_8_10121192	10121192	A	G	PC1	83.7223	Intergenic;
8	rs_8_10137727	10137727	A	C	PC1	83.7223	HCCS;upstream;TLCD4-B;downstream;
8	rs_8_10140605	10140605	C	T	PC1	83.7223	Intron;TLCD4-B;
8	rs_8_10142508	10142508	T	C	PC1	83.7223	Intron;TLCD4-B;
8	rs_8_10144535	10144535	A	G	PC1	83.7223	Intron;TLCD4-B;
8	rs_8_10144753	10144753	C	T	PC1	83.7223	Intron;TLCD4-B;
8	rs_8_10144815	10144815	G	T	PC1	83.7223	Intron;TLCD4-B;
8	rs_8_10144944	10144944	C	T	PC1	83.7223	Intron;TLCD4-B;
8	rs_8_10146590	10146590	A	G	PC1	83.7223	Intron;TLCD4-B;
8	rs_8_10150266	10150266	C	G	PC1	83.7223	Intron;TLCD4-B;
8	rs_8_10153813	10153813	C	T	PC1	83.7223	TLCD4-B;upstream;
8	rs_8_10154362	10154362	A	G	PC1	83.7223	TLCD4-B;upstream;
8	rs_8_10163566	10163566	A	C	PC1	83.7223	TLCD4-B;upstream;
8	rs_8_10164717	10164717	T	A	PC1	83.7223	ALG14;upstream;
8	rs_8_10171448	10171448	C	T	PC1	83.7223	ALG14;upstream;
8	rs_8_10327303	10327303	A	G	PC1	83.7223	Intergenic;
8	rs_8_10334561	10334561	G	A	PC1	83.7223	Intron;F3;
8	rs_8_10335592	10335592	G	A	PC1	83.7223	Exon;F3;F3;+;0;GTG;Nonsynonymous;Val;Met;
8	rs_8_10336073	10336073	C	T	PC1	83.7223	Intron;F3;
8	rs_8_10337767	10337767	C	G	PC1	83.7223	Intergenic;
8	rs_8_10344308	10344308	A	G	PC1	83.7223	F3;downstream;ABCD3;downstream;
8	rs_8_10344796	10344796	C	T	PC1	83.7223	F3;downstream;ABCD3;downstream;
8	rs_8_10345455	10345455	C	T	PC1	83.7223	F3;downstream;ABCD3;downstream;
8	rs_8_10376768	10376768	G	A	PC1	83.7223	ABCD3;upstream;
8	rs_8_10391966	10391966	A	G	PC1	83.7223	Intergenic;
8	rs_8_10392698	10392698	C	T	PC1	83.7223	ARHGAP29;upstream;
8	rs_8_10392712	10392712	T	C	PC1	83.7223	ARHGAP29;upstream;
8	rs_8_10393062	10393062	A	G	PC1	83.7223	ARHGAP29;upstream;
8	rs_8_10401199	10401199	A	G	PC1	83.7223	ARHGAP29;upstream;
8	rs_8_10402574	10402574	T	C	PC1	83.7223	Intron;ARHGAP29;
8	rs_8_10402635	10402635	G	A	PC1	83.7223	Intron;ARHGAP29;
8	rs_8_10402819	10402819	A	G	PC1	83.7223	Intron;ARHGAP29;
8	rs_8_10403124	10403124	G	T	PC1	83.7223	Intron;ARHGAP29;
8	rs_8_10403941	10403941	G	A	PC1	83.7223	Intron;ARHGAP29;
8	rs_8_10404028	10404028	T	G	PC1	83.7223	Intron;ARHGAP29;
8	rs_8_10404689	10404689	C	G	PC1	83.7223	Intron;ARHGAP29;
8	rs_8_10405615	10405615	A	G	PC1	83.7223	Intron;ARHGAP29;
8	rs_8_10405785	10405785	A	G	PC1	83.7223	Intron;ARHGAP29;
8	rs_8_10406725	10406725	C	T	PC1	83.7223	Intron;ARHGAP29;
8	rs_8_10407323	10407323	G	A	PC1	83.7223	Intron;ARHGAP29;
8	rs_8_10408101	10408101	C	T	PC1	83.7223	Intron;ARHGAP29;
8	rs_8_10408159	10408159	G	T	PC1	83.7223	Intron;ARHGAP29;
8	rs_8_10408902	10408902	C	T	PC1	83.7223	Intron;ARHGAP29;
8	rs_8_10409748	10409748	G	A	PC1	83.7223	Intron;ARHGAP29;
8	rs_8_10411081	10411081	T	C	PC1	83.7223	Intron;ARHGAP29;
8	rs_8_10411925	10411925	G	A	PC1	83.7223	Intron;ARHGAP29;
8	rs_8_10412325	10412325	T	C	PC1	83.7223	Intron;ARHGAP29;
8	rs_8_10412936	10412936	T	C	PC1	83.7223	Intron;ARHGAP29;
8	rs_8_10413651	10413651	C	T	PC1	83.7223	Intron;ARHGAP29;
8	rs_8_10413934	10413934	T	C	PC1	83.7223	Intron;ARHGAP29;
8	rs_8_10414033	10414033	G	A	PC1	83.7223	Intron;ARHGAP29;
8	rs_8_10414163	10414163	T	G	PC1	83.7223	Intron;ARHGAP29;
8	rs_8_10415613	10415613	C	T	PC1	83.7223	Intron;ARHGAP29;
8	rs_8_10415627	10415627	C	T	PC1	83.7223	Intron;ARHGAP29;
8	rs_8_10416037	10416037	G	A	PC1	83.7223	Intron;ARHGAP29;
8	rs_8_10416057	10416057	T	C	PC1	83.7223	Intron;ARHGAP29;
8	rs_8_10416183	10416183	T	G	PC1	83.7223	Intron;ARHGAP29;
8	rs_8_10418487	10418487	G	T	PC1	83.7223	Intron;ARHGAP29;
8	rs_8_10422500	10422500	T	C	PC1	83.7223	Intron;ARHGAP29;
8	rs_8_10422779	10422779	C	G	PC1	83.7223	Intron;ARHGAP29;
8	rs_8_10428092	10428092	T	C	PC1	83.7223	Intron;ARHGAP29;
8	rs_8_10430208	10430208	T	A	PC1	83.7223	Intron;ARHGAP29;
8	rs_8_10431201	10431201	C	T	PC1	83.7223	Intron;ARHGAP29;
8	rs_8_10443895	10443895	C	G	PC1	83.7223	Intron;ARHGAP29;
8	rs_8_10444714	10444714	A	G	PC1	83.7223	Intron;ARHGAP29;
8	rs_8_10452600	10452600	A	C	PC1	83.7223	ARHGAP29;downstream;ABCA4;upstream;
8	rs_8_10453228	10453228	C	T	PC1	83.7223	ARHGAP29;downstream;ABCA4;upstream;
8	rs_8_10454334	10454334	G	A	PC1	83.7223	ARHGAP29;downstream;ABCA4;upstream;
8	rs_8_10454417	10454417	G	A	PC1	83.7223	ARHGAP29;downstream;ABCA4;upstream;
8	rs_8_10454665	10454665	T	C	PC1	83.7223	ARHGAP29;downstream;ABCA4;upstream;
8	rs_8_10455090	10455090	G	A	PC1	83.7223	ARHGAP29;downstream;ABCA4;upstream;
8	rs_8_10455192	10455192	G	A	PC1	83.7223	ARHGAP29;downstream;ABCA4;upstream;
8	rs_8_10456033	10456033	G	C	PC1	83.7223	ARHGAP29;downstream;ABCA4;upstream;
8	rs_8_10456066	10456066	T	C	PC1	83.7223	ARHGAP29;downstream;ABCA4;upstream;
8	rs_8_10457266	10457266	C	T	PC1	83.7223	ARHGAP29;downstream;ABCA4;upstream;
8	rs_8_10458197	10458197	A	G	PC1	83.7223	ARHGAP29;downstream;ABCA4;upstream;
8	rs_8_10458551	10458551	C	A	PC1	83.7223	ARHGAP29;downstream;ABCA4;upstream;
8	rs_8_10460181	10460181	A	C	PC1	83.7223	ARHGAP29;downstream;ABCA4;upstream;
8	rs_8_10460838	10460838	T	A	PC1	83.7223	ARHGAP29;downstream;ABCA4;upstream;
8	rs_8_10460908	10460908	G	A	PC1	83.7223	ARHGAP29;downstream;ABCA4;upstream;
8	rs_8_10461304	10461304	G	T	PC1	83.7223	Intron;ABCA4;

8	rs_8_10462763	10462763	G	A	PC1	83.7223	Intron;ABCA4;
8	rs_8_10462879	10462879	G	A	PC1	83.7223	Intron;ABCA4;
8	rs_8_10466566	10466566	G	A	PC1	83.7223	Intron;ABCA4;
8	rs_8_10467891	10467891	T	C	PC1	83.7223	Intron;ABCA4;
8	rs_8_10469044	10469044	C	G	PC1	83.7223	Intron;ABCA4;
8	rs_8_10469453	10469453	C	T	PC1	83.7223	Intron;ABCA4;
8	rs_8_10472334	10472334	T	C	PC1	83.7223	Intron;ABCA4;
8	rs_8_10475564	10475564	C	T	PC1	83.7223	Intron;ABCA4;
8	rs_8_10476504	10476504	A	G	PC1	83.7223	Intron;ABCA4;
8	rs_8_10476540	10476540	T	G	PC1	83.7223	Intron;ABCA4;
8	rs_8_10477823	10477823	G	A	PC1	83.7223	Intron;ABCA4;
8	rs_8_10478470	10478470	C	A	PC1	83.7223	Intron;ABCA4;
8	rs_8_10480012	10480012	A	T	PC1	83.7223	Intron;ABCA4;
8	rs_8_10482909	10482909	C	A	PC1	83.7223	Intron;ABCA4;
8	rs_8_10483298	10483298	T	C	PC1	83.7223	Intron;ABCA4;
8	rs_8_10484003	10484003	T	A	PC1	83.7223	Intron;ABCA4;
8	rs_8_10484383	10484383	C	T	PC1	83.7223	Intron;ABCA4;
8	rs_8_10484793	10484793	A	G	PC1	83.7223	Intron;ABCA4;
8	rs_8_10487991	10487991	T	A	PC1	83.7223	Intron;ABCA4;
8	rs_8_10488039	10488039	T	C	PC1	83.7223	Intron;ABCA4;
8	rs_8_10488523	10488523	A	T	PC1	83.7223	Intron;ABCA4;
8	rs_8_10493385	10493385	C	T	PC1	83.7223	Intron;ABCA4;
8	rs_8_10496311	10496311	T	C	PC1	83.7223	Intron;ABCA4;
8	rs_8_10504985	10504985	G	C	PC1	83.7223	Intron;ABCA4;
8	rs_8_10527720	10527720	C	T	PC1	83.7223	Exon;ABCA4;ABCA4;+;2;ACC;Synonymous;Thr;Thr;
8	rs_8_10527873	10527873	A	G	PC1	83.7223	Intron;ABCA4;
8	rs_8_10532200	10532200	T	C	PC1	83.7223	Intron;ABCA4;
8	rs_8_10534570	10534570	T	C	PC1	83.7223	ABCA4;downstream;TECR;downstream;
8	rs_8_10535571	10535571	A	G	PC1	83.7223	ABCA4;downstream;TECR;downstream;
8	rs_8_10537642	10537642	C	T	PC1	83.7223	Intron;TECR;
8	rs_8_10541816	10541816	T	G	PC1	83.7223	Intron;TECR;
8	rs_8_10541822	10541822	C	G	PC1	83.7223	Intron;TECR;
8	rs_8_10551269	10551269	C	T	PC1	83.7223	Intron;TECR;
8	rs_8_10617816	10617816	G	A	PC1	83.7223	Intergenic;
8	rs_8_10634623	10634623	C	T	PC1	83.7223	Intergenic;
8	rs_8_10645085	10645085	G	A	PC1	83.7223	Intergenic;
8	rs_8_10657835	10657835	G	A	PC1	83.7223	FER;upstream;
8	rs_8_10660732	10660732	C	G	PC1	83.7223	FER;upstream;
8	rs_8_10663383	10663383	T	C	PC1	83.7223	Intron;FER;
8	rs_8_10663663	10663663	C	T	PC1	83.7223	Intron;FER;
8	rs_8_10668494	10668494	C	T	PC1	83.7223	Intron;FER;
8	rs_8_10674239	10674239	A	C	PC1	83.7223	Intron;FER;
8	rs_8_10691358	10691358	C	T	PC1	83.7223	Intron;FNBP1L;
8	rs_8_10703694	10703694	G	A	PC1	83.7223	Intron;FNBP1L;
8	rs_8_10703934	10703934	G	A	PC1	83.7223	Intron;FNBP1L;
8	rs_8_10707727	10707727	C	T	PC1	83.7223	Intron;FNBP1L;
8	rs_8_10715219	10715219	T	A	PC1	83.7223	Intron;FNBP1L;
8	rs_8_10716271	10716271	G	C	PC1	83.7223	Intron;FNBP1L;
8	rs_8_10723172	10723172	C	G	PC1	83.7223	Intron;FNBP1L;
8	rs_8_10726563	10726563	G	A	PC1	83.7223	Intron;FNBP1L;
8	rs_8_10734095	10734095	C	T	PC1	83.7223	FNBP1L;upstream;DR1;downstream;
8	rs_8_10864572	10864572	G	A	PC1	83.7223	Exon;RPL5;RPL5;-;2;TAC;Synonymous;Tyr;Tyr;
8	rs_8_11494411	11494411	T	C	PC1	83.7223	Intergenic;
8	rs_8_11497433	11497433	T	C	PC1	83.7223	Intergenic;
8	rs_8_12117757	12117757	T	C	PC1	83.7223	Intergenic;
8	rs_8_12465603	12465603	G	A	PC1	83.7223	Intergenic;
8	rs_8_12951930	12951930	A	G	PC1	83.7223	Intron;COL24A1;
8	rs_8_12969897	12969897	C	T	PC1	83.7223	Intron;COL24A1;
8	rs_8_13012600	13012600	G	A	PC1	83.7223	Intron;ZNHIT6;
8	rs_8_13075857	13075857	A	G	PC1	83.7223	DDAH1;upstream;
8	rs_8_13079672	13079672	G	C	PC1	83.7223	DDAH1;upstream;
8	rs_8_13092104	13092104	A	G	PC1	83.7223	Intron;DDAH1;
8	rs_8_13092717	13092717	A	G	PC1	83.7223	Intron;DDAH1;
8	rs_8_13095695	13095695	C	T	PC1	83.7223	Intron;DDAH1;
8	rs_8_13107307	13107307	C	T	PC1	83.7223	Intron;DDAH1;
8	rs_8_13125359	13125359	G	A	PC1	83.7223	Intron;DDAH1;
8	rs_8_13136943	13136943	C	T	PC1	83.7223	DDAH1;downstream;
8	rs_8_13138255	13138255	C	G	PC1	83.7223	DDAH1;downstream;C1ORF52;upstream;
8	rs_8_13280423	13280423	G	C	PC1	83.7223	Intron;SSX2IP;
8	rs_8_13285682	13285682	A	G	PC1	83.7223	Intron;SSX2IP;
8	rs_8_13287069	13287069	G	A	PC1	83.7223	Intron;SSX2IP;
8	rs_8_13289079	13289079	C	G	PC1	83.7223	Intron;SSX2IP;
8	rs_8_13292865	13292865	A	G	PC1	83.7223	Exon;SSX2IP;SSX2IP;-;0;TCA;Nonsynonymous;Ser;Pro;
8	rs_8_13315547	13315547	A	C	PC1	83.7223	Intron;SSX2IP;
8	rs_8_13316404	13316404	T	C	PC1	83.7223	Intron;SSX2IP;
8	rs_8_13319435	13319435	C	T	PC1	83.7223	SSX2IP;upstream;
8	rs_8_13334309	13334309	T	C	PC1	83.7223	LPAR3;downstream;
8	rs_8_13335744	13335744	A	G	PC1	83.7223	LPAR3;downstream;
8	rs_8_13336759	13336759	T	A	PC1	83.7223	LPAR3;downstream;
8	rs_8_13338907	13338907	G	A	PC1	83.7223	LPAR3;downstream;
8	rs_8_13339706	13339706	A	C	PC1	83.7223	LPAR3;downstream;
8	rs_8_13341169	13341169	G	A	PC1	83.7223	Intergenic;
8	rs_8_13350835	13350835	T	C	PC1	83.7223	Intergenic;
8	rs_8_13353229	13353229	A	G	PC1	83.7223	LPAR3;upstream;MCOLN3;downstream;
8	rs_8_13353775	13353775	T	C	PC1	83.7223	LPAR3;upstream;MCOLN3;downstream;
8	rs_8_13356763	13356763	T	C	PC1	83.7223	LPAR3;upstream;MCOLN3;downstream;
8	rs_8_13380210	13380210	G	C	PC1	83.7223	Intron;MCOLN3;
8	rs_8_13381208	13381208	C	T	PC1	83.7223	Intron;MCOLN3;

8	rs_8_13393668	13393668	T	A	PC1	83.7223	Intron;MCOLN3;
8	rs_8_13399425	13399425	A	C	PC1	83.7223	MCOLN3;upstream;WDR63;upstream;
8	rs_8_13402510	13402510	C	T	PC1	83.7223	Intron;WDR63;
8	rs_8_13413494	13413494	G	T	PC1	83.7223	Exon;WDR63;WDR63;+;0;GCT;Nonsynonymous;Ala;Ser;
8	rs_8_13415325	13415325	T	C	PC1	83.7223	Intron;WDR63;
8	rs_8_13416689	13416689	G	C	PC1	83.7223	Intron;WDR63;
8	rs_8_13417161	13417161	T	C	PC1	83.7223	Intron;WDR63;
8	rs_8_13419344	13419344	C	A	PC1	83.7223	Intron;WDR63;
8	rs_8_13422273	13422273	A	G	PC1	83.7223	Intron;WDR63;
8	rs_8_13422713	13422713	C	T	PC1	83.7223	Intron;WDR63;
8	rs_8_13423322	13423322	T	C	PC1	83.7223	Intron;WDR63;
8	rs_8_13424716	13424716	G	C	PC1	83.7223	Intron;WDR63;
8	rs_8_13425489	13425489	A	G	PC1	83.7223	Intron;WDR63;
8	rs_8_13426172	13426172	G	A	PC1	83.7223	Intron;WDR63;
8	rs_8_13426241	13426241	C	T	PC1	83.7223	Intron;WDR63;
8	rs_8_13427412	13427412	C	T	PC1	83.7223	Intron;WDR63;
8	rs_8_13429131	13429131	T	C	PC1	83.7223	WDR63;downstream;
8	rs_8_13430461	13430461	T	C	PC1	83.7223	WDR63;downstream;
8	rs_8_13478265	13478265	A	G	PC1	83.7223	Intron;VTG2;
8	rs_8_13478479	13478479	G	A	PC1	83.7223	Exon;VTG2;VTG2;+;0;GTG;Nonsynonymous;Val;Met;
8	rs_8_13480004	13480004	G	A	PC1	83.7223	Intron;VTG2;
8	rs_8_13482963	13482963	T	C	PC1	83.7223	Exon;VTG2;VTG2;+;2;TGT;Synonymous;Cys;Cys;
8	rs_8_13483150	13483150	A	G	PC1	83.7223	Intron;VTG2;
8	rs_8_13483255	13483255	T	C	PC1	83.7223	Intron;VTG2;
8	rs_8_13489963	13489963	A	G	PC1	83.7223	Intron;VTG2;
8	rs_8_13492638	13492638	G	A	PC1	77.99387	Intron;VTG2;
8	rs_8_13495345	13495345	G	A	PC1	83.7223	Intron;VTG2;
8	rs_8_13496898	13496898	C	G	PC1	83.7223	Exon;CTBS;CTBS;+;2;AAC;Nonsynonymous;Asn;Lys;
8	rs_8_13497097	13497097	A	G	PC1	83.7223	Intron;CTBS;
8	rs_8_13500949	13500949	G	T	PC1	83.7223	Intron;CTBS;
8	rs_8_13502505	13502505	A	G	PC1	83.7223	VTG2;downstream;SPATA1;downstream;
8	rs_8_13503565	13503565	G	T	PC1	83.7223	Exon;SPATA1;SPATA1;-;2;TCC;Synonymous;Ser;Ser;
8	rs_8_13512315	13512315	G	C	PC1	83.7223	Intron;SPATA1;
8	rs_8_13516925	13516925	G	C	PC1	83.7223	SPATA1;upstream;RPF1;downstream;
8	rs_8_13518719	13518719	C	T	PC1	83.7223	SPATA1;upstream;RPF1;downstream;
8	rs_8_13519893	13519893	G	T	PC1	83.7223	SPATA1;upstream;RPF1;downstream;
8	rs_8_13521639	13521639	T	C	PC1	83.7223	Intron;GNG5;
8	rs_8_13521708	13521708	T	C	PC1	83.7223	Intron;GNG5;
8	rs_8_13530185	13530185	G	C	PC1	83.7223	Intron;RPF1;
8	rs_8_13531315	13531315	G	C	PC1	83.7223	Intron;RPF1;
8	rs_8_13532423	13532423	A	G	PC1	83.7223	Intron;RPF1;
8	rs_8_13539307	13539307	A	G	PC1	83.7223	Intron;RPF1;
8	rs_8_13541067	13541067	C	T	PC1	83.7223	Intron;RPF1;
8	rs_8_13545351	13545351	T	C	PC1	83.7223	Intron;RPF1;
8	rs_8_13545696	13545696	G	A	PC1	83.7223	Intron;RPF1;
8	rs_8_13546740	13546740	T	C	PC1	83.7223	Intron;RPF1;
8	rs_8_13549322	13549322	G	A	PC1	83.7223	RPF1;upstream;
8	rs_8_13559891	13559891	T	C	PC1	83.7223	Intergenic;
8	rs_8_13560018	13560018	T	C	PC1	83.7223	Intergenic;
8	rs_8_13561157	13561157	T	C	PC1	83.7223	Intron;UOX;
8	rs_8_13564947	13564947	A	G	PC1	83.7223	Intron;UOX;
8	rs_8_13565726	13565726	C	T	PC1	83.7223	Intron;UOX;
8	rs_8_13569110	13569110	T	C	PC1	83.7223	DNASE2B;upstream;SAMD13;downstream;
8	rs_8_13570960	13570960	C	T	PC1	83.7223	UOX;downstream;SAMD13;downstream;
8	rs_8_13578955	13578955	A	T	PC1	83.7223	Intron;SAMD13;
8	rs_8_13590513	13590513	T	C	PC1	83.7223	SAMD13;upstream;PRKACB;downstream;
8	rs_8_13591805	13591805	T	C	PC1	83.7223	SAMD13;upstream;PRKACB;downstream;
8	rs_8_13743887	13743887	C	G	PC1	83.7223	Exon;TTLL7;TTLL7;+;2;GAC;Nonsynonymous;Asp;Glu;
8	rs_8_13830361	13830361	G	T	PC1	83.7223	Intergenic;
8	rs_8_13854024	13854024	C	T	PC1	83.7223	Intergenic;
8	rs_8_14475358	14475358	C	T	PC1	83.7223	Intergenic;
8	rs_8_15880826	15880826	T	C	PC1	83.7223	Intron;NEXN;
8	rs_8_16219494	16219494	T	C	PC1	83.7223	Intron;ST6GALNAC5;
8	rs_8_16338572	16338572	C	T	PC1	83.7223	Intron;ST6GALNAC3;
8	rs_8_16338743	16338743	G	A	PC1	83.7223	Intron;ST6GALNAC3;
8	rs_8_16340006	16340006	C	T	PC1	83.7223	Intron;ST6GALNAC3;
8	rs_8_16340014	16340014	C	G	PC1	83.7223	Intron;ST6GALNAC3;
8	rs_8_16340197	16340197	T	A	PC1	83.7223	Intron;ST6GALNAC3;
8	rs_8_16341138	16341138	T	C	PC1	83.7223	Intron;ST6GALNAC3;
8	rs_8_16341632	16341632	A	G	PC1	83.7223	Intron;ST6GALNAC3;
8	rs_8_16353223	16353223	A	G	PC1	83.7223	Intron;ST6GALNAC3;
8	rs_8_16359386	16359386	C	A	PC1	83.7223	Intron;ST6GALNAC3;
8	rs_8_16360725	16360725	T	C	PC1	83.7223	Intron;ST6GALNAC3;
8	rs_8_16361502	16361502	C	T	PC1	83.7223	Intron;ST6GALNAC3;
8	rs_8_16363531	16363531	C	T	PC1	83.7223	Intron;ST6GALNAC3;
8	rs_8_24035077	24035077	T	G	PC1	83.7223	Intergenic;
8	rs_8_24417588	24417588	G	A	PC1	83.7223	Intron;NFIA;
8	rs_8_25129105	25129105	T	G	PC1	83.7223	Intron;ALG6;
8	rs_8_26535043	26535043	G	A	PC2	117.082	Intergenic;
8	rs_8_26535062	26535062	T	A	PC2	88.41587	Intergenic;
8	rs_8_26535065	26535065	T	A	PC2	88.41587	Intergenic;
8	rs_8_26535114	26535114	G	A	PC2	88.41587	Intergenic;
8	rs_8_26535139	26535139	C	T	PC2	88.41587	Intergenic;
8	rs_8_26535157	26535157	C	T	PC2	88.41587	Intergenic;
8	rs_8_26535158	26535158	A	G	PC2	78.79307	Intergenic;
8	rs_8_26537707	26537707	G	A	PC2	112.8336	Intergenic;
8	rs_8_26541919	26541919	T	C	PC2	96.74259	Intergenic;
8	rs_8_26541956	26541956	C	T	PC2	96.74259	Intergenic;

8	rs_8_26602636	26602636	A	G	PC2	85.54607	Intron;NEGR1;
8	rs_8_26607587	26607587	G	A	PC2	83.9828	Intron;NEGR1;
8	rs_8_27625538	27625538	T	C	PC1	83.7223	FMO3;upstream;PRRX1;downstream;
8	rs_8_27629246	27629246	G	T	PC1	83.7223	Intron;PRRX1;
8	rs_8_27629694	27629694	C	T	PC1	83.7223	Intron;PRRX1;
8	rs_8_27633564	27633564	A	G	PC1	83.7223	Intron;PRRX1;
8	rs_8_27634063	27634063	A	G	PC1	83.7223	Intron;PRRX1;
8	rs_8_27634536	27634536	G	A	PC1	83.7223	Intron;PRRX1;
8	rs_8_27636012	27636012	T	C	PC1	83.7223	Intron;PRRX1;
8	rs_8_27642471	27642471	T	G	PC1	83.7223	Intron;PRRX1;
8	rs_8_27642611	27642611	G	A	PC1	83.7223	Intron;PRRX1;
8	rs_8_27643454	27643454	A	G	PC1	77.99387	Intron;PRRX1;
8	rs_8_30301669	30301669	A	G	PC2	79.00075	Intron;LAMC1;
8	rs_8_30303384	30303384	G	A	PC2	104.5075	Intron;LAMC1;
8	rs_8_30311430	30311430	A	T	PC2	104.5075	Intron;LAMC1;
8	rs_8_30319720	30319720	G	T	PC2	99.02893	Intron;LAMC1;
8	rs_8_30417264	30417264	A	G	PC2	78.27507	Intron;SMG7;
8	rs_8_30441677	30441677	T	G	PC2	82.70778	Intron;SMG7;
8	rs_8_30453675	30453675	A	G	PC2	76.58372	Intron;NCF2;
8	rs_8_30454675	30454675	T	C	PC2	76.58372	Intron;NCF2;
8	rs_8_30454703	30454703	G	C	PC2	76.58372	Intron;NCF2;
8	rs_8_30454718	30454718	T	C	PC2	76.58372	Intron;NCF2;
8	rs_8_30536244	30536244	C	T	PC2	76.87849	Intron;RGL1;
9	rs_9_1762562	1762562	C	T	PC2	77.10366	Intergenic;
9	rs_9_1762571	1762571	T	A	PC2	79.67288	Intergenic;
9	rs_9_1763895	1763895	C	T	PC2	79.67288	Intergenic;
9	rs_9_1947981	1947981	C	T	PC2	129.8614	Intron;CLK3;
9	rs_9_1952335	1952335	A	G	PC2	95.21785	Intron;CLK3;
9	rs_9_1952710	1952710	A	G	PC2	80.50709	Intron;CLK3;
9	rs_9_4320423	4320423	G	A	PC2	100.1102	Intergenic;
9	rs_9_4321628	4321628	C	T	PC2	100.1102	Intergenic;
9	rs_9_4325273	4325273	T	C	PC2	100.1102	Intergenic;
9	rs_9_4325281	4325281	C	T	PC2	100.1102	Intergenic;
9	rs_9_4325350	4325350	G	T	PC2	100.1102	Intergenic;
9	rs_9_4326339	4326339	C	T	PC2	100.1102	Intergenic;
9	rs_9_4332404	4332404	A	T	PC2	78.19788	Intergenic;
9	rs_9_4336775	4336775	C	T	PC2	89.30023	Intergenic;
9	rs_9_4338203	4338203	G	C	PC2	89.30023	Intergenic;
9	rs_9_4339214	4339214	T	C	PC2	89.30023	Intergenic;
9	rs_9_4343459	4343459	G	A	PC2	96.91934	Intergenic;
9	rs_9_4347407	4347407	T	A	PC2	89.30023	Intergenic;
9	rs_9_4347677	4347677	G	A	PC2	89.30023	Intergenic;
9	rs_9_4349659	4349659	A	G	PC2	89.30023	Intergenic;
9	rs_9_4354433	4354433	A	G	PC2	79.036	Intergenic;
9	rs_9_4358006	4358006	G	C	PC2	79.036	Intergenic;
9	rs_9_4367107	4367107	C	T	PC2	94.34745	Intergenic;
9	rs_9_4368621	4368621	T	C	PC2	78.19788	Intergenic;
9	rs_9_4368696	4368696	A	G	PC2	89.30023	Intergenic;
9	rs_9_4371277	4371277	T	G	PC2	78.07288	Intergenic;
9	rs_9_4374405	4374405	T	C	PC2	79.4354	Intergenic;
9	rs_9_4374956	4374956	A	G	PC2	89.30023	Intergenic;
9	rs_9_4381817	4381817	G	A	PC2	76.79927	Intergenic;
9	rs_9_4388623	4388623	A	G	PC2	81.33948	Intergenic;
9	rs_9_4391829	4391829	A	G	PC2	89.30023	Intergenic;
9	rs_9_4426755	4426755	C	A	PC2	89.30023	Intergenic;
9	rs_9_4444901	4444901	C	A	PC2	89.30023	Intergenic;
9	rs_9_4444902	4444902	T	G	PC2	89.30023	Intergenic;
9	rs_9_4445960	4445960	C	T	PC2	78.33056	Intergenic;
9	rs_9_4446850	4446850	G	A	PC2	78.97231	Intergenic;
9	rs_9_4448950	4448950	T	C	PC2	89.30023	Intergenic;
9	rs_9_4452246	4452246	C	A	PC2	89.30023	Intergenic;
9	rs_9_4456941	4456941	T	A	PC2	78.48766	Intergenic;
9	rs_9_4458304	4458304	G	A	PC2	78.19788	Intergenic;
9	rs_9_4461740	4461740	A	G	PC2	89.30023	Intergenic;
9	rs_9_4462987	4462987	A	G	PC2	89.30023	Intergenic;
9	rs_9_4470255	4470255	T	C	PC2	78.24782	Intergenic;
9	rs_9_4478973	4478973	T	A	PC2	78.24782	Intergenic;
9	rs_9_4480472	4480472	C	T	PC2	78.24782	RORA;upstream;
9	rs_9_4486199	4486199	C	G	PC2	78.24782	RORA;upstream;
9	rs_9_4486421	4486421	G	A	PC2	78.24782	RORA;upstream;
9	rs_9_4501757	4501757	C	T	PC2	87.21447	Intron;RORA;
9	rs_9_4503873	4503873	A	C	PC2	78.24782	Intron;RORA;
9	rs_9_4506726	4506726	A	G	PC2	89.30023	Intron;RORA;
9	rs_9_4513368	4513368	A	G	PC2	79.00928	Intron;RORA;
9	rs_9_4513370	4513370	A	G	PC2	79.00928	Intron;RORA;
9	rs_9_4513431	4513431	C	A	PC2	79.00928	Intron;RORA;
9	rs_9_4516805	4516805	A	G	PC2	83.0138	Intron;RORA;
9	rs_9_10799701	10799701	A	T	PC1	77.99387	Intron;PDE8A;
9	rs_9_20330995	20330995	T	C	PC1	77.99387	TP53BP1;downstream;TUBGCP4;downstream;
9	rs_9_20351418	20351418	T	C	PC1	77.99387	TUBGCP4;upstream;IQGAP1;upstream;
10	rs_10_1068150{	10681508	T	C	Others	106.3041	Intron;CFAP100;
11	rs_11_333160	333160	C	G	PC1	83.7223	ZFHX3;downstream;
11	rs_11_349290	349290	A	G	PC1	83.7223	Exon;ZFHX3;ZFHX3;-;2;AGT;Synonymous;Ser;Ser;
11	rs_11_4110195	4110195	C	T	PC2	77.66844	Intergenic;
11	rs_11_4110908	4110908	A	G	PC2	89.4237	Intergenic;
11	rs_11_4128424	4128424	A	G	PC2	76.93143	Intergenic;
11	rs_11_4275481	4275481	C	T	PC2	76.53192	HSD17B2;upstream;
11	rs_11_4336378	4336378	A	T	PC2	116.4811	Intron;PLCG2;

11	rs_11_4343959	4343959	A	G	PC2	133.3067	Intron;PLCG2;
11	rs_11_4344590	4344590	A	G	PC2	122.9318	Intron;PLCG2;
11	rs_11_4346638	4346638	C	T	PC2	160.3058	Intron;PLCG2;
11	rs_11_4365567	4365567	C	T	PC2	88.85091	CMIP;downstream;
11	rs_11_4366332	4366332	C	T	PC2	88.85091	CMIP;downstream;
11	rs_11_4368290	4368290	C	T	PC2	88.85091	CMIP;downstream;
11	rs_11_4369756	4369756	T	C	PC2	88.85091	CMIP;downstream;
11	rs_11_4369781	4369781	A	C	PC2	79.72713	CMIP;downstream;
11	rs_11_4379012	4379012	C	T	PC2	78.78964	Intron;CMIP;
12	rs_12_4807075	4807075	A	G	PC1	87.09052	Intergenic;
12	rs_12_5683212	5683212	A	G	PC1	87.09052	Intergenic;
12	rs_12_6083716	6083716	T	C	PC1	87.09052	Intergenic;
12	rs_12_6085496	6085496	C	G	PC1	87.09052	Intergenic;
12	rs_12_6085937	6085937	C	G	PC1	87.09052	Intergenic;
12	rs_12_6087323	6087323	G	A	PC1	87.09052	Intergenic;
12	rs_12_6415731	6415731	A	G	PC1	87.09052	KLHL4;upstream;
12	rs_12_6578992	6578992	T	C	PC1	87.09052	Intergenic;
12	rs_12_6606515	6606515	G	A	PC1	83.7223	Intron;MAP7;
12	rs_12_6683827	6683827	C	A	PC1	87.09052	Intron;MAP7;
12	rs_12_6783222	6783222	A	G	PC1	87.09052	Intron;MAP7;
12	rs_12_6783641	6783641	C	T	PC1	87.09052	Intron;MAP7;
12	rs_12_7404545	7404545	G	A	PC1	87.09052	Intergenic;
12	rs_12_9035911	9035911	A	G	PC1	87.09052	Intron;GABRB4;
12	rs_12_9057321	9057321	G	A	PC1	87.09052	GABRB4;upstream;
12	rs_12_9160571	9160571	G	A	PC1	87.09052	GABRG4;upstream;
12	rs_12_10696516	10696516	C	T	PC2	81.05426	Intron;SLC16A2;
12	rs_12_11440287	11440287	A	G	PC1	87.09052	Intergenic;
12	rs_12_14066035	14066035	A	G	PC2	101.3649	Intron;THOC2;
13	rs_13_9386	9386	G	A	PC1	77.99387	Intron;EGR1;
13	rs_13_14353	14353	A	G	PC1	77.99387	Intron;EGR1;
13	rs_13_14487	14487	T	C	PC1	77.99387	Intron;EGR1;
13	rs_13_14949	14949	T	C	PC1	77.99387	Intron;EGR1;
13	rs_13_15291	15291	G	A	PC1	77.99387	Intron;EGR1;
13	rs_13_15380	15380	G	A	PC1	77.99387	Intron;EGR1;
13	rs_13_17867	17867	A	G	PC1	77.99387	Intron;EGR1;
13	rs_13_22330	22330	C	T	PC1	77.99387	EGR1;upstream;
13	rs_13_25589	25589	C	A	PC1	77.99387	EGR1;upstream;
13	rs_13_29538	29538	G	T	PC1	77.99387	Intergenic;
13	rs_13_62853	62853	T	A	PC1	77.99387	REEP2;upstream;KDM3B;downstream;
13	rs_13_63473	63473	T	C	PC1	77.99387	REEP2;upstream;KDM3B;downstream;
13	rs_13_68156	68156	A	G	PC1	77.99387	Intron;KDM3B;
13	rs_13_71359	71359	C	A	PC1	77.99387	Intron;KDM3B;
13	rs_13_72047	72047	T	C	PC1	77.99387	Intron;KDM3B;
13	rs_13_72697	72697	C	T	PC1	77.99387	Intron;KDM3B;
13	rs_13_75350	75350	A	G	PC1	77.99387	Intron;KDM3B;
13	rs_13_78296	78296	G	A	PC1	77.99387	Intron;KDM3B;
13	rs_13_84295	84295	C	A	PC1	77.99387	Intron;KDM3B;
13	rs_13_84938	84938	C	A	PC1	77.99387	Intron;KDM3B;
13	rs_13_90112	90112	G	T	PC1	77.99387	Intron;KDM3B;
13	rs_13_90423	90423	A	G	PC1	77.99387	Intron;KDM3B;
13	rs_13_90489	90489	C	T	PC1	77.99387	Intron;KDM3B;
13	rs_13_98659	98659	G	A	PC1	77.99387	Intron;KDM3B;
13	rs_13_108206	108206	C	T	PC1	77.99387	Intron;KDM3B;
13	rs_13_108333	108333	G	A	PC1	77.99387	Intron;KDM3B;
13	rs_13_108465	108465	C	T	PC1	77.99387	Intron;KDM3B;
13	rs_13_112666	112666	A	T	PC1	77.99387	Intron;KDM3B;
13	rs_13_113707	113707	A	G	PC1	77.99387	Intron;KDM3B;
13	rs_13_114670	114670	A	G	PC1	77.99387	Intron;KDM3B;
13	rs_13_114923	114923	C	T	PC1	77.99387	Intron;KDM3B;
13	rs_13_114972	114972	C	T	PC1	77.99387	Intron;KDM3B;
13	rs_13_117980	117980	A	G	PC1	77.99387	KDM3B;upstream;CTBPL;downstream;
13	rs_13_118524	118524	G	C	PC1	77.99387	KDM3B;upstream;CTBPL;downstream;
13	rs_13_119564	119564	C	T	PC1	77.99387	KDM3B;upstream;CTBPL;downstream;
13	rs_13_122830	122830	A	C	PC1	77.99387	KDM3B;upstream;GFRA3;upstream;
13	rs_13_132493	132493	G	C	PC1	77.99387	Intron;GFRA3;
13	rs_13_134328	134328	G	A	PC1	77.99387	Intron;GFRA3;
13	rs_13_136331	136331	A	G	PC1	77.99387	Intron;GFRA3;
13	rs_13_136678	136678	T	A	PC1	77.99387	Intron;GFRA3;
13	rs_13_149372	149372	G	T	PC1	77.99387	Intron;GFRA3;
13	rs_13_151936	151936	G	A	PC1	77.99387	Intron;GFRA3;
13	rs_13_156603	156603	G	C	PC1	77.99387	GFRA3;downstream;
13	rs_13_195650	195650	T	C	PC1	77.99387	CDC23;downstream;
13	rs_13_254785	254785	A	G	PC1	77.99387	DPYSL2;upstream;
13	rs_13_674257	674257	A	G	PC1	77.99387	Intergenic;
13	rs_13_1577920	1577920	G	A	PC2	92.59002	Intron;P4HA2;
13	rs_13_1581420	1581420	A	G	PC2	92.59002	Intron;P4HA2;
13	rs_13_1583641	1583641	C	T	PC2	92.59002	Intron;P4HA2;
13	rs_13_1586130	1586130	A	G	PC2	78.15034	P4HA2;downstream;
13	rs_13_1589407	1589407	G	A	PC2	83.48941	P4HA2;downstream;Predicted;downstream;
13	rs_13_1592223	1592223	T	G	PC2	80.35912	Intron;Predicted;
13	rs_13_1594774	1594774	C	T	PC2	80.27411	Intron;Predicted;
13	rs_13_1595603	1595603	C	T	PC2	84.8197	Intron;Predicted;
13	rs_13_1602013	1602013	G	T	PC2	85.33591	Predicted;upstream;
13	rs_13_1609377	1609377	A	T	PC2	92.94772	Intergenic;
13	rs_13_1609604	1609604	T	C	PC2	92.94772	Intergenic;
13	rs_13_1662103	1662103	C	T	PC2	92.59002	ACSL6;downstream;
13	rs_13_1673633	1673633	T	C	PC2	92.59002	MEIKIN;downstream;FNIP1;upstream;
13	rs_13_1677347	1677347	G	A	PC2	92.94772	Intron;FNIP1;

13	rs_13_1960103	1960103	C	T	PC2	77.93653	Intron;HMCN1;
13	rs_13_13267521	13267521	G	A	PC1	77.99387	Intergenic;
13	rs_13_17112964	17112964	C	G	PC1	77.99387	UBE2D2;upstream;
13	rs_13_17820288	17820288	G	A	Others	89.02837	Intergenic;
14	rs_14_15344605	15344605	C	T	PC2	100.7353	Intron;LYRM1;
14	rs_14_15356480	15356480	A	G	PC2	100.7353	Intron;DNAH3;
14	rs_14_15357842	15357842	G	A	PC2	100.7353	Intron;DNAH3;
15	rs_15_5674509	5674509	T	C	PC2	78.20741	Predicted;upstream;
15	rs_15_5678090	5678090	A	G	PC2	79.67139	Predicted;upstream;
15	rs_15_5806339	5806339	T	G	PC2	79.67139	Intron;COL9A3;
15	rs_15_5811560	5811560	G	A	PC2	79.67139	Intron;COL9A3;
15	rs_15_5813663	5813663	C	T	PC2	79.67139	Intron;COL9A3;
15	rs_15_5820182	5820182	A	G	PC2	79.67139	Intron;COL9A3;
15	rs_15_5820876	5820876	G	A	PC2	79.67139	Intron;COL9A3;
15	rs_15_5821143	5821143	T	C	PC2	79.67139	Intron;COL9A3;
15	rs_15_5830298	5830298	A	G	PC2	79.67139	COL9A3;upstream;MRGBP;downstream;
15	rs_15_9041715	9041715	A	G	PC2	78.53665	Intron;KCNS1;
15	rs_15_9051136	9051136	G	A	PC2	78.53665	Exon;KCNS1;KCNS1;-;2;CTC;Synonymous;Leu;Leu;
15	rs_15_9054401	9054401	T	A	PC2	78.91928	Intron;RBPJL;
15	rs_15_9059645	9059645	C	T	PC2	89.97707	Intron;RBPJL;
15	rs_15_9086483	9086483	T	A	PC2	78.17966	Intron;SDC4;
15	rs_15_9095363	9095363	A	G	PC2	78.17966	SDC4;upstream;
15	rs_15_9113846	9113846	C	T	PC2	77.61004	Predicted;upstream;
15	rs_15_9123904	9123904	G	A	PC2	77.61004	SRC;downstream;
15	rs_15_9130433	9130433	G	A	PC2	77.61004	SRC;downstream;
15	rs_15_9137650	9137650	G	A	PC2	87.19123	Intron;SRC;
15	rs_15_12427570	12427570	A	G	PC2	76.6088	Intron;EIF2S2,;
16	rs_16_112009	112009	C	T	PC2	116.5949	Intergenic;
16	rs_16_112657	112657	C	T	PC2	116.5949	Intergenic;
16	rs_16_115465	115465	A	C	PC2	116.5949	Intergenic;
16	rs_16_117504	117504	G	A	PC2	116.5949	Intergenic;
16	rs_16_118492	118492	G	A	PC2	79.42954	Intergenic;
16	rs_16_118756	118756	A	T	PC2	116.5949	Intergenic;
16	rs_16_121559	121559	C	A	PC2	95.3781	Intergenic;
16	rs_16_121736	121736	C	T	PC2	95.3781	Intergenic;
16	rs_16_135560	135560	G	C	PC2	95.33872	Intergenic;
16	rs_16_139967	139967	A	T	PC2	112.1699	Intergenic;
16	rs_16_140148	140148	C	T	PC2	132.3564	Intergenic;
16	rs_16_143351	143351	T	C	PC2	132.4035	Intergenic;
16	rs_16_145374	145374	G	A	PC2	167.3956	Intergenic;
16	rs_16_145782	145782	A	G	PC2	167.3956	Intergenic;
16	rs_16_147612	147612	T	C	PC2	84.69526	Intergenic;
16	rs_16_151752	151752	T	C	PC2	103.3782	Intergenic;
16	rs_16_151773	151773	G	A	PC2	85.89711	Intergenic;
16	rs_16_151794	151794	A	G	PC2	167.3956	Intergenic;
16	rs_16_153627	153627	C	A	PC2	132.3564	Intergenic;
16	rs_16_162461	162461	G	A	PC2	132.4035	Intergenic;
16	rs_16_162505	162505	T	C	PC2	132.4035	Intergenic;
16	rs_16_203120	203120	C	T	PC2	97.30505	Intergenic;
16	rs_16_208436	208436	A	G	PC2	98.7067	Intergenic;
16	rs_16_214404	214404	C	A	PC2	174.4731	Intergenic;
16	rs_16_215459	215459	C	T	PC2	98.56957	Intergenic;
16	rs_16_215991	215991	C	T	PC2	116.2342	Intergenic;
16	rs_16_216418	216418	C	G	PC2	138.4894	Intergenic;
16	rs_16_218568	218568	G	T	PC2	119.8686	Intergenic;
16	rs_16_225191	225191	C	G	PC2	84.5061	Intergenic;
16	rs_16_227902	227902	G	T	PC2	119.8686	Intergenic;
16	rs_16_228195	228195	T	C	PC2	119.8686	Intergenic;
16	rs_16_228447	228447	G	A	PC2	119.8686	Intergenic;
16	rs_16_230437	230437	A	C	PC2	119.8686	Intergenic;
16	rs_16_234888	234888	T	C	PC2	119.8686	Intergenic;
16	rs_16_235537	235537	A	T	PC2	84.18573	Intergenic;
16	rs_16_265553	265553	G	A	PC2	121.1636	Intergenic;
16	rs_16_265951	265951	C	T	PC2	102.746	Intergenic;
16	rs_16_269301	269301	C	T	PC2	102.746	Intergenic;
16	rs_16_269434	269434	G	T	PC2	89.10429	Intergenic;
16	rs_16_269500	269500	C	T	PC2	89.10429	Intergenic;
16	rs_16_269693	269693	T	C	PC2	79.47632	Intergenic;
16	rs_16_275922	275922	T	C	PC2	82.45494	Intergenic;
16	rs_16_277663	277663	C	G	PC2	96.31035	Intergenic;
16	rs_16_277979	277979	A	G	PC2	96.31035	Intergenic;
16	rs_16_280799	280799	C	T	PC2	96.31035	Intergenic;
16	rs_16_284154	284154	G	A	PC2	81.77378	Intergenic;
16	rs_16_286777	286777	G	A	PC2	81.77378	Intergenic;
16	rs_16_294954	294954	A	G	PC2	96.31035	Intergenic;
16	rs_16_301031	301031	C	G	PC2	96.31035	Intergenic;
16	rs_16_303143	303143	A	G	PC2	77.07858	Intergenic;
16	rs_16_308263	308263	G	A	PC2	77.07858	Intergenic;
16	rs_16_415617	415617	G	A	PC2	89.92118	Intergenic;
16	rs_16_418595	418595	T	C	PC2	89.92118	Intron;Predicted;
16	rs_16_419033	419033	A	T	PC2	89.92118	Intron;Predicted;
16	rs_16_419426	419426	T	C	PC2	89.92118	Intron;Predicted;
16	rs_16_419881	419881	A	G	PC2	89.92118	Intron;Predicted;
16	rs_16_419897	419897	G	T	PC2	89.92118	Intron;Predicted;
16	rs_16_421236	421236	G	A	PC2	89.92118	Intergenic;
16	rs_16_429750	429750	T	C	PC2	89.92118	Predicted;downstream;
16	rs_16_437544	437544	T	C	PC2	89.92118	Intergenic;
16	rs_16_701574	701574	G	A	PC2	88.54605	OSBP2;downstream;

16	rs_16_1249229C	12492290	C	T	PC2	108.3206	Intron;PPIL2;
16	rs_16_12494163	12494163	A	G	PC2	108.3206	Intron;PPIL2;
16	rs_16_1250116C	12501160	C	T	PC2	108.3206	PPIL2;downstream;YPEL1;downstream;
16	rs_16_12534242	12534242	A	T	PC2	108.3206	Intergenic;
16	rs_16_12535931	12535931	C	T	PC2	108.3206	Intergenic;
16	rs_16_12557457	12557457	A	G	PC2	79.23736	Intron;MAPK1;
16	rs_16_12559494	12559494	C	T	PC2	79.23736	Intron;MAPK1;
16	rs_16_12573091	12573091	G	A	PC2	81.43773	MAPK1;upstream;
16	rs_16_12574052	12574052	G	A	PC2	79.23736	MAPK1;upstream;
16	rs_16_12575629	12575629	C	G	PC2	79.23736	MAPK1;upstream;
16	rs_16_12576093	12576093	C	T	PC2	79.23736	MAPK1;upstream;
16	rs_16_12577748	12577748	A	C	PC2	79.23736	MAPK1;upstream;
16	rs_16_12579244	12579244	A	G	PC2	79.23736	MAPK1;upstream;
16	rs_16_12586685	12586685	G	T	PC2	79.23736	Intergenic;
16	rs_16_12589833	12589833	C	T	PC2	79.23736	PPM1F;downstream;
16	rs_16_12592659	12592659	C	T	PC2	79.23736	PPM1F;downstream;
16	rs_16_12595127	12595127	C	G	PC2	79.23736	PPM1F;downstream;
16	rs_16_12597614	12597614	C	T	PC2	79.23736	Intron;PPM1F;
16	rs_16_12605654	12605654	T	C	PC2	79.23736	Intron;PPM1F;
16	rs_16_12609568	12609568	G	A	PC2	79.23736	Intron;PPM1F;
16	rs_16_12630392	12630392	A	G	PC2	79.23736	TOP3B;upstream;
16	rs_16_12634003	12634003	T	C	PC2	79.23736	TOP3B;upstream;
16	rs_16_12643605	12643605	C	A	PC2	81.43773	Intergenic;
16	rs_16_12690488	12690488	T	C	PC2	80.53959	Intergenic;
16	rs_16_12738188	12738188	C	A	PC2	83.11424	UBE2L3;upstream;
16	rs_16_12739343	12739343	C	G	PC2	80.53959	UBE2L3;upstream;
16	rs_16_12745543	12745543	C	T	PC2	80.53959	Intergenic;
16	rs_16_1276180C	12761800	A	G	PC2	80.09791	Intergenic;
16	rs_16_12762768	12762768	T	C	PC2	80.53959	Intergenic;
16	rs_16_12771827	12771827	G	A	PC2	82.28969	Intergenic;
16	rs_16_12777491	12777491	A	C	PC2	78.49291	Intergenic;
16	rs_16_12780756	12780756	C	T	PC2	78.49291	Intergenic;
16	rs_16_12784684	12784684	A	T	PC2	83.41778	Intergenic;
16	rs_16_12785649	12785649	A	G	PC2	78.49291	Intergenic;
16	rs_16_12785675	12785675	T	C	PC2	83.11424	Intergenic;
16	rs_16_12787738	12787738	C	A	PC2	78.49291	Intergenic;
16	rs_16_12794111	12794111	A	G	PC2	78.49291	Intergenic;
16	rs_16_12901325	12901325	A	G	PC2	83.11424	Intergenic;
16	rs_16_12933846	12933846	A	G	PC2	83.11424	PI4KA;downstream;
16	rs_16_12979782	12979782	T	A	PC2	90.6857	Intron;PI4KA;
16	rs_16_12979788	12979788	T	C	PC2	90.6857	Intron;PI4KA;
16	rs_16_12994233	12994233	T	C	PC2	80.53959	Exon;PI4KA;PI4KA;-;0;ATT;Nonsynonymous;Ile;Val;
16	rs_16_12994632	12994632	T	C	PC2	80.53959	Intron;PI4KA;
17	rs_17_4272976	4272976	G	T	PC2	76.59766	Intergenic;
17	rs_17_4274576	4274576	G	T	PC2	76.59766	Intergenic;
17	rs_17_5631514	5631514	C	T	PC2	80.39066	Intron;RNF213;
17	rs_17_9347074	9347074	C	T	PC1	77.99387	Intron;MYH10;
17	rs_17_9882247	9882247	C	T	PC1	77.99387	XRCC6;downstream;
17	rs_17_9900295	9900295	C	G	PC1	77.99387	Intergenic;
17	rs_17_9901299	9901299	C	G	PC1	77.99387	Intergenic;
17	rs_17_10051665	10051665	C	T	PC1	77.99387	Intron;DNAH9;
17	rs_17_10084335	10084335	C	T	PC1	77.99387	Intron;DNAH9;
17	rs_17_10084637	10084637	G	A	PC1	77.99387	Intron;DNAH9;
17	rs_17_1008656C	10086560	T	G	PC1	77.99387	Intron;DNAH9;
17	rs_17_10102917	10102917	T	C	PC1	77.99387	Intron;ELAC2;
17	rs_17_10108591	10108591	A	G	PC1	77.99387	Intron;ELAC2;
17	rs_17_10110065	10110065	T	C	PC1	77.99387	Exon;ELAC2;ELAC2;-;2;TCA;Synonymous;Ser;Ser;
17	rs_17_10116861	10116861	G	A	PC1	77.99387	ELAC2;upstream;
17	rs_17_10117175	10117175	A	G	PC1	77.99387	ELAC2;upstream;
17	rs_17_10117445	10117445	A	G	PC1	77.99387	ELAC2;upstream;
17	rs_17_1012067C	10120670	T	C	PC1	77.99387	ELAC2;upstream;
17	rs_17_10123939	10123939	T	A	PC1	77.99387	Intergenic;
17	rs_17_1012469C	10124690	T	C	PC1	77.99387	Intergenic;
17	rs_17_10125234	10125234	G	A	PC1	77.99387	Intergenic;
17	rs_17_10127579	10127579	G	A	PC1	77.99387	Intergenic;
17	rs_17_10129903	10129903	T	A	PC1	77.99387	Intergenic;
17	rs_17_10146346	10146346	T	G	PC1	77.99387	Intergenic;
17	rs_17_10224431	10224431	T	C	PC1	77.99387	Intergenic;
17	rs_17_10234126	10234126	A	G	PC1	77.99387	Intergenic;
17	rs_17_10234242	10234242	T	C	PC1	77.99387	Intergenic;
17	rs_17_10238984	10238984	A	G	PC1	77.99387	Intron;MYOCD;
17	rs_17_10241426	10241426	A	G	PC1	77.99387	Intergenic;
17	rs_17_10245905	10245905	A	G	PC1	77.99387	Intergenic;
17	rs_17_10246564	10246564	C	G	PC1	77.99387	Intergenic;
17	rs_17_10247329	10247329	T	A	PC1	77.99387	Intergenic;
17	rs_17_10249467	10249467	T	C	PC1	77.99387	MYOCD;downstream;
17	rs_17_10283282	10283282	A	C	PC1	77.99387	Intergenic;
17	rs_17_10284898	10284898	G	A	PC1	77.99387	Intergenic;
17	rs_17_10285462	10285462	A	G	PC1	77.99387	Intergenic;
17	rs_17_1028732C	10287320	T	C	PC1	77.99387	Intergenic;
17	rs_17_10289702	10289702	A	G	PC1	77.99387	Intergenic;
17	rs_17_10294466	10294466	A	G	PC1	77.99387	Intergenic;
17	rs_17_10301302	10301302	C	T	PC1	77.99387	MYOCD;upstream;
17	rs_17_10307006	10307006	A	G	PC1	77.99387	Intron;MYOCD;
17	rs_17_10307057	10307057	A	G	PC1	77.99387	Intron;MYOCD;
17	rs_17_10308678	10308678	T	C	PC1	77.99387	Intron;MYOCD;
17	rs_17_10312556	10312556	T	C	PC1	77.99387	Intron;MYOCD;
17	rs_17_10315293	10315293	A	G	PC1	77.99387	Intron;MYOCD;

17	rs_17_10323957	10323957	T	C	PC1	77.99387	Intron;MYOCD;
17	rs_17_10325465	10325465	T	C	PC1	77.99387	Intron;MYOCD;
17	rs_17_10330703	10330703	T	G	PC1	77.99387	Intron;MYOCD;
17	rs_17_10331085	10331085	G	C	PC1	77.99387	Intron;MYOCD;
17	rs_17_10333683	10333683	C	T	PC1	77.99387	Exon;MYOCD;MYOCD;+;2;CCC;Synonymous;Pro;Pro;
17	rs_17_10339383	10339383	A	G	PC1	77.99387	Intron;MYOCD;
17	rs_17_10413302	10413302	G	A	PC1	77.99387	Intron;MAP2K4;
17	rs_17_10413331	10413331	T	A	PC1	77.99387	Intron;MAP2K4;
17	rs_17_10415921	10415921	T	A	PC1	77.99387	Intron;MAP2K4;
17	rs_17_10426921	10426921	A	T	PC1	77.99387	Intron;MAP2K4;
17	rs_17_10429169	10429169	G	T	PC1	77.99387	Intron;MAP2K4;
17	rs_17_10443734	10443734	C	G	PC1	77.99387	MAP2K4;upstream;
17	rs_17_10446789	10446789	C	G	PC1	77.99387	MAP2K4;upstream;ADPRM;downstream;
17	rs_17_10461866	10461866	T	C	PC1	77.99387	Intergenic;
17	rs_17_10463391	10463391	A	G	PC1	77.99387	ADPRM;upstream;
17	rs_17_10469823	10469823	A	G	PC1	77.99387	Intergenic;
17	rs_17_10482747	10482747	G	A	PC1	77.99387	MYH;upstream;
17	rs_17_10509123	10509123	A	T	PC1	77.99387	Intron;MYH;
17	rs_17_10512753	10512753	C	G	PC1	77.99387	Intron;MYH;
17	rs_17_10514360	10514360	C	T	PC1	77.99387	Intron;MYH;
17	rs_17_10552381	10552381	C	T	PC1	77.99387	Intron;MYH;
17	rs_17_10553766	10553766	A	G	PC1	77.99387	Intron;MYH;
17	rs_17_10574048	10574048	T	C	PC1	77.99387	Intron;MYH;
17	rs_17_10583046	10583046	A	T	PC1	77.99387	MYH;downstream;
17	rs_17_10590785	10590785	T	G	PC1	77.99387	MYH;downstream;
17	rs_17_10594738	10594738	A	G	PC1	77.99387	Intergenic;
17	rs_17_10599457	10599457	T	G	PC1	77.99387	Intergenic;
17	rs_17_10599700	10599700	C	G	PC1	77.99387	Intergenic;
17	rs_17_10606775	10606775	C	G	PC1	77.99387	Intergenic;
17	rs_17_10611065	10611065	A	G	PC1	77.99387	Intergenic;
17	rs_17_10614716	10614716	A	G	PC1	77.99387	Intergenic;
17	rs_17_10634124	10634124	C	T	PC1	77.99387	Intergenic;
17	rs_17_10636562	10636562	T	C	PC1	77.99387	MYH;upstream;
17	rs_17_10640734	10640734	G	A	PC1	77.99387	Intergenic;
17	rs_17_10642280	10642280	C	G	PC1	77.99387	Intergenic;
17	rs_17_10645661	10645661	T	A	PC1	77.99387	Intron;MYH;
17	rs_17_10647399	10647399	G	A	PC1	77.99387	Intergenic;
17	rs_17_10649891	10649891	C	T	PC1	77.99387	Intergenic;
17	rs_17_10651674	10651674	A	G	PC1	77.99387	Intergenic;
17	rs_17_10663926	10663926	G	A	PC1	77.99387	Intergenic;
17	rs_17_10676966	10676966	G	A	PC1	77.99387	Intergenic;
17	rs_17_10679825	10679825	G	A	PC1	77.99387	Intergenic;
17	rs_17_10683526	10683526	C	A	PC1	77.99387	Intergenic;
17	rs_17_10683867	10683867	T	A	PC1	77.99387	Intergenic;
17	rs_17_10689014	10689014	C	T	PC1	77.99387	Intergenic;
17	rs_17_10697036	10697036	T	C	PC1	77.99387	Intergenic;
17	rs_17_10702042	10702042	T	G	PC1	77.99387	Intergenic;
17	rs_17_10702043	10702043	G	A	PC1	77.99387	Intergenic;
17	rs_17_10714836	10714836	C	A	PC1	77.99387	Intergenic;
17	rs_17_10714959	10714959	C	G	PC1	77.99387	Intergenic;
17	rs_17_10720650	10720650	C	T	PC1	77.99387	Intergenic;
17	rs_17_10729289	10729289	A	G	PC1	77.99387	Intergenic;
17	rs_17_10732740	10732740	C	T	PC1	77.99387	Intergenic;
17	rs_17_10751007	10751007	G	A	PC1	77.99387	Intron;MYH1D;
17	rs_17_10755350	10755350	A	G	PC1	77.99387	Intron;MYH1D;
17	rs_17_10756034	10756034	G	A	PC1	77.99387	Intron;MYH1D;
17	rs_17_10756042	10756042	C	A	PC1	77.99387	Intron;MYH1D;
17	rs_17_10765098	10765098	T	C	PC1	77.99387	Intron;MYH1D;
17	rs_17_10769163	10769163	T	C	PC1	77.99387	Intron;MYH1D;
17	rs_17_10771017	10771017	T	C	PC1	77.99387	Intron;MYH1D;
17	rs_17_10775902	10775902	A	G	PC1	77.99387	MYH1D;downstream;
17	rs_17_10784266	10784266	T	C	PC1	77.99387	Intergenic;
17	rs_17_10804063	10804063	G	A	PC1	77.99387	Intergenic;
17	rs_17_10814620	10814620	A	G	PC1	77.99387	Intergenic;
17	rs_17_10820063	10820063	A	G	PC1	77.99387	Intergenic;
17	rs_17_10821827	10821827	A	G	PC1	77.99387	Intergenic;
17	rs_17_10824721	10824721	C	A	PC1	77.99387	Intergenic;
17	rs_17_10827497	10827497	T	C	PC1	77.99387	Intergenic;
17	rs_17_10840105	10840105	T	G	PC1	77.99387	Intergenic;
17	rs_17_10857335	10857335	T	C	PC1	77.99387	Intron;GAS7;
17	rs_17_10875038	10875038	T	C	PC1	77.99387	Intron;GAS7;
17	rs_17_10883262	10883262	A	G	PC1	77.99387	Intron;GAS7;
17	rs_17_10896295	10896295	T	C	PC1	77.99387	Intron;GAS7;
17	rs_17_10902388	10902388	T	C	PC1	77.99387	Intron;GAS7;
17	rs_17_10902805	10902805	G	C	PC1	77.99387	Intron;GAS7;
17	rs_17_10903244	10903244	G	A	PC1	77.99387	Intron;GAS7;
17	rs_17_10905220	10905220	G	A	PC1	77.99387	Intron;GAS7;
17	rs_17_10909011	10909011	A	G	PC1	77.99387	Intron;GAS7;
17	rs_17_10941634	10941634	A	G	PC1	77.99387	Intron;GAS7;
17	rs_17_10944942	10944942	G	A	PC1	77.99387	Intron;GAS7;
17	rs_17_10950255	10950255	G	A	PC1	77.99387	Intron;GAS7;
17	rs_17_10951116	10951116	G	T	PC1	77.99387	Intron;GAS7;
17	rs_17_10953540	10953540	G	A	PC1	77.99387	Intron;GAS7;
17	rs_17_10958802	10958802	G	A	PC1	77.99387	GAS7;downstream;
17	rs_17_10961537	10961537	A	C	PC1	77.99387	GAS7;downstream;
17	rs_17_10964958	10964958	G	A	PC1	77.99387	GAS7;downstream;GLP2R;downstream;
17	rs_17_10974369	10974369	A	G	PC1	77.99387	GLP2R;downstream;
17	rs_17_10977983	10977983	T	C	PC1	77.99387	Intron;GLP2R;

17	rs_17_10987417	10987417	T	C	PC1	77.99387	Intron;GLP2R;
17	rs_17_10989854	10989854	A	T	PC1	77.99387	Intron;GLP2R;
17	rs_17_10998769	10998769	T	C	PC1	77.99387	Intron;GLP2R;
17	rs_17_11001108	11001108	A	T	PC1	77.99387	Intron;GLP2R;
17	rs_17_11004063	11004063	G	C	PC1	77.99387	Intron;GLP2R;
17	rs_17_11007221	11007221	A	G	PC1	77.99387	Intron;GLP2R;
17	rs_17_11015983	11015983	G	A	PC1	77.99387	Intron;GSG1L;
17	rs_17_11023051	11023051	A	G	PC1	77.99387	Intron;GSG1L;
17	rs_17_11036383	11036383	T	C	PC1	77.99387	Intron;DHRS7C;
17	rs_17_11056217	11056217	G	A	PC1	77.99387	Intergenic;
17	rs_17_11056793	11056793	A	G	PC1	77.99387	Intergenic;
17	rs_17_11059839	11059839	A	G	PC1	77.99387	Intergenic;
17	rs_17_11069577	11069577	A	T	PC1	77.99387	Intergenic;
17	rs_17_11075791	11075791	G	A	PC1	77.99387	Intergenic;
17	rs_17_11079998	11079998	T	C	PC1	77.99387	Intergenic;
17	rs_17_11083828	11083828	A	T	PC1	77.99387	Intergenic;
17	rs_17_11088246	11088246	A	G	PC1	77.99387	Predicted;upstream;
18	rs_18_227719	227719	G	A	PC1	87.09052	Intron;STX1A;
18	rs_18_2113602	2113602	C	T	PC2	86.04118	Intron;AUTS2;
18	rs_18_3354619	3354619	A	G	PC2	117.5414	Intron;ANKFY1;
18	rs_18_6482979	6482979	C	T	PC2	77.09998	Intron;SLC6A4;
18	rs_18_6624913	6624913	G	A	PC2	79.58128	Intron;TIMM22;
18	rs_18_6625273	6625273	C	T	PC2	79.58128	Intron;TIMM22;
18	rs_18_6627092	6627092	G	C	PC2	79.58128	ABR;downstream;
18	rs_18_6627647	6627647	G	A	PC2	89.1122	ABR;downstream;
18	rs_18_6640265	6640265	G	T	PC2	91.38074	SPECC1;upstream;
18	rs_18_6648202	6648202	T	C	PC2	79.582	Intron;SPECC1;
18	rs_18_7449386	7449386	A	G	PC2	108.9125	Intron;PPM1E;
18	rs_18_7450738	7450738	A	G	PC2	96.17589	Intron;PPM1E;
18	rs_18_7453214	7453214	G	A	PC2	97.07469	Intron;PPM1E;
19	rs_19_10024386	10024386	A	G	PC1	77.99387	Intron;NPDC1;
19	rs_19_10048413	10048413	C	G	PC1	77.99387	Intron;POU5F3;
20	rs_20_1542531	1542531	A	T	PC1	77.99387	Intron;AADACL4L1;
20	rs_20_1545929	1545929	T	G	PC1	77.99387	Intron;AADACL4L1;
20	rs_20_1545941	1545941	G	A	PC1	77.99387	Intron;AADACL4L1;
20	rs_20_1545965	1545965	A	C	PC1	77.99387	Intron;AADACL4L1;
20	rs_20_1546120	1546120	G	A	PC1	77.99387	Intron;AADACL4L1;
20	rs_20_1547859	1547859	T	G	PC1	77.99387	Intron;AADACL4L1;
20	rs_20_2734073	2734073	C	T	PC2	84.27815	Intron;EXOSC10;
20	rs_20_2736655	2736655	C	T	PC2	84.27815	Intron;EXOSC10;
20	rs_20_2736657	2736657	G	T	PC2	84.27815	Intron;EXOSC10;
20	rs_20_2744278	2744278	G	A	PC2	88.63878	Exon;EXOSC10;EXOSC10;+;2;GTG;Synonymous;Val;Val;
20	rs_20_2749038	2749038	A	T	PC2	93.0618	Intron;SRM;
20	rs_20_2749341	2749341	T	A	PC2	93.0618	Intron;SRM;
20	rs_20_2749826	2749826	G	A	PC2	84.73258	Intron;SRM;
20	rs_20_2751561	2751561	G	T	PC2	82.42201	Intron;SRM;
20	rs_20_2800881	2800881	C	G	PC2	77.00416	Intergenic;
20	rs_20_2800903	2800903	A	C	PC2	77.00416	Intergenic;
20	rs_20_2809415	2809415	A	G	PC2	160.7836	Intergenic;
20	rs_20_2812005	2812005	A	G	PC2	173.2482	Intergenic;
20	rs_20_2813162	2813162	A	G	PC2	140.0391	Intergenic;
20	rs_20_2814005	2814005	C	T	PC2	108.8165	Intergenic;
20	rs_20_2815950	2815950	G	A	PC2	102.7197	Intergenic;
20	rs_20_2816028	2816028	A	G	PC2	79.58458	Intergenic;
20	rs_20_2816229	2816229	C	T	PC2	78.74374	Intergenic;
20	rs_20_4394645	4394645	C	T	PC1	77.99387	Intron;FAM132A;
20	rs_20_6948827	6948827	G	A	PC1	77.99387	Intron;IGSF21;
21	rs_21_12688	12688	C	T	PC1	83.7223	C2CD2L;downstream;
21	rs_21_21153	21153	G	A	PC1	83.7223	Exon;C2CD2L;C2CD2L;-;2;GGC;Synonymous;Gly;Gly;
21	rs_21_25852	25852	C	T	PC1	83.7223	Intron;C2CD2L;
21	rs_21_26042	26042	A	G	PC1	83.7223	Intron;C2CD2L;
21	rs_21_27232	27232	C	T	PC1	83.7223	Intron;C2CD2L;
21	rs_21_27699	27699	C	T	PC1	83.7223	Intron;C2CD2L;
21	rs_21_63824	63824	A	G	PC1	83.7223	Intron;VPS11;
21	rs_21_68023	68023	A	G	PC1	83.7223	VPS11;upstream;SIK2;upstream;
21	rs_21_70673	70673	C	G	PC1	83.7223	Intron;SIK2;
21	rs_21_71779	71779	C	G	PC1	83.7223	Intron;SIK2;
21	rs_21_73623	73623	T	A	PC1	83.7223	Intron;SIK2;
21	rs_21_76281	76281	G	A	PC1	83.7223	Intron;SIK2;
21	rs_21_80043	80043	T	C	PC1	83.7223	Intron;SIK2;
21	rs_21_82574	82574	A	G	PC1	83.7223	Intron;SIK2;
21	rs_21_117258	117258	A	C	PC1	83.7223	SIK2;downstream;
22	rs_22_2699202	2699202	G	A	PC2	85.15276	Intron;EPB41;
22	rs_22_2706597	2706597	C	A	PC2	95.48667	EPB41;downstream;
22	rs_22_2709668	2709668	G	A	PC2	121.5103	Exon;TMEM200B;TMEM200B;-;2;GTC;Synonymous;Val;Val;
22	rs_22_2709698	2709698	C	T	PC2	98.45372	Exon;TMEM200B;TMEM200B;-;2;TCG;Synonymous;Ser;Ser;
22	rs_22_2733771	2733771	T	C	PC2	121.5934	Intron;L3MBTL3;
22	rs_22_2736386	2736386	T	C	PC2	105.5434	Intron;L3MBTL3;
22	rs_22_2747013	2747013	G	A	PC2	86.06007	Intron;L3MBTL3;
22	rs_22_2764132	2764132	G	A	PC2	90.24974	Intron;L3MBTL3;
22	rs_22_3100378	3100378	C	T	PC2	79.98623	Intergenic;
22	rs_22_3130643	3130643	C	T	PC2	98.01766	Intron;Predicted;
22	rs_22_3131228	3131228	A	C	PC2	98.01766	Intron;Predicted;
22	rs_22_3133689	3133689	G	A	PC2	98.01766	Intron;Predicted;
22	rs_22_3137989	3137989	G	C	PC2	98.01766	Predicted;downstream;
22	rs_22_3141869	3141869	G	A	PC2	88.06461	Predicted;downstream;
22	rs_22_3143781	3143781	T	A	PC2	89.3701	Predicted;downstream;
22	rs_22_3151509	3151509	C	G	PC2	81.57283	Intergenic;

22	rs_22_3154004	3154004	A	G	PC2	89.3701	Intergenic;
22	rs_22_3154214	3154214	G	A	PC2	89.3701	Intergenic;
22	rs_22_3154225	3154225	C	T	PC2	89.3701	Intergenic;
22	rs_22_3155828	3155828	C	T	PC2	89.3701	Intergenic;
22	rs_22_3190697	3190697	C	T	PC2	89.29005	Intergenic;
22	rs_22_3217979	3217979	C	T	PC2	76.57825	Intergenic;
22	rs_22_3228905	3228905	C	T	PC2	82.65008	Intergenic;
22	rs_22_3243192	3243192	G	C	PC2	76.57825	TDRP;upstream;
22	rs_22_3249702	3249702	C	G	PC2	76.57825	Intergenic;
22	rs_22_3253570	3253570	C	A	PC2	76.57825	Intergenic;
22	rs_22_3257037	3257037	T	A	PC2	76.57825	PDIK1L;downstream;
22	rs_22_3307589	3307589	G	A	PC2	83.19616	Exon;PAQR7;PAQR7;+;2;TCG;Synonymous;Ser;Ser;
22	rs_22_3311284	3311284	G	T	PC2	96.34278	STMN1;downstream;RRAGC;upstream;
22	rs_22_3311710	3311710	T	G	PC2	82.25258	STMN1;downstream;RRAGC;upstream;
22	rs_22_3311711	3311711	G	T	PC2	82.25258	STMN1;downstream;RRAGC;upstream;
22	rs_22_3315500	3315500	T	C	PC2	96.34278	Intron;MYCBP;
22	rs_22_3328679	3328679	C	T	PC2	96.34278	RRAGC;downstream;
22	rs_22_3330540	3330540	C	T	PC2	96.34278	RRAGC;downstream;
22	rs_22_3331860	3331860	T	C	PC2	83.0848	RRAGC;downstream;
22	rs_22_3339534	3339534	C	T	PC2	77.84403	Intergenic;
22	rs_22_3343830	3343830	A	G	PC2	96.34278	Intergenic;
22	rs_22_3346472	3346472	T	G	PC2	96.34278	Intergenic;
22	rs_22_3352515	3352515	G	A	PC2	97.75253	Intergenic;
22	rs_22_3353324	3353324	A	C	PC2	112.8185	Intergenic;
22	rs_22_3353989	3353989	G	A	PC2	78.59905	Intergenic;
22	rs_22_3355136	3355136	T	C	PC2	96.34278	Intergenic;
22	rs_22_3355466	3355466	T	G	PC2	82.14748	Intergenic;
22	rs_22_3356070	3356070	G	A	PC2	96.34278	Intergenic;
22	rs_22_3356167	3356167	T	A	PC2	83.78402	Intergenic;
22	rs_22_3356456	3356456	T	C	PC2	83.78402	Intergenic;
22	rs_22_3356552	3356552	T	C	PC2	96.34278	Intergenic;
22	rs_22_3356716	3356716	T	C	PC2	83.13405	Intergenic;
22	rs_22_3356924	3356924	C	T	PC2	96.34278	Intergenic;
22	rs_22_3359462	3359462	A	T	PC2	83.02439	Intergenic;
22	rs_22_3361377	3361377	C	T	PC2	83.02439	Intergenic;
22	rs_22_3361525	3361525	T	C	PC2	83.02439	Intergenic;
22	rs_22_3365124	3365124	C	T	PC2	83.02439	Intergenic;
22	rs_22_3365723	3365723	G	A	PC2	83.02439	Intergenic;
22	rs_22_3366328	3366328	A	G	PC2	83.02439	Intergenic;
22	rs_22_3374226	3374226	G	A	PC2	83.0848	Intergenic;
22	rs_22_3388080	3388080	C	T	PC2	81.32418	Intergenic;
22	rs_22_3388967	3388967	A	G	PC2	100.3284	Intergenic;
22	rs_22_3389250	3389250	C	T	PC2	120.9603	Intergenic;
22	rs_22_3397232	3397232	C	T	PC2	100.3284	Intergenic;
22	rs_22_3398449	3398449	C	T	PC2	120.9603	Intergenic;
22	rs_22_3398715	3398715	G	T	PC2	100.3826	Intergenic;
22	rs_22_3401655	3401655	A	G	PC2	120.9603	Intergenic;
22	rs_22_3403793	3403793	T	C	PC2	101.9691	Intergenic;
22	rs_22_3403815	3403815	C	T	PC2	98.84642	Intergenic;
22	rs_22_3403845	3403845	C	T	PC2	84.73133	Intergenic;
22	rs_22_3404065	3404065	T	A	PC2	106.7362	Intergenic;
22	rs_22_3412424	3412424	C	T	PC2	86.61201	Intergenic;
22	rs_22_3431741	3431741	A	G	PC2	129.3415	Intergenic;
22	rs_22_3463503	3463503	T	C	PC2	85.53346	Predicted;upstream;
22	rs_22_3469079	3469079	T	C	PC2	133.162	Predicted;upstream;
24	rs_24_124149	124149	C	A	PC1	77.99387	AARSD1;downstream;AOC2;downstream;
24	rs_24_130525	130525	T	C	PC1	77.99387	Intron;AOC2;
24	rs_24_210478	210478	A	G	PC1	77.99387	Intron;PLEKHH3;
25	rs_25_1726721	1726721	C	T	PC2	95.0807	Intron;DAZAP1;
25	rs_25_2122691	2122691	A	G	PC2	77.92566	Exon;RNF126;RNF126;+;2;TTA;Synonymous;Leu;Leu;
25	rs_25_2425480	2425480	C	T	PC2	119.8414	Intron;MBD3;
25	rs_25_2760546	2760546	C	T	PC2	79.22952	SF3A2;downstream;
25	rs_25_2771171	2771171	C	T	PC2	86.74921	OAZ1;upstream;
25	rs_25_2812292	2812292	T	A	PC2	89.05186	Intergenic;
25	rs_25_2825783	2825783	G	C	PC2	89.05186	Intergenic;
25	rs_25_2825957	2825957	C	T	PC2	89.05186	Intergenic;
25	rs_25_2831133	2831133	C	G	PC2	89.05186	RRP8;upstream;
25	rs_25_3049462	3049462	C	T	PC2	81.84025	Intron;MLLT1;
25	rs_25_3049636	3049636	A	T	PC2	81.84025	Intron;MLLT1;
25	rs_25_3086006	3086006	C	A	PC2	108.9375	ACSBG2;upstream;
25	rs_25_3086046	3086046	A	G	PC2	81.54894	ACSBG2;upstream;
25	rs_25_3086047	3086047	C	A	PC2	81.54894	ACSBG2;upstream;
25	rs_25_3101887	3101887	G	C	PC2	88.96996	Intergenic;
25	rs_25_3102047	3102047	T	A	PC2	92.97804	Intergenic;
25	rs_25_3107305	3107305	A	G	PC2	120.8571	RFX2;upstream;
25	rs_25_3110058	3110058	G	A	PC2	106.3688	RFX2;upstream;
26	rs_26_759939	759939	T	C	PC2	81.95274	Intron;LRRTM1;
26	rs_26_2453297	2453297	A	G	PC1	77.99387	Intron;ANK1;
26	rs_26_2454641	2454641	T	C	PC1	77.99387	Intron;ANK1;
26	rs_26_3509361	3509361	G	T	PC2	120.1117	TMEM230;downstream;
26	rs_26_4225127	4225127	C	T	PC2	87.02108	Intron;SEMA4C;
27	rs_27_180341	180341	G	C	PC1	87.09052	SV2A;downstream;BOLA1;downstream;
27	rs_27_681256	681256	G	A	PC1	87.09052	Intergenic;

* SNP information
- Intron, followed by the gene name where it belongs to
- downstream, match the front gene name
- upstream, match the front gene name

- Exon, followed by gene name, forward/reverse strand, codon shift, 3-bases codon for reference, mutation type, Amino Acid for reference, Amino Acid for Alternative
- Predicted, one gene that is not found in public resources

Table S4. Selective signals in the Italian breed Camosciata.

scaffold	begin	end	SNPs number	PBS_p3	PiRatio_p1_p3*
1	7890001	7900000	83	1.172885054	239.6097561
1	8220001	8230000	59	1.240907363	167.8977933
1	8240001	8250000	69	1.238844531	185.5609756
1	8260001	8270000	34	1.483236691	74.73170732
1	8280001	8290000	77	1.358793763	68.53116531
1	8290001	8300000	70	1.224476029	87.34030197
1	8300001	8310000	94	1.281349751	72.16868917
1	8350001	8360000	59	1.181261234	89.975112
1	8380001	8390000	86	1.141539887	140.3019744
1	8400001	8410000	75	1.263410149	101.5749129
1	8480001	8490000	60	1.204690799	174.4297329
1	8590001	8600000	36	1.356295932	88.64111498
1	8690001	8700000	64	1.351636638	169.5795587
1	8770001	8780000	91	1.297492437	233.2264808
1	8790001	8800000	104	1.267097557	109.7382708
1	8800001	8810000	87	1.390686445	105.9744483
1	8900001	8910000	85	1.164650304	124.3019744
1	8950001	8960000	88	1.270779703	262.0487805
1	9000001	9010000	56	1.183214253	134.2067364
1	9010001	9020000	33	1.209611547	74.20209059
1	9040001	9050000	62	1.22397774	74.39256678
1	9230001	9240000	42	1.276202045	101.8722416
1	9270001	9280000	54	1.237984774	136.3159117
1	9290001	9300000	55	1.736120158	116.5900116
1	9300001	9310000	34	1.303164835	71.92566783
1	9310001	9320000	34	1.418090967	71.51684088
1	9360001	9370000	43	1.33144188	98.20209059
1	9540001	9550000	46	1.265008077	116.6922184
1	9560001	9570000	34	1.22539155	84.73867596
1	9650001	9660000	48	1.33250775	110.3925668
1	9780001	9790000	64	1.28212266	79.6562137
1	9790001	9800000	48	1.302879727	113.7653891
1	9830001	9840000	55	1.321616754	161.300813
1	9840001	9850000	63	1.367379136	72.92915215
1	9880001	9890000	56	1.260793921	133.3519164
1	9890001	9900000	61	1.477031678	133.203252
1	9920001	9930000	76	1.542363762	163.1869919
1	9940001	9950000	75	1.232320967	74.15606515
1	9990001	10000000	84	1.371221766	196.0603949
1	10010001	10020000	64	1.266747547	73.70499419
1	10030001	10040000	50	1.546663822	104.8641115
1	10170001	10180000	57	1.26800514	66.48581384
1	10290001	10300000	64	1.163397129	69.44792877
1	10360001	10370000	43	1.477243525	112.4552846
1	10450001	10460000	116	1.352953541	82.5203252
1	10690001	10700000	94	1.270900632	81.50832365
1	10700001	10710000	106	1.295054168	303.14518
1	10730001	10740000	99	1.383161734	79.10181959
1	10770001	10780000	61	1.188675005	77.51916376
1	10830001	10840000	102	1.395591803	134.504065
1	10850001	10860000	75	1.268066616	69.96825397

1	10920001	10930000	77	1.334869501	108.7108014
1	10930001	10940000	59	1.334559717	81.43089431
1	10970001	10980000	84	1.291050478	115.8885017
1	11090001	11100000	57	1.328887171	81.2294674
1	11120001	11130000	75	1.257745426	94.34146341
1	11160001	11170000	55	1.157325431	148.4878049
1	11190001	11200000	53	1.267911731	149.2590012
1	11210001	11220000	51	1.731395603	75.45644599
1	11230001	11240000	61	1.329436237	80.89663182
1	11600001	11610000	43	1.347391734	100.7015099
1	11680001	11690000	56	1.501536038	141.2590012
1	11720001	11730000	56	1.173894352	80.34843206
1	11760001	11770000	44	1.267518574	99.86527294
1	11780001	11790000	39	1.143308154	94.25319396
1	12080001	12090000	31	1.271275928	69.65853659
1	12100001	12110000	25	1.218588083	67.91173055
1	12240001	12250000	46	1.356203463	101.8629501
1	12420001	12430000	40	1.411904256	116.2183508
1	13310001	13320000	42	1.500124081	89.43089431
1	13330001	13340000	50	1.477088581	114.1835075
1	13360001	13370000	64	1.320596966	79.50754936
1	13370001	13380000	102	1.193269034	254.5319396
1	13540001	13550000	36	1.401919247	67.74448316
1	13550001	13560000	68	1.360665181	177.7560976
1	13710001	13720000	69	1.301704377	95.81881533
1	14040001	14050000	37	1.310028422	84.27409988
1	14090001	14100000	42	1.310678287	99.54936121
1	14130001	14140000	53	1.277770345	157.1939605
1	14170001	14180000	54	1.208004349	136.6782811
1	14220001	14230000	51	1.518972272	113.0499419
1	14230001	14240000	42	1.18557243	117.1939605
1	14260001	14270000	49	1.360794121	130.7409988
1	14350001	14360000	56	1.181584595	72.53890825
1	14430001	14440000	49	1.172273793	141.2404181
1	14500001	14510000	43	1.453911431	96.36236934
1	14570001	14580000	77	1.330251242	125.9558653
1	15180001	15190000	24	1.197235674	70.00232288
1	15240001	15250000	40	1.376838829	88.41811847
1	15320001	15330000	41	1.388930922	108.3670151
1	15660001	15670000	50	1.421634255	69.80720093
1	15830001	15840000	58	1.276756439	71.59117305
1	15980001	15990000	30	1.19811326	82.23925668
1	16000001	16010000	60	1.258646156	163.5679443
1	16030001	16040000	30	1.182687382	78.99651568
1	16100001	16110000	33	1.234523513	72.49245064
1	16130001	16140000	46	1.483891198	73.71893148
1	16200001	16210000	57	1.412426982	143.0987224
1	16210001	16220000	38	1.218949646	96.68757259
1	17210001	17220000	37	1.471223552	70.44831591
1	17250001	17260000	34	1.325637091	72.57607433
1	17340001	17350000	61	1.245812124	80.92450639
1	17370001	17380000	55	1.196604616	132.0603949
1	17900001	17910000	32	1.388873998	78.64343786

1	17970001	17980000	66	1.305669803	85.55632985
1	18510001	18520000	45	1.341893092	69.40766551
1	18630001	18640000	31	1.185291134	71.37746806
1	18940001	18950000	38	1.172154845	87.92566783
1	75280001	75290000	40	1.192205323	90.7224158
1	75340001	75350000	35	1.359711114	68.57142857
1	75470001	75480000	46	1.189373082	125.7421603
1	75690001	75700000	35	1.204572834	98.02555168
1	75760001	75770000	63	1.163427721	182.2810685
1	75850001	75860000	38	1.299341121	68.69221835
1	76070001	76080000	37	1.147152703	91.93031359
1	76220001	76230000	61	1.326023032	71.12195122
1	76520001	76530000	66	1.180577631	180.970964
1	76570001	76580000	45	1.27069077	123.1312427
1	76600001	76610000	67	1.246118966	84.78977933
1	76620001	76630000	58	1.342057118	138.2950058
1	76720001	76730000	42	1.170455177	114.8339141
1	135790001	135800000	40	1.210538168	77.18466899
1	170220001	170230000	49	1.317181665	75.39140534
4	5660001	5670000	112	1.17254796	256.5017422
8	5940001	5950000	27	1.261930865	73.93263647
11	5500001	5510000	50	1.295955641	117.2404181
11	5510001	5520000	46	1.274847767	116.3484321
12	11570001	11580000	72	1.28626435	94.99651568
14	420001	430000	35	1.248034616	95.53542393
14	430001	440000	43	1.240455606	95.07084785
14	470001	480000	56	1.272114488	136.2415796
14	480001	490000	33	1.222302868	82.64808362
14	1110001	1120000	51	1.213192529	119.9721254
14	1340001	1350000	79	1.247582781	67.66388461
14	1370001	1380000	62	1.449939258	134.9221835
14	1530001	1540000	91	1.346505795	243.0011614
14	1610001	1620000	68	1.141416888	83.1358885
14	1690001	1700000	71	1.451389626	90.7456446
14	1700001	1710000	91	1.220174892	127.883856
18	4430001	4440000	99	1.243717995	90.46225319
18	4470001	4480000	122	1.537802315	166.0162602

* population information
- p1, wild HGF from Sudan and Kenya
- p2, domestic HGF for Italian breed Selvatica
- p3, domestic HGF for Italian breed Camosciata
